# Epidemiological trends and risk factors of CKD-T1DM in children and adolescents across 204 countries and territories (1990–2021)

**DOI:** 10.3389/fendo.2025.1551467

**Published:** 2025-03-26

**Authors:** Beiyan Liu, Fengrui Li, Huanxi Cui, Lin Li, Ying Ma, Qizhi Yang, Ying Cui

**Affiliations:** ^1^ Department of Endocrinology, Rizhao Hospital Affiliated to Qingdao University/Rizhao International Heart Hospital, Rizhao, Shandong, China; ^2^ Department of Endocrinology, The First Affiliated Hospital of Xinxiang Medical University, Xinxiang, Henan, China; ^3^ Department of Neurointerventional, The First Affiliated Hospital of Xinxiang Medical University, Xinxiang, Henan, China; ^4^ Department of Neurology, Rizhao Hospital Affiliated to Qingdao University/Rizhao International Heart Hospital, Rizhao, Shandong, China; ^5^ Department of Intensive Care Unit, Rizhao People’s Hospital, Rizhao, Shandong, China; ^6^ Department of General Surgery, Northern Jiangsu People’s Hospital Affiliated to Yangzhou University, Yangzhou, Jiangsu, China

**Keywords:** type 1 diabetes mellitus, chronic kidney disease, children and adolescents, global burden of disease, epidemiology

## Abstract

**Background:**

Global T1DM incidence in children and adolescents is rising, leading to Chronic Kidney Disease due to Type 1 Diabetes Mellitus (CKD-T1DM), a critical public health concern. Severe cases evolve into end-stage kidney disease (ESKD), requiring dialysis or transplantation, severely impacting quality of life and imposing substantial burdens.

**Methods:**

This study used Global Burden of Disease (GBD) data to analyze global and regional CKD-T1DM incidence, prevalence, mortality, and Disability-Adjusted Life Years (DALYs) rates in children and adolescents (1990-2021). It calculated age-standardized ratios and estimated annual percentage change (EAPC), presenting findings via maps and comparing age-specific burdens and mortality patterns.

**Findings:**

From 1990 to 2021, CKD-T1DM prevalence and incidence in children and adolescents increased globally, while mortality and DALYs declined. Middle SDI (Socio-Demographic Index) nations saw a surge in new cases, contrasting with High SDI countries’ success in reducing DALYs. Male incidence and DALYs were higher than those of females, with notable rises in Eastern Europe, Central Latin America, and Central Europe, and drops in East Asia. The 10-14 age group exhibited higher incidence, and the 15-19 age group higher DALYs.

**Interpretation:**

Global CKD-T1DM management in children and adolescents faces challenges. Future research should focus on SDI-specific needs, resource allocation, public awareness, and community health education. Early detection and comprehensive health protection are crucial, especially in middle and low SDI countries and high-incidence areas.

## Introduction

Type 1 diabetes mellitus (T1DM) is an autoimmune disease mainly affecting children and adolescents. It results from the destruction of pancreatic β-cells, leading to insulin deficiency, chronic hyperglycemia, and severe complications such as chronic kidney disease (CKD). Chronic kidney disease due to type 1 diabetes mellitus (CKD-T1DM) is marked by reduced glomerular filtration rate and proteinuria. This condition can progress to end-stage kidney disease (ESKD), necessitating dialysis or kidney transplantation (1). This condition severely impacts quality of life and elevates the risks of cardiovascular events (2) and ESKD, significantly affecting individuals, families, and socio-economic aspects.

The incidence of T1DM in children and adolescents has been rising annually, with over 1.2 million patients reported in 2021 and more than 98,200 new cases each year. In 2019, the global prevalence of diabetes was 463 million individuals. Projections suggest a 25% increase in diabetes cases by 2030 and a 51% increase by 2045, which will result in direct healthcare costs surpassing 1 trillion dollars (3). The prevalence of chronic kidney disease among patients with type 1 diabetes mellitus is approximately 30-40% and significantly increases with the duration of the disease (4). There are substantial variations in prevalence across different regions and countries (5), potentially influenced by genetic, environmental, and healthcare level factors. Advances in early diagnosis and treatment in developed countries have extended survival, but have also increased the risk of CKD.

Although there have been advancements in the management and treatment of type 1 diabetes mellitus, the incidence of chronic kidney disease remains high, especially among children and adolescents. Known risk factors include hyperglycemia, hypertension, dyslipidemia, genetic factors, and obesity (6). A Mendelian randomization study identified the percentage of eosinophils as a potential risk factor for T1DM and CKD (7). Novel biomarkers such as TWEAK (TNF-like weak inducer of apoptosis) and KIM-1 (Kidney injury molecule - 1) offer new tools for early diagnosis (8). In addition to traditional glucose control and blood pressure management, novel drugs like SGLT2 inhibitors and GLP-1 receptor agonists have significantly reduced CKD progression (9). Despite research advancements, studies on CKD-T1DM in children and adolescents face multiple challenges: small sample sizes limit generalizability and reliability; long-term follow-up is complex, with poor patient compliance (10), making data collection difficult; treatment options are limited, and there is a lack of large-scale, multi-center clinical trial data; mental health and social support are crucial for disease management and prognosis, but related research is scarce.

The burden of T1DM and CKD in children and adolescents is escalating annually, posing a significant global public health concern (11). Early screening, lifestyle interventions, and new drugs show promise in slowing disease progression. However, these approaches face challenges, including small sample sizes, difficulties with long-term follow-up, and limited treatment options. Future research requires larger multi-center studies to achieve a comprehensive understanding of the mechanisms underlying CKD-T1DM in children and adolescents and to establish effective management strategies. Research on the disease burden of CKD-T1DM in children and adolescents is conducted at global, regional, and national levels. These studies aim to evaluate the disease burden, identify high-risk groups, inform policy decisions, and promote research collaborations. Ultimately, they provide scientific evidence and effective strategies to improve the management of CKD-T1DM in this demographic.

## Methods

### Study population

This study included all children and adolescents diagnosed with chronic kidney disease due to T1DM. Although there is no universal definition for CKD-T1DM, previous studies have defined it in children and adolescents as those aged 10-19 who show decreased glomerular filtration rate or impaired renal function. This age-grouping system aligns with the age categorizations in the Global Burden of Disease (GBD) database, facilitating data analysis and comparison. Additionally, the 10-19 age range marks a period of profound endocrine system changes, potentially elevating the risk of chronic complications in T1DM patients, particularly nephropathy. The onset of chronic diseases during this period can markedly affect future health and socioeconomic outcomes, underscoring the significant public health implications of research and interventions targeting this age group. In line with the aforementioned rationale and extant research, the study population for this investigation includes children and adolescents with CKD-T1DM who are aged 10-19.

### Data collection

The diagnosis of chronic kidney disease due to diabetes mellitus type 1 in this study corresponds to the ICD-10 code “E10.2”. The data were obtained from the Global Health Data Exchange (GHDx) (http://ghdx.healthdata.org/). We adhered to the Guidelines for Accurate and Transparent Health Estimates Reporting for cross-sectional studies.

The search parameters included:

- Cause: “chronic kidney disease due to type 1 diabetes mellitus”
- Measures: “incidence, prevalence, deaths, DALYs (Disability-Adjusted Life Years)”
- Location: “all locations”
- Years: “1990–2021”
- Metrics: “number, rate, and percent”
- Sex: “male, female, and both”
- Age: “age-standardized, 10 to 19 years, and corresponding 5-year bands”.

### Statistical analysis

We used data from the GBD database to analyze the incidence, prevalence, mortality, and disability-adjusted life years (DALYs) rates of CKD-T1DM in children and adolescents from 1990 to 2021 at global, regional, and national levels. We also calculated age-standardized rates of incidence (ASIR), prevalence (ASPR), mortality (ASMR), and DALYs (ASDR) for childhood and adolescent CKD-T1DM, and created world maps for each of these measures. The estimated annual percentage change (EAPC) was calculated using the formula 100*(exp(β)-1), with the 95% confidence interval (CI) obtained from the linear regression model. We computed the EAPC for childhood and adolescent CKD-T1DM at the global, regional, and national levels and produced world maps illustrating the EAPC of ASIR, EAPC of ASPR, EAPC of ASMR, and EAPC of ASDR. We categorized CKD-T1DM patients into two age groups: 10-14 years and 15-19 years. We then compared the age distribution of the disease burden among these age groups in 1990 and 2021. Additionally, we compared the age distribution of deaths due to CKD-T1DM in children and adolescents globally between these years. All statistical analyses were conducted using R software, version 4.3.1 (R Core Team). Due to the descriptive nature of this study, the reported figures and trends are derived from observational data, not statistical inference. Observed changes in trends should be interpreted with caution, especially if they are not statistically significant.

## Results

### Trends in global burden

**Figure 1:**
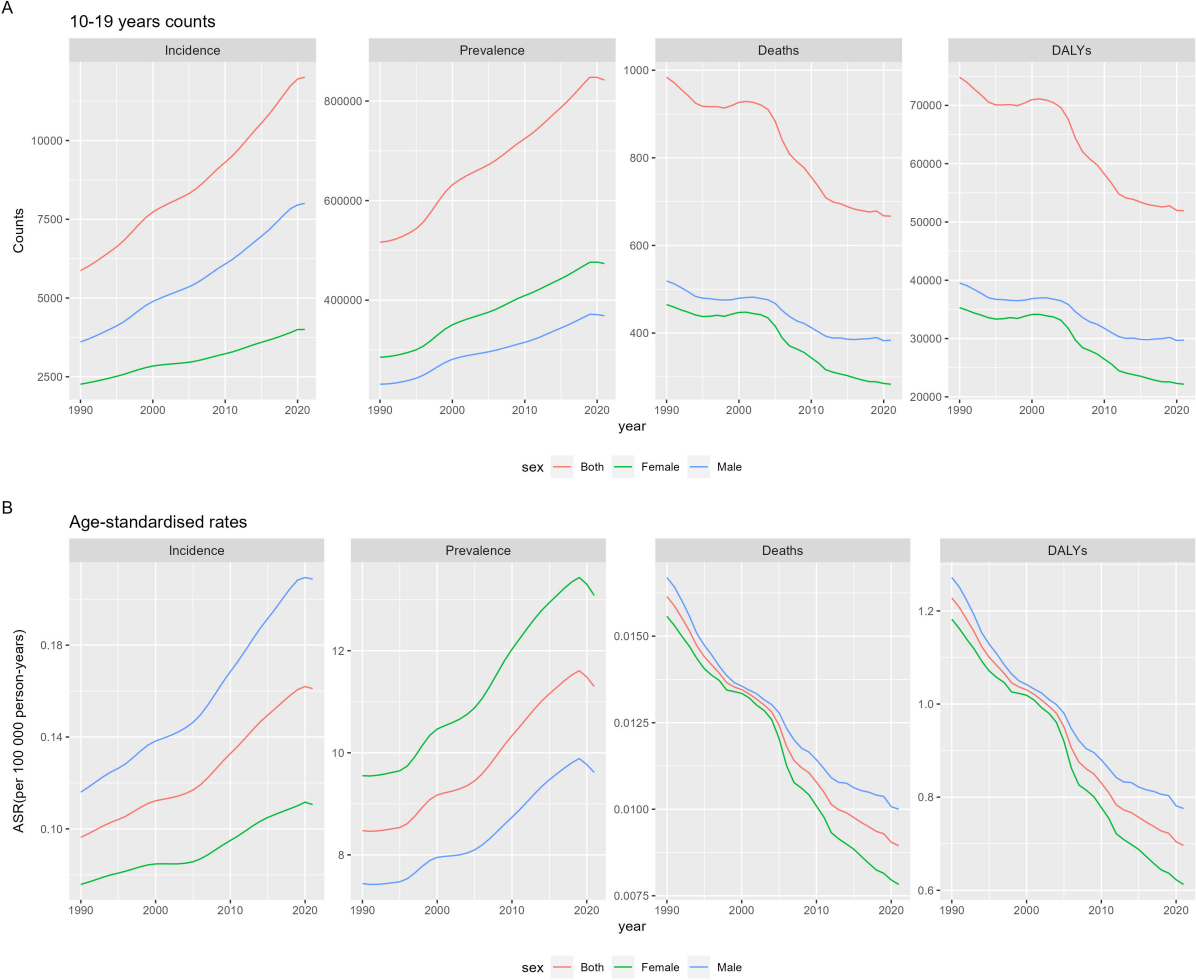
Time and sex trends of chronic kidney disease due to diabetes mellitus type 1 in children and adolescents from 1990 to 2021, including counts **(A)** and age-standardized rates **(B)** in 10-19 years population.

Between 1990 and 2021, global new cases of CKD-T1DM in children and adolescents rose from 5,869 (95% UI: 3,342.0-9,450.6) to 12,008 (95% UI: 7,668.3-17,585.0) (**Figure 1A**). Similarly, global prevalent cases of CKD-T1DM among children and adolescents increased from 516,356 (95% UI: 402,335.8-660,096.6) in 1990 to 842,201 (95% UI: 636,833.0-1,083,908.1) in 2021 (**Appendix, p. 1**). From 1990 to 2021, the number of deaths due to CKD-T1DM among children and adolescents globally showed a declining trend, decreasing from 984 cases (95% UI: 590.3-1473.0) in 1990 to 667 cases (95% UI: 380.7-1016.7) in 2021. The DALYs due to CKD-T1DM also showed a declining trend, decreasing from 74,823 cases (95% UI: 45,349.6-110,532.4) in 1990 to 51,922 cases (95% UI: 30,201.7-77,279.1) in 2021 (**Appendix, p. 2**). The ASIR of CKD-T1DM rose from 0.1 (0.1-0.2) to 0.2 (0.1-0.2) per 100,000 between 1990 and 2021. Additionally, the ASPR exhibited an upward trend, rising from 8.5 (6.6-10.9) per 100,000 in 1990 to 11.3 (8.5-14.5) per 100,000 in 2021. Conversely, both the ASMR and ASDR generally displayed decreasing trends, with the ASDR decreasing from 1.2 (0.7-1.8) per 100,000 in 1990 to 0.7 (0.4-1.0) per 100,000 in 2021 (**Figure 1B**, **Appendix, pp. 3-4**).

From 1990 to 2021, males exhibited higher incidence, mortality, and DALYs for CKD-T1DM than females. In 2021, the incidence of CKD-T1DM in males was 2.0 times higher than in females (8,006.6 (5,237.4-11,554.4) vs 4,001.4 (2,371.8-6,049.0)). Additionally, male deaths were 1.4 times higher (383.5 (213.4-582.6) vs 283.3 (162.2-432.4)), and male DALYs were 1.3 times higher (29,752.5 (17,018.9-44,297.0) vs 22,170.0 (13,075.8-33,130.8)). During the period from 1990 to 2021, the ASIR and ASPR for CKD-T1DM in males and females exhibited contrasting trends. In 2021, male ASIR was 2.0 times higher than female ASIR [0.2 (0.1-0.3) per 100,000 vs 0.1 (0.1-0.2) per 100,000], while female ASPR was 1.4 times higher than male ASPR [13.1 (9.7-17.2) per 100,000 vs 9.6 (7.4-12.1) per 100,000]. The ASMR and ASDR showed identical decreasing trends, yet male indicators consistently exceeded those of females. For example, in 2021, male ASDR was 1.3 times higher than female ASDR [0.8 (0.4-1.2) per 100,000 vs 0.6 (0.4-0.9) per 100,000].

### Trends in regional burden

When calculated by Socio-Demographic Index (SDI) quintiles (**Table 1**), the incidence of CKD-T1DM has been increasing across all regions globally. Middle SDI countries experienced the highest increase in age-standardized incidence, with an EAPC of ASIR of 2.24 (95% CI 2.13-2.34). The age-standardized incidence increased from 0.1 (95% UI 0.1-0.2) per 100,000 in 1990 to 0.2 (95% UI 0.1-0.3) per 100,000 in 2021. The smallest increase occurred in High SDI countries, which had an EAPC of ASIR of 0.39 (95% CI 0.31-0.48). When calculated by SDI quintiles, the DALYs for CKD-T1DM have generally shown a decreasing trend across all regions globally. The most significant decrease was observed in High SDI countries, with an EAPC of ASDR of -2.77 (-3.01 - -2.53). The age-standardized DALYs rate decreased from 0.2 (0.1-0.3) per 100,000 in 1990 to 0.1 (0.1-0.1) per 100,000 in 2021. Low-middle SDI countries showed the smallest decrease, with an EAPC of ASDR of -1.02 (-1.08 - -0.96) and an age-standardized DALYs rate dropping from 0.8 (0.4-1.2) per 100,000 in 1990 to 0.6 (0.3-0.9) per 100,000 in 2021 (**Appendix, p. 5**).

**Table 1:** Region and sex trends of chronic kidney disease due to diabetes mellitus type 1 in children and adolescents from 1990 to 2021.

Location	Sex	Incidence(95%UI)		Incidence(95%UI)			DALY(95%UI)		DALY(95%UI)		
		Case,1990	ASIR(per100000),	Case,2021	ASIR(per100000),	EAPC of ASIR(95%CI),	Case,1990	ASDR(per100000),	Case,2021	ASDR(per100000),	EAPC of ASDR(95%CI),
			1990		2021	1990-2021		1990		2021	1990-2021
Global	Both	5868.8 (3342-9450.6)	0.1 (0.1-0.2)	12008 (7668.3-17584.9)	0.2 (0.1-0.2)	1.78 (1.69-1.87)	74822.5 (45349.6-110532.4)	1.2 (0.7-1.8)	51922.5 (30201.7-77279.1)	0.7 (0.4-1)	-1.88 (-1.94–1.81)
	Male	3605.1 (2038.8-5686.9)	0.1 (0.1-0.2)	8006.6 (5237.4-11554.4)	0.2 (0.1-0.3)	2.04 (1.95-2.13)	39526 (22746.2-59749.3)	1.3 (0.7-1.9)	29752.5 (17018.9-44297)	0.8 (0.4-1.2)	-1.58 (-1.65–1.51)
	Female	2263.6 (1240.6-3705.2)	0.1 (0-0.1)	4001.4 (2371.8-6049)	0.1 (0.1-0.2)	1.29 (1.19-1.38)	35296.6 (21400.8-52996.5)	1.2 (0.7-1.8)	22170 (13075.8-33130.8)	0.6 (0.4-0.9)	-2.23 (-2.35–2.12)
High SDI	Both	536.3 (230.3-1073.8)	0.1 (0-0.1)	586.4 (252.4-1123.9)	0.1 (0-0.2)	0.39 (0.31-0.48)	1535.3 (974.2-2227)	0.2 (0.1-0.3)	676.4 (436.7-969)	0.1 (0.1-0.1)	-2.77 (-3.01–2.53)
	Male	536.3 (230.3-1073.8)	0.1 (0-0.1)	586.4 (252.4-1123.9)	0.1 (0-0.2)	0.39 (0.31-0.48)	800 (502.5-1177.4)	0.2 (0.1-0.3)	355.7 (226.7-533.5)	0.1 (0.1-0.1)	-2.79 (-3.08–2.5)
	Female	171.6 (67.6-354.3)	0 (0-0.1)	211.8 (85.2-449.4)	0.1 (0-0.1)	0.76 (0.68-0.84)	735.3 (467.1-1060.1)	0.2 (0.1-0.3)	320.8 (205.4-447.2)	0.1 (0.1-0.1)	-2.75 (-2.94–2.56)
High-middle SDI	Both	801.9 (402.3-1474.3)	0.1 (0-0.1)	1141 (618.4-1919.5)	0.1 (0.1-0.2)	1.88 (1.7-2.05)	12598.9 (7500.6-18872.9)	1.2 (0.7-1.8)	4560.8 (2783.3-6797.2)	0.5 (0.3-0.8)	-2.7 (-2.93–2.47)
	Male	471.3 (234.8-867.2)	0.1 (0-0.2)	696.1 (396.3-1168.5)	0.2 (0.1-0.3)	1.9 (1.73-2.07)	6871.1 (3917-10754.5)	1.3 (0.7-2)	2640.7 (1587.7-3963.3)	0.6 (0.3-0.9)	-2.52 (-2.68–2.36)
	Female	330.6 (159.6-607.9)	0.1 (0-0.1)	444.9 (229.4-799.9)	0.1 (0.1-0.2)	1.79 (1.61-1.98)	5727.8 (3404.6-8930.7)	1.1 (0.6-1.7)	1920.1 (1136.6-2913.8)	0.5 (0.3-0.7)	-2.94 (-3.27–2.61)
Middle SDI	Both	2097.1 (1136.2-3529.1)	0.1 (0.1-0.2)	3930.2 (2412.4-5847.7)	0.2 (0.1-0.3)	2.24 (2.13-2.34)	43418.5 (25609.3-63941.4)	2 (1.2-3)	24538.7 (14730-36188.9)	1.1 (0.7-1.7)	-1.81 (-1.88–1.74)
	Male	1229.5 (653.2-2043.1)	0.1 (0.1-0.2)	2518.3 (1564.4-3712.5)	0.2 (0.1-0.3)	2.51 (2.4-2.61)	22678.7 (12721.1-34757.1)	2.1 (1.2-3.2)	13878.2 (8120.1-20586.6)	1.2 (0.7-1.8)	-1.57 (-1.67–1.46)
	Female	867.6 (456.4-1522.2)	0.1 (0-0.1)	1411.9 (814.5-2231)	0.1 (0.1-0.2)	1.77 (1.66-1.88)	20739.8 (12584.1-30600.3)	2 (1.2-2.9)	10660.5 (6323.2-16061.3)	1 (0.6-1.5)	-2.11 (-2.18–2.04)
Low-middle SDI	Both	1641.4 (857.4-2780.2)	0.1 (0.1-0.2)	3764.5 (2118.7-6057.5)	0.2 (0.1-0.3)	1.49 (1.42-1.56)	11417.2 (6388.3-17730.7)	0.8 (0.4-1.2)	12616.2 (7323.4-19157.7)	0.6 (0.3-0.9)	-1.02 (-1.08–0.96)
	Male	988.9 (503.9-1665.3)	0.1 (0.1-0.2)	2484.3 (1475.1-3813.8)	0.2 (0.1-0.3)	1.77 (1.72-1.83)	5246.5 (3011.3-8123.2)	0.7 (0.4-1.1)	6601.8 (3786-10234.9)	0.6 (0.3-0.9)	-0.51 (-0.56–0.46)
	Female	652.5 (313.7-1125.3)	0.1 (0-0.2)	1280.2 (623-2239.3)	0.1 (0.1-0.2)	1 (0.89-1.11)	6170.7 (3477.1-9706.7)	0.8 (0.5-1.3)	6014.4 (3445.2-9043.4)	0.6 (0.3-0.8)	-1.5 (-1.62–1.39)
Low SDI	Both	786.4 (435.4-1391.1)	0.1 (0.1-0.2)	2575.6 (1538.8-4148.6)	0.2 (0.1-0.3)	1.12 (1-1.25)	5787.9 (3097.4-9219.9)	0.9 (0.5-1.4)	9451 (5284.4-15473.9)	0.6 (0.3-1)	-1.54 (-1.66–1.42)
	Male	547.5 (305.6-943.3)	0.2 (0.1-0.3)	1927.2 (1204.2-2993.2)	0.2 (0.2-0.4)	1.4 (1.27-1.53)	3894.5 (2002.6-6280.9)	1.2 (0.6-1.9)	6230.3 (3394-10661.1)	0.8 (0.4-1.4)	-1.53 (-1.65–1.41)
	Female	239 (114.7-451.9)	0.1 (0-0.1)	648.5 (324.6-1184.4)	0.1 (0-0.2)	0.41 (0.29-0.52)	1893.4 (1072.8-2972)	0.6 (0.3-0.9)	3220.8 (1863.2-5055.8)	0.4 (0.2-0.7)	-1.54 (-1.71–1.36)
Andean Latin America	Both	46.9 (10.2-148.7)	0.1 (0-0.3)	108.1 (24.8-300.9)	0.2 (0-0.5)	2.06 (2-2.13)	317.4 (174.7-523.6)	0.6 (0.3-1)	339.4 (169.3-595.2)	0.5 (0.3-0.9)	-0.53 (-0.89–0.17)
	Male	30.2 (7.1-93.6)	0.1 (0-0.4)	68 (16.3-187.5)	0.2 (0-0.5)	1.86 (1.79-1.93)	181.3 (95.4-314.5)	0.7 (0.4-1.2)	198 (94.4-344.7)	0.6 (0.3-1)	-0.45 (-0.83–0.05)
	Female	16.8 (2.6-54.5)	0.1 (0-0.2)	40.1 (7.2-125.7)	0.1 (0-0.4)	2.34 (2.26-2.42)	136.1 (72.6-226.7)	0.5 (0.3-0.9)	141.5 (72-256.2)	0.4 (0.2-0.8)	-0.68 (-1.01–0.35)
Australasia	Both	8.7 (1.2-31.8)	0 (0-0.2)	19 (2.4-68.4)	0.1 (0-0.3)	2.09 (2.03-2.15)	3.1 (1.3-9.3)	0 (0-0.1)	5.1 (1.8-15.8)	0 (0-0.1)	1.4 (1.27-1.54)
	Male	5.3 (0.7-18.1)	0.1 (0-0.2)	11.3 (1.5-38.7)	0.1 (0-0.3)	2.12 (2.04-2.19)	1.4 (0.6-3.6)	0 (0-0)	2.3 (0.8-7.2)	0 (0-0.1)	1.41 (1.28-1.53)
	Female	3.4 (0.4-14.5)	0 (0-0.2)	7.7 (0.9-32.7)	0.1 (0-0.3)	2.05 (2-2.1)	1.7 (0.7-4.9)	0 (0-0.1)	2.8 (1-8.9)	0 (0-0.1)	1.4 (1.26-1.55)
Caribbean	Both	44.2 (14-108.1)	0.1 (0-0.3)	82.2 (28.6-197.4)	0.2 (0.1-0.5)	2.03 (1.94-2.11)	399.5 (222.4-643.3)	1 (0.5-1.5)	527.6 (281-931.9)	1.2 (0.6-2.1)	1.2 (1-1.4)
	Male	23.3 (7.4-56.5)	0.1 (0-0.3)	42.9 (15.5-97.1)	0.2 (0.1-0.4)	1.92 (1.83-2)	298.6 (161.8-480.6)	1.4 (0.8-2.3)	400.1 (207.9-687.4)	1.8 (0.9-3.1)	1.14 (0.92-1.36)
	Female	20.9 (5.9-53.8)	0.1 (0-0.3)	39.3 (12-105.4)	0.2 (0.1-0.5)	2.14 (2.04-2.23)	100.9 (55.6-168.4)	0.5 (0.3-0.8)	127.5 (62.2-240.2)	0.6 (0.3-1.1)	1.24 (1.05-1.42)
Central Asia	Both	298 (109.3-726.6)	0.4 (0.1-0.9)	609.4 (218.4-1435.1)	0.7 (0.2-1.6)	2.21 (1.96-2.45)	142.1 (82.1-228.2)	0.2 (0.1-0.3)	221.1 (125.8-373)	0.3 (0.1-0.4)	1.09 (0.71-1.46)
	Male	160.3 (60.2-357.7)	0.4 (0.1-0.9)	354.7 (138-788.8)	0.8 (0.3-1.7)	2.5 (2.26-2.74)	72.2 (42.3-116.8)	0.2 (0.1-0.3)	115 (64.6-205.2)	0.3 (0.1-0.5)	1.04 (0.66-1.43)
	Female	137.7 (43.6-354.6)	0.4 (0.1-0.9)	254.7 (81.9-640.4)	0.6 (0.2-1.5)	1.82 (1.58-2.07)	69.9 (39.8-112)	0.2 (0.1-0.3)	106.2 (60.1-184.5)	0.2 (0.1-0.4)	1.13 (0.76-1.5)
Central Europe	Both	100.2 (40-217.5)	0.1 (0-0.2)	122.7 (55.1-241.8)	0.2 (0.1-0.3)	2.6 (2.54-2.66)	134.6 (84-204.6)	0.1 (0.1-0.2)	72.8 (39.8-123.8)	0.1 (0.1-0.2)	-0.14 (-0.23–0.04)
	Male	59.8 (23.8-132.1)	0.1 (0-0.2)	70.8 (31.8-144.5)	0.2 (0.1-0.4)	2.49 (2.42-2.57)	79.2 (49.5-123.1)	0.1 (0.1-0.2)	43.5 (22.8-78.4)	0.1 (0.1-0.2)	-0.07 (-0.17-0.04)
	Female	40.4 (14.5-90.9)	0.1 (0-0.2)	51.8 (22.4-105.5)	0.2 (0.1-0.3)	2.74 (2.67-2.81)	55.4 (33.9-82.7)	0.1 (0.1-0.1)	29.3 (16.9-48.3)	0.1 (0-0.1)	-0.25 (-0.35–0.15)
Central Latin America	Both	306.7 (131.6-576.1)	0.1 (0.1-0.3)	732 (406.2-1203.4)	0.3 (0.2-0.5)	2.63 (2.48-2.78)	787.6 (443.2-1282.5)	0.4 (0.2-0.6)	828.1 (457.6-1341.8)	0.3 (0.2-0.5)	0.29 (-0.11-0.69)
	Male	208.6 (92.7-383.9)	0.2 (0.1-0.3)	527.1 (297.5-848.6)	0.4 (0.2-0.7)	2.72 (2.55-2.89)	335.5 (189.4-534.7)	0.3 (0.2-0.5)	432.3 (239.7-708)	0.3 (0.2-0.6)	1.11 (0.62-1.61)
	Female	98.1 (38.9-197.8)	0.1 (0-0.2)	204.8 (104.3-358.4)	0.2 (0.1-0.3)	2.33 (2.2-2.45)	452.1 (246.8-762.9)	0.4 (0.2-0.7)	395.8 (215.4-661.9)	0.3 (0.2-0.5)	-0.45 (-0.77–0.13)
Central Sub-Saharan Africa	Both	75.1 (17.4-230.4)	0.1 (0-0.3)	273.5 (65.4-818.8)	0.1 (0-0.4)	1.1 (0.93-1.27)	505.3 (247-882.9)	0.7 (0.3-1.2)	1146.7 (575.2-2154.4)	0.6 (0.3-1.1)	-0.45 (-0.52–0.39)
	Male	55.8 (13.1-174.9)	0.2 (0-0.5)	207.3 (50.7-594.8)	0.2 (0.1-0.6)	1.26 (1.1-1.41)	353.6 (170.8-651.1)	1 (0.5-1.8)	783.6 (374.8-1558.4)	0.8 (0.4-1.6)	-0.58 (-0.64–0.51)
	Female	19.3 (3.2-70.3)	0.1 (0-0.2)	66.3 (12.5-231)	0.1 (0-0.2)	0.6 (0.39-0.81)	151.7 (76.7-255.3)	0.4 (0.2-0.7)	363 (177.3-638.8)	0.4 (0.2-0.7)	-0.18 (-0.26–0.11)
East Asia	Both	617 (233.8-1304.3)	0 (0-0.1)	529.4 (214.3-1072.6)	0.1 (0-0.1)	0.44 (0.18-0.69)	38503.2 (22601.4-58744.3)	2.8 (1.7-4.3)	9963.5 (5830.1-14963.2)	1 (0.6-1.6)	-3.49 (-3.66–3.32)
	Male	358.7 (133.9-741.5)	0.1 (0-0.1)	334.2 (127.2-681.1)	0.1 (0-0.1)	0.49 (0.24-0.74)	21061.7 (11612.5-33032.2)	3 (1.7-4.7)	5964.7 (3373-9230.7)	1.2 (0.7-1.8)	-3.28 (-3.41–3.14)
	Female	258.3 (96.5-562.2)	0 (0-0.1)	195.2 (73.1-399.2)	0 (0-0.1)	0.28 (0.01-0.54)	17441.6 (10222.3-26983.3)	2.6 (1.5-4.1)	3998.8 (2333.8-6040.2)	0.9 (0.5-1.3)	-3.79 (-4.03–3.55)
Eastern Europe	Both	254 (121.5-470.3)	0.1 (0.1-0.3)	412.6 (212.7-728.4)	0.3 (0.2-0.5)	2.93 (2.76-3.1)	289.3 (172.2-443)	0.2 (0.1-0.2)	131.2 (82.6-203.2)	0.1 (0.1-0.2)	-1.78 (-1.92–1.64)
	Male	152.6 (72.5-277.2)	0.2 (0.1-0.3)	257.3 (137.5-449.9)	0.4 (0.2-0.7)	3.02 (2.87-3.17)	127 (76-193.1)	0.1 (0.1-0.2)	65.3 (38-105.9)	0.1 (0.1-0.2)	-1.44 (-1.59–1.28)
	Female	101.4 (45.7-191.9)	0.1 (0-0.2)	155.3 (71.8-278)	0.2 (0.1-0.4)	2.76 (2.56-2.96)	162.3 (95.1-248.4)	0.2 (0.1-0.3)	65.9 (41.6-97)	0.1 (0.1-0.1)	-2.08 (-2.23–1.94)
Eastern Sub-Saharan Africa	Both	333.5 (166.1-644.4)	0.1 (0.1-0.2)	1104.2 (617.5-1922.6)	0.2 (0.1-0.3)	1.27 (1.1-1.44)	4277.2 (2188.6-6798.4)	1.6 (0.8-2.6)	6277.7 (3353-10437.3)	1 (0.6-1.7)	-1.93 (-2.07–1.78)
	Male	269.8 (139.5-501.7)	0.2 (0.1-0.4)	959.6 (544.4-1670.7)	0.3 (0.2-0.6)	1.53 (1.36-1.69)	2900.5 (1395.8-4825.5)	2.2 (1.1-3.7)	4293.5 (2248-7382.6)	1.4 (0.7-2.4)	-1.85 (-1.99–1.71)
	Female	63.7 (24.7-142.5)	0 (0-0.1)	144.6 (57.3-309.1)	0 (0-0.1)	-0.15 (-0.36-0.06)	1376.6 (741.6-2210.1)	1.1 (0.6-1.7)	1984.3 (1092.1-3199.7)	0.7 (0.4-1.1)	-2.1 (-2.31–1.89)
High-income Asia Pacific	Both	105.3 (34.7-250.2)	0.1 (0-0.2)	57.8 (20.4-127.3)	0.1 (0-0.1)	-0.3 (-0.44–0.16)	219.2 (136.8-324.6)	0.1 (0.1-0.2)	58.2 (37.3-86.7)	0.1 (0-0.1)	-2.37 (-2.56–2.19)
	Male	71.6 (24.1-169.1)	0.1 (0-0.2)	38.4 (13.9-85)	0.1 (0-0.2)	-0.28 (-0.45–0.12)	115.3 (69.2-177.5)	0.1 (0.1-0.2)	27.4 (17.2-41.5)	0.1 (0-0.1)	-2.58 (-2.8–2.36)
	Female	33.7 (9.1-81.4)	0 (0-0.1)	19.4 (6-44.2)	0 (0-0.1)	-0.34 (-0.46–0.23)	103.9 (66.7-155.9)	0.1 (0.1-0.2)	30.7 (20-45.1)	0.1 (0-0.1)	-2.16 (-2.33–1.99)
High-income North America	Both	233.4 (100.5-495)	0.1 (0-0.2)	199.9 (76.5-406.6)	0.1 (0-0.1)	-1.28 (-1.41–1.15)	114 (70.2-172.8)	0 (0-0.1)	108.2 (66-166.3)	0 (0-0.1)	-0.64 (-0.99–0.29)
	Male	175.4 (72.3-364.4)	0.1 (0.1-0.3)	148.9 (54-305)	0.1 (0-0.2)	-1.24 (-1.36–1.11)	47.8 (28.6-72.5)	0 (0-0.1)	46.2 (28.3-71.8)	0 (0-0.1)	-0.77 (-1.18–0.36)
	Female	58 (18.6-124.8)	0.1 (0-0.1)	51 (15.8-113.5)	0 (0-0.1)	-1.39 (-1.62–1.15)	66.2 (41.5-98.6)	0.1 (0-0.1)	62 (37.7-94.5)	0 (0-0.1)	-0.54 (-0.89–0.19)
North Africa and Middle East	Both	450.4 (176.3-869.9)	0.1 (0-0.2)	1105.5 (456.9-2201.4)	0.2 (0.1-0.3)	1.81 (1.68-1.94)	872.1 (472.9-1453.5)	0.2 (0.1-0.3)	1087.2 (567.6-1832)	0.2 (0.1-0.3)	-0.24 (-0.29–0.19)
	Male	202.8 (74.2-413.4)	0.1 (0-0.2)	541.2 (221.8-1072.6)	0.2 (0.1-0.3)	2.16 (1.96-2.35)	406.9 (219.4-723.1)	0.2 (0.1-0.3)	512.9 (270.4-848.7)	0.2 (0.1-0.3)	-0.31 (-0.36–0.25)
	Female	247.7 (92.9-490.5)	0.1 (0-0.2)	564.4 (228.8-1135.8)	0.2 (0.1-0.4)	1.52 (1.43-1.61)	465.2 (250.6-758.3)	0.2 (0.1-0.3)	574.4 (307.6-975.5)	0.2 (0.1-0.3)	-0.17 (-0.23–0.1)
Oceania	Both	9.1 (2.7-24.4)	0.1 (0-0.3)	23.4 (6.4-72.1)	0.1 (0-0.4)	0.95 (0.83-1.06)	188.6 (86.6-322.9)	2.2 (1-3.8)	484 (261.2-828.6)	3 (1.6-5.2)	0.82 (0.63-1.02)
	Male	5 (1.3-13.6)	0.1 (0-0.3)	14.1 (3.5-40.9)	0.2 (0-0.5)	1.22 (1.1-1.34)	98.3 (40.2-189.3)	2.2 (0.9-4.3)	237.7 (119-410.4)	2.8 (1.4-4.9)	0.64 (0.5-0.79)
	Female	4.1 (1.3-11.7)	0.1 (0-0.3)	9.3 (2.7-27)	0.1 (0-0.4)	0.56 (0.44-0.68)	90.3 (37.4-168.8)	2.2 (0.9-4.1)	246.3 (126.7-425.3)	3.2 (1.7-5.6)	1.01 (0.77-1.25)
South Asia	Both	1362.3 (621.7-2379.8)	0.1 (0-0.2)	3032 (1589.3-5193.6)	0.1 (0.1-0.3)	1.28 (1.24-1.33)	2880.5 (1735.4-4411.2)	0.2 (0.1-0.3)	4034.5 (2444.2-6200.7)	0.2 (0.1-0.3)	-0.27 (-0.46–0.07)
	Male	839.7 (384-1454.8)	0.1 (0.1-0.2)	2088.6 (1149.9-3466.8)	0.2 (0.1-0.3)	1.59 (1.52-1.66)	1605.9 (968.4-2466.1)	0.2 (0.1-0.3)	2153.1 (1309.4-3464)	0.2 (0.1-0.3)	-0.26 (-0.48–0.05)
	Female	522.6 (238.6-938.3)	0.1 (0-0.1)	943.4 (430-1824.6)	0.1 (0-0.2)	0.72 (0.56-0.89)	1274.6 (766-1969.6)	0.2 (0.1-0.3)	1881.5 (1164-2930.6)	0.2 (0.1-0.3)	-0.26 (-0.44–0.09)
Southeast Asia	Both	906.7 (407.3-1725.3)	0.2 (0.1-0.3)	1819.6 (1023.2-2916.8)	0.3 (0.2-0.4)	2.42 (2.3-2.54)	23044.9 (12946.2-35410.8)	3.8 (2.2-5.9)	22935.3 (13254.6-35477.5)	3.5 (2-5.4)	-0.26 (-0.31–0.2)
	Male	532.9 (239.4-987.7)	0.2 (0.1-0.3)	1092.9 (626.8-1700.4)	0.3 (0.2-0.5)	2.55 (2.4-2.7)	10454.2 (5761.8-16349.7)	3.5 (1.9-5.4)	11929.9 (6671-18625.4)	3.5 (2-5.5)	0.2 (0.09-0.32)
	Female	373.7 (166.1-775.9)	0.1 (0.1-0.3)	726.7 (377.7-1305.9)	0.2 (0.1-0.4)	2.21 (2.13-2.29)	12590.8 (7029.9-19064.4)	4.2 (2.4-6.4)	11005.4 (6164.1-16707.7)	3.4 (1.9-5.2)	-0.67 (-0.74–0.61)
Southern Latin America	Both	27.6 (4.5-85.1)	0.1 (0-0.2)	40.3 (7.7-117.8)	0.1 (0-0.2)	1.3 (1.17-1.42)	73.5 (40.3-119.2)	0.1 (0.1-0.2)	60.9 (35-104.1)	0.1 (0.1-0.2)	-0.33 (-0.56–0.1)
	Male	17.8 (2.8-55.4)	0.1 (0-0.2)	26.5 (5-78.6)	0.1 (0-0.3)	1.43 (1.28-1.59)	35 (19-58.6)	0.1 (0.1-0.2)	30.5 (17.4-52)	0.1 (0.1-0.2)	-0.35 (-0.62–0.08)
	Female	9.9 (1.4-36.9)	0 (0-0.1)	13.9 (2.2-48.7)	0 (0-0.2)	1.02 (0.91-1.13)	38.5 (21.5-63.4)	0.1 (0.1-0.2)	30.4 (17.1-53.1)	0.1 (0.1-0.2)	-0.31 (-0.54–0.08)
Southern Sub-Saharan Africa	Both	54.8 (20.9-135.8)	0.1 (0-0.2)	82.4 (32.7-190.5)	0.1 (0-0.2)	0.55 (0.2-0.89)	101.1 (56.4-163.4)	0.1 (0.1-0.2)	206.8 (111.3-355.6)	0.2 (0.1-0.4)	1.35 (0.81-1.89)
	Male	31.6 (12-73.6)	0.1 (0-0.2)	50.3 (20.2-109.6)	0.1 (0-0.2)	0.76 (0.4-1.13)	55.8 (29.3-93.7)	0.2 (0.1-0.3)	128.9 (67.3-224.9)	0.3 (0.2-0.5)	1.33 (0.78-1.89)
	Female	23.3 (8.5-62.4)	0.1 (0-0.2)	32.1 (11.6-78.7)	0.1 (0-0.2)	0.18 (-0.13-0.5)	45.3 (25.8-75)	0.1 (0.1-0.2)	77.9 (42.3-135.5)	0.2 (0.1-0.3)	1.35 (0.73-1.98)
Tropical Latin America	Both	139.4 (48.3-297.6)	0.1 (0-0.2)	193.3 (71.5-377.2)	0.1 (0-0.2)	1.3 (1.21-1.4)	731.1 (411.6-1159.1)	0.4 (0.2-0.6)	477.7 (271.5-749.4)	0.3 (0.1-0.4)	-0.86 (-1.05–0.66)
	Male	81.1 (27.6-174.7)	0.1 (0-0.2)	114.2 (43.3-219.7)	0.1 (0-0.2)	1.3 (1.17-1.43)	423.7 (238.3-655.4)	0.4 (0.2-0.7)	294.7 (166.2-463.3)	0.3 (0.2-0.5)	-0.5 (-0.76–0.24)
	Female	58.3 (19.2-134.3)	0.1 (0-0.1)	79.1 (27.3-167)	0.1 (0-0.2)	1.28 (1.21-1.34)	307.3 (177-489.5)	0.3 (0.2-0.5)	183 (104.3-285.4)	0.2 (0.1-0.3)	-1.42 (-1.58–1.27)
Western Europe	Both	122.3 (37.6-278.3)	0 (0-0.1)	165.4 (51.5-380.1)	0.1 (0-0.1)	1.28 (1.19-1.37)	104.3 (64-168.7)	0 (0-0.1)	112.5 (62.9-196.9)	0 (0-0.1)	0.63 (0.51-0.76)
	Male	80.3 (23.4-187.9)	0.1 (0-0.1)	107.4 (33.6-235.2)	0.1 (0-0.2)	1.24 (1.14-1.34)	60 (36.2-98.1)	0 (0-0.1)	64.1 (35.6-113.8)	0 (0-0.1)	0.59 (0.45-0.73)
	Female	42 (11.9-103.4)	0 (0-0.1)	58 (17.2-145.7)	0 (0-0.1)	1.35 (1.28-1.43)	44.3 (28-70.1)	0 (0-0)	48.4 (28.3-84.1)	0 (0-0.1)	0.69 (0.58-0.81)
Western Sub-Saharan Africa	Both	373 (198.7-648)	0.1 (0.1-0.3)	1295.5 (743.5-2106.8)	0.2 (0.1-0.3)	0.76 (0.61-0.91)	1134 (620.8-1866.7)	0.5 (0.2-0.7)	2844 (1546.5-4731.1)	0.4 (0.2-0.7)	-0.27 (-0.4–0.14)
	Male	242.6 (130.5-418.8)	0.2 (0.1-0.3)	951 (551.3-1519.7)	0.3 (0.2-0.5)	1.18 (0.98-1.37)	812.1 (436.1-1387)	0.7 (0.4-1.1)	2028.8 (1035.1-3543.5)	0.6 (0.3-1.1)	-0.28 (-0.39–0.16)
	Female	130.4 (62.6-236.4)	0.1 (0-0.2)	344.5 (159.6-622.3)	0.1 (0-0.2)	-0.25 (-0.34–0.16)	321.9 (180-517.1)	0.3 (0.1-0.4)	815.2 (459.9-1332.2)	0.2 (0.1-0.4)	-0.32 (-0.48–0.16)

At the regional level, from 1990 to 2021, the global ASIR of CKD - T1DM generally showed an increasing trend. The largest increase was observed in Eastern Europe, rising from 0.1 (0.1 - 0.3) per 100,000 in 1990 to 0.3 (0.2 - 0.5) per 100,000 in 2021, with an EAPC of 2.93 (2.76 - 3.1). In Central Latin America, the ASIR increased from 0.1 (0.1 - 0.3) per 100,000 to 0.3 (0.2 - 0.5) per 100,000, with an EAPC of 2.63 (2.48 - 2.78). In Central Europe, the ASIR increased from 0.1 (0 - 0.2) per 100,000 to 0.2 (0.1 - 0.3) per 100,000, with an EAPC of 2.6 (2.54 - 2.66). The smallest increase was observed in East Asia, rising from 0 (0 - 0.1) per 100,000 in 1990 to 0.1 (0 - 0.1) per 100,000 in 2021, with an EAPC of 0.44 (0.18 - 0.69). Following this, Southern Sub - Saharan Africa changed from 0.08 (0.03 - 0.19) per 100,000 in 1990 to 0.09 (0.04 - 0.22) per 100,000 in 2021, with an EAPC of 0.55 (0.2 - 0.89). In contrast, two regions exhibited a decreasing trend. Notably, High - Income North America saw a decline from 0.10 (0.04 - 0.22) per 100,000 in 1990 to 0.07 (0.03 - 0.15) per 100,000 in 2021, with an EAPC of - 1.28 (- 1.41 to - 1.15) (**Appendix, p. 6**).

At the regional level, from 1990 to 2021, the standardized DALYs for CKD - T1DM globally exhibited a mix of increasing and decreasing trends. Predominantly, a downward trend was observed, while only a small portion showed an upward trend. Notably, the most significant decrease in ASDR was seen in East Asia, where it decreased from 2.8 (1.7 - 4.3) per 100,000 in 1990 to 1 (0.6 - 1.6) per 100,000 in 2021, with an EAPC of - 3.49 (- 3.66 to - 3.32). Furthermore, in High - Income Asia Pacific, there was a shift from 0.1 (0.1 - 0.2) per 100,000 to 0.1 (0 - 0.1) per 100,000, accompanied by an EAPC of - 2.37 (- 2.56 to - 2.19). Conversely, regions experiencing larger increases include Southern Sub - Saharan Africa, where the rate increased from 0.1 (0.1 - 0.2) per 100,000 in 1990 to 0.2 (0.1 - 0.4) per 100,000 in 2021, with an EAPC of 1.35 (0.81 - 1.89). Similarly, in the Caribbean, there was an increase from 1 (0.5 - 1.5) per 100,000 to 1.2 (0.6 - 2.1) per 100,000, with an EAPC of 1.2 (1 - 1.4) (**Appendix, p. 7**).

Overall, a trend emerged showing that male rates of CKD - T1DM were generally higher than those of females across different regions.

### Trends in national burden

**Figure 2:**
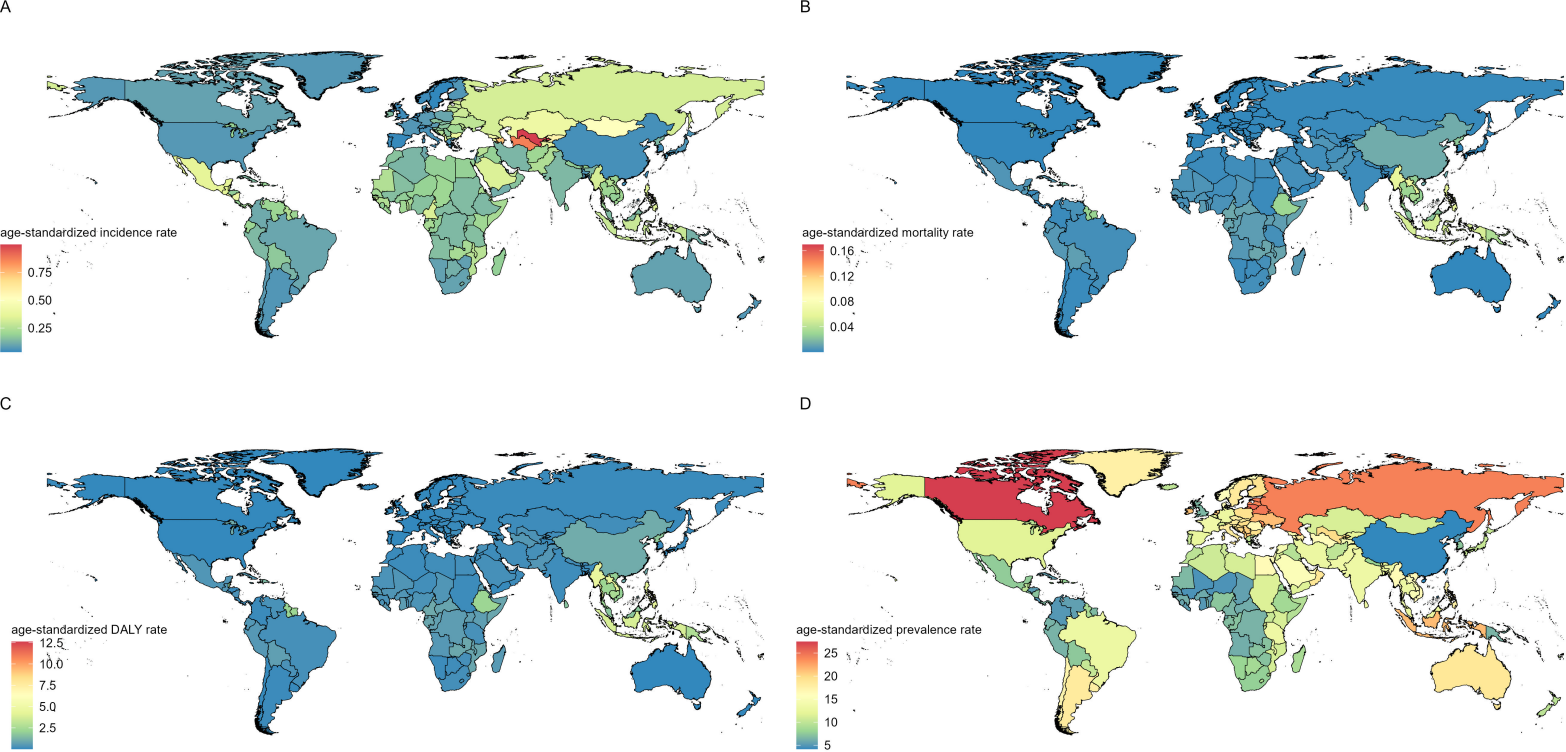
Global disease burden distribution of chronic kidney disease due to diabetes mellitus type 1 in children and adolescents from 1990 to 2021, including age-standardized incidence rate **(A)**, age-standardized mortality rate **(B)**, age-standardized DALY rate **(C)**, and age-standardized prevalence rate **(D)**.

In 2021, India, Indonesia, and Pakistan had the highest number of new cases of CKD - T1DM. India reported 2,129 cases (1127.8 – 3745.1), Indonesia reported 776 cases (397.3 – 1391.2), and Pakistan reported 709 cases (210.0 – 1866.5) (**Figure 2**, **Appendix, pp. 8–17**). The countries with the highest number of deaths were Indonesia with 135 (72.3 – 219.6), China with 127 (73.3 – 191.3), and the Philippines with 74 (43.4 – 112.9) (**Appendix, pp. 18–27**). Uzbekistan had the highest ASIR at 1.0 per 100,000, followed by Turkmenistan at 0.9 per 100,000, and Azerbaijan at 0.7 per 100,000. The lowest rates were in Sweden (0.035 per 100,000), Spain (0.039 per 100,000), and Portugal (0.043 per 100,000) (**Appendix, pp. 28–37**). The countries with the highest ASPR were Canada (27.5 per 100,000 [9.2 – 62.7]), Lithuania (25.4 per 100,000 [8.2 – 60.4]), and Belarus (24.6 per 100,000 [7.2 – 62.4]). The lowest rates were in China (4.2 per 100,000 [2.9 – 6.2]), Mali (4.8 per 100,000 [1.0 – 11.9]), and Niger (4.8 per 100,000 [1.1 – 12.8]) (**Appendix, pp. 38–47**). Niue had the highest ASMR at 0.17 per 100,000, followed by American Samoa and Tokelau, both at 0.12 per 100,000 (**Appendix, pp. 48–57**). The countries with the highest ASDR were Niue (12.6 per 100,000 [5.4 – 24.2]), American Samoa (9.2 per 100,000 [4.9 – 14.6]), and Tokelau (9.1 per 100,000 [4.6 – 16.0]). The lowest rates were in Iceland (0.019 per 100,000 [0.006 – 0.061]), Australia (0.023 per 100,000 [0.006 – 0.083]), and Greenland (0.023 per 100,000 [0.008 – 0.067]) (**Appendix, pp. 58–67**).

**Figure 3:**
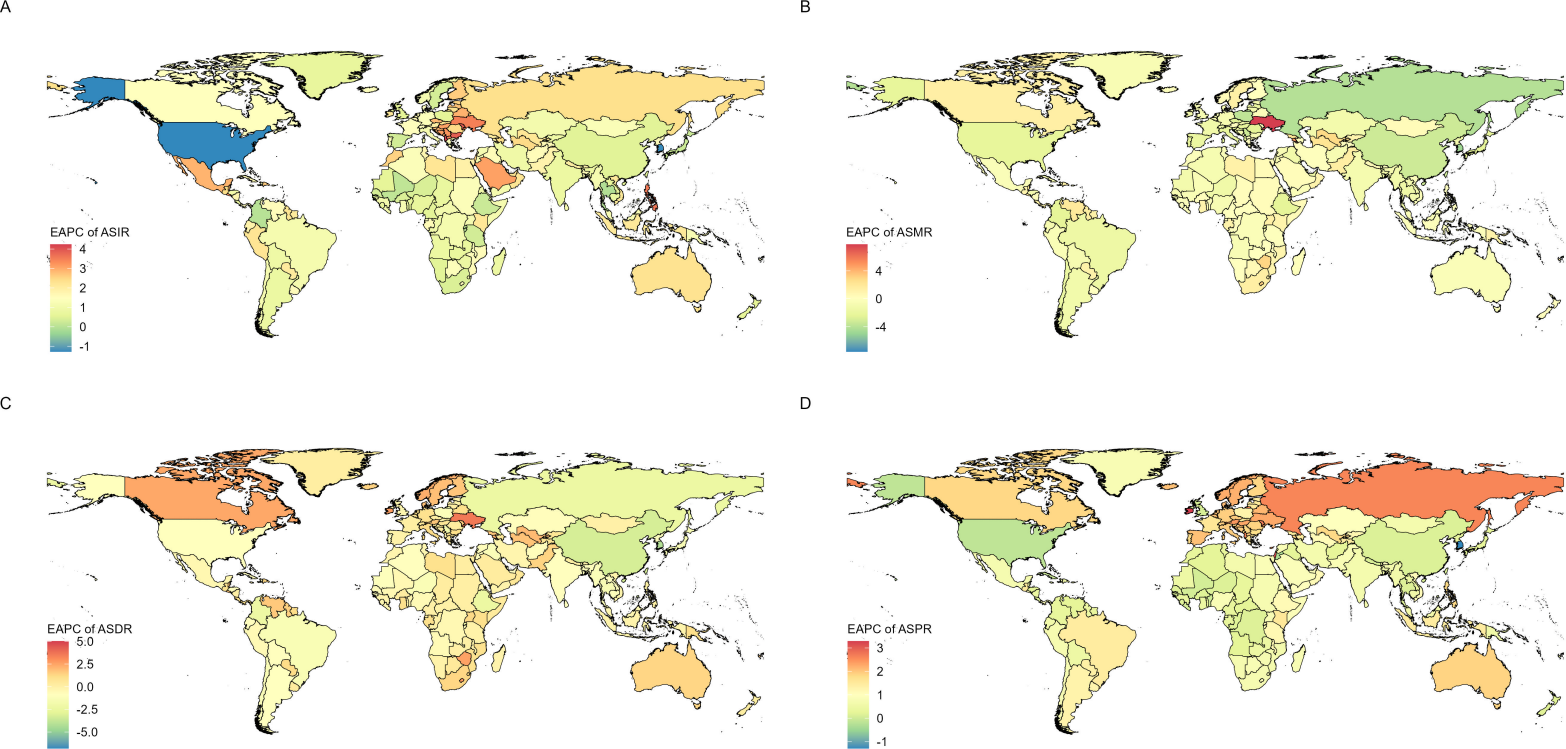
EAPC distribution of the global disease burden of chronic kidney disease due to diabetes mellitus type 1 in children and adolescents from 1990 to 2021, including EAPC of ASIR **(A)**, ASMR **(B)**, ASDR **(C)**, and ASPR **(D)**.

According to the EAPC calculations (**Figure 3**): From 1990 to 2021, the ASIR of CKD - T1DM increased in 198 of the 204 countries and territories. Albania had the largest increase, growing from 0.10 (0.01 – 0.42) per 100,000 in 1990 to 0.37 (0.05 – 1.29) per 100,000 in 2021, with an EAPC of 4.24 (3.56 – 4.92). The Republic of Moldova also saw an increase, rising from 0.08 (0.01 – 0.34) per 100,000 in 1990 to 0.27 (0.03 – 0.91) per 100,000 in 2021, with an EAPC of 3.93 (2.46 – 5.41). Bulgaria showed an increase from 0.12 (0.01 – 0.49) per 100,000 in 1990 to 0.39 (0.05 – 1.41) per 100,000 in 2021, with an EAPC of 3.89 (2.76 – 5.03). Five countries and territories showed a decreasing trend in the ASIR of CKD - T1DM. The most notable decline was in the United States, which decreased from 0.11 (0.04 – 0.23) per 100,000 in 1990 to 0.07 (0.03 – 0.15) per 100,000 in 2021, with an EAPC of -1.28 (-3.95 – 1.47). The Republic of Korea should be “the Republic of Korea” here. It experienced a decrease from 0.07 (0.01 – 0.29) per 100,000 in 1990 to 0.05 (0.00 – 0.21) per 100,000 in 2021, with an EAPC of -1.26 (-3.52 – 1.05). Singapore experienced a decrease from 0.08 (0.01 – 0.33) per 100,000 in 1990 to 0.07 (0.00 – 0.31) per 100,000 in 2021, with an EAPC of -0.30 (-1.63 – 1.05).Notably, in Colombia, the ASIR of CKD - T1DM remained unchanged, with an EAPC of 0.00 (-2.53 – 2.59) (**Appendix, pp. 68–73**).

The ASPR of CKD - T1DM increased in 199 out of 204 countries and territories. Ireland had the largest increase, rising from 7.7 (2.3 – 20.09) per 100,000 in 1990 to 21.2 (7.2 – 48.3) per 100,000 in 2021, with an EAPC of 3.29 (2.7 – 3.89). Similarly, Israel’s rate rose from 6.2 (1.8 – 16.5) to 16.2 (4.9 – 39.3) per 100,000 during the same period, with an EAPC of 3.13 (2.71 – 3.55). The Republic of Moldova also saw an increase, from 10.3 (3.0 – 25.2) to 23.6 (7.1 – 55.7) per 100,000, with an EAPC of 2.7 (2.22 – 3.18). Five countries and territories showed a decreasing trend in the ASPR of CKD - T1DM. The Republic of Korea had the most significant decline, dropping from 11.0 (2.9 – 28.4) per 100,000 in 1990 to 7.4 (1.9 – 20.1) in 2021, with an EAPC of -1.29 (-1.93 – -0.65); Jordan’s rate decreased from 12.8 (3.3 – 35.9) to 11.5 (2.9 – 32.2) per 100,000 during the same period, with an EAPC of -0.33 (-1.67 – 1.03); the United States also experienced a decline from 12.4 (9.0 – 16.6) to 11.8 (8.5 – 15.6) per 100,000, with an EAPC of -0.16 (-0.88 – 0.57) (**Appendix, pp. 74 - 79**).

Among 204 countries and territories globally, the age - standardized DALYs rate of CKD - T1DM exhibits varying trends, with more countries showing an overall increase. The number of countries with rising ASDR is 1.5 times that of those with declining ASDR. Among these, a smaller portion (82) of countries and territories have seen a decline in the ASDR of CKD - T1DM. The largest decline was observed in Seychelles, where the rate decreased from 2.1 (1.2 – 3.4) per 100,000 in 1990 to 0.3 (0.1 – 0.5) per 100,000 in 2021, resulting in an EAPC of - 6.81 (- 9.82 – - 3.7). The Republic of Korea saw a decrease from 0.2 (0.1 – 0.4) per 100,000 in 1990 to 0.1 (0.0 – 0.1) per 100,000 in 2021, with an EAPC of - 4.2 (- 4.35 – - 4.05). China experienced a decline from 2.8 (1.7 – 4.3) per 100,000 in 1990 to 1.0 (0.6 – 1.5) per 100,000 in 2021, with an EAPC of - 3.28 (- 3.81 – - 2.74). The majority (120) of countries have seen an increase in the age - standardized DALYs rate. The largest increase was observed in Niue, where the rate rose from 2.7 (1.3 – 4.8) per 100,000 in 1990 to 12.6 (5.4 – 24.2) per 100,000 in 2021, with an EAPC of 5.02 (3.4 – 6.66). Tokelau experienced an increase from 2.3 (1.1 – 4.4) per 100,000 in 1990 to 9.1 (4.6 – 16.0) per 100,000 in 2021, with an EAPC of 4.41 (2.61 – 6.23). Ukraine saw an increase from 0.03 (0.01 – 0.09) per 100,000 in 1990 to 0.09 (0.04 – 0.21) per 100,000 in 2021, with an EAPC of 3.58 (3.15 – 4.01). Additionally, the age - standardized DALYs rate for CKD - T1DM in two countries (Central African Republic and Congo) remained relatively stable, with an EAPC of 0 (**Appendix, pp. 80 - 85**).

Among the 204 countries and territories worldwide, the ASMR for CKD - T1DM show mixed trends, with some countries experiencing increases while others report decreases. The number of countries reporting a decrease in ASMR is 1.1 times greater than the number of those experiencing an increase. More than half of the countries (109) have shown a decreasing trend in ASMR. The most significant decline occurred in Seychelles, where the ASMR dropped from 0.028 (0.016 – 0.045) per 100,000 in 1990 to 0.003 (0.002 – 0.006) per 100,000 in 2021, corresponding to an EAPC of - 7.58 (- 11.19 – - 3.83). The second - largest decrease was in the Republic of Korea, where the ASMR fell from 0.003 (0.001 – 0.005) per 100,000 in 1990 to 0.001 (0 – 0.001) per 100,000 in 2021, resulting in an EAPC of - 4.78 (- 5 – - 4.57). The remaining 95 countries have shown an increasing trend in ASMR. The largest increase was observed in Ukraine, with an EAPC of 7.79 (6.62 – 8.97); followed by Niue, with an EAPC of 5.02 (3.39 – 6.68) (**Appendix, pp. 86-91**).

### The burden trend by age group

**Figure 4:**
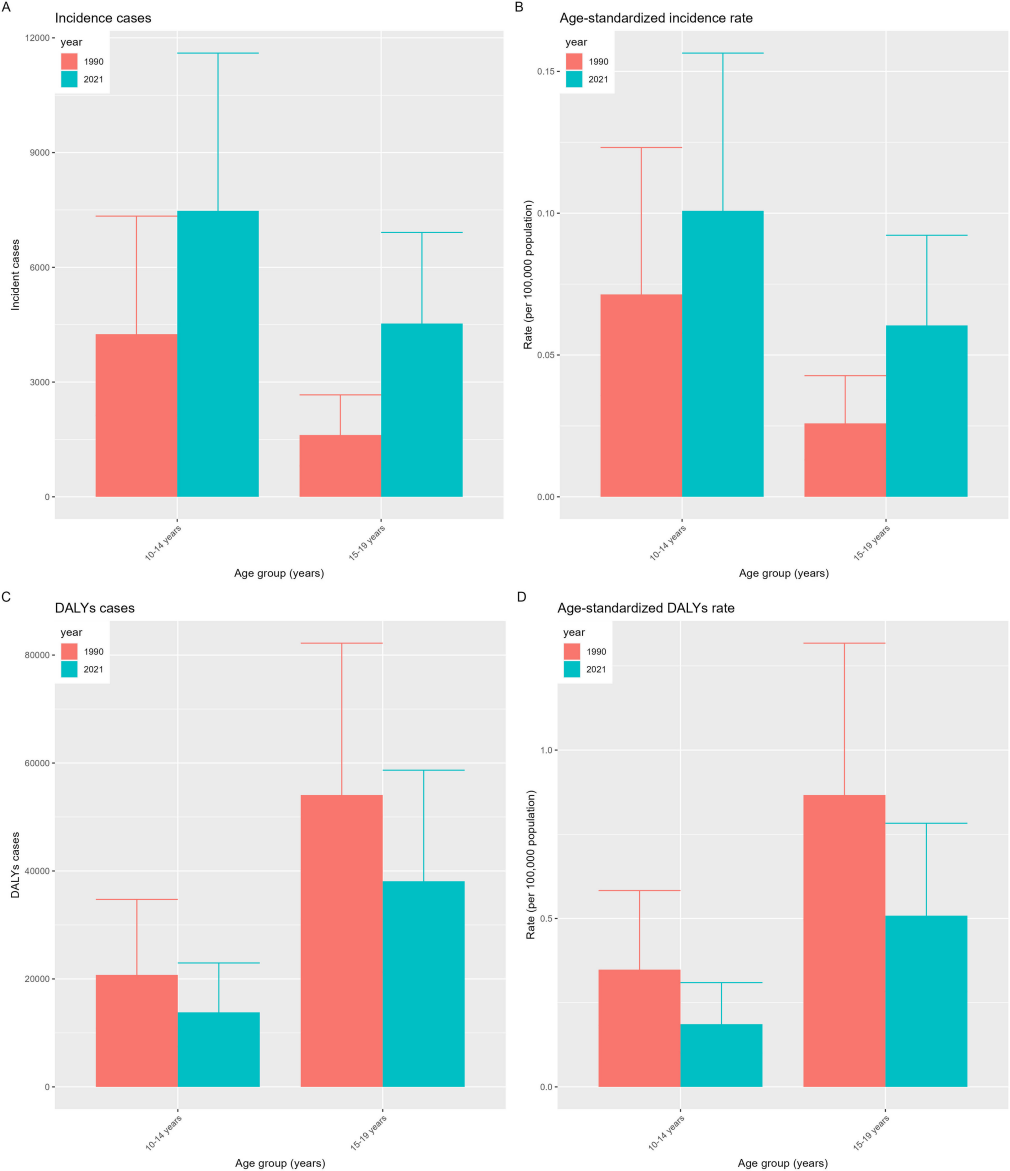
Distribution of disease burden of chronic kidney disease due to diabetes mellitus type 1 in children and adolescents by age group in 1990 and 2021, including incidence cases **(A)**, age-standardized incidence rate **(B)**, DALYs cases **(C)**, and age-standardized DALYs rate **(D)**.

**Figure 4** illustrates a global comparative analysis of incidence rates, DALYs, ASIR, and ASDR for CKD-T1DM among children and adolescents from 1990 to 2021. As depicted in the figure, the incidence and ASIR of CKD-T1DM are relatively higher in the 10-14 age group compared to the 15-19 age group. Conversely, the DALYs and ASDR of CKD-T1DM are relatively higher in the 15-19 age group compared to the 10-14 age group. In summary, the new cases and ASIR of CKD-T1DM in both age groups have increased in 2021 compared to 1990, whereas the DALYs and ASDR have both decreased.

### Contribution proportion of risk factors

**Figure 5:**
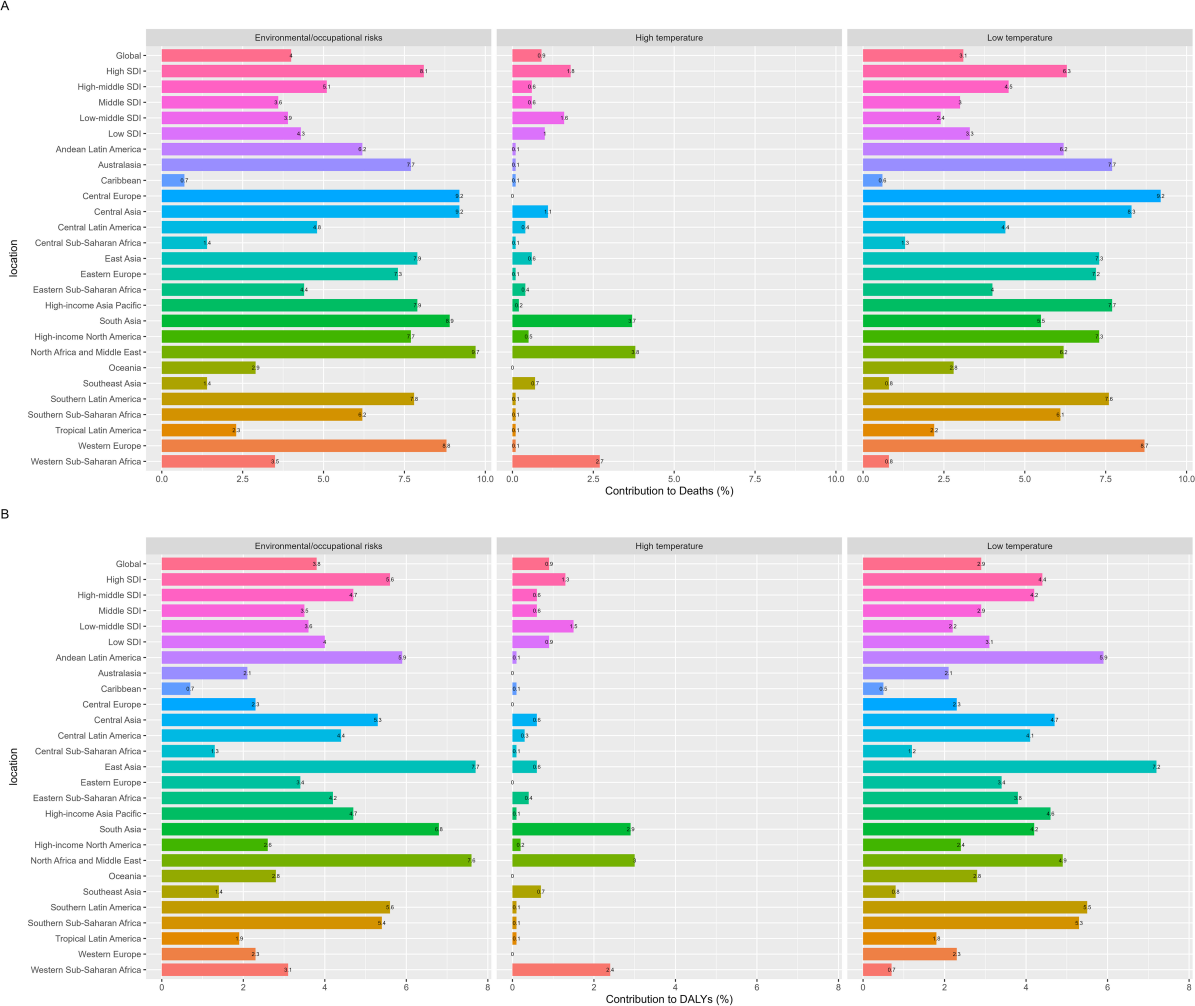
The contribution of risk factors to the mortality **(A)** and DALYs **(B)** of chronic kidney disease due to diabetes mellitus type 1 in children and adolescents globally in 2021.

**Figure 5A** shows the contribution percentages of three risk factors to global deaths from CKD - T1DM among children and adolescents in 2021. Globally, the main factors contributing to CKD - T1DM deaths are environmental/occupational risks (4%), low temperature (3.1%), and high temperature (0.9%). Generally, the contributions of these risk factors vary by the development status of each region.

In high SDI countries, environmental/occupational risks accounted for the highest percentage of deaths at 8.1%, followed by low temperature at 6.3%. Across the 21 global regions, environmental/occupational risks and low temperature are the largest contributors to CKD - T1DM deaths. For example, low temperature contributed 9.2% of deaths in Central Europe and 8.7% in Western Europe, while environmental/occupational risks contributed 9.7% in North Africa and the Middle East and 9.2% in Central Asia. In 2021, among the 10 - 19 age group globally, there were minimal gender differences in the severity of these risk factors, with environmental/occupational risks and low temperature remaining the predominant ones contributing to global deaths (**Appendix details on pp. 92-104**).

Moving on to the contributions to DALYs, **Figure 5B** illustrates the global contributions of the same three risk factors to DALYs for CKD - T1DM in 2021. The primary factors contributing to global DALYs in CKD - T1DM are environmental/occupational risks (3.8%), low temperature (2.9%), and high temperature (0.9%). Similar to the death contributions, the contributions of different risk factors vary by region based on development status.

According to SDI quintiles, environmental/occupational risks and low temperature are the main risk factors for global DALYs in CKD - T1DM, contributing up to 5.6% and 4.4% respectively in high SDI countries. When looking at the 21 global regions, these two factors continue to dominate DALYs contributions. For instance, environmental/occupational risks contributed 7.7% of DALYs in East Asia and 7.6% in North Africa and the Middle East, while low temperature contributed 7.2% in East Asia and 5.9% in Andean Latin America. In 2021, for the global 10 - 19 age group, there were also minimal gender differences in the severity of risk factors, and environmental/occupational risks and low temperature remained the primary contributors to global DALYs (**Appendix details on pp. 105-117**).

## Discussion

T1DM predominantly affects children and adolescents, frequently resulting in chronic complications like CKD-T1DM. It primarily impacts this age group and often leads to chronic complications such as CKD-T1DM. Although treatment has improved, the incidence and prevalence of CKD-T1DM in children and adolescents continue to rise significantly. This increase varies by region and country, likely due to insufficient medical resources and health management. Chronic kidney disease due to type 1 diabetes mellitus (CKD-T1DM) has become a major health threat for children and adolescents. It is associated with a poor prognosis and an increased socioeconomic burden, highlighting the need for global strategies to improve early diagnosis and treatment. Despite its significance, there is limited research on T1DM and chronic kidney disease in adolescents. In - depth analysis of this age group can address existing research gaps. Additionally, public health policies and educational programs aimed at children and adolescents are especially effective, as early intervention can reduce future medical burdens. This study systematically analyzed the global, regional, and national burden of CKD-T1DM in children and adolescents from 1990 to 2021, focusing on incidence, disease burden, and risk factors. It provides scientific evidence for public health policy formulation and has significant public health implications.

### Analysis of global trend changes

Between 1990 and 2021, the global incidence of CKD-T1DM in children and adolescents has continuously increased, showing a sustained high prevalence in this age group. Although the disease burden has increased, the number of deaths and DALYs has decreased, reflecting improvements in treatment effectiveness and survival rates. These changes can be attributed to several factors. Firstly, advancements in diagnostic technology have enhanced the early detection of CKD-T1DM, leading to an increase in reported cases. Secondly, unhealthy lifestyles, including high-sugar diets and lack of exercise, exacerbate the incidence of diabetes and its complications. Additionally, environmental factors and genetic susceptibility play significant roles in the disease prevalence. Advancements in medical treatments, such as insulin therapy and the use of ACE inhibitors and ARBs, have substantially improved patient prognosis. These treatments have reduced mortality rates and lessened the loss of quality of life associated with CKD-T1DM. Gender differences play a significant role in CKD-T1DM. Males have higher incidences, mortality rates, and DALYs compared to females. This may be influenced by physiological differences, social roles, and behaviors, including a greater susceptibility to smoking and stress. With smoking identified as a significant risk factor (12), it has been proven that smoking causes kidney damage through nicotine toxicity, thrombosis, oxidative stress, and the release of inflammatory factors, affecting CKD progression. Smoking is also associated with an increased incidence of albuminuria, nephrosclerosis, and glomerulonephritis (13). Data also shows that females exhibit higher risks in indicators such as ASPR, possibly related to physiological cycles and hormonal changes, affecting disease severity and development speed. Females, poor blood sugar control, elevated body mass index, and albuminuria are associated with a sharp decline in renal function, indicating that females are a risk factor for the estimated Glomerular Filtration Rate (eGFR) trajectory decline, which helps to identify individuals at high CKD risk (14).

To tackle these challenges, a multi-tiered intervention approach is essential. It is crucial to strengthen public health education and initiatives that promote healthy lifestyles. Physical activity has been shown to enhance metabolic status, bone density, cardiorespiratory health, and insulin sensitivity in children with T1DM (15). Early diagnosis is crucial, as many T1DM patients show eGFR abnormalities early on, with a decrease in eGFR during adolescence indicating a higher risk for CKD. Therefore, systematic serum creatinine monitoring should begin at T1DM diagnosis and be conducted regularly (16). The 2018 guidelines from the International Society for Pediatric and Adolescent Diabetes (ISPAD) state that nonproteinuric diabetic kidney disease is indicated by an eGFR below 60 mL/min/1.73 m² and a urinary albumin-creatinine ratio (Ua: CR) of 300 mg/g or lower. This highlights the critical importance of regularly monitoring eGFR. Active and comprehensive treatment from the outset of the disease is recommended to avoid medical complacency, as research shows that children with T1DM may experience acute kidney injury (AKI) initially, predisposing them to CKD (17). According to the Kidney Disease/Improving Global Outcomes (KDIGO) Clinical Practice Guideline, AKI is defined by either an increase in serum creatinine of at least 0.3 mg/dL (≥ 26.5 μmol/L) within 48 hours, or a urine output of less than 0.5 mL/kg/h sustained for over 6 hours. Additionally, advancing and disseminating medical technological innovations is essential to guarantee that patients access efficient treatment and management regimens. The development of personalized drugs, continuous glucose monitoring technologies, and effective patient-physician communication strategies are pivotal for successfully managing diabetes and its associated complications, as highlighted in the 9th Cardiovascular Outcome Trial conference (18). A cross-sectional study in Brazil found that CKD is directly linked to factors like female gender, high HbA1c and uric acid levels, long diabetes duration, elevated systolic blood pressure, and low to mid-economic status (19). Consequently, policymakers must take into account gender disparities and develop tailored prevention and intervention strategies to ensure that all gender groups receive suitable supportive care. By implementing these integrated approaches, we can enhance our ability to control and diminish the global impact of CKD-T1DM, thereby enhancing patients’ quality of life and prognostic outcomes.

### Analysis of the reasons for SDI quintile trend changes

Between 1990 and 2021, the rates of CKD and T1DM among children and adolescents increased worldwide, with different trends observed in various SDI countries and regions. The age-standardized incidence has risen most significantly in Middle SDI countries, while High SDI countries have experienced a more modest rise. The total DALYs for CKD-T1DM have shown a decreasing trend globally, with the most significant reduction in High SDI countries and slower progress in Low-middle SDI countries. These changes are influenced by several factors. First, advancements in medical technology and diagnostic methods have enhanced detection rates. Additionally, improved access to healthcare services in Middle SDI countries has resulted in more timely diagnoses. High SDI countries have seen a smaller increase in incidence due to previously better healthcare coverage and health management measures. High SDI countries have reduced DALYs significantly due to advanced medical technology and effective disease management strategies. These include the use of new drugs, renal replacement therapy, and patient education programs. Low-middle SDI countries, with limited medical resources, have patients who struggle to access timely and effective treatment, resulting in a less significant reduction in DALYs.

To tackle these challenges effectively, a comprehensive and targeted strategy is essential. Firstly, GBD data from 1990 to 2021 indicate that the incidence of T1DM in adolescents in Middle SDI countries has increased the most, with a more severe burden of T1DM (20). Therefore, public health education and health promotion activities should be particularly strengthened in these lower-middle development level countries to enhance public awareness and prevention efforts for long-term CKD-T1DM. For high-risk populations, CKD screening combined with risk stratification and treatment should be immediately implemented, and it is recommended that this be conducted at primary or community healthcare facilities (21). Secondly, the dissemination of medical technologies should be promoted to improve medical facilities and personnel training in Low-middle SDI countries, ensuring patients receive high-quality healthcare services. The International Society of Nephrology recommends using risk scores and clinical judgment, along with point-of-care devices, for screening to identify acute kidney disease. In resource-limited settings, follow-up for persistent kidney disease after discharge should be strengthened (22). Additionally, social determinants contribute to health disparities and inequalities. Data from the T1D Exchange clinic registry show that in poor areas, there is a higher risk of poor glycemic control, dyslipidemia, and hypertension (23). Policymakers should promote international cooperation and assistance to help resource-poor countries improve healthcare conditions, reduce the global burden of CKD-T1DM, and improve patient quality of life and outcomes.

### Analysis of the reasons for regional trend changes

From 1990 to 2021, the global ASIR of CKD-T1DM in children and adolescents generally increased. However, there were notable regional disparities: Eastern Europe, Central Latin America, and Central Europe experienced significant increases, while East Asia and sub-Saharan Africa South saw moderate growth. In contrast, North America experienced a decline. DALYs notably declined in most regions, particularly in East Asia and high-income Asia Pacific, but increased in sub-Saharan Africa South and the Caribbean. DALYs significantly decreased in most regions, especially in East Asia and the high-income Asia-Pacific. In contrast, they increased in sub-Saharan Africa South and the Caribbean. Male incidence rates and DALYs consistently surpassed those of females. The shifts in incidence and DALYs were complex and influenced by several factors. For instance, disparities in medical standards and public health measures between regions resulted in increased incidence rates in Eastern Europe and Central Latin America due to unequal medical resources. On the contrary, North America and East Asia saw declines in both incidence rates and DALYs, thanks to advancements in medical technologies and management practices. Lifestyle and environmental factors significantly impacted these trends. In East Asia, industrialization and urbanization contributed to a rise in diabetes incidence; however, effective interventions helped reduce DALYs. Conversely, sub-Saharan Africa South and the Caribbean experienced increasing DALYs due to issues such as poverty, underdevelopment, limited medical resources, and insufficient public health interventions and research (24).

In response to the global challenge of CKD-T1DM in children and adolescents, a multifaceted approach is advised: Initially, leverage international collaboration to optimize medical resource allocation and formulate targeted public health policies. Experts in nephrology and endocrinology emphasize that continuous glucose monitors (CGMs) are essential for managing glucose levels and predicting risks in T1DM and CKD patients. However, their use is limited by barriers within the healthcare system. Improving collaboration and education among stakeholders, optimizing the allocation of resources, and narrowing the gap in technology usage are crucial (25). Secondly, promote early screening by introducing novel functional biomarkers such as microalbuminuria and KIM-1, which will improve CKD detection rates. The 2018 guidelines from the ISPAD define microalbuminuria as either a urinary albumin-creatinine ratio (UACR) between 30 and 300 mg/g or an albumin excretion rate (AER) between 20 and 200 μg/min. It is considered an early and sensitive marker for the onset of diabetic nephropathy in type 1 diabetes patients. Its occurrence signals a substantial increase in the risk of nephropathy progression. Without appropriate intervention, patients may progress to ESRD. Metabolomics research reveals that histidine phenylalanine, leucine phenylalanine, and tryptophan are strongly correlated with CKD progression (26). Thirdly, adopt multidisciplinary team-based comprehensive management, incorporating lifestyle interventions, psychological counseling, and medication, with a focus on promoting new drugs like SGLT2 inhibitors (27) and GLP-1 receptor agonists, and enhancing high-intensity interval training (28). Intelligent healthcare innovations, such as artificial pancreas, have the potential to enhance glycemic outcomes in T1DM patients (29). Fourthly, implement differentiated strategies to comprehensively enhance disease management and outcomes in impoverished and industrialized regions. a lack of sufficient health check-ups raises the risk of CKD patients advancing to end-stage renal failure due to missed early detection and intervention opportunities. Early warning models for CKD constructed using machine learning algorithms have demonstrated promising performance and could serve as effective screening tools. Promoting these models can facilitate large-scale screening, thereby enhancing patient quality of life and reducing mortality (30). In industrialized regions, prolonged exposure to PM_2.5_ and NO_2_ pollution decelerates eGFR growth and elevates CKD risk. Early air pollution control is essential for lifelong kidney health and mitigating the CKD burden (31). Lastly, the disease burden of diabetic chronic kidney disease in China is escalating, with marked gender disparities, particularly affecting males. Targeted interventions are crucial for mitigating the high disease burden in males (32).

### Analysis of national trend changes

The burden of CKD-T1DM among children and adolescents varies significantly across different regions and over time worldwide (33). In densely populated countries with high rates of T1DM, like India, Indonesia, and Pakistan, the number of new CKD-T1DM cases is significant. However, limited medical resources lead to poorer health outcomes. Indonesia, China, and the Philippines exhibit relatively high mortality rates attributable to population aging, suboptimal diabetes management, and limited renal replacement therapy resources. China has comparatively fewer deaths due to sustained investment and advancements in diabetes and kidney disease management. High incidence rates in Central Asia and the Caucasus region may be associated with genetic factors, environmental conditions, and challenges in chronic disease management. Northern and Western European countries enjoy lower incidence rates owing to advanced healthcare systems and robust public education. From 1990 to 2021, incidence and prevalence rates generally increased worldwide. In contrast, the United States and South Korea have successfully reduced these rates through improved prevention, diagnosis, and treatment strategies. Global management confronts challenges while also benefiting from successful practices and learnable lessons.

Global strategies to address CKD-T1DM in children and adolescents focus on improving early diagnosis and treatment, as well as increasing public awareness about T1DM and its complications, especially in low- and middle-income countries. Strengthening healthcare systems through guaranteed insulin supply, laboratory facilities, and trained medical staff in rural regions can enhance disease management and minimize complications (34). Furthermore, governments must prioritize healthcare worker training to elevate competencies in managing diabetes and kidney diseases. Policies ought to provide additional funding to upgrade medical infrastructure and guarantee patients’ access to essential services. In Japan, characterized by high dialysis usage and a significant presence of diabetic kidney disease patients, the government partners with healthcare providers to offer enhanced support and preventive interventions (35). Moreover, community health initiatives and health education are crucial, fostering healthy lifestyles and mitigating diabetes risk factors. The ECHO T1D program in the U.S. represents an effective tele-education model, aiding primary care providers in managing T1DM and enhancing health outcomes in underserved areas (36). Ultimately, improving healthcare accessibility and quality guarantees that diabetes patients receive holistic care. Encouraging the use of nutritional supplements such as omega-3 fatty acids may aid in better blood glucose management and retard disease advancement (37).

### Analysis of age group trend changes

The global burden of CKD-T1DM in children and adolescents varied by age group and changed over time from 1990 to 2021. The incidence and ASIR were higher among 10-14-year-olds, while DALYs and ASDR were elevated in the 15-19-year-old group. This suggests that diagnosis is easier in children, but adolescents face a greater risk of disease progression and mortality. Although new cases and ASIR increased, DALYs and ASDR decreased, indicating improved treatment efficacy and quality of life due to advancements in medical technology and diabetes management.

Solutions should include enhanced early diagnosis and management of diabetes in children and adolescents, with educational programs commencing at the time of diagnosis to prevent diabetes-related complications in these young populations (38). One effective measure is to promote health education in schools and communities, as early-onset T1DM is an independent risk factor for CKD (39). Therefore, efforts should be made to enhance parental and adolescent awareness of diabetes and its complications. Strengthening the diagnostic and treatment capabilities of pediatric and adolescent endocrinology, using precision medicine’s professional interpretations to ensure the accuracy of diagnostic and treatment decisions, enhance treatment outcomes, and promote the clinical implementation of precision diabetic medicine (40). Enhancing public education and promoting healthy lifestyles, with a focus on reducing obesity and unhealthy dietary habits, which are key risk factors for diabetes, is particularly important. Early CKD risk significantly increases in individuals with high-normal BMI and obesity, particularly those with severe obesity (41). A multicenter study in Brazil revealed that nearly one-quarter of adolescent T1DM patients were overweight or obese (42). Healthy eating plays a crucial role in the management of chronic kidney disease. The distribution of food outlets influences residents’ nutritional intake, and this factor should be considered in formulating public health policies related to food (43). Additionally, policies should provide financial support to improve medical infrastructure and ensure that diabetic patients receive high-quality medical services. Comprehensive intervention strategies can effectively reduce the disease burden and enhance both the quality of life and life expectancy for young patients.

### Analysis of risk factor characteristics

In 2021, environmental/occupational risks and low temperature emerged as primary risk factors in the global disease burden of CKD-T1DM among children and adolescents, significantly impacting mortality and DALYs. The contribution of these risk factors varies by region and country, influenced by climate, industrial activity, labor protection standards, and public health awareness. Gender analysis shows that these risk factors affect adolescent health equally, regardless of gender. This consistency may be related to their lifestyle, occupational exposure, and adaptability to environmental conditions.

Multi-level intervention measures are essential: First, strengthening environmental protection and occupational health management to ensure safe working environments and labor protection. Attention should be paid to health risks including water, sanitation, air pollution, alcohol consumption, and hyperglycemia, with a deeper understanding of the environmental determinants of diabetes to prevent or delay the disease (44). Concurrently, actively investigate the pathophysiological mechanisms of CKD-T1DM, addressing metabolic and hemodynamic disturbances and their interactions that drive diabetic nephropathy development, to formulate corresponding treatment strategies (45). Islet autoimmunity constitutes the initial stage of the disease, influenced by potential triggers like infections, diet, and toxins, necessitating prospective recording in early childhood (46). Secondly, cold can expedite disease onset by affecting insulin resistance and β-cell destruction (47). It is crucial to enhance public health awareness, particularly in cold regions, by educating people on strategies to cope with low temperatures to reduce the risk of diabetes and its complications. Thirdly, we should strengthen the formulation and implementation of health policies to ensure adolescents in high-development index countries and industrialized regions receive health protection and medical support. Early screening and effective therapies have the potential to alter the early course of T1DM (48). Finally, promoting global cooperation to reduce wars, conflicts, and inequalities can enhance the prevention and control levels of diabetes and its complications. Research indicates that the foundation of chronic diseases is established in early life, necessitating early intervention for vulnerable groups such as war-displaced children and children from low socioeconomic backgrounds (49). Comprehensive measures can effectively address the disease burden of CKD-T1DM, thereby improving the health status of children and adolescents.

### Limitations and prospects

This study uses the GBD database to provide a detailed analysis of the disease burden of CKD-T1DM in children and adolescents globally, regionally, and nationally from 1990 to 2021. However, the GBD database faces challenges such as slow updates, limited coverage, and high data access barriers, relying on incomplete data from multiple sources, which can introduce uncertainty in disease burden estimates in low-income countries and regions with underdeveloped data systems. While the GBD is an authoritative source of disease data, this study must acknowledge its limitations.

Looking ahead, we propose several research directions: First, we recommend collecting comprehensive longitudinal data through multicenter collaborative projects. This will provide deeper insights into the developmental trajectories of CKD-T1DM across various cultural and socio-economic contexts. While some countries have up-to-date data, the majority have outdated information, leading to a significant gap in global data collection. This data is crucial for allocating resources globally and supporting public health initiatives (50). Second, developing and validating new models and algorithms to enhance the accuracy and foresight of disease burden predictions, aiding policymakers and public health experts in early intervention. KidneyIntelX™, combining electronic health records and biomarkers, outperforms traditional models in predictive performance, improving the prediction of kidney failure in high-risk patients (51). Third, evaluating new intervention measures, such as interleukin-35 (IL-35), which may serve as a new therapeutic target for T1DM (52), and developing comprehensive diabetes and kidney health management programs. Fourth, advocating for the end of diabetes stigma and discrimination (53). Negative judgments affect emotions, mental, and physical health, especially burdening children, adolescents, and CKD-T1DM patients. In summary, continuous improvement in data collection and deepened analysis hold promise for significantly reducing the global burden of CKD-T1DM and improving the health and quality of life for children and adolescents.

## Conclusion

This study utilized the Global Burden of Disease database to examine the incidence, prevalence, mortality, and DALYs of CKD-T1DM in children and adolescents from 1990 to 2021. The results indicate that, despite advances in medical technology reducing mortality and disability-adjusted life years (DALYs), the global prevalence and incidence of CKD-T1DM continue to rise. While new cases in low- and middle-SDI countries have significantly increased, high-SDI countries have effectively mitigated the disease burden. Generally, male patients experience higher incidence and DALYs than female patients. New cases of CKD-T1DM have significantly increased in Eastern Europe, Central Latin America, and Central Europe, while DALYs in East Asia have decreased due to effective interventions. At the national level, India and Indonesia have the highest new cases, but diagnoses and treatments are often delayed due to limited medical resources; in contrast, Northern and Western European countries have lower incidence rates due to advanced healthcare and public education. The incidence is higher in the 10-14 age group, while DALYs are higher in the 15-19 age group, indicating that children are more easily diagnosed, but adolescents have a higher risk of disease progression and mortality. Globally, the management of CKD-T1DM faces challenges but also provides successful experiences that can be learned from. Future research should focus on improving medical resources and health education tailored to the needs of different SDI countries, especially in low- and middle-SDI countries with high incidence rates. This will enhance the prevention and control of diabetes and its complications. Additionally, it should strengthen health protection, health education, and psychological support concerning environmental and occupational risk factors to ensure comprehensive health protection for children and adolescents.

## Data Availability

The original contributions presented in the study are included in the article/**Supplementary Material**. Further inquiries can be directed to the corresponding author.
