# Supplementary material for: Epidemiological trends and risk factors of CKD-T1DM in children and adolescents across 204 countries and territories (1990–2021)
Source: Front Endocrinol (Lausanne). 2025 Mar 26;16:1551467. doi: 10.3389/fendo.2025.1551467 (PMC11978622; doi:10.3389/fendo.2025.1551467)
Supplement: Supplementary file 1 [file DataSheet1.docx]

Appendix

**Appendix 1：Global time and sex burden of Chronic kidney disease due to diabetes mellitus type 1（CKD-T1DM）, 1990–2021**

| **measure** | **location** | **sex** | **age** | **cause** | **metric** | **year** | **val** | **upper** | **lower** |
| --- | --- | --- | --- | --- | --- | --- | --- | --- | --- |
| Incidence | Global | Male | 10-19 years | CKD-T1DM | Number | 1990 | 3605.114528 | 5686.874937 | 2038.788524 |
| Incidence | Global | Female | 10-19 years | CKD-T1DM | Number | 1990 | 2263.647727 | 3705.170854 | 1240.605567 |
| Incidence | Global | Both | 10-19 years | CKD-T1DM | Number | 1990 | 5868.762255 | 9450.576078 | 3341.994778 |
| Incidence | Global | Male | 10-19 years | CKD-T1DM | Number | 2021 | 8006.623204 | 11554.35054 | 5237.407631 |
| Incidence | Global | Female | 10-19 years | CKD-T1DM | Number | 2021 | 4001.417217 | 6049.004808 | 2371.799627 |
| Incidence | Global | Both | 10-19 years | CKD-T1DM | Number | 2021 | 12008.04042 | 17584.94533 | 7668.311869 |

Appendix

**Appendix 2：Global time and sex burden of Chronic kidney disease due to diabetes mellitus type 1（CKD-T1DM）, 1990–2021**

| **measure** | **Location** | **sex** | **age** | **cause** | **metric** | **year** | **val** | **upper** | **lower** |
| --- | --- | --- | --- | --- | --- | --- | --- | --- | --- |
| Prevalence | Global | Male | 10-19 years | CKD-T1DM | Number | 1990 | 231198.5843 | 289170.6321 | 182588.2926 |
| Prevalence | Global | Female | 10-19 years | CKD-T1DM | Number | 1990 | 285157.4812 | 371029.7511 | 219678.7637 |
| Prevalence | Global | Both | 10-19 years | CKD-T1DM | Number | 1990 | 516356.0654 | 660096.6016 | 402335.772 |
| Prevalence | Global | Male | 10-19 years | CKD-T1DM | Number | 2021 | 368709.4048 | 465501.2836 | 283981.0641 |
| Prevalence | Global | Female | 10-19 years | CKD-T1DM | Number | 2021 | 473491.1681 | 621466.3723 | 350563.6906 |
| Prevalence | Global | Both | 10-19 years | CKD-T1DM | Number | 2021 | 842200.5729 | 1083908.097 | 636833.0285 |

Appendix

**Appendix 3：Global time and sex burden of Chronic kidney disease due to diabetes mellitus type 1（CKD-T1DM）, 1990–2021**

| **measure** | **Location** | **sex** | **age** | **cause** | **metric** | **year** | **val** | **upper** | **lower** |
| --- | --- | --- | --- | --- | --- | --- | --- | --- | --- |
| Deaths | Global | Male | 10-19 years | CKD-T1DM | Number | 1990 | 518.8787731 | 794.1670812 | 288.2439302 |
| Deaths | Global | Female | 10-19 years | CKD-T1DM | Number | 1990 | 465.0267836 | 705.847208 | 277.5233318 |
| Deaths | Global | Both | 10-19 years | CKD-T1DM | Number | 1990 | 983.9055566 | 1472.986811 | 590.3227871 |
| Deaths | Global | Male | 10-19 years | CKD-T1DM | Number | 2021 | 383.530471 | 582.6153435 | 213.4486789 |
| Deaths | Global | Female | 10-19 years | CKD-T1DM | Number | 2021 | 283.2544143 | 432.4075229 | 162.1523731 |
| Deaths | Global | Both | 10-19 years | CKD-T1DM | Number | 2021 | 666.7848853 | 1016.710123 | 380.7132568 |

Appendix

**Appendix 4：Global time and sex burden of Chronic kidney disease due to diabetes mellitus type 1（CKD-T1DM）, 1990–2021**

| **measure** | **Location** | **sex** | **age** | **cause** | **metric** | **year** | **val** | **upper** | **lower** |
| --- | --- | --- | --- | --- | --- | --- | --- | --- | --- |
| DALYs | Global | Male | 10-19 years | CKD-T1DM | Number | 1990 | 39525.95354 | 59749.3281 | 22746.1988 |
| DALYs | Global | Female | 10-19 years | CKD-T1DM | Number | 1990 | 35296.55269 | 52996.45102 | 21400.75405 |
| DALYs | Global | Both | 10-19 years | CKD-T1DM | Number | 1990 | 74822.50623 | 110532.4225 | 45349.58598 |
| DALYs | Global | Male | 10-19 years | CKD-T1DM | Number | 2021 | 29752.47319 | 44296.96336 | 17018.88338 |
| DALYs | Global | Female | 10-19 years | CKD-T1DM | Number | 2021 | 22169.99145 | 33130.81267 | 13075.83435 |
| DALYs | Global | Both | 10-19 years | CKD-T1DM | Number | 2021 | 51922.46464 | 77279.06936 | 30201.70853 |

Appendix

**Appendix 5：Global time and sex burden of Chronic kidney disease due to diabetes mellitus type 1（CKD-T1DM）, 1990–2021**

| **measure** | **Location** | **sex** | **age** | **cause** | **metric** | **year** | **val** | **upper** | **lower** |
| --- | --- | --- | --- | --- | --- | --- | --- | --- | --- |
| Incidence | Global | Male | Age-standardized | CKD-T1DM | Rate | 1990 | 0.115995144 | 0.182976122 | 0.065598351 |
| Incidence | Global | Female | Age-standardized | CKD-T1DM | Rate | 1990 | 0.075815431 | 0.124095778 | 0.041551097 |
| Incidence | Global | Both | Age-standardized | CKD-T1DM | Rate | 1990 | 0.096308332 | 0.155087082 | 0.054843241 |
| Incidence | Global | Male | Age-standardized | CKD-T1DM | Rate | 2021 | 0.208770317 | 0.301276252 | 0.136563845 |
| Incidence | Global | Female | Age-standardized | CKD-T1DM | Rate | 2021 | 0.110574129 | 0.167156636 | 0.065541698 |
| Incidence | Global | Both | Age-standardized | CKD-T1DM | Rate | 2021 | 0.161097434 | 0.235916059 | 0.102876516 |

Appendix

**Appendix 6：Global time and sex burden of Chronic kidney disease due to diabetes mellitus type 1（CKD-T1DM）, 1990–2021**

| **measure** | **Location** | **sex** | **age** | **cause** | **metric** | **year** | **val** | **upper** | **lower** |
| --- | --- | --- | --- | --- | --- | --- | --- | --- | --- |
| Prevalence | Global | Male | Age-standardized | CKD-T1DM | Rate | 1990 | 7.438851905 | 9.304111936 | 5.874807896 |
| Prevalence | Global | Female | Age-standardized | CKD-T1DM | Rate | 1990 | 9.550663369 | 12.42674833 | 7.357611356 |
| Prevalence | Global | Both | Age-standardized | CKD-T1DM | Rate | 1990 | 8.473573989 | 10.83240378 | 6.602463224 |
| Prevalence | Global | Male | Age-standardized | CKD-T1DM | Rate | 2021 | 9.61398801 | 12.13780745 | 7.404721739 |
| Prevalence | Global | Female | Age-standardized | CKD-T1DM | Rate | 2021 | 13.08433259 | 17.17344114 | 9.687386438 |
| Prevalence | Global | Both | Age-standardized | CKD-T1DM | Rate | 2021 | 11.29879203 | 14.54149114 | 8.543622713 |

Appendix

**Appendix 7：Global time and sex burden of Chronic kidney disease due to diabetes mellitus type 1（CKD-T1DM）, 1990–2021**

| **measure** | **Location** | **sex** | **age** | **cause** | **metric** | **year** | **val** | **upper** | **lower** |
| --- | --- | --- | --- | --- | --- | --- | --- | --- | --- |
| DALYs | Global | Male | Age-standardized | CKD-T1DM | Rate | 1990 | 1.271753959 | 1.922444311 | 0.731862631 |
| DALYs | Global | Female | Age-standardized | CKD-T1DM | Rate | 1990 | 1.182173063 | 1.774988548 | 0.716766739 |
| DALYs | Global | Both | Age-standardized | CKD-T1DM | Rate | 1990 | 1.227862099 | 1.813873648 | 0.74420172 |
| DALYs | Global | Male | Age-standardized | CKD-T1DM | Rate | 2021 | 0.775786885 | 1.155030138 | 0.44376232 |
| DALYs | Global | Female | Age-standardized | CKD-T1DM | Rate | 2021 | 0.612639815 | 0.915528316 | 0.361334228 |
| DALYs | Global | Both | Age-standardized | CKD-T1DM | Rate | 2021 | 0.696581252 | 1.036760317 | 0.405179994 |

Appendix

**Appendix 8：Global time and sex burden of Chronic kidney disease due to diabetes mellitus type 1（CKD-T1DM）, 1990–2021**

| **measure** | **Location** | **sex** | **age** | **cause** | **metric** | **year** | **val** | **upper** | **lower** |
| --- | --- | --- | --- | --- | --- | --- | --- | --- | --- |
| Deaths | Global | Male | Age-standardized | CKD-T1DM | Rate | 1990 | 0.016695009 | 0.025552454 | 0.009274295 |
| Deaths | Global | Female | Age-standardized | CKD-T1DM | Rate | 1990 | 0.015574953 | 0.023640653 | 0.009294976 |
| Deaths | Global | Both | Age-standardized | CKD-T1DM | Rate | 1990 | 0.016146216 | 0.024172201 | 0.009687392 |
| Deaths | Global | Male | Age-standardized | CKD-T1DM | Rate | 2021 | 0.010000443 | 0.015191522 | 0.005565611 |
| Deaths | Global | Female | Age-standardized | CKD-T1DM | Rate | 2021 | 0.007827379 | 0.011949038 | 0.004480877 |
| Deaths | Global | Both | Age-standardized | CKD-T1DM | Rate | 2021 | 0.008945451 | 0.013639977 | 0.005107572 |

Appendix

**Appendix 9：****Region and sex trends of Chronic kidney disease due to diabetes mellitus type 1 in children and adolescents from 1990 to 2021**

| **location** | **Num_1990** | **ASR_1990** | **Num_2019** | **ASR_2019** | **EAPC_CI** |
| --- | --- | --- | --- | --- | --- |
| Middle SDI | 2097.1 (1136.2-3529.1) | 0.1 (0.1-0.2) | 3930.2 (2412.4-5847.7) | 0.2 (0.1-0.3) | 2.24 (2.13-2.34) |
| High-middle SDI | 801.9 (402.3-1474.3) | 0.1 (0-0.1) | 1141 (618.4-1919.5) | 0.1 (0.1-0.2) | 1.88 (1.7-2.05) |
| Low-middle SDI | 1641.4 (857.4-2780.2) | 0.1 (0.1-0.2) | 3764.5 (2118.7-6057.5) | 0.2 (0.1-0.3) | 1.49 (1.42-1.56) |
| Low SDI | 786.4 (435.4-1391.1) | 0.1 (0.1-0.2) | 2575.6 (1538.8-4148.6) | 0.2 (0.1-0.3) | 1.12 (1-1.25) |
| High SDI | 536.3 (230.3-1073.8) | 0.1 (0-0.1) | 586.4 (252.4-1123.9) | 0.1 (0-0.2) | 0.39 (0.31-0.48) |

Appendix

**Appendix 10：Region and sex trends of Chronic kidney disease due to diabetes mellitus type 1 in children and adolescents from 1990 to 2021**

| **location** | **Num_1990** | **ASR_1990** | **Num_2019** | **ASR_2019** | **EAPC_CI** |
| --- | --- | --- | --- | --- | --- |
| Low-middle SDI | 11417.2 (6388.3-17730.7) | 0.8 (0.4-1.2) | 12616.2 (7323.4-19157.7) | 0.6 (0.3-0.9) | -1.02 (-1.08--0.96) |
| Low SDI | 5787.9 (3097.4-9219.9) | 0.9 (0.5-1.4) | 9451 (5284.4-15473.9) | 0.6 (0.3-1) | -1.54 (-1.66--1.42) |
| Middle SDI | 43418.5 (25609.3-63941.4) | 2 (1.2-3) | 24538.7 (14730-36188.9) | 1.1 (0.7-1.7) | -1.81 (-1.88--1.74) |
| High-middle SDI | 12598.9 (7500.6-18872.9) | 1.2 (0.7-1.8) | 4560.8 (2783.3-6797.2) | 0.5 (0.3-0.8) | -2.7 (-2.93--2.47) |
| High SDI | 1535.3 (974.2-2227) | 0.2 (0.1-0.3) | 676.4 (436.7-969) | 0.1 (0.1-0.1) | -2.77 (-3.01--2.53) |

Appendix

**Appendix 11：Region and sex trends of Chronic kidney disease due to diabetes mellitus type 1 in children and adolescents from 1990 to 2021**

| **Location** | **Num_1990** | **ASR_1990** | **Num_2019** | **ASR_2019** | **EAPC_CI** |
| --- | --- | --- | --- | --- | --- |
| Eastern Europe | 254 (121.5-470.3) | 0.1 (0.1-0.3) | 412.6 (212.7-728.4) | 0.3 (0.2-0.5) | 2.93 (2.76-3.1) |
| Central Latin America | 306.7 (131.6-576.1) | 0.1 (0.1-0.3) | 732 (406.2-1203.4) | 0.3 (0.2-0.5) | 2.63 (2.48-2.78) |
| Central Europe | 100.2 (40-217.5) | 0.1 (0-0.2) | 122.7 (55.1-241.8) | 0.2 (0.1-0.3) | 2.6 (2.54-2.66) |
| Southeast Asia | 906.7 (407.3-1725.3) | 0.2 (0.1-0.3) | 1819.6 (1023.2-2916.8) | 0.3 (0.2-0.4) | 2.42 (2.3-2.54) |
| Central Asia | 298 (109.3-726.6) | 0.4 (0.1-0.9) | 609.4 (218.4-1435.1) | 0.7 (0.2-1.6) | 2.21 (1.96-2.45) |
| Australasia | 8.7 (1.2-31.8) | 0 (0-0.2) | 19 (2.4-68.4) | 0.1 (0-0.3) | 2.09 (2.03-2.15) |
| Andean Latin America | 46.9 (10.2-148.7) | 0.1 (0-0.3) | 108.1 (24.8-300.9) | 0.2 (0-0.5) | 2.06 (2-2.13) |
| Caribbean | 44.2 (14-108.1) | 0.1 (0-0.3) | 82.2 (28.6-197.4) | 0.2 (0.1-0.5) | 2.03 (1.94-2.11) |
| North Africa and Middle East | 450.4 (176.3-869.9) | 0.1 (0-0.2) | 1105.5 (456.9-2201.4) | 0.2 (0.1-0.3) | 1.81 (1.68-1.94) |
| Tropical Latin America | 139.4 (48.3-297.6) | 0.1 (0-0.2) | 193.3 (71.5-377.2) | 0.1 (0-0.2) | 1.3 (1.21-1.4) |
| Southern Latin America | 27.6 (4.5-85.1) | 0.1 (0-0.2) | 40.3 (7.7-117.8) | 0.1 (0-0.2) | 1.3 (1.17-1.42) |
| High-income North America | 233.4 (100.5-495) | 0.1 (0-0.2) | 199.9 (76.5-406.6) | 0.1 (0-0.1) | -1.28 (-1.41--1.15) |
| South Asia | 1362.3 (621.7-2379.8) | 0.1 (0-0.2) | 3032 (1589.3-5193.6) | 0.1 (0.1-0.3) | 1.28 (1.24-1.33) |
| Western Europe | 122.3 (37.6-278.3) | 0 (0-0.1) | 165.4 (51.5-380.1) | 0.1 (0-0.1) | 1.28 (1.19-1.37) |
| Eastern Sub-Saharan Africa | 333.5 (166.1-644.4) | 0.1 (0.1-0.2) | 1104.2 (617.5-1922.6) | 0.2 (0.1-0.3) | 1.27 (1.1-1.44) |
| Central Sub-Saharan Africa | 75.1 (17.4-230.4) | 0.1 (0-0.3) | 273.5 (65.4-818.8) | 0.1 (0-0.4) | 1.1 (0.93-1.27) |
| Oceania | 9.1 (2.7-24.4) | 0.1 (0-0.3) | 23.4 (6.4-72.1) | 0.1 (0-0.4) | 0.95 (0.83-1.06) |
| Western Sub-Saharan Africa | 373 (198.7-648) | 0.1 (0.1-0.3) | 1295.5 (743.5-2106.8) | 0.2 (0.1-0.3) | 0.76 (0.61-0.91) |
| Southern Sub-Saharan Africa | 54.8 (20.9-135.8) | 0.1 (0-0.2) | 82.4 (32.7-190.5) | 0.1 (0-0.2) | 0.55 (0.2-0.89) |
| East Asia | 617 (233.8-1304.3) | 0 (0-0.1) | 529.4 (214.3-1072.6) | 0.1 (0-0.1) | 0.44 (0.18-0.69) |
| High-income Asia Pacific | 105.3 (34.7-250.2) | 0.1 (0-0.2) | 57.8 (20.4-127.3) | 0.1 (0-0.1) | -0.3 (-0.44--0.16) |

Appendix

**Appendix 12：Region and sex trends of Chronic kidney disease due to diabetes mellitus type 1 in children and adolescents from 1990 to 2021**

| **Location** | **Num_1990** | **ASR_1990** | **Num_2019** | **ASR_2019** | **EAPC_CI** |
| --- | --- | --- | --- | --- | --- |
| East Asia | 38503.2 (22601.4-58744.3) | 2.8 (1.7-4.3) | 9963.5 (5830.1-14963.2) | 1 (0.6-1.6) | -3.49 (-3.66--3.32) |
| High-income Asia Pacific | 219.2 (136.8-324.6) | 0.1 (0.1-0.2) | 58.2 (37.3-86.7) | 0.1 (0-0.1) | -2.37 (-2.56--2.19) |
| Eastern Sub-Saharan Africa | 4277.2 (2188.6-6798.4) | 1.6 (0.8-2.6) | 6277.7 (3353-10437.3) | 1 (0.6-1.7) | -1.93 (-2.07--1.78) |
| Eastern Europe | 289.3 (172.2-443) | 0.2 (0.1-0.2) | 131.2 (82.6-203.2) | 0.1 (0.1-0.2) | -1.78 (-1.92--1.64) |
| Australasia | 3.1 (1.3-9.3) | 0 (0-0.1) | 5.1 (1.8-15.8) | 0 (0-0.1) | 1.4 (1.27-1.54) |
| Southern Sub-Saharan Africa | 101.1 (56.4-163.4) | 0.1 (0.1-0.2) | 206.8 (111.3-355.6) | 0.2 (0.1-0.4) | 1.35 (0.81-1.89) |
| Caribbean | 399.5 (222.4-643.3) | 1 (0.5-1.5) | 527.6 (281-931.9) | 1.2 (0.6-2.1) | 1.2 (1-1.4) |
| Central Asia | 142.1 (82.1-228.2) | 0.2 (0.1-0.3) | 221.1 (125.8-373) | 0.3 (0.1-0.4) | 1.09 (0.71-1.46) |
| Tropical Latin America | 731.1 (411.6-1159.1) | 0.4 (0.2-0.6) | 477.7 (271.5-749.4) | 0.3 (0.1-0.4) | -0.86 (-1.05--0.66) |
| Oceania | 188.6 (86.6-322.9) | 2.2 (1-3.8) | 484 (261.2-828.6) | 3 (1.6-5.2) | 0.82 (0.63-1.02) |
| High-income North America | 114 (70.2-172.8) | 0 (0-0.1) | 108.2 (66-166.3) | 0 (0-0.1) | -0.64 (-0.99--0.29) |
| Western Europe | 104.3 (64-168.7) | 0 (0-0.1) | 112.5 (62.9-196.9) | 0 (0-0.1) | 0.63 (0.51-0.76) |
| Andean Latin America | 317.4 (174.7-523.6) | 0.6 (0.3-1) | 339.4 (169.3-595.2) | 0.5 (0.3-0.9) | -0.53 (-0.89--0.17) |
| Central Sub-Saharan Africa | 505.3 (247-882.9) | 0.7 (0.3-1.2) | 1146.7 (575.2-2154.4) | 0.6 (0.3-1.1) | -0.45 (-0.52--0.39) |
| Southern Latin America | 73.5 (40.3-119.2) | 0.1 (0.1-0.2) | 60.9 (35-104.1) | 0.1 (0.1-0.2) | -0.33 (-0.56--0.1) |
| Central Latin America | 787.6 (443.2-1282.5) | 0.4 (0.2-0.6) | 828.1 (457.6-1341.8) | 0.3 (0.2-0.5) | 0.29 (-0.11-0.69) |
| South Asia | 2880.5 (1735.4-4411.2) | 0.2 (0.1-0.3) | 4034.5 (2444.2-6200.7) | 0.2 (0.1-0.3) | -0.27 (-0.46--0.07) |
| Western Sub-Saharan Africa | 1134 (620.8-1866.7) | 0.5 (0.2-0.7) | 2844 (1546.5-4731.1) | 0.4 (0.2-0.7) | -0.27 (-0.4--0.14) |
| Southeast Asia | 23044.9 (12946.2-35410.8) | 3.8 (2.2-5.9) | 22935.3 (13254.6-35477.5) | 3.5 (2-5.4) | -0.26 (-0.31--0.2) |
| North Africa and Middle East | 872.1 (472.9-1453.5) | 0.2 (0.1-0.3) | 1087.2 (567.6-1832) | 0.2 (0.1-0.3) | -0.24 (-0.29--0.19) |
| Central Europe | 134.6 (84-204.6) | 0.1 (0.1-0.2) | 72.8 (39.8-123.8) | 0.1 (0.1-0.2) | -0.14 (-0.23--0.04) |

Appendix

**Appendix 13：Global disease burden distribution of** **Chronic kidney disease due to diabetes mellitus type 1 in children and adolescents from 1990 to 2021.**

| **measure** | **location** | **sex** | **age** | **cause** | **metric** | **year** | **val** | **upper** | **lower** |
| --- | --- | --- | --- | --- | --- | --- | --- | --- | --- |
| Incidence | India | Both | 10-19 years | CKD-T1DM | Number | 2021 | 2129.287 | 3745.094 | 1127.79 |
| Incidence | Indonesia | Both | 10-19 years | CKD-T1DM | Number | 2021 | 776.1732 | 1391.173 | 397.3064 |
| Incidence | Pakistan | Both | 10-19 years | CKD-T1DM | Number | 2021 | 708.8575 | 1866.458 | 210.0011 |
| Incidence | Nigeria | Both | 10-19 years | CKD-T1DM | Number | 2021 | 510.5765 | 838.566 | 266.6069 |
| Incidence | China | Both | 10-19 years | CKD-T1DM | Number | 2021 | 489.6276 | 1000.753 | 187.1319 |
| Incidence | Mexico | Both | 10-19 years | CKD-T1DM | Number | 2021 | 466.4199 | 805.8598 | 257.3366 |
| Incidence | Philippines | Both | 10-19 years | CKD-T1DM | Number | 2021 | 436.8711 | 655.5401 | 267.7808 |
| Incidence | Uzbekistan | Both | 10-19 years | CKD-T1DM | Number | 2021 | 311.65 | 1099.586 | 35.75322 |
| Incidence | Russian Federation | Both | 10-19 years | CKD-T1DM | Number | 2021 | 307.4606 | 534.6797 | 150.4323 |
| Incidence | Egypt | Both | 10-19 years | CKD-T1DM | Number | 2021 | 226.3366 | 835.9914 | 19.1346 |
| Incidence | Ethiopia | Both | 10-19 years | CKD-T1DM | Number | 2021 | 214.5783 | 495.1076 | 76.99181 |
| Incidence | Myanmar | Both | 10-19 years | CKD-T1DM | Number | 2021 | 192.7177 | 760.3246 | 19.12418 |
| Incidence | Brazil | Both | 10-19 years | CKD-T1DM | Number | 2021 | 183.2133 | 365.2774 | 66.69437 |
| Incidence | Viet Nam | Both | 10-19 years | CKD-T1DM | Number | 2021 | 180.5066 | 736.8481 | 16.43807 |
| Incidence | United States of America | Both | 10-19 years | CKD-T1DM | Number | 2021 | 177.2832 | 367.8852 | 65.30332 |
| Incidence | Democratic Republic of the Congo | Both | 10-19 years | CKD-T1DM | Number | 2021 | 172.883 | 679.8487 | 16.1991 |
| Incidence | Kenya | Both | 10-19 years | CKD-T1DM | Number | 2021 | 150.3104 | 239.0991 | 89.03746 |
| Incidence | Cameroon | Both | 10-19 years | CKD-T1DM | Number | 2021 | 143.6638 | 573.2386 | 11.65453 |
| Incidence | Bangladesh | Both | 10-19 years | CKD-T1DM | Number | 2021 | 126.2296 | 486.6906 | 9.988107 |
| Incidence | United Republic of Tanzania | Both | 10-19 years | CKD-T1DM | Number | 2021 | 120.8017 | 434.0668 | 12.75595 |
| Incidence | Mozambique | Both | 10-19 years | CKD-T1DM | Number | 2021 | 115.7301 | 441.5208 | 13.27404 |
| Incidence | Uganda | Both | 10-19 years | CKD-T1DM | Number | 2021 | 103.4123 | 386.098 | 11.36135 |
| Incidence | T眉rkiye | Both | 10-19 years | CKD-T1DM | Number | 2021 | 100.6027 | 392.1249 | 8.43111 |
| Incidence | Saudi Arabia | Both | 10-19 years | CKD-T1DM | Number | 2021 | 98.864 | 379.7859 | 8.739865 |
| Incidence | Iran (Islamic Republic of) | Both | 10-19 years | CKD-T1DM | Number | 2021 | 94.82214 | 195.6423 | 40.58408 |
| Incidence | Iraq | Both | 10-19 years | CKD-T1DM | Number | 2021 | 94.78156 | 374.5238 | 6.583984 |
| Incidence | C么te d'Ivoire | Both | 10-19 years | CKD-T1DM | Number | 2021 | 92.28845 | 353.6281 | 8.089142 |
| Incidence | Afghanistan | Both | 10-19 years | CKD-T1DM | Number | 2021 | 91.29428 | 408.9471 | 6.596298 |
| Incidence | Thailand | Both | 10-19 years | CKD-T1DM | Number | 2021 | 90.12093 | 404.7178 | 8.319643 |
| Incidence | Ghana | Both | 10-19 years | CKD-T1DM | Number | 2021 | 89.65308 | 360.4031 | 8.560602 |
| Incidence | Sudan | Both | 10-19 years | CKD-T1DM | Number | 2021 | 84.95254 | 378.6238 | 7.414866 |
| Incidence | Malawi | Both | 10-19 years | CKD-T1DM | Number | 2021 | 78.98895 | 286.9626 | 9.86968 |
| Incidence | Madagascar | Both | 10-19 years | CKD-T1DM | Number | 2021 | 73.50298 | 261.5814 | 7.995617 |
| Incidence | Ukraine | Both | 10-19 years | CKD-T1DM | Number | 2021 | 72.43822 | 250.7616 | 9.18942 |
| Incidence | Niger | Both | 10-19 years | CKD-T1DM | Number | 2021 | 69.58834 | 246.0039 | 6.170471 |
| Incidence | Kazakhstan | Both | 10-19 years | CKD-T1DM | Number | 2021 | 68.84605 | 270.091 | 6.424784 |
| Incidence | Somalia | Both | 10-19 years | CKD-T1DM | Number | 2021 | 68.46304 | 276.6408 | 7.200145 |
| Incidence | Guatemala | Both | 10-19 years | CKD-T1DM | Number | 2021 | 68.19227 | 285.3193 | 6.187824 |
| Incidence | Angola | Both | 10-19 years | CKD-T1DM | Number | 2021 | 68.05133 | 292.8242 | 5.903115 |
| Incidence | Nepal | Both | 10-19 years | CKD-T1DM | Number | 2021 | 66.11446 | 277.0444 | 5.340087 |
| Incidence | Azerbaijan | Both | 10-19 years | CKD-T1DM | Number | 2021 | 63.45231 | 246.3126 | 6.665994 |
| Incidence | Zambia | Both | 10-19 years | CKD-T1DM | Number | 2021 | 61.84812 | 271.229 | 7.136687 |
| Incidence | Morocco | Both | 10-19 years | CKD-T1DM | Number | 2021 | 58.90937 | 266.1464 | 4.737694 |
| Incidence | Burkina Faso | Both | 10-19 years | CKD-T1DM | Number | 2021 | 55.92742 | 229.3268 | 5.370294 |
| Incidence | Yemen | Both | 10-19 years | CKD-T1DM | Number | 2021 | 54.99579 | 242.192 | 5.559711 |
| Incidence | Algeria | Both | 10-19 years | CKD-T1DM | Number | 2021 | 54.67409 | 194.4608 | 4.582641 |
| Incidence | South Africa | Both | 10-19 years | CKD-T1DM | Number | 2021 | 52.63334 | 128.6311 | 17.65016 |
| Incidence | Chad | Both | 10-19 years | CKD-T1DM | Number | 2021 | 51.67546 | 203.4495 | 4.67608 |
| Incidence | Mali | Both | 10-19 years | CKD-T1DM | Number | 2021 | 51.39834 | 207.3076 | 3.772029 |
| Incidence | Venezuela (Bolivarian Republic of) | Both | 10-19 years | CKD-T1DM | Number | 2021 | 50.6501 | 204.0873 | 4.26085 |
| Incidence | Syrian Arab Republic | Both | 10-19 years | CKD-T1DM | Number | 2021 | 50.11815 | 209.0648 | 4.574284 |
| Incidence | Peru | Both | 10-19 years | CKD-T1DM | Number | 2021 | 48.45771 | 195.1419 | 4.613433 |
| Incidence | Colombia | Both | 10-19 years | CKD-T1DM | Number | 2021 | 48.11228 | 194.9641 | 3.997484 |
| Incidence | Senegal | Both | 10-19 years | CKD-T1DM | Number | 2021 | 45.45788 | 182.4237 | 4.419172 |
| Incidence | Guinea | Both | 10-19 years | CKD-T1DM | Number | 2021 | 45.41323 | 177.3372 | 4.197579 |
| Incidence | Turkmenistan | Both | 10-19 years | CKD-T1DM | Number | 2021 | 45.32851 | 162.5693 | 4.810024 |
| Incidence | Tajikistan | Both | 10-19 years | CKD-T1DM | Number | 2021 | 44.19567 | 175.7483 | 4.141232 |
| Incidence | Malaysia | Both | 10-19 years | CKD-T1DM | Number | 2021 | 43.42647 | 174.8975 | 3.278585 |
| Incidence | Japan | Both | 10-19 years | CKD-T1DM | Number | 2021 | 41.61265 | 87.31362 | 14.84145 |
| Incidence | Benin | Both | 10-19 years | CKD-T1DM | Number | 2021 | 40.97508 | 169.9234 | 3.41992 |
| Incidence | Kyrgyzstan | Both | 10-19 years | CKD-T1DM | Number | 2021 | 40.77637 | 143.0464 | 4.18724 |
| Incidence | Ecuador | Both | 10-19 years | CKD-T1DM | Number | 2021 | 37.87684 | 162.8668 | 3.329993 |
| Incidence | Germany | Both | 10-19 years | CKD-T1DM | Number | 2021 | 34.79366 | 128.0002 | 2.94121 |
| Incidence | Sri Lanka | Both | 10-19 years | CKD-T1DM | Number | 2021 | 34.42356 | 139.8519 | 3.442281 |
| Incidence | Cambodia | Both | 10-19 years | CKD-T1DM | Number | 2021 | 34.08326 | 141.0671 | 3.032433 |
| Incidence | France | Both | 10-19 years | CKD-T1DM | Number | 2021 | 32.84284 | 119.5801 | 2.918752 |
| Incidence | Rwanda | Both | 10-19 years | CKD-T1DM | Number | 2021 | 32.23557 | 113.1267 | 3.989577 |
| Incidence | Burundi | Both | 10-19 years | CKD-T1DM | Number | 2021 | 30.29438 | 122.6288 | 2.835031 |
| Incidence | Nicaragua | Both | 10-19 years | CKD-T1DM | Number | 2021 | 30.06205 | 117.4606 | 2.42613 |
| Incidence | El Salvador | Both | 10-19 years | CKD-T1DM | Number | 2021 | 29.50049 | 109.5869 | 2.46889 |
| Incidence | Haiti | Both | 10-19 years | CKD-T1DM | Number | 2021 | 29.07846 | 121.9562 | 2.866045 |
| Incidence | Togo | Both | 10-19 years | CKD-T1DM | Number | 2021 | 28.32876 | 103.7797 | 2.969684 |
| Incidence | South Sudan | Both | 10-19 years | CKD-T1DM | Number | 2021 | 28.05085 | 109.765 | 3.195425 |
| Incidence | Democratic People's Republic of Korea | Both | 10-19 years | CKD-T1DM | Number | 2021 | 27.90546 | 124.9994 | 2.631947 |
| Incidence | Argentina | Both | 10-19 years | CKD-T1DM | Number | 2021 | 27.07041 | 97.2084 | 2.492882 |
| Incidence | Romania | Both | 10-19 years | CKD-T1DM | Number | 2021 | 26.55086 | 89.4192 | 2.728851 |
| Incidence | Jordan | Both | 10-19 years | CKD-T1DM | Number | 2021 | 24.56688 | 113.2641 | 2.040491 |
| Incidence | Canada | Both | 10-19 years | CKD-T1DM | Number | 2021 | 22.57896 | 85.36744 | 1.744765 |
| Incidence | Honduras | Both | 10-19 years | CKD-T1DM | Number | 2021 | 22.30148 | 98.14092 | 2.015433 |
| Incidence | Poland | Both | 10-19 years | CKD-T1DM | Number | 2021 | 22.29781 | 45.58028 | 6.889415 |
| Incidence | Sierra Leone | Both | 10-19 years | CKD-T1DM | Number | 2021 | 21.92872 | 79.23708 | 1.95538 |
| Incidence | Bolivia (Plurinational State of) | Both | 10-19 years | CKD-T1DM | Number | 2021 | 21.75345 | 92.08725 | 1.835963 |
| Incidence | United Kingdom | Both | 10-19 years | CKD-T1DM | Number | 2021 | 21.49951 | 45.04826 | 7.114362 |
| Incidence | Dominican Republic | Both | 10-19 years | CKD-T1DM | Number | 2021 | 20.6953 | 86.63776 | 1.701389 |
| Incidence | Lao People's Democratic Republic | Both | 10-19 years | CKD-T1DM | Number | 2021 | 20.16367 | 81.95738 | 1.73545 |
| Incidence | Eritrea | Both | 10-19 years | CKD-T1DM | Number | 2021 | 19.72839 | 67.88036 | 2.294333 |
| Incidence | Italy | Both | 10-19 years | CKD-T1DM | Number | 2021 | 18.83617 | 45.47372 | 4.998431 |
| Incidence | Zimbabwe | Both | 10-19 years | CKD-T1DM | Number | 2021 | 18.63105 | 80.12102 | 1.899055 |
| Incidence | Belarus | Both | 10-19 years | CKD-T1DM | Number | 2021 | 16.95233 | 65.92545 | 1.969968 |
| Incidence | Australia | Both | 10-19 years | CKD-T1DM | Number | 2021 | 16.44375 | 64.96599 | 1.304604 |
| Incidence | Mongolia | Both | 10-19 years | CKD-T1DM | Number | 2021 | 15.75319 | 66.02278 | 1.422345 |
| Incidence | Liberia | Both | 10-19 years | CKD-T1DM | Number | 2021 | 15.3568 | 57.987 | 1.668631 |
| Incidence | Papua New Guinea | Both | 10-19 years | CKD-T1DM | Number | 2021 | 15.03908 | 61.64909 | 1.295375 |
| Incidence | Bulgaria | Both | 10-19 years | CKD-T1DM | Number | 2021 | 14.8384 | 54.40546 | 1.759991 |
| Incidence | Mauritania | Both | 10-19 years | CKD-T1DM | Number | 2021 | 14.82998 | 55.91615 | 1.315138 |
| Incidence | Republic of Korea | Both | 10-19 years | CKD-T1DM | Number | 2021 | 13.79284 | 56.71137 | 1.030323 |
| Incidence | Palestine | Both | 10-19 years | CKD-T1DM | Number | 2021 | 13.57905 | 53.40259 | 1.420933 |
| Incidence | United Arab Emirates | Both | 10-19 years | CKD-T1DM | Number | 2021 | 13.07773 | 54.56301 | 1.076348 |
| Incidence | Congo | Both | 10-19 years | CKD-T1DM | Number | 2021 | 12.7572 | 51.59106 | 1.150584 |
| Incidence | Libya | Both | 10-19 years | CKD-T1DM | Number | 2021 | 12.40065 | 48.88687 | 1.242 |
| Incidence | Taiwan (Province of China) | Both | 10-19 years | CKD-T1DM | Number | 2021 | 11.83978 | 43.40127 | 1.220896 |
| Incidence | Chile | Both | 10-19 years | CKD-T1DM | Number | 2021 | 11.39338 | 45.73162 | 0.922491 |
| Incidence | Central African Republic | Both | 10-19 years | CKD-T1DM | Number | 2021 | 11.14138 | 42.20509 | 1.092167 |
| Incidence | Tunisia | Both | 10-19 years | CKD-T1DM | Number | 2021 | 10.90693 | 44.82154 | 0.891973 |
| Incidence | Spain | Both | 10-19 years | CKD-T1DM | Number | 2021 | 10.86239 | 37.7973 | 0.846003 |
| Incidence | Georgia | Both | 10-19 years | CKD-T1DM | Number | 2021 | 10.61067 | 37.10513 | 1.293055 |
| Incidence | Cuba | Both | 10-19 years | CKD-T1DM | Number | 2021 | 10.35816 | 48.42884 | 0.767085 |
| Incidence | Serbia | Both | 10-19 years | CKD-T1DM | Number | 2021 | 10.33908 | 34.98588 | 0.955049 |
| Incidence | Czechia | Both | 10-19 years | CKD-T1DM | Number | 2021 | 10.16934 | 36.41273 | 0.921298 |
| Incidence | Paraguay | Both | 10-19 years | CKD-T1DM | Number | 2021 | 10.04473 | 39.42251 | 0.820592 |
| Incidence | Hungary | Both | 10-19 years | CKD-T1DM | Number | 2021 | 8.919335 | 30.28358 | 1.111905 |
| Incidence | Panama | Both | 10-19 years | CKD-T1DM | Number | 2021 | 8.825573 | 34.97043 | 0.858064 |
| Incidence | Armenia | Both | 10-19 years | CKD-T1DM | Number | 2021 | 8.821682 | 33.49714 | 0.847472 |
| Incidence | Guinea-Bissau | Both | 10-19 years | CKD-T1DM | Number | 2021 | 8.264223 | 37.27774 | 0.668028 |
| Incidence | Gambia | Both | 10-19 years | CKD-T1DM | Number | 2021 | 8.019159 | 35.31097 | 0.749221 |
| Incidence | Costa Rica | Both | 10-19 years | CKD-T1DM | Number | 2021 | 7.89327 | 32.31393 | 0.653279 |
| Incidence | Israel | Both | 10-19 years | CKD-T1DM | Number | 2021 | 7.269644 | 28.91288 | 0.6414 |
| Incidence | Albania | Both | 10-19 years | CKD-T1DM | Number | 2021 | 7.036001 | 24.22058 | 0.866217 |
| Incidence | Lebanon | Both | 10-19 years | CKD-T1DM | Number | 2021 | 6.270042 | 26.6493 | 0.545845 |
| Incidence | Ireland | Both | 10-19 years | CKD-T1DM | Number | 2021 | 5.862411 | 21.3494 | 0.692354 |
| Incidence | Oman | Both | 10-19 years | CKD-T1DM | Number | 2021 | 5.798357 | 23.0244 | 0.533837 |
| Incidence | Republic of Moldova | Both | 10-19 years | CKD-T1DM | Number | 2021 | 5.728228 | 18.9313 | 0.685147 |
| Incidence | Jamaica | Both | 10-19 years | CKD-T1DM | Number | 2021 | 5.628825 | 20.75155 | 0.512438 |
| Incidence | Netherlands | Both | 10-19 years | CKD-T1DM | Number | 2021 | 5.369682 | 20.56898 | 0.412749 |
| Incidence | Gabon | Both | 10-19 years | CKD-T1DM | Number | 2021 | 5.170498 | 20.49315 | 0.44981 |
| Incidence | Bosnia and Herzegovina | Both | 10-19 years | CKD-T1DM | Number | 2021 | 4.976542 | 15.81068 | 0.577747 |
| Incidence | Slovakia | Both | 10-19 years | CKD-T1DM | Number | 2021 | 4.765645 | 17.12672 | 0.449252 |
| Incidence | Mauritius | Both | 10-19 years | CKD-T1DM | Number | 2021 | 4.310526 | 16.38799 | 0.386396 |
| Incidence | Kuwait | Both | 10-19 years | CKD-T1DM | Number | 2021 | 4.227632 | 17.63612 | 0.354982 |
| Incidence | Belgium | Both | 10-19 years | CKD-T1DM | Number | 2021 | 4.219439 | 17.6051 | 0.375203 |
| Incidence | Croatia | Both | 10-19 years | CKD-T1DM | Number | 2021 | 4.198371 | 14.94384 | 0.425672 |
| Incidence | Austria | Both | 10-19 years | CKD-T1DM | Number | 2021 | 4.158892 | 16.19675 | 0.345913 |
| Incidence | Lithuania | Both | 10-19 years | CKD-T1DM | Number | 2021 | 4.149672 | 14.3331 | 0.464523 |
| Incidence | Greece | Both | 10-19 years | CKD-T1DM | Number | 2021 | 3.67152 | 15.31288 | 0.250133 |
| Incidence | Timor-Leste | Both | 10-19 years | CKD-T1DM | Number | 2021 | 3.624711 | 13.58153 | 0.341792 |
| Incidence | Equatorial Guinea | Both | 10-19 years | CKD-T1DM | Number | 2021 | 3.526921 | 12.71565 | 0.317888 |
| Incidence | Djibouti | Both | 10-19 years | CKD-T1DM | Number | 2021 | 3.402583 | 11.45598 | 0.411337 |
| Incidence | North Macedonia | Both | 10-19 years | CKD-T1DM | Number | 2021 | 3.317811 | 11.6631 | 0.376941 |
| Incidence | Puerto Rico | Both | 10-19 years | CKD-T1DM | Number | 2021 | 3.286294 | 14.20733 | 0.294796 |
| Incidence | Latvia | Both | 10-19 years | CKD-T1DM | Number | 2021 | 3.285587 | 11.36985 | 0.417146 |
| Incidence | Lesotho | Both | 10-19 years | CKD-T1DM | Number | 2021 | 3.125777 | 13.70202 | 0.345785 |
| Incidence | Botswana | Both | 10-19 years | CKD-T1DM | Number | 2021 | 3.072871 | 12.02789 | 0.276571 |
| Incidence | Switzerland | Both | 10-19 years | CKD-T1DM | Number | 2021 | 2.853378 | 11.24284 | 0.216902 |
| Incidence | Namibia | Both | 10-19 years | CKD-T1DM | Number | 2021 | 2.666558 | 10.53214 | 0.245387 |
| Incidence | Portugal | Both | 10-19 years | CKD-T1DM | Number | 2021 | 2.659646 | 11.06384 | 0.205442 |
| Incidence | Estonia | Both | 10-19 years | CKD-T1DM | Number | 2021 | 2.592078 | 9.185729 | 0.282476 |
| Incidence | New Zealand | Both | 10-19 years | CKD-T1DM | Number | 2021 | 2.569447 | 8.499514 | 0.386304 |
| Incidence | Solomon Islands | Both | 10-19 years | CKD-T1DM | Number | 2021 | 2.561274 | 10.00975 | 0.241399 |
| Incidence | Sweden | Both | 10-19 years | CKD-T1DM | Number | 2021 | 2.49672 | 8.547194 | 0.294961 |
| Incidence | Denmark | Both | 10-19 years | CKD-T1DM | Number | 2021 | 2.460789 | 9.851282 | 0.205978 |
| Incidence | Finland | Both | 10-19 years | CKD-T1DM | Number | 2021 | 2.368786 | 9.676171 | 0.242535 |
| Incidence | Eswatini | Both | 10-19 years | CKD-T1DM | Number | 2021 | 2.266471 | 9.35867 | 0.222928 |
| Incidence | Trinidad and Tobago | Both | 10-19 years | CKD-T1DM | Number | 2021 | 2.226335 | 9.517499 | 0.23529 |
| Incidence | Fiji | Both | 10-19 years | CKD-T1DM | Number | 2021 | 1.972286 | 8.38102 | 0.152333 |
| Incidence | Guyana | Both | 10-19 years | CKD-T1DM | Number | 2021 | 1.915371 | 7.445223 | 0.149305 |
| Incidence | Slovenia | Both | 10-19 years | CKD-T1DM | Number | 2021 | 1.901857 | 7.092995 | 0.193796 |
| Incidence | Uruguay | Both | 10-19 years | CKD-T1DM | Number | 2021 | 1.876162 | 7.490469 | 0.127403 |
| Incidence | Comoros | Both | 10-19 years | CKD-T1DM | Number | 2021 | 1.860652 | 7.092697 | 0.179683 |
| Incidence | Singapore | Both | 10-19 years | CKD-T1DM | Number | 2021 | 1.777888 | 8.249393 | 0.1223 |
| Incidence | Qatar | Both | 10-19 years | CKD-T1DM | Number | 2021 | 1.762859 | 6.854224 | 0.126372 |
| Incidence | Belize | Both | 10-19 years | CKD-T1DM | Number | 2021 | 1.746018 | 6.600564 | 0.143253 |
| Incidence | Norway | Both | 10-19 years | CKD-T1DM | Number | 2021 | 1.683983 | 4.114352 | 0.422158 |
| Incidence | Montenegro | Both | 10-19 years | CKD-T1DM | Number | 2021 | 1.5545 | 5.293916 | 0.188029 |
| Incidence | Bahrain | Both | 10-19 years | CKD-T1DM | Number | 2021 | 1.550945 | 7.081954 | 0.139167 |
| Incidence | Bhutan | Both | 10-19 years | CKD-T1DM | Number | 2021 | 1.508119 | 5.836396 | 0.156437 |
| Incidence | Suriname | Both | 10-19 years | CKD-T1DM | Number | 2021 | 1.153561 | 4.852822 | 0.09776 |
| Incidence | Bahamas | Both | 10-19 years | CKD-T1DM | Number | 2021 | 1.070884 | 4.104386 | 0.102122 |
| Incidence | Cabo Verde | Both | 10-19 years | CKD-T1DM | Number | 2021 | 1.059936 | 3.884323 | 0.086463 |
| Incidence | Sao Tome and Principe | Both | 10-19 years | CKD-T1DM | Number | 2021 | 1.056362 | 4.664889 | 0.090738 |
| Incidence | Cyprus | Both | 10-19 years | CKD-T1DM | Number | 2021 | 0.707465 | 2.823156 | 0.061201 |
| Incidence | Vanuatu | Both | 10-19 years | CKD-T1DM | Number | 2021 | 0.6675 | 2.726667 | 0.050161 |
| Incidence | Brunei Darussalam | Both | 10-19 years | CKD-T1DM | Number | 2021 | 0.600464 | 2.16635 | 0.058664 |
| Incidence | Barbados | Both | 10-19 years | CKD-T1DM | Number | 2021 | 0.55826 | 2.375556 | 0.052087 |
| Incidence | Samoa | Both | 10-19 years | CKD-T1DM | Number | 2021 | 0.515078 | 2.246993 | 0.038351 |
| Incidence | Maldives | Both | 10-19 years | CKD-T1DM | Number | 2021 | 0.467273 | 1.871666 | 0.032815 |
| Incidence | Kiribati | Both | 10-19 years | CKD-T1DM | Number | 2021 | 0.365431 | 1.581429 | 0.029298 |
| Incidence | Micronesia (Federated States of) | Both | 10-19 years | CKD-T1DM | Number | 2021 | 0.347548 | 1.453659 | 0.033887 |
| Incidence | Saint Lucia | Both | 10-19 years | CKD-T1DM | Number | 2021 | 0.32733 | 1.265979 | 0.032951 |
| Incidence | Grenada | Both | 10-19 years | CKD-T1DM | Number | 2021 | 0.322977 | 1.319005 | 0.02403 |
| Incidence | Saint Vincent and the Grenadines | Both | 10-19 years | CKD-T1DM | Number | 2021 | 0.283656 | 1.188225 | 0.025906 |
| Incidence | Luxembourg | Both | 10-19 years | CKD-T1DM | Number | 2021 | 0.267495 | 1.111138 | 0.016415 |
| Incidence | Dominica | Both | 10-19 years | CKD-T1DM | Number | 2021 | 0.217821 | 0.945487 | 0.019352 |
| Incidence | Antigua and Barbuda | Both | 10-19 years | CKD-T1DM | Number | 2021 | 0.211285 | 0.758717 | 0.020258 |
| Incidence | Tonga | Both | 10-19 years | CKD-T1DM | Number | 2021 | 0.204937 | 0.745379 | 0.016386 |
| Incidence | Guam | Both | 10-19 years | CKD-T1DM | Number | 2021 | 0.204481 | 0.855565 | 0.015777 |
| Incidence | Malta | Both | 10-19 years | CKD-T1DM | Number | 2021 | 0.17966 | 0.643428 | 0.016073 |
| Incidence | United States Virgin Islands | Both | 10-19 years | CKD-T1DM | Number | 2021 | 0.14626 | 0.577682 | 0.016855 |
| Incidence | Seychelles | Both | 10-19 years | CKD-T1DM | Number | 2021 | 0.140875 | 0.601843 | 0.011885 |
| Incidence | Iceland | Both | 10-19 years | CKD-T1DM | Number | 2021 | 0.135497 | 0.595259 | 0.009264 |
| Incidence | Marshall Islands | Both | 10-19 years | CKD-T1DM | Number | 2021 | 0.117178 | 0.529008 | 0.010078 |
| Incidence | Saint Kitts and Nevis | Both | 10-19 years | CKD-T1DM | Number | 2021 | 0.116651 | 0.477005 | 0.011813 |
| Incidence | American Samoa | Both | 10-19 years | CKD-T1DM | Number | 2021 | 0.115513 | 0.46891 | 0.009274 |
| Incidence | Northern Mariana Islands | Both | 10-19 years | CKD-T1DM | Number | 2021 | 0.100088 | 0.366319 | 0.008509 |
| Incidence | Bermuda | Both | 10-19 years | CKD-T1DM | Number | 2021 | 0.060643 | 0.229281 | 0.004725 |
| Incidence | Palau | Both | 10-19 years | CKD-T1DM | Number | 2021 | 0.040734 | 0.173275 | 0.003176 |
| Incidence | Nauru | Both | 10-19 years | CKD-T1DM | Number | 2021 | 0.033934 | 0.154536 | 0.002614 |
| Incidence | Andorra | Both | 10-19 years | CKD-T1DM | Number | 2021 | 0.029964 | 0.122 | 0.002273 |
| Incidence | Greenland | Both | 10-19 years | CKD-T1DM | Number | 2021 | 0.029591 | 0.115011 | 0.002657 |
| Incidence | Tuvalu | Both | 10-19 years | CKD-T1DM | Number | 2021 | 0.026909 | 0.112755 | 0.001985 |
| Incidence | Cook Islands | Both | 10-19 years | CKD-T1DM | Number | 2021 | 0.025554 | 0.110307 | 0.00191 |
| Incidence | San Marino | Both | 10-19 years | CKD-T1DM | Number | 2021 | 0.011983 | 0.051733 | 0.000996 |
| Incidence | Monaco | Both | 10-19 years | CKD-T1DM | Number | 2021 | 0.011901 | 0.051476 | 0.000897 |
| Incidence | Niue | Both | 10-19 years | CKD-T1DM | Number | 2021 | 0.002993 | 0.012295 | 0.000225 |
| Incidence | Tokelau | Both | 10-19 years | CKD-T1DM | Number | 2021 | 0.002676 | 0.01085 | 0.000204 |

Appendix

**Appendix 14：Global disease burden distribution of Chronic kidney disease due to diabetes mellitus type 1 in children and adolescents from 1990 to 2021**

| **measure** | **Location** | **sex** | **age** | **cause** | **metric** | **year** | **val** | **upper** | **lower** |
| --- | --- | --- | --- | --- | --- | --- | --- | --- | --- |
| Deaths | Indonesia | Both | 10-19 years | CKD-T1DM | Number | 2021 | 134.8314242 | 219.6063966 | 72.32770832 |
| Deaths | China | Both | 10-19 years | CKD-T1DM | Number | 2021 | 126.8024609 | 191.3337032 | 73.26488248 |
| Deaths | Philippines | Both | 10-19 years | CKD-T1DM | Number | 2021 | 74.28863602 | 112.8605218 | 43.37812528 |
| Deaths | Ethiopia | Both | 10-19 years | CKD-T1DM | Number | 2021 | 45.95966398 | 78.62765691 | 24.09723235 |
| Deaths | Myanmar | Both | 10-19 years | CKD-T1DM | Number | 2021 | 35.99828637 | 58.16064562 | 18.81113512 |
| Deaths | India | Both | 10-19 years | CKD-T1DM | Number | 2021 | 23.96389924 | 42.57355787 | 11.76142718 |
| Deaths | Viet Nam | Both | 10-19 years | CKD-T1DM | Number | 2021 | 23.12798581 | 38.33797342 | 10.9478193 |
| Deaths | Pakistan | Both | 10-19 years | CKD-T1DM | Number | 2021 | 15.14961404 | 25.94348781 | 8.134972383 |
| Deaths | Nigeria | Both | 10-19 years | CKD-T1DM | Number | 2021 | 13.23060736 | 25.54320641 | 5.798976499 |
| Deaths | Thailand | Both | 10-19 years | CKD-T1DM | Number | 2021 | 13.12752549 | 21.39942404 | 6.985368797 |
| Deaths | Democratic Republic of the Congo | Both | 10-19 years | CKD-T1DM | Number | 2021 | 9.98018107 | 19.96845581 | 4.293103818 |
| Deaths | Malaysia | Both | 10-19 years | CKD-T1DM | Number | 2021 | 6.966555523 | 11.48815295 | 3.640269729 |
| Deaths | Mexico | Both | 10-19 years | CKD-T1DM | Number | 2021 | 6.744618865 | 11.57677227 | 3.585332488 |
| Deaths | Cambodia | Both | 10-19 years | CKD-T1DM | Number | 2021 | 6.253486816 | 11.01901386 | 2.914319539 |
| Deaths | Kenya | Both | 10-19 years | CKD-T1DM | Number | 2021 | 6.005707742 | 11.12572947 | 2.969226624 |
| Deaths | Uganda | Both | 10-19 years | CKD-T1DM | Number | 2021 | 5.669007399 | 10.43978707 | 2.664983757 |
| Deaths | Cameroon | Both | 10-19 years | CKD-T1DM | Number | 2021 | 5.490299612 | 10.21475758 | 2.445510071 |
| Deaths | Brazil | Both | 10-19 years | CKD-T1DM | Number | 2021 | 5.1646834 | 8.668138802 | 2.584163291 |
| Deaths | Mozambique | Both | 10-19 years | CKD-T1DM | Number | 2021 | 4.983549162 | 9.667949447 | 2.322953397 |
| Deaths | Democratic People's Republic of Korea | Both | 10-19 years | CKD-T1DM | Number | 2021 | 4.971304778 | 8.352476694 | 2.59392378 |
| Deaths | Lao People's Democratic Republic | Both | 10-19 years | CKD-T1DM | Number | 2021 | 4.96601211 | 8.478940256 | 2.606154727 |
| Deaths | Sri Lanka | Both | 10-19 years | CKD-T1DM | Number | 2021 | 4.598927753 | 7.894628931 | 2.345472293 |
| Deaths | Papua New Guinea | Both | 10-19 years | CKD-T1DM | Number | 2021 | 4.374559665 | 7.877771059 | 2.103813782 |
| Deaths | Somalia | Both | 10-19 years | CKD-T1DM | Number | 2021 | 3.386188245 | 6.737645252 | 1.414654188 |
| Deaths | Ghana | Both | 10-19 years | CKD-T1DM | Number | 2021 | 3.358972171 | 6.347488275 | 1.606038913 |
| Deaths | Zambia | Both | 10-19 years | CKD-T1DM | Number | 2021 | 3.261333762 | 7.380809402 | 1.312546313 |
| Deaths | Malawi | Both | 10-19 years | CKD-T1DM | Number | 2021 | 3.044584805 | 6.045989795 | 1.424253535 |
| Deaths | Angola | Both | 10-19 years | CKD-T1DM | Number | 2021 | 2.827148441 | 5.439957117 | 1.307420792 |
| Deaths | Egypt | Both | 10-19 years | CKD-T1DM | Number | 2021 | 2.824904405 | 5.888710227 | 1.232664645 |
| Deaths | Bangladesh | Both | 10-19 years | CKD-T1DM | Number | 2021 | 2.762643727 | 5.390268411 | 1.354065565 |
| Deaths | Haiti | Both | 10-19 years | CKD-T1DM | Number | 2021 | 2.710258145 | 6.012457413 | 1.134533754 |
| Deaths | Madagascar | Both | 10-19 years | CKD-T1DM | Number | 2021 | 2.695691032 | 5.111557127 | 1.175383887 |
| Deaths | United Republic of Tanzania | Both | 10-19 years | CKD-T1DM | Number | 2021 | 2.406492164 | 4.45621293 | 1.154277486 |
| Deaths | Peru | Both | 10-19 years | CKD-T1DM | Number | 2021 | 2.155088499 | 3.86394805 | 0.974304416 |
| Deaths | Dominican Republic | Both | 10-19 years | CKD-T1DM | Number | 2021 | 1.984666926 | 3.416676085 | 0.918211395 |
| Deaths | South Sudan | Both | 10-19 years | CKD-T1DM | Number | 2021 | 1.98038435 | 3.612018023 | 0.892549794 |
| Deaths | C么te d'Ivoire | Both | 10-19 years | CKD-T1DM | Number | 2021 | 1.693132669 | 3.146522076 | 0.725500438 |
| Deaths | Taiwan (Province of China) | Both | 10-19 years | CKD-T1DM | Number | 2021 | 1.66452681 | 2.491019344 | 0.990221965 |
| Deaths | Iran (Islamic Republic of) | Both | 10-19 years | CKD-T1DM | Number | 2021 | 1.613703976 | 2.897463059 | 0.794308706 |
| Deaths | Mali | Both | 10-19 years | CKD-T1DM | Number | 2021 | 1.608707049 | 2.985490872 | 0.746795154 |
| Deaths | Burkina Faso | Both | 10-19 years | CKD-T1DM | Number | 2021 | 1.586564396 | 3.024450639 | 0.672957269 |
| Deaths | South Africa | Both | 10-19 years | CKD-T1DM | Number | 2021 | 1.539812123 | 2.798314638 | 0.755629027 |
| Deaths | Senegal | Both | 10-19 years | CKD-T1DM | Number | 2021 | 1.45130948 | 2.808707581 | 0.604234936 |
| Deaths | Burundi | Both | 10-19 years | CKD-T1DM | Number | 2021 | 1.381214962 | 2.735096255 | 0.634388588 |
| Deaths | Afghanistan | Both | 10-19 years | CKD-T1DM | Number | 2021 | 1.321717919 | 3.097327877 | 0.491535545 |
| Deaths | Chad | Both | 10-19 years | CKD-T1DM | Number | 2021 | 1.278583867 | 2.60442566 | 0.565466325 |
| Deaths | Ecuador | Both | 10-19 years | CKD-T1DM | Number | 2021 | 1.133392018 | 1.979531488 | 0.552023693 |
| Deaths | Guatemala | Both | 10-19 years | CKD-T1DM | Number | 2021 | 1.113397575 | 1.942814795 | 0.546532008 |
| Deaths | Bolivia (Plurinational State of) | Both | 10-19 years | CKD-T1DM | Number | 2021 | 1.096353492 | 2.063953616 | 0.487848066 |
| Deaths | Rwanda | Both | 10-19 years | CKD-T1DM | Number | 2021 | 0.99605798 | 1.892687844 | 0.455099324 |
| Deaths | Venezuela (Bolivarian Republic of) | Both | 10-19 years | CKD-T1DM | Number | 2021 | 0.943077823 | 1.669007543 | 0.44508474 |
| Deaths | Benin | Both | 10-19 years | CKD-T1DM | Number | 2021 | 0.926106865 | 1.663670492 | 0.432618974 |
| Deaths | T眉rkiye | Both | 10-19 years | CKD-T1DM | Number | 2021 | 0.907130716 | 1.697402149 | 0.403449861 |
| Deaths | Iraq | Both | 10-19 years | CKD-T1DM | Number | 2021 | 0.902190737 | 1.898831153 | 0.391061347 |
| Deaths | Niger | Both | 10-19 years | CKD-T1DM | Number | 2021 | 0.889927309 | 1.815137141 | 0.347746064 |
| Deaths | Mauritius | Both | 10-19 years | CKD-T1DM | Number | 2021 | 0.838309727 | 1.281672423 | 0.499414491 |
| Deaths | Nepal | Both | 10-19 years | CKD-T1DM | Number | 2021 | 0.828884469 | 1.604499547 | 0.370846407 |
| Deaths | Saudi Arabia | Both | 10-19 years | CKD-T1DM | Number | 2021 | 0.821976128 | 1.608537755 | 0.345926088 |
| Deaths | Central African Republic | Both | 10-19 years | CKD-T1DM | Number | 2021 | 0.812367889 | 1.589007508 | 0.356270188 |
| Deaths | Sudan | Both | 10-19 years | CKD-T1DM | Number | 2021 | 0.778517398 | 1.654027794 | 0.313516664 |
| Deaths | Uzbekistan | Both | 10-19 years | CKD-T1DM | Number | 2021 | 0.736767905 | 1.304450366 | 0.354178568 |
| Deaths | Fiji | Both | 10-19 years | CKD-T1DM | Number | 2021 | 0.708670615 | 1.204391251 | 0.355922912 |
| Deaths | Eritrea | Both | 10-19 years | CKD-T1DM | Number | 2021 | 0.700520122 | 1.443073062 | 0.299620618 |
| Deaths | Zimbabwe | Both | 10-19 years | CKD-T1DM | Number | 2021 | 0.656788494 | 1.233801991 | 0.289202452 |
| Deaths | Guinea | Both | 10-19 years | CKD-T1DM | Number | 2021 | 0.642111471 | 1.258986206 | 0.278119591 |
| Deaths | Syrian Arab Republic | Both | 10-19 years | CKD-T1DM | Number | 2021 | 0.636382122 | 1.225774759 | 0.27755209 |
| Deaths | Russian Federation | Both | 10-19 years | CKD-T1DM | Number | 2021 | 0.635460384 | 1.085737137 | 0.349089324 |
| Deaths | Congo | Both | 10-19 years | CKD-T1DM | Number | 2021 | 0.631017613 | 1.17573786 | 0.288016514 |
| Deaths | Timor-Leste | Both | 10-19 years | CKD-T1DM | Number | 2021 | 0.628199247 | 1.120462816 | 0.3196846 |
| Deaths | Colombia | Both | 10-19 years | CKD-T1DM | Number | 2021 | 0.565829777 | 0.966276727 | 0.280679894 |
| Deaths | Algeria | Both | 10-19 years | CKD-T1DM | Number | 2021 | 0.562374918 | 1.072288236 | 0.245385224 |
| Deaths | Liberia | Both | 10-19 years | CKD-T1DM | Number | 2021 | 0.522866629 | 0.962847506 | 0.231241634 |
| Deaths | Togo | Both | 10-19 years | CKD-T1DM | Number | 2021 | 0.514078459 | 0.949799123 | 0.222837882 |
| Deaths | Sierra Leone | Both | 10-19 years | CKD-T1DM | Number | 2021 | 0.496945065 | 1.00651788 | 0.224688241 |
| Deaths | Cuba | Both | 10-19 years | CKD-T1DM | Number | 2021 | 0.487339365 | 0.831904672 | 0.261293995 |
| Deaths | Argentina | Both | 10-19 years | CKD-T1DM | Number | 2021 | 0.48534552 | 0.839143546 | 0.242920905 |
| Deaths | United States of America | Both | 10-19 years | CKD-T1DM | Number | 2021 | 0.46257297 | 0.763914836 | 0.238193439 |
| Deaths | El Salvador | Both | 10-19 years | CKD-T1DM | Number | 2021 | 0.45792863 | 0.792332828 | 0.22983382 |
| Deaths | Solomon Islands | Both | 10-19 years | CKD-T1DM | Number | 2021 | 0.396128316 | 0.674007305 | 0.20486193 |
| Deaths | Morocco | Both | 10-19 years | CKD-T1DM | Number | 2021 | 0.390146067 | 0.782398041 | 0.171840228 |
| Deaths | Paraguay | Both | 10-19 years | CKD-T1DM | Number | 2021 | 0.299565861 | 0.541749348 | 0.141974491 |
| Deaths | Jordan | Both | 10-19 years | CKD-T1DM | Number | 2021 | 0.297170877 | 0.558263207 | 0.139806889 |
| Deaths | Nicaragua | Both | 10-19 years | CKD-T1DM | Number | 2021 | 0.287833174 | 0.514731399 | 0.135831565 |
| Deaths | Jamaica | Both | 10-19 years | CKD-T1DM | Number | 2021 | 0.285948176 | 0.505393628 | 0.1409724 |
| Deaths | Japan | Both | 10-19 years | CKD-T1DM | Number | 2021 | 0.269467586 | 0.469312813 | 0.150184102 |
| Deaths | Yemen | Both | 10-19 years | CKD-T1DM | Number | 2021 | 0.268219443 | 0.709716781 | 0.094672375 |
| Deaths | Turkmenistan | Both | 10-19 years | CKD-T1DM | Number | 2021 | 0.257102557 | 0.469309079 | 0.120913237 |
| Deaths | Gabon | Both | 10-19 years | CKD-T1DM | Number | 2021 | 0.252771783 | 0.485200472 | 0.107436731 |
| Deaths | Mauritania | Both | 10-19 years | CKD-T1DM | Number | 2021 | 0.248941423 | 0.467887198 | 0.105461851 |
| Deaths | Trinidad and Tobago | Both | 10-19 years | CKD-T1DM | Number | 2021 | 0.2322257 | 0.408142862 | 0.116415821 |
| Deaths | Equatorial Guinea | Both | 10-19 years | CKD-T1DM | Number | 2021 | 0.221767328 | 0.433471487 | 0.082504053 |
| Deaths | Gambia | Both | 10-19 years | CKD-T1DM | Number | 2021 | 0.220317911 | 0.433872578 | 0.100905733 |
| Deaths | Guyana | Both | 10-19 years | CKD-T1DM | Number | 2021 | 0.21989494 | 0.389778047 | 0.108482022 |
| Deaths | Guinea-Bissau | Both | 10-19 years | CKD-T1DM | Number | 2021 | 0.207892714 | 0.430588725 | 0.092399507 |
| Deaths | Kazakhstan | Both | 10-19 years | CKD-T1DM | Number | 2021 | 0.203742661 | 0.375346296 | 0.09757078 |
| Deaths | Vanuatu | Both | 10-19 years | CKD-T1DM | Number | 2021 | 0.182124617 | 0.349955538 | 0.087399368 |
| Deaths | Azerbaijan | Both | 10-19 years | CKD-T1DM | Number | 2021 | 0.180581634 | 0.344886379 | 0.083387126 |
| Deaths | Republic of Korea | Both | 10-19 years | CKD-T1DM | Number | 2021 | 0.167958524 | 0.301759548 | 0.082021625 |
| Deaths | Suriname | Both | 10-19 years | CKD-T1DM | Number | 2021 | 0.164270381 | 0.284702665 | 0.079755948 |
| Deaths | Honduras | Both | 10-19 years | CKD-T1DM | Number | 2021 | 0.144543484 | 0.284573958 | 0.062626788 |
| Deaths | Ukraine | Both | 10-19 years | CKD-T1DM | Number | 2021 | 0.143522696 | 0.243828435 | 0.071490064 |
| Deaths | Belize | Both | 10-19 years | CKD-T1DM | Number | 2021 | 0.138656924 | 0.235717922 | 0.070467475 |
| Deaths | Libya | Both | 10-19 years | CKD-T1DM | Number | 2021 | 0.138505606 | 0.275400862 | 0.053937227 |
| Deaths | Puerto Rico | Both | 10-19 years | CKD-T1DM | Number | 2021 | 0.118618582 | 0.191208534 | 0.065407445 |
| Deaths | Kiribati | Both | 10-19 years | CKD-T1DM | Number | 2021 | 0.117890093 | 0.226729702 | 0.051894853 |
| Deaths | Kyrgyzstan | Both | 10-19 years | CKD-T1DM | Number | 2021 | 0.116986422 | 0.216832005 | 0.055644746 |
| Deaths | Djibouti | Both | 10-19 years | CKD-T1DM | Number | 2021 | 0.107913461 | 0.199828888 | 0.047119517 |
| Deaths | Samoa | Both | 10-19 years | CKD-T1DM | Number | 2021 | 0.106197875 | 0.175583016 | 0.053611129 |
| Deaths | Panama | Both | 10-19 years | CKD-T1DM | Number | 2021 | 0.106023362 | 0.181170559 | 0.050291379 |
| Deaths | Palestine | Both | 10-19 years | CKD-T1DM | Number | 2021 | 0.096580489 | 0.186433254 | 0.042627446 |
| Deaths | Micronesia (Federated States of) | Both | 10-19 years | CKD-T1DM | Number | 2021 | 0.093835153 | 0.163760421 | 0.046101546 |
| Deaths | Maldives | Both | 10-19 years | CKD-T1DM | Number | 2021 | 0.093155454 | 0.152821981 | 0.05043623 |
| Deaths | Chile | Both | 10-19 years | CKD-T1DM | Number | 2021 | 0.088837211 | 0.153533598 | 0.044799195 |
| Deaths | Costa Rica | Both | 10-19 years | CKD-T1DM | Number | 2021 | 0.082585983 | 0.141967087 | 0.041133905 |
| Deaths | Bahamas | Both | 10-19 years | CKD-T1DM | Number | 2021 | 0.081388106 | 0.142068263 | 0.041932794 |
| Deaths | Tunisia | Both | 10-19 years | CKD-T1DM | Number | 2021 | 0.080979703 | 0.147322196 | 0.034129303 |
| Deaths | Eswatini | Both | 10-19 years | CKD-T1DM | Number | 2021 | 0.076022763 | 0.142175306 | 0.031743337 |
| Deaths | American Samoa | Both | 10-19 years | CKD-T1DM | Number | 2021 | 0.075262422 | 0.120055207 | 0.039447165 |
| Deaths | Lesotho | Both | 10-19 years | CKD-T1DM | Number | 2021 | 0.073734402 | 0.138512416 | 0.029516958 |
| Deaths | United Kingdom | Both | 10-19 years | CKD-T1DM | Number | 2021 | 0.073087016 | 0.125025903 | 0.038229517 |
| Deaths | Comoros | Both | 10-19 years | CKD-T1DM | Number | 2021 | 0.062307352 | 0.115305904 | 0.028176699 |
| Deaths | Poland | Both | 10-19 years | CKD-T1DM | Number | 2021 | 0.062008287 | 0.107388477 | 0.032483661 |
| Deaths | Germany | Both | 10-19 years | CKD-T1DM | Number | 2021 | 0.06069308 | 0.109487833 | 0.029360656 |
| Deaths | Mongolia | Both | 10-19 years | CKD-T1DM | Number | 2021 | 0.056313485 | 0.101730512 | 0.027396436 |
| Deaths | Tajikistan | Both | 10-19 years | CKD-T1DM | Number | 2021 | 0.05508071 | 0.109829917 | 0.022916247 |
| Deaths | Lebanon | Both | 10-19 years | CKD-T1DM | Number | 2021 | 0.053405312 | 0.103699791 | 0.022900208 |
| Deaths | Botswana | Both | 10-19 years | CKD-T1DM | Number | 2021 | 0.050252339 | 0.103836643 | 0.021662241 |
| Deaths | Oman | Both | 10-19 years | CKD-T1DM | Number | 2021 | 0.050215358 | 0.0969611 | 0.023201741 |
| Deaths | Marshall Islands | Both | 10-19 years | CKD-T1DM | Number | 2021 | 0.048227319 | 0.159350302 | 0.010576102 |
| Deaths | Italy | Both | 10-19 years | CKD-T1DM | Number | 2021 | 0.047905047 | 0.082401091 | 0.025158558 |
| Deaths | Namibia | Both | 10-19 years | CKD-T1DM | Number | 2021 | 0.046049561 | 0.092998744 | 0.019724031 |
| Deaths | Georgia | Both | 10-19 years | CKD-T1DM | Number | 2021 | 0.045957737 | 0.080778869 | 0.022314049 |
| Deaths | Spain | Both | 10-19 years | CKD-T1DM | Number | 2021 | 0.043067325 | 0.075165554 | 0.022786008 |
| Deaths | France | Both | 10-19 years | CKD-T1DM | Number | 2021 | 0.040770378 | 0.067540426 | 0.02199776 |
| Deaths | United Arab Emirates | Both | 10-19 years | CKD-T1DM | Number | 2021 | 0.03930037 | 0.077662089 | 0.014433699 |
| Deaths | Serbia | Both | 10-19 years | CKD-T1DM | Number | 2021 | 0.035202698 | 0.063442956 | 0.018249944 |
| Deaths | Saint Lucia | Both | 10-19 years | CKD-T1DM | Number | 2021 | 0.034141447 | 0.058344873 | 0.016868628 |
| Deaths | Barbados | Both | 10-19 years | CKD-T1DM | Number | 2021 | 0.031229096 | 0.055257305 | 0.015326591 |
| Deaths | Israel | Both | 10-19 years | CKD-T1DM | Number | 2021 | 0.030729188 | 0.054742137 | 0.014416841 |
| Deaths | United States Virgin Islands | Both | 10-19 years | CKD-T1DM | Number | 2021 | 0.027063842 | 0.050598569 | 0.013067569 |
| Deaths | Singapore | Both | 10-19 years | CKD-T1DM | Number | 2021 | 0.026538522 | 0.048565693 | 0.012488307 |
| Deaths | Kuwait | Both | 10-19 years | CKD-T1DM | Number | 2021 | 0.025853327 | 0.049498252 | 0.011706254 |
| Deaths | Guam | Both | 10-19 years | CKD-T1DM | Number | 2021 | 0.025841976 | 0.044442955 | 0.01250155 |
| Deaths | Tonga | Both | 10-19 years | CKD-T1DM | Number | 2021 | 0.025771691 | 0.044571819 | 0.012222936 |
| Deaths | Saint Vincent and the Grenadines | Both | 10-19 years | CKD-T1DM | Number | 2021 | 0.025237937 | 0.041943955 | 0.013519023 |
| Deaths | Grenada | Both | 10-19 years | CKD-T1DM | Number | 2021 | 0.025192141 | 0.041280968 | 0.013574469 |
| Deaths | Sao Tome and Principe | Both | 10-19 years | CKD-T1DM | Number | 2021 | 0.025123592 | 0.049295161 | 0.010836503 |
| Deaths | Bulgaria | Both | 10-19 years | CKD-T1DM | Number | 2021 | 0.02510008 | 0.04572551 | 0.012392162 |
| Deaths | Dominica | Both | 10-19 years | CKD-T1DM | Number | 2021 | 0.022376494 | 0.037563978 | 0.011040455 |
| Deaths | Canada | Both | 10-19 years | CKD-T1DM | Number | 2021 | 0.021556299 | 0.040187351 | 0.010075314 |
| Deaths | Armenia | Both | 10-19 years | CKD-T1DM | Number | 2021 | 0.021469677 | 0.039688403 | 0.010133051 |
| Deaths | Cabo Verde | Both | 10-19 years | CKD-T1DM | Number | 2021 | 0.021368025 | 0.04089476 | 0.009545778 |
| Deaths | Romania | Both | 10-19 years | CKD-T1DM | Number | 2021 | 0.021147923 | 0.03860348 | 0.009990737 |
| Deaths | Uruguay | Both | 10-19 years | CKD-T1DM | Number | 2021 | 0.018831991 | 0.032387108 | 0.009260097 |
| Deaths | Northern Mariana Islands | Both | 10-19 years | CKD-T1DM | Number | 2021 | 0.018640087 | 0.031825909 | 0.009840946 |
| Deaths | Antigua and Barbuda | Both | 10-19 years | CKD-T1DM | Number | 2021 | 0.017746743 | 0.029411861 | 0.008874521 |
| Deaths | Bahrain | Both | 10-19 years | CKD-T1DM | Number | 2021 | 0.016168633 | 0.030793686 | 0.007097718 |
| Deaths | Palau | Both | 10-19 years | CKD-T1DM | Number | 2021 | 0.016007055 | 0.028293844 | 0.007128392 |
| Deaths | Bhutan | Both | 10-19 years | CKD-T1DM | Number | 2021 | 0.015789537 | 0.03017161 | 0.006458283 |
| Deaths | Slovakia | Both | 10-19 years | CKD-T1DM | Number | 2021 | 0.015444064 | 0.027944588 | 0.007608517 |
| Deaths | Estonia | Both | 10-19 years | CKD-T1DM | Number | 2021 | 0.014668149 | 0.025508386 | 0.007370532 |
| Deaths | Greece | Both | 10-19 years | CKD-T1DM | Number | 2021 | 0.014528934 | 0.023100623 | 0.007723365 |
| Deaths | Qatar | Both | 10-19 years | CKD-T1DM | Number | 2021 | 0.014450044 | 0.028332119 | 0.006396441 |
| Deaths | Saint Kitts and Nevis | Both | 10-19 years | CKD-T1DM | Number | 2021 | 0.014059598 | 0.024499815 | 0.007185848 |
| Deaths | Bosnia and Herzegovina | Both | 10-19 years | CKD-T1DM | Number | 2021 | 0.014036308 | 0.024856049 | 0.006925543 |
| Deaths | Czechia | Both | 10-19 years | CKD-T1DM | Number | 2021 | 0.013621089 | 0.026850195 | 0.006496833 |
| Deaths | Portugal | Both | 10-19 years | CKD-T1DM | Number | 2021 | 0.012821487 | 0.022200917 | 0.006529169 |
| Deaths | Hungary | Both | 10-19 years | CKD-T1DM | Number | 2021 | 0.012538041 | 0.02470951 | 0.005528329 |
| Deaths | Nauru | Both | 10-19 years | CKD-T1DM | Number | 2021 | 0.011463253 | 0.019227441 | 0.005394549 |
| Deaths | Belarus | Both | 10-19 years | CKD-T1DM | Number | 2021 | 0.011305958 | 0.019873415 | 0.005637157 |
| Deaths | Netherlands | Both | 10-19 years | CKD-T1DM | Number | 2021 | 0.010597438 | 0.017767814 | 0.005500201 |
| Deaths | Republic of Moldova | Both | 10-19 years | CKD-T1DM | Number | 2021 | 0.010204055 | 0.017555475 | 0.005082662 |
| Deaths | Austria | Both | 10-19 years | CKD-T1DM | Number | 2021 | 0.009830981 | 0.015900312 | 0.005391335 |
| Deaths | Brunei Darussalam | Both | 10-19 years | CKD-T1DM | Number | 2021 | 0.009621844 | 0.017729089 | 0.004439864 |
| Deaths | Belgium | Both | 10-19 years | CKD-T1DM | Number | 2021 | 0.009254085 | 0.015484419 | 0.00470933 |
| Deaths | Albania | Both | 10-19 years | CKD-T1DM | Number | 2021 | 0.008597403 | 0.016559904 | 0.004106052 |
| Deaths | North Macedonia | Both | 10-19 years | CKD-T1DM | Number | 2021 | 0.007675481 | 0.014643494 | 0.003475694 |
| Deaths | Australia | Both | 10-19 years | CKD-T1DM | Number | 2021 | 0.007659546 | 0.01374249 | 0.003472779 |
| Deaths | Croatia | Both | 10-19 years | CKD-T1DM | Number | 2021 | 0.007495547 | 0.01351501 | 0.003510991 |
| Deaths | Tuvalu | Both | 10-19 years | CKD-T1DM | Number | 2021 | 0.006893291 | 0.012082028 | 0.003351432 |
| Deaths | Lithuania | Both | 10-19 years | CKD-T1DM | Number | 2021 | 0.006488971 | 0.011276074 | 0.003301687 |
| Deaths | New Zealand | Both | 10-19 years | CKD-T1DM | Number | 2021 | 0.006335351 | 0.010401897 | 0.003279446 |
| Deaths | Latvia | Both | 10-19 years | CKD-T1DM | Number | 2021 | 0.005278056 | 0.009394391 | 0.00256398 |
| Deaths | Switzerland | Both | 10-19 years | CKD-T1DM | Number | 2021 | 0.005215652 | 0.008635701 | 0.002739109 |
| Deaths | Sweden | Both | 10-19 years | CKD-T1DM | Number | 2021 | 0.004257308 | 0.007323714 | 0.002233084 |
| Deaths | Montenegro | Both | 10-19 years | CKD-T1DM | Number | 2021 | 0.004213933 | 0.007964826 | 0.001990449 |
| Deaths | Norway | Both | 10-19 years | CKD-T1DM | Number | 2021 | 0.003212852 | 0.005345946 | 0.001679169 |
| Deaths | Finland | Both | 10-19 years | CKD-T1DM | Number | 2021 | 0.003122456 | 0.005053391 | 0.001664877 |
| Deaths | Ireland | Both | 10-19 years | CKD-T1DM | Number | 2021 | 0.003111426 | 0.005387516 | 0.001545908 |
| Deaths | Denmark | Both | 10-19 years | CKD-T1DM | Number | 2021 | 0.002942726 | 0.004701619 | 0.001607897 |
| Deaths | Seychelles | Both | 10-19 years | CKD-T1DM | Number | 2021 | 0.002786382 | 0.00523351 | 0.001332232 |
| Deaths | Niue | Both | 10-19 years | CKD-T1DM | Number | 2021 | 0.002745339 | 0.005257423 | 0.001175417 |
| Deaths | Cook Islands | Both | 10-19 years | CKD-T1DM | Number | 2021 | 0.002695666 | 0.00443427 | 0.001438653 |
| Deaths | Bermuda | Both | 10-19 years | CKD-T1DM | Number | 2021 | 0.002337623 | 0.004042274 | 0.001150925 |
| Deaths | Tokelau | Both | 10-19 years | CKD-T1DM | Number | 2021 | 0.001891723 | 0.003325764 | 0.000970754 |
| Deaths | Slovenia | Both | 10-19 years | CKD-T1DM | Number | 2021 | 0.001536301 | 0.002901661 | 0.000736815 |
| Deaths | Malta | Both | 10-19 years | CKD-T1DM | Number | 2021 | 0.00064307 | 0.00113023 | 0.000322305 |
| Deaths | Cyprus | Both | 10-19 years | CKD-T1DM | Number | 2021 | 0.00062007 | 0.001292856 | 0.00028161 |
| Deaths | Luxembourg | Both | 10-19 years | CKD-T1DM | Number | 2021 | 0.000459638 | 0.00081105 | 0.00021762 |
| Deaths | Iceland | Both | 10-19 years | CKD-T1DM | Number | 2021 | 0.000143061 | 0.000254686 | 7.35E-05 |
| Deaths | Andorra | Both | 10-19 years | CKD-T1DM | Number | 2021 | 6.23E-05 | 0.000115186 | 2.82E-05 |
| Deaths | Monaco | Both | 10-19 years | CKD-T1DM | Number | 2021 | 3.63E-05 | 6.41E-05 | 1.77E-05 |
| Deaths | Greenland | Both | 10-19 years | CKD-T1DM | Number | 2021 | 3.27E-05 | 6.91E-05 | 1.34E-05 |
| Deaths | San Marino | Both | 10-19 years | CKD-T1DM | Number | 2021 | 1.39E-05 | 2.56E-05 | 6.56E-06 |

Appendix

**Appendix 15：Global disease burden distribution of Chronic kidney disease due to diabetes mellitus type 1 in children and adolescents from 1990 to 2021**

| **measure** | **location** | **sex** | **age** | **cause** | **metric** | **year** | **val** | **upper** | **lower** |
| --- | --- | --- | --- | --- | --- | --- | --- | --- | --- |
| Incidence | Uzbekistan | Both | Age-standardized | CKD-T1DM | Rate | 2021 | 0.999393794 | 3.526132726 | 0.114652801 |
| Incidence | Turkmenistan | Both | Age-standardized | CKD-T1DM | Rate | 2021 | 0.866401324 | 3.107322561 | 0.091937969 |
| Incidence | Azerbaijan | Both | Age-standardized | CKD-T1DM | Rate | 2021 | 0.738743194 | 2.867693854 | 0.077608801 |
| Incidence | Kyrgyzstan | Both | Age-standardized | CKD-T1DM | Rate | 2021 | 0.572523652 | 2.008453532 | 0.058791254 |
| Incidence | Mongolia | Both | Age-standardized | CKD-T1DM | Rate | 2021 | 0.513861308 | 2.153630487 | 0.0463962 |
| Incidence | Mauritius | Both | Age-standardized | CKD-T1DM | Rate | 2021 | 0.450026479 | 1.71093467 | 0.040340453 |
| Incidence | El Salvador | Both | Age-standardized | CKD-T1DM | Rate | 2021 | 0.443724833 | 1.648325645 | 0.037135236 |
| Incidence | Georgia | Both | Age-standardized | CKD-T1DM | Rate | 2021 | 0.432639838 | 1.512925778 | 0.052723079 |
| Incidence | Armenia | Both | Age-standardized | CKD-T1DM | Rate | 2021 | 0.413089079 | 1.568556123 | 0.039684199 |
| Incidence | Nicaragua | Both | Age-standardized | CKD-T1DM | Rate | 2021 | 0.409133345 | 1.598595535 | 0.033018728 |
| Incidence | Kazakhstan | Both | Age-standardized | CKD-T1DM | Rate | 2021 | 0.404972219 | 1.588752494 | 0.037792424 |
| Incidence | Tajikistan | Both | Age-standardized | CKD-T1DM | Rate | 2021 | 0.401834145 | 1.597932121 | 0.037652756 |
| Incidence | Bulgaria | Both | Age-standardized | CKD-T1DM | Rate | 2021 | 0.385425851 | 1.413176431 | 0.045715588 |
| Incidence | Albania | Both | Age-standardized | CKD-T1DM | Rate | 2021 | 0.37402465 | 1.287534142 | 0.046046944 |
| Incidence | Sao Tome and Principe | Both | Age-standardized | CKD-T1DM | Rate | 2021 | 0.36154356 | 1.596573912 | 0.031055301 |
| Incidence | Mexico | Both | Age-standardized | CKD-T1DM | Rate | 2021 | 0.361203181 | 0.624070942 | 0.199285607 |
| Incidence | Guatemala | Both | Age-standardized | CKD-T1DM | Rate | 2021 | 0.354996525 | 1.485320251 | 0.032212682 |
| Incidence | Montenegro | Both | Age-standardized | CKD-T1DM | Rate | 2021 | 0.349040709 | 1.188673081 | 0.042219116 |
| Incidence | Grenada | Both | Age-standardized | CKD-T1DM | Rate | 2021 | 0.348364122 | 1.422684727 | 0.025919175 |
| Incidence | Philippines | Both | Age-standardized | CKD-T1DM | Rate | 2021 | 0.341581563 | 0.51255481 | 0.209372885 |
| Incidence | Belize | Both | Age-standardized | CKD-T1DM | Rate | 2021 | 0.336199727 | 1.270953542 | 0.027583723 |
| Incidence | Saudi Arabia | Both | Age-standardized | CKD-T1DM | Rate | 2021 | 0.335904449 | 1.290376363 | 0.02969493 |
| Incidence | Dominica | Both | Age-standardized | CKD-T1DM | Rate | 2021 | 0.335521601 | 1.456381769 | 0.029808546 |
| Incidence | Cameroon | Both | Age-standardized | CKD-T1DM | Rate | 2021 | 0.332145474 | 1.325307007 | 0.026944852 |
| Incidence | Myanmar | Both | Age-standardized | CKD-T1DM | Rate | 2021 | 0.322465988 | 1.272217324 | 0.031999631 |
| Incidence | Estonia | Both | Age-standardized | CKD-T1DM | Rate | 2021 | 0.319915607 | 1.133707203 | 0.034863288 |
| Incidence | Russian Federation | Both | Age-standardized | CKD-T1DM | Rate | 2021 | 0.319884392 | 0.556284967 | 0.156510966 |
| Incidence | Belarus | Both | Age-standardized | CKD-T1DM | Rate | 2021 | 0.300119409 | 1.167126458 | 0.03487578 |
| Incidence | Latvia | Both | Age-standardized | CKD-T1DM | Rate | 2021 | 0.299215972 | 1.035443389 | 0.03798917 |
| Incidence | Solomon Islands | Both | Age-standardized | CKD-T1DM | Rate | 2021 | 0.296614454 | 1.159202912 | 0.027955812 |
| Incidence | Guinea-Bissau | Both | Age-standardized | CKD-T1DM | Rate | 2021 | 0.295800561 | 1.334278509 | 0.023910669 |
| Incidence | Palau | Both | Age-standardized | CKD-T1DM | Rate | 2021 | 0.294505547 | 1.252760479 | 0.0229597 |
| Incidence | Indonesia | Both | Age-standardized | CKD-T1DM | Rate | 2021 | 0.293875948 | 0.52672818 | 0.150428794 |
| Incidence | United Arab Emirates | Both | Age-standardized | CKD-T1DM | Rate | 2021 | 0.293628798 | 1.225080667 | 0.024166791 |
| Incidence | Antigua and Barbuda | Both | Age-standardized | CKD-T1DM | Rate | 2021 | 0.290523826 | 1.043261702 | 0.027855975 |
| Incidence | Micronesia (Federated States of) | Both | Age-standardized | CKD-T1DM | Rate | 2021 | 0.276701027 | 1.157333527 | 0.026979566 |
| Incidence | Saint Vincent and the Grenadines | Both | Age-standardized | CKD-T1DM | Rate | 2021 | 0.276470559 | 1.158126537 | 0.025249707 |
| Incidence | Republic of Moldova | Both | Age-standardized | CKD-T1DM | Rate | 2021 | 0.27459971 | 0.907528545 | 0.03284457 |
| Incidence | Lithuania | Both | Age-standardized | CKD-T1DM | Rate | 2021 | 0.273897238 | 0.946050166 | 0.030660658 |
| Incidence | Ukraine | Both | Age-standardized | CKD-T1DM | Rate | 2021 | 0.273840277 | 0.947961093 | 0.034739027 |
| Incidence | Bahamas | Both | Age-standardized | CKD-T1DM | Rate | 2021 | 0.272376724 | 1.043940619 | 0.025974354 |
| Incidence | Malawi | Both | Age-standardized | CKD-T1DM | Rate | 2021 | 0.264307749 | 0.960215809 | 0.033025288 |
| Incidence | Togo | Both | Age-standardized | CKD-T1DM | Rate | 2021 | 0.261561053 | 0.958203519 | 0.027419257 |
| Incidence | Saint Kitts and Nevis | Both | Age-standardized | CKD-T1DM | Rate | 2021 | 0.261115428 | 1.067741265 | 0.02644264 |
| Incidence | Mozambique | Both | Age-standardized | CKD-T1DM | Rate | 2021 | 0.260754328 | 0.994801174 | 0.02990806 |
| Incidence | C么te d'Ivoire | Both | Age-standardized | CKD-T1DM | Rate | 2021 | 0.260076271 | 0.996552497 | 0.022795853 |
| Incidence | Syrian Arab Republic | Both | Age-standardized | CKD-T1DM | Rate | 2021 | 0.259795844 | 1.083722413 | 0.023711567 |
| Incidence | Barbados | Both | Age-standardized | CKD-T1DM | Rate | 2021 | 0.258330986 | 1.099272647 | 0.024102943 |
| Incidence | United States Virgin Islands | Both | Age-standardized | CKD-T1DM | Rate | 2021 | 0.258004368 | 1.019035363 | 0.029731889 |
| Incidence | Kiribati | Both | Age-standardized | CKD-T1DM | Rate | 2021 | 0.25450948 | 1.101407842 | 0.020404733 |
| Incidence | Lao People's Democratic Republic | Both | Age-standardized | CKD-T1DM | Rate | 2021 | 0.248782221 | 1.011201755 | 0.021412231 |
| Incidence | Guinea | Both | Age-standardized | CKD-T1DM | Rate | 2021 | 0.248489876 | 0.97034483 | 0.022968103 |
| Incidence | Bosnia and Herzegovina | Both | Age-standardized | CKD-T1DM | Rate | 2021 | 0.247865626 | 0.78747953 | 0.028775731 |
| Incidence | North Macedonia | Both | Age-standardized | CKD-T1DM | Rate | 2021 | 0.246708467 | 0.867254286 | 0.028028913 |
| Incidence | Guyana | Both | Age-standardized | CKD-T1DM | Rate | 2021 | 0.245946448 | 0.956016185 | 0.019171723 |
| Incidence | Mauritania | Both | Age-standardized | CKD-T1DM | Rate | 2021 | 0.24581137 | 0.926826994 | 0.02179881 |
| Incidence | Nauru | Both | Age-standardized | CKD-T1DM | Rate | 2021 | 0.244771477 | 1.114686869 | 0.018855364 |
| Incidence | Saint Lucia | Both | Age-standardized | CKD-T1DM | Rate | 2021 | 0.244574319 | 0.945913351 | 0.024620349 |
| Incidence | Pakistan | Both | Age-standardized | CKD-T1DM | Rate | 2021 | 0.242778866 | 0.639249116 | 0.071923952 |
| Incidence | Djibouti | Both | Age-standardized | CKD-T1DM | Rate | 2021 | 0.241474291 | 0.813007317 | 0.029191718 |
| Incidence | Gambia | Both | Age-standardized | CKD-T1DM | Rate | 2021 | 0.239541344 | 1.054778466 | 0.022380065 |
| Incidence | Eritrea | Both | Age-standardized | CKD-T1DM | Rate | 2021 | 0.235065703 | 0.808801135 | 0.027337202 |
| Incidence | Zambia | Both | Age-standardized | CKD-T1DM | Rate | 2021 | 0.226847481 | 0.994817949 | 0.026176048 |
| Incidence | Gabon | Both | Age-standardized | CKD-T1DM | Rate | 2021 | 0.226503048 | 0.89773952 | 0.019704757 |
| Incidence | Benin | Both | Age-standardized | CKD-T1DM | Rate | 2021 | 0.225679202 | 0.935889993 | 0.018835954 |
| Incidence | Somalia | Both | Age-standardized | CKD-T1DM | Rate | 2021 | 0.225336838 | 0.910525682 | 0.023698301 |
| Incidence | Romania | Both | Age-standardized | CKD-T1DM | Rate | 2021 | 0.216961927 | 0.730694424 | 0.02229897 |
| Incidence | Jamaica | Both | Age-standardized | CKD-T1DM | Rate | 2021 | 0.2164093 | 0.797827015 | 0.019701514 |
| Incidence | Northern Mariana Islands | Both | Age-standardized | CKD-T1DM | Rate | 2021 | 0.215367601 | 0.788239825 | 0.018308767 |
| Incidence | Ghana | Both | Age-standardized | CKD-T1DM | Rate | 2021 | 0.21227647 | 0.853346082 | 0.020269402 |
| Incidence | Viet Nam | Both | Age-standardized | CKD-T1DM | Rate | 2021 | 0.211877958 | 0.864909492 | 0.019294946 |
| Incidence | Comoros | Both | Age-standardized | CKD-T1DM | Rate | 2021 | 0.211802435 | 0.807378609 | 0.020453699 |
| Incidence | Senegal | Both | Age-standardized | CKD-T1DM | Rate | 2021 | 0.210780586 | 0.845867919 | 0.020490962 |
| Incidence | Kenya | Both | Age-standardized | CKD-T1DM | Rate | 2021 | 0.210762498 | 0.335260461 | 0.124846701 |
| Incidence | Afghanistan | Both | Age-standardized | CKD-T1DM | Rate | 2021 | 0.208147293 | 0.932382978 | 0.015039296 |
| Incidence | Panama | Both | Age-standardized | CKD-T1DM | Rate | 2021 | 0.207979166 | 0.82409619 | 0.020220709 |
| Incidence | Suriname | Both | Age-standardized | CKD-T1DM | Rate | 2021 | 0.206750701 | 0.869762838 | 0.017521281 |
| Incidence | Thailand | Both | Age-standardized | CKD-T1DM | Rate | 2021 | 0.206348774 | 0.926677283 | 0.019049382 |
| Incidence | Chad | Both | Age-standardized | CKD-T1DM | Rate | 2021 | 0.206340195 | 0.812374123 | 0.018671594 |
| Incidence | Fiji | Both | Age-standardized | CKD-T1DM | Rate | 2021 | 0.205200455 | 0.871977441 | 0.015849066 |
| Incidence | Liberia | Both | Age-standardized | CKD-T1DM | Rate | 2021 | 0.204959725 | 0.773924303 | 0.022270413 |
| Incidence | Trinidad and Tobago | Both | Age-standardized | CKD-T1DM | Rate | 2021 | 0.204477786 | 0.874134807 | 0.021610167 |
| Incidence | Palestine | Both | Age-standardized | CKD-T1DM | Rate | 2021 | 0.203837369 | 0.801635322 | 0.021329872 |
| Incidence | Ecuador | Both | Age-standardized | CKD-T1DM | Rate | 2021 | 0.202380276 | 0.870216012 | 0.017792533 |
| Incidence | Venezuela (Bolivarian Republic of) | Both | Age-standardized | CKD-T1DM | Rate | 2021 | 0.201390756 | 0.811475092 | 0.016941643 |
| Incidence | Samoa | Both | Age-standardized | CKD-T1DM | Rate | 2021 | 0.198403367 | 0.865521333 | 0.014772506 |
| Incidence | Bhutan | Both | Age-standardized | CKD-T1DM | Rate | 2021 | 0.196991028 | 0.762352162 | 0.020433796 |
| Incidence | Tuvalu | Both | Age-standardized | CKD-T1DM | Rate | 2021 | 0.195122916 | 0.817598658 | 0.014391449 |
| Incidence | Haiti | Both | Age-standardized | CKD-T1DM | Rate | 2021 | 0.194151779 | 0.814279965 | 0.019136078 |
| Incidence | Niger | Both | Age-standardized | CKD-T1DM | Rate | 2021 | 0.192371133 | 0.680057269 | 0.017057749 |
| Incidence | Costa Rica | Both | Age-standardized | CKD-T1DM | Rate | 2021 | 0.192289134 | 0.78720445 | 0.015914631 |
| Incidence | American Samoa | Both | Age-standardized | CKD-T1DM | Rate | 2021 | 0.191147113 | 0.775938059 | 0.015346229 |
| Incidence | Dominican Republic | Both | Age-standardized | CKD-T1DM | Rate | 2021 | 0.190334674 | 0.796807347 | 0.015647667 |
| Incidence | South Sudan | Both | Age-standardized | CKD-T1DM | Rate | 2021 | 0.190154749 | 0.744089204 | 0.021661564 |
| Incidence | Timor-Leste | Both | Age-standardized | CKD-T1DM | Rate | 2021 | 0.189665774 | 0.710663812 | 0.017884543 |
| Incidence | Iraq | Both | Age-standardized | CKD-T1DM | Rate | 2021 | 0.188657827 | 0.745470343 | 0.013105082 |
| Incidence | Egypt | Both | Age-standardized | CKD-T1DM | Rate | 2021 | 0.188484119 | 0.696180517 | 0.015934537 |
| Incidence | Sierra Leone | Both | Age-standardized | CKD-T1DM | Rate | 2021 | 0.18727228 | 0.676688253 | 0.016699029 |
| Incidence | Cambodia | Both | Age-standardized | CKD-T1DM | Rate | 2021 | 0.186383905 | 0.771423965 | 0.016582826 |
| Incidence | Madagascar | Both | Age-standardized | CKD-T1DM | Rate | 2021 | 0.185296504 | 0.659430731 | 0.020156459 |
| Incidence | Niue | Both | Age-standardized | CKD-T1DM | Rate | 2021 | 0.185041945 | 0.760190703 | 0.013899169 |
| Incidence | Congo | Both | Age-standardized | CKD-T1DM | Rate | 2021 | 0.184676328 | 0.746844898 | 0.016656142 |
| Incidence | Libya | Both | Age-standardized | CKD-T1DM | Rate | 2021 | 0.184488839 | 0.727307057 | 0.018477669 |
| Incidence | Rwanda | Both | Age-standardized | CKD-T1DM | Rate | 2021 | 0.18389034 | 0.645339976 | 0.022758855 |
| Incidence | Cabo Verde | Both | Age-standardized | CKD-T1DM | Rate | 2021 | 0.182991683 | 0.670605755 | 0.014927371 |
| Incidence | Burkina Faso | Both | Age-standardized | CKD-T1DM | Rate | 2021 | 0.182466428 | 0.748191862 | 0.017520894 |
| Incidence | Nepal | Both | Age-standardized | CKD-T1DM | Rate | 2021 | 0.181266447 | 0.759574396 | 0.014640952 |
| Incidence | Honduras | Both | Age-standardized | CKD-T1DM | Rate | 2021 | 0.178721011 | 0.786487828 | 0.0161514 |
| Incidence | Vanuatu | Both | Age-standardized | CKD-T1DM | Rate | 2021 | 0.176424887 | 0.720676654 | 0.013257987 |
| Incidence | Marshall Islands | Both | Age-standardized | CKD-T1DM | Rate | 2021 | 0.175643617 | 0.792957115 | 0.015106933 |
| Incidence | Bolivia (Plurinational State of) | Both | Age-standardized | CKD-T1DM | Rate | 2021 | 0.173062402 | 0.732612172 | 0.014606244 |
| Incidence | Tokelau | Both | Age-standardized | CKD-T1DM | Rate | 2021 | 0.172768007 | 0.700395955 | 0.013169671 |
| Incidence | Croatia | Both | Age-standardized | CKD-T1DM | Rate | 2021 | 0.17073482 | 0.607720008 | 0.017310752 |
| Incidence | Bermuda | Both | Age-standardized | CKD-T1DM | Rate | 2021 | 0.170022209 | 0.64282921 | 0.013248704 |
| Incidence | Burundi | Both | Age-standardized | CKD-T1DM | Rate | 2021 | 0.166236794 | 0.672910907 | 0.015556895 |
| Incidence | Serbia | Both | Age-standardized | CKD-T1DM | Rate | 2021 | 0.165207507 | 0.559037065 | 0.015260663 |
| Incidence | Sri Lanka | Both | Age-standardized | CKD-T1DM | Rate | 2021 | 0.164650776 | 0.668923471 | 0.016464719 |
| Incidence | Slovenia | Both | Age-standardized | CKD-T1DM | Rate | 2021 | 0.164598915 | 0.613873373 | 0.01677239 |
| Incidence | Seychelles | Both | Age-standardized | CKD-T1DM | Rate | 2021 | 0.164415743 | 0.702414768 | 0.013871355 |
| Incidence | Jordan | Both | Age-standardized | CKD-T1DM | Rate | 2021 | 0.162910374 | 0.751088099 | 0.013531112 |
| Incidence | Uganda | Both | Age-standardized | CKD-T1DM | Rate | 2021 | 0.162324312 | 0.606050713 | 0.017833696 |
| Incidence | Oman | Both | Age-standardized | CKD-T1DM | Rate | 2021 | 0.161583062 | 0.641622029 | 0.014876465 |
| Incidence | Czechia | Both | Age-standardized | CKD-T1DM | Rate | 2021 | 0.160594691 | 0.575031576 | 0.01454919 |
| Incidence | Hungary | Both | Age-standardized | CKD-T1DM | Rate | 2021 | 0.160422509 | 0.544678338 | 0.019998642 |
| Incidence | Morocco | Both | Age-standardized | CKD-T1DM | Rate | 2021 | 0.160106832 | 0.723345996 | 0.012876341 |
| Incidence | Tonga | Both | Age-standardized | CKD-T1DM | Rate | 2021 | 0.159810326 | 0.581247955 | 0.012777886 |
| Incidence | Equatorial Guinea | Both | Age-standardized | CKD-T1DM | Rate | 2021 | 0.159091174 | 0.573573272 | 0.014339191 |
| Incidence | Cook Islands | Both | Age-standardized | CKD-T1DM | Rate | 2021 | 0.157550445 | 0.68008479 | 0.011777547 |
| Incidence | Brunei Darussalam | Both | Age-standardized | CKD-T1DM | Rate | 2021 | 0.156971864 | 0.566322193 | 0.015335846 |
| Incidence | Nigeria | Both | Age-standardized | CKD-T1DM | Rate | 2021 | 0.155437644 | 0.255289327 | 0.08116461 |
| Incidence | Eswatini | Both | Age-standardized | CKD-T1DM | Rate | 2021 | 0.15332387 | 0.633102094 | 0.015080764 |
| Incidence | Mali | Both | Age-standardized | CKD-T1DM | Rate | 2021 | 0.152472278 | 0.614974538 | 0.011189659 |
| Incidence | United Republic of Tanzania | Both | Age-standardized | CKD-T1DM | Rate | 2021 | 0.1510785 | 0.542858055 | 0.01595301 |
| Incidence | Angola | Both | Age-standardized | CKD-T1DM | Rate | 2021 | 0.150852246 | 0.649115721 | 0.013085683 |
| Incidence | Slovakia | Both | Age-standardized | CKD-T1DM | Rate | 2021 | 0.148630615 | 0.534146944 | 0.01401123 |
| Incidence | Ireland | Both | Age-standardized | CKD-T1DM | Rate | 2021 | 0.148554049 | 0.540995776 | 0.017544324 |
| Incidence | Central African Republic | Both | Age-standardized | CKD-T1DM | Rate | 2021 | 0.148162021 | 0.561258275 | 0.014524025 |
| Incidence | Guam | Both | Age-standardized | CKD-T1DM | Rate | 2021 | 0.146845847 | 0.614416348 | 0.011330342 |
| Incidence | Sudan | Both | Age-standardized | CKD-T1DM | Rate | 2021 | 0.145055354 | 0.646495132 | 0.012660787 |
| Incidence | Cuba | Both | Age-standardized | CKD-T1DM | Rate | 2021 | 0.144670734 | 0.676397803 | 0.010713755 |
| Incidence | Malaysia | Both | Age-standardized | CKD-T1DM | Rate | 2021 | 0.143538363 | 0.578092208 | 0.010836771 |
| Incidence | Puerto Rico | Both | Age-standardized | CKD-T1DM | Rate | 2021 | 0.142936189 | 0.61794286 | 0.012822065 |
| Incidence | Peru | Both | Age-standardized | CKD-T1DM | Rate | 2021 | 0.139641404 | 0.562343625 | 0.013294609 |
| Incidence | Democratic Republic of the Congo | Both | Age-standardized | CKD-T1DM | Rate | 2021 | 0.139318115 | 0.547857421 | 0.013054075 |
| Incidence | Ethiopia | Both | Age-standardized | CKD-T1DM | Rate | 2021 | 0.139114114 | 0.320985217 | 0.049914873 |
| Incidence | India | Both | Age-standardized | CKD-T1DM | Rate | 2021 | 0.13835829 | 0.243351345 | 0.073282316 |
| Incidence | T眉rkiye | Both | Age-standardized | CKD-T1DM | Rate | 2021 | 0.137693263 | 0.536694902 | 0.011539522 |
| Incidence | Democratic People's Republic of Korea | Both | Age-standardized | CKD-T1DM | Rate | 2021 | 0.137534052 | 0.616068587 | 0.012971739 |
| Incidence | Kuwait | Both | Age-standardized | CKD-T1DM | Rate | 2021 | 0.136343114 | 0.568773269 | 0.011448332 |
| Incidence | Iran (Islamic Republic of) | Both | Age-standardized | CKD-T1DM | Rate | 2021 | 0.133508108 | 0.275461354 | 0.057141759 |
| Incidence | Algeria | Both | Age-standardized | CKD-T1DM | Rate | 2021 | 0.133366026 | 0.474346514 | 0.011178396 |
| Incidence | Lebanon | Both | Age-standardized | CKD-T1DM | Rate | 2021 | 0.13278562 | 0.5643732 | 0.011559797 |
| Incidence | Bahrain | Both | Age-standardized | CKD-T1DM | Rate | 2021 | 0.129747284 | 0.592454651 | 0.01164228 |
| Incidence | Paraguay | Both | Age-standardized | CKD-T1DM | Rate | 2021 | 0.129435327 | 0.507994508 | 0.010574061 |
| Incidence | Lesotho | Both | Age-standardized | CKD-T1DM | Rate | 2021 | 0.128181254 | 0.561889832 | 0.0141799 |
| Incidence | Maldives | Both | Age-standardized | CKD-T1DM | Rate | 2021 | 0.127045777 | 0.508882402 | 0.008921957 |
| Incidence | Qatar | Both | Age-standardized | CKD-T1DM | Rate | 2021 | 0.124364665 | 0.483545864 | 0.008915168 |
| Incidence | Papua New Guinea | Both | Age-standardized | CKD-T1DM | Rate | 2021 | 0.124025983 | 0.508414788 | 0.010682846 |
| Incidence | Botswana | Both | Age-standardized | CKD-T1DM | Rate | 2021 | 0.118798024 | 0.465001668 | 0.010692314 |
| Incidence | Yemen | Both | Age-standardized | CKD-T1DM | Rate | 2021 | 0.117385493 | 0.516945609 | 0.011866899 |
| Incidence | Colombia | Both | Age-standardized | CKD-T1DM | Rate | 2021 | 0.109392779 | 0.443289385 | 0.00908907 |
| Incidence | Tunisia | Both | Age-standardized | CKD-T1DM | Rate | 2021 | 0.109171245 | 0.4486342 | 0.008928069 |
| Incidence | Brazil | Both | Age-standardized | CKD-T1DM | Rate | 2021 | 0.101260344 | 0.20188558 | 0.036861386 |
| Incidence | Poland | Both | Age-standardized | CKD-T1DM | Rate | 2021 | 0.099677335 | 0.203756383 | 0.030797578 |
| Incidence | Taiwan (Province of China) | Both | Age-standardized | CKD-T1DM | Rate | 2021 | 0.098560424 | 0.361294492 | 0.010163365 |
| Incidence | South Africa | Both | Age-standardized | CKD-T1DM | Rate | 2021 | 0.093111332 | 0.227555622 | 0.031224123 |
| Incidence | Canada | Both | Age-standardized | CKD-T1DM | Rate | 2021 | 0.092432936 | 0.349474181 | 0.007142655 |
| Incidence | Australia | Both | Age-standardized | CKD-T1DM | Rate | 2021 | 0.091491646 | 0.361465363 | 0.007258711 |
| Incidence | Cyprus | Both | Age-standardized | CKD-T1DM | Rate | 2021 | 0.089414666 | 0.35681132 | 0.007734999 |
| Incidence | Namibia | Both | Age-standardized | CKD-T1DM | Rate | 2021 | 0.089353073 | 0.352919134 | 0.008222626 |
| Incidence | Zimbabwe | Both | Age-standardized | CKD-T1DM | Rate | 2021 | 0.088391126 | 0.380117449 | 0.009009669 |
| Incidence | Austria | Both | Age-standardized | CKD-T1DM | Rate | 2021 | 0.081070962 | 0.315729706 | 0.006743015 |
| Incidence | Israel | Both | Age-standardized | CKD-T1DM | Rate | 2021 | 0.08055857 | 0.32039814 | 0.007107676 |
| Incidence | Chile | Both | Age-standardized | CKD-T1DM | Rate | 2021 | 0.078086557 | 0.31342979 | 0.006322456 |
| Incidence | Malta | Both | Age-standardized | CKD-T1DM | Rate | 2021 | 0.07679668 | 0.275037399 | 0.006870373 |
| Incidence | Germany | Both | Age-standardized | CKD-T1DM | Rate | 2021 | 0.076604427 | 0.281815138 | 0.006475596 |
| Incidence | United States of America | Both | Age-standardized | CKD-T1DM | Rate | 2021 | 0.071463506 | 0.148295894 | 0.026324013 |
| Incidence | Bangladesh | Both | Age-standardized | CKD-T1DM | Rate | 2021 | 0.070268459 | 0.270926927 | 0.005560098 |
| Incidence | Greenland | Both | Age-standardized | CKD-T1DM | Rate | 2021 | 0.069136747 | 0.268714188 | 0.006208699 |
| Incidence | Uruguay | Both | Age-standardized | CKD-T1DM | Rate | 2021 | 0.06895593 | 0.275302598 | 0.004682527 |
| Incidence | France | Both | Age-standardized | CKD-T1DM | Rate | 2021 | 0.068511029 | 0.249447199 | 0.006088593 |
| Incidence | Luxembourg | Both | Age-standardized | CKD-T1DM | Rate | 2021 | 0.068229432 | 0.283415641 | 0.004186899 |
| Incidence | New Zealand | Both | Age-standardized | CKD-T1DM | Rate | 2021 | 0.067538532 | 0.223411774 | 0.01015409 |
| Incidence | Finland | Both | Age-standardized | CKD-T1DM | Rate | 2021 | 0.066775266 | 0.272767925 | 0.006836974 |
| Incidence | Singapore | Both | Age-standardized | CKD-T1DM | Rate | 2021 | 0.06642634 | 0.308217907 | 0.004569447 |
| Incidence | Argentina | Both | Age-standardized | CKD-T1DM | Rate | 2021 | 0.066250097 | 0.237900606 | 0.006100894 |
| Incidence | Japan | Both | Age-standardized | CKD-T1DM | Rate | 2021 | 0.063303935 | 0.132827297 | 0.0225778 |
| Incidence | Denmark | Both | Age-standardized | CKD-T1DM | Rate | 2021 | 0.06293004 | 0.251927914 | 0.005267493 |
| Incidence | Greece | Both | Age-standardized | CKD-T1DM | Rate | 2021 | 0.061450268 | 0.256291854 | 0.004186475 |
| Incidence | Andorra | Both | Age-standardized | CKD-T1DM | Rate | 2021 | 0.061175542 | 0.249075816 | 0.004640838 |
| Incidence | Monaco | Both | Age-standardized | CKD-T1DM | Rate | 2021 | 0.059610271 | 0.257842992 | 0.00449265 |
| Incidence | San Marino | Both | Age-standardized | CKD-T1DM | Rate | 2021 | 0.058152914 | 0.251053773 | 0.004832025 |
| Incidence | Italy | Both | Age-standardized | CKD-T1DM | Rate | 2021 | 0.056897083 | 0.137359256 | 0.015098407 |
| Incidence | Switzerland | Both | Age-standardized | CKD-T1DM | Rate | 2021 | 0.056808677 | 0.223836733 | 0.004318366 |
| Incidence | Belgium | Both | Age-standardized | CKD-T1DM | Rate | 2021 | 0.056033312 | 0.233792209 | 0.004982618 |
| Incidence | China | Both | Age-standardized | CKD-T1DM | Rate | 2021 | 0.052700836 | 0.1077156 | 0.020141852 |
| Incidence | Iceland | Both | Age-standardized | CKD-T1DM | Rate | 2021 | 0.051951326 | 0.22822917 | 0.003551866 |
| Incidence | Republic of Korea | Both | Age-standardized | CKD-T1DM | Rate | 2021 | 0.051604591 | 0.212180169 | 0.003854856 |
| Incidence | Netherlands | Both | Age-standardized | CKD-T1DM | Rate | 2021 | 0.047791817 | 0.18307018 | 0.00367359 |
| Incidence | United Kingdom | Both | Age-standardized | CKD-T1DM | Rate | 2021 | 0.046622784 | 0.097689443 | 0.015427855 |
| Incidence | Norway | Both | Age-standardized | CKD-T1DM | Rate | 2021 | 0.045088295 | 0.11016092 | 0.011303206 |
| Incidence | Portugal | Both | Age-standardized | CKD-T1DM | Rate | 2021 | 0.043444831 | 0.180725859 | 0.003355864 |
| Incidence | Spain | Both | Age-standardized | CKD-T1DM | Rate | 2021 | 0.039344187 | 0.136903899 | 0.00306427 |
| Incidence | Sweden | Both | Age-standardized | CKD-T1DM | Rate | 2021 | 0.035465216 | 0.121410533 | 0.004189833 |

Appendix

**Appendix 16：Global disease burden distribution of Chronic kidney disease due to diabetes mellitus type 1 in children and adolescents from 1990 to 2021**

| **measure** | **location** | **sex** | **age** | **cause** | **metric** | **year** | **val** | **upper** | **lower** |
| --- | --- | --- | --- | --- | --- | --- | --- | --- | --- |
| Prevalence | Canada | Both | Age-standardized | CKD-T1DM | Rate | 2021 | 27.46261532 | 62.66584966 | 9.237368953 |
| Prevalence | Lithuania | Both | Age-standardized | CKD-T1DM | Rate | 2021 | 25.4125771 | 60.44128021 | 8.212419277 |
| Prevalence | Belarus | Both | Age-standardized | CKD-T1DM | Rate | 2021 | 24.64587758 | 62.40389748 | 7.201250446 |
| Prevalence | Estonia | Both | Age-standardized | CKD-T1DM | Rate | 2021 | 24.6051512 | 58.59155684 | 7.237655919 |
| Prevalence | Russian Federation | Both | Age-standardized | CKD-T1DM | Rate | 2021 | 24.22914005 | 31.87442881 | 17.6991888 |
| Prevalence | Latvia | Both | Age-standardized | CKD-T1DM | Rate | 2021 | 23.93876008 | 58.23847553 | 7.435518249 |
| Prevalence | Republic of Moldova | Both | Age-standardized | CKD-T1DM | Rate | 2021 | 23.59762504 | 55.72635526 | 7.08357065 |
| Prevalence | Indonesia | Both | Age-standardized | CKD-T1DM | Rate | 2021 | 21.41867117 | 33.66511708 | 12.84355991 |
| Prevalence | Ukraine | Both | Age-standardized | CKD-T1DM | Rate | 2021 | 21.31770007 | 48.78017335 | 6.700390338 |
| Prevalence | Montenegro | Both | Age-standardized | CKD-T1DM | Rate | 2021 | 21.31571461 | 49.44223667 | 6.699423267 |
| Prevalence | North Macedonia | Both | Age-standardized | CKD-T1DM | Rate | 2021 | 21.30735891 | 46.10129235 | 6.860101813 |
| Prevalence | Ireland | Both | Age-standardized | CKD-T1DM | Rate | 2021 | 21.17557767 | 48.27956544 | 7.23950449 |
| Prevalence | Netherlands | Both | Age-standardized | CKD-T1DM | Rate | 2021 | 20.81087754 | 49.35200072 | 5.675032906 |
| Prevalence | Serbia | Both | Age-standardized | CKD-T1DM | Rate | 2021 | 20.44567648 | 48.6878205 | 5.84614761 |
| Prevalence | Albania | Both | Age-standardized | CKD-T1DM | Rate | 2021 | 20.3807678 | 44.73537954 | 6.968202842 |
| Prevalence | Bosnia and Herzegovina | Both | Age-standardized | CKD-T1DM | Rate | 2021 | 20.07156827 | 46.73278981 | 6.144072969 |
| Prevalence | Uzbekistan | Both | Age-standardized | CKD-T1DM | Rate | 2021 | 19.77483672 | 45.98446057 | 6.597036089 |
| Prevalence | Mauritius | Both | Age-standardized | CKD-T1DM | Rate | 2021 | 19.7199376 | 58.04889329 | 5.472346357 |
| Prevalence | Hungary | Both | Age-standardized | CKD-T1DM | Rate | 2021 | 19.68912664 | 44.4478125 | 6.606125887 |
| Prevalence | Bulgaria | Both | Age-standardized | CKD-T1DM | Rate | 2021 | 19.53343285 | 44.26930305 | 6.237730397 |
| Prevalence | Poland | Both | Age-standardized | CKD-T1DM | Rate | 2021 | 19.36071862 | 26.97772208 | 12.71327324 |
| Prevalence | Brunei Darussalam | Both | Age-standardized | CKD-T1DM | Rate | 2021 | 19.31465617 | 45.94273071 | 5.32757445 |
| Prevalence | Finland | Both | Age-standardized | CKD-T1DM | Rate | 2021 | 19.22167879 | 45.43706098 | 5.319473188 |
| Prevalence | Croatia | Both | Age-standardized | CKD-T1DM | Rate | 2021 | 19.03306657 | 43.19708132 | 5.875043186 |
| Prevalence | Oman | Both | Age-standardized | CKD-T1DM | Rate | 2021 | 18.87092624 | 47.60290161 | 5.565747423 |
| Prevalence | Australia | Both | Age-standardized | CKD-T1DM | Rate | 2021 | 18.74556091 | 44.44427597 | 5.931142211 |
| Prevalence | Sweden | Both | Age-standardized | CKD-T1DM | Rate | 2021 | 18.44978374 | 44.74174829 | 6.428562741 |
| Prevalence | Slovenia | Both | Age-standardized | CKD-T1DM | Rate | 2021 | 18.41492603 | 43.93020231 | 5.748156817 |
| Prevalence | Argentina | Both | Age-standardized | CKD-T1DM | Rate | 2021 | 18.26476393 | 43.41398026 | 5.063091782 |
| Prevalence | Slovakia | Both | Age-standardized | CKD-T1DM | Rate | 2021 | 18.24978475 | 43.84965938 | 4.891792432 |
| Prevalence | Austria | Both | Age-standardized | CKD-T1DM | Rate | 2021 | 18.20502492 | 45.04710648 | 4.836763173 |
| Prevalence | Italy | Both | Age-standardized | CKD-T1DM | Rate | 2021 | 18.19472514 | 26.29113684 | 12.0427299 |
| Prevalence | Cyprus | Both | Age-standardized | CKD-T1DM | Rate | 2021 | 18.07152224 | 42.36832762 | 4.829689078 |
| Prevalence | Azerbaijan | Both | Age-standardized | CKD-T1DM | Rate | 2021 | 17.88829473 | 45.23847309 | 5.169633728 |
| Prevalence | Uruguay | Both | Age-standardized | CKD-T1DM | Rate | 2021 | 17.82979126 | 44.47906624 | 4.919427154 |
| Prevalence | Philippines | Both | Age-standardized | CKD-T1DM | Rate | 2021 | 17.80748914 | 24.12824197 | 12.60956698 |
| Prevalence | Czechia | Both | Age-standardized | CKD-T1DM | Rate | 2021 | 17.78946589 | 40.64842993 | 5.469051128 |
| Prevalence | Greenland | Both | Age-standardized | CKD-T1DM | Rate | 2021 | 17.67485074 | 46.17150762 | 4.531008534 |
| Prevalence | Malta | Both | Age-standardized | CKD-T1DM | Rate | 2021 | 17.45483111 | 40.78504063 | 5.050218212 |
| Prevalence | Chile | Both | Age-standardized | CKD-T1DM | Rate | 2021 | 17.06810084 | 41.70946978 | 5.090589068 |
| Prevalence | United Arab Emirates | Both | Age-standardized | CKD-T1DM | Rate | 2021 | 16.5292999 | 40.97405214 | 4.224410281 |
| Prevalence | Romania | Both | Age-standardized | CKD-T1DM | Rate | 2021 | 16.50115775 | 40.31989504 | 5.050561447 |
| Prevalence | Denmark | Both | Age-standardized | CKD-T1DM | Rate | 2021 | 16.50108417 | 39.18008216 | 5.229365965 |
| Prevalence | Egypt | Both | Age-standardized | CKD-T1DM | Rate | 2021 | 16.43113649 | 41.50590244 | 4.32378888 |
| Prevalence | Myanmar | Both | Age-standardized | CKD-T1DM | Rate | 2021 | 16.34018721 | 42.84990564 | 4.52530552 |
| Prevalence | Viet Nam | Both | Age-standardized | CKD-T1DM | Rate | 2021 | 16.27143274 | 47.23326197 | 4.076664414 |
| Prevalence | Armenia | Both | Age-standardized | CKD-T1DM | Rate | 2021 | 16.26180106 | 38.57849381 | 4.921828397 |
| Prevalence | Israel | Both | Age-standardized | CKD-T1DM | Rate | 2021 | 16.23982098 | 39.32999418 | 4.902425025 |
| Prevalence | Turkmenistan | Both | Age-standardized | CKD-T1DM | Rate | 2021 | 16.18283305 | 39.66626672 | 4.277926273 |
| Prevalence | T眉rkiye | Both | Age-standardized | CKD-T1DM | Rate | 2021 | 15.98518789 | 43.90689609 | 4.343887691 |
| Prevalence | Norway | Both | Age-standardized | CKD-T1DM | Rate | 2021 | 15.55534699 | 24.15980589 | 9.797727788 |
| Prevalence | Lao People's Democratic Republic | Both | Age-standardized | CKD-T1DM | Rate | 2021 | 15.32005776 | 40.53416627 | 3.917362603 |
| Prevalence | Saudi Arabia | Both | Age-standardized | CKD-T1DM | Rate | 2021 | 15.11324502 | 39.30973034 | 3.789916643 |
| Prevalence | Cambodia | Both | Age-standardized | CKD-T1DM | Rate | 2021 | 14.9193535 | 40.57872404 | 3.931672552 |
| Prevalence | Georgia | Both | Age-standardized | CKD-T1DM | Rate | 2021 | 14.69585026 | 34.52092168 | 4.266992372 |
| Prevalence | Palestine | Both | Age-standardized | CKD-T1DM | Rate | 2021 | 14.69210939 | 38.20966674 | 4.513109558 |
| Prevalence | Syrian Arab Republic | Both | Age-standardized | CKD-T1DM | Rate | 2021 | 14.45175065 | 39.32390752 | 3.889737394 |
| Prevalence | Tajikistan | Both | Age-standardized | CKD-T1DM | Rate | 2021 | 14.42494538 | 33.35227409 | 4.086683439 |
| Prevalence | Luxembourg | Both | Age-standardized | CKD-T1DM | Rate | 2021 | 13.93795458 | 35.15500719 | 3.78108296 |
| Prevalence | Maldives | Both | Age-standardized | CKD-T1DM | Rate | 2021 | 13.61734253 | 34.48839364 | 3.540228445 |
| Prevalence | France | Both | Age-standardized | CKD-T1DM | Rate | 2021 | 13.57494446 | 32.69640334 | 3.856994047 |
| Prevalence | Pakistan | Both | Age-standardized | CKD-T1DM | Rate | 2021 | 13.46422201 | 25.48764779 | 6.825867141 |
| Prevalence | Timor-Leste | Both | Age-standardized | CKD-T1DM | Rate | 2021 | 13.27741499 | 37.85359523 | 2.872744888 |
| Prevalence | Sri Lanka | Both | Age-standardized | CKD-T1DM | Rate | 2021 | 13.2028969 | 36.58852004 | 3.274061866 |
| Prevalence | India | Both | Age-standardized | CKD-T1DM | Rate | 2021 | 13.18142546 | 19.4122704 | 8.758124936 |
| Prevalence | Andorra | Both | Age-standardized | CKD-T1DM | Rate | 2021 | 13.17478137 | 33.27813758 | 3.630122221 |
| Prevalence | United Republic of Tanzania | Both | Age-standardized | CKD-T1DM | Rate | 2021 | 13.09304944 | 33.2603918 | 3.373842921 |
| Prevalence | Malawi | Both | Age-standardized | CKD-T1DM | Rate | 2021 | 13.07311367 | 31.77980844 | 3.933468425 |
| Prevalence | Thailand | Both | Age-standardized | CKD-T1DM | Rate | 2021 | 13.06079851 | 35.96682607 | 3.531353604 |
| Prevalence | Monaco | Both | Age-standardized | CKD-T1DM | Rate | 2021 | 13.04889698 | 34.92983945 | 3.173486456 |
| Prevalence | Brazil | Both | Age-standardized | CKD-T1DM | Rate | 2021 | 13.03325363 | 19.93323418 | 8.311601254 |
| Prevalence | Malaysia | Both | Age-standardized | CKD-T1DM | Rate | 2021 | 12.96612707 | 35.26213857 | 2.942364786 |
| Prevalence | San Marino | Both | Age-standardized | CKD-T1DM | Rate | 2021 | 12.91475242 | 33.2020685 | 3.334708871 |
| Prevalence | Iran (Islamic Republic of) | Both | Age-standardized | CKD-T1DM | Rate | 2021 | 12.89108053 | 18.14846693 | 8.892715682 |
| Prevalence | Germany | Both | Age-standardized | CKD-T1DM | Rate | 2021 | 12.8586329 | 30.89447787 | 3.669565903 |
| Prevalence | Bahrain | Both | Age-standardized | CKD-T1DM | Rate | 2021 | 12.58631799 | 32.84030168 | 3.489370672 |
| Prevalence | Spain | Both | Age-standardized | CKD-T1DM | Rate | 2021 | 12.55303761 | 31.50870921 | 3.932405268 |
| Prevalence | Bangladesh | Both | Age-standardized | CKD-T1DM | Rate | 2021 | 12.45668432 | 33.92504007 | 3.456011963 |
| Prevalence | Qatar | Both | Age-standardized | CKD-T1DM | Rate | 2021 | 12.43189638 | 34.4881278 | 3.404475933 |
| Prevalence | Bhutan | Both | Age-standardized | CKD-T1DM | Rate | 2021 | 12.25598451 | 33.21016732 | 3.343564482 |
| Prevalence | Kyrgyzstan | Both | Age-standardized | CKD-T1DM | Rate | 2021 | 12.24757761 | 31.47003384 | 3.346559712 |
| Prevalence | Belgium | Both | Age-standardized | CKD-T1DM | Rate | 2021 | 12.1306398 | 33.11599821 | 3.286659813 |
| Prevalence | Singapore | Both | Age-standardized | CKD-T1DM | Rate | 2021 | 12.04492658 | 32.03159749 | 2.969376602 |
| Prevalence | Rwanda | Both | Age-standardized | CKD-T1DM | Rate | 2021 | 11.80137181 | 30.93567601 | 3.188173555 |
| Prevalence | United States of America | Both | Age-standardized | CKD-T1DM | Rate | 2021 | 11.79188298 | 15.61334277 | 8.497112146 |
| Prevalence | Yemen | Both | Age-standardized | CKD-T1DM | Rate | 2021 | 11.61277292 | 32.33597137 | 2.985402749 |
| Prevalence | Jordan | Both | Age-standardized | CKD-T1DM | Rate | 2021 | 11.45416102 | 32.22405261 | 2.861374739 |
| Prevalence | Sudan | Both | Age-standardized | CKD-T1DM | Rate | 2021 | 11.44607689 | 30.09126371 | 2.962599186 |
| Prevalence | Switzerland | Both | Age-standardized | CKD-T1DM | Rate | 2021 | 11.23587665 | 29.54106929 | 3.016456792 |
| Prevalence | Seychelles | Both | Age-standardized | CKD-T1DM | Rate | 2021 | 11.22415702 | 30.64283051 | 2.75875047 |
| Prevalence | Kazakhstan | Both | Age-standardized | CKD-T1DM | Rate | 2021 | 11.19788278 | 29.7505702 | 3.24987707 |
| Prevalence | Kenya | Both | Age-standardized | CKD-T1DM | Rate | 2021 | 11.18440429 | 15.07474242 | 7.912124478 |
| Prevalence | Libya | Both | Age-standardized | CKD-T1DM | Rate | 2021 | 11.0823109 | 32.55912195 | 2.811713156 |
| Prevalence | Mozambique | Both | Age-standardized | CKD-T1DM | Rate | 2021 | 11.06346698 | 25.26200627 | 3.526185724 |
| Prevalence | Mongolia | Both | Age-standardized | CKD-T1DM | Rate | 2021 | 10.99272007 | 28.00049521 | 3.154634317 |
| Prevalence | Kuwait | Both | Age-standardized | CKD-T1DM | Rate | 2021 | 10.72317426 | 30.28659187 | 2.609245994 |
| Prevalence | Algeria | Both | Age-standardized | CKD-T1DM | Rate | 2021 | 10.63477993 | 28.73007942 | 2.690831637 |
| Prevalence | Tunisia | Both | Age-standardized | CKD-T1DM | Rate | 2021 | 10.63092393 | 31.14859092 | 2.770532822 |
| Prevalence | Uganda | Both | Age-standardized | CKD-T1DM | Rate | 2021 | 10.50295482 | 28.00362118 | 2.630540485 |
| Prevalence | Japan | Both | Age-standardized | CKD-T1DM | Rate | 2021 | 10.35663302 | 14.76004748 | 7.006847906 |
| Prevalence | Djibouti | Both | Age-standardized | CKD-T1DM | Rate | 2021 | 10.28393873 | 25.44745069 | 2.848209779 |
| Prevalence | Lebanon | Both | Age-standardized | CKD-T1DM | Rate | 2021 | 10.27563104 | 27.21091297 | 2.497885222 |
| Prevalence | Portugal | Both | Age-standardized | CKD-T1DM | Rate | 2021 | 10.27157225 | 26.24678576 | 2.770182504 |
| Prevalence | Paraguay | Both | Age-standardized | CKD-T1DM | Rate | 2021 | 10.25913487 | 29.45878876 | 2.928986983 |
| Prevalence | Barbados | Both | Age-standardized | CKD-T1DM | Rate | 2021 | 10.06470252 | 25.04100646 | 2.812278745 |
| Prevalence | Greece | Both | Age-standardized | CKD-T1DM | Rate | 2021 | 9.991978707 | 24.10504275 | 2.663046023 |
| Prevalence | Burundi | Both | Age-standardized | CKD-T1DM | Rate | 2021 | 9.967139879 | 27.24957554 | 2.335269195 |
| Prevalence | Belize | Both | Age-standardized | CKD-T1DM | Rate | 2021 | 9.951802744 | 25.16699452 | 2.695335092 |
| Prevalence | Afghanistan | Both | Age-standardized | CKD-T1DM | Rate | 2021 | 9.9061429 | 27.72467071 | 2.454217632 |
| Prevalence | Iceland | Both | Age-standardized | CKD-T1DM | Rate | 2021 | 9.861394759 | 26.78863442 | 2.671985747 |
| Prevalence | New Zealand | Both | Age-standardized | CKD-T1DM | Rate | 2021 | 9.848509759 | 22.14863387 | 3.747382481 |
| Prevalence | Morocco | Both | Age-standardized | CKD-T1DM | Rate | 2021 | 9.793940622 | 26.91458252 | 2.337541111 |
| Prevalence | Guatemala | Both | Age-standardized | CKD-T1DM | Rate | 2021 | 9.79054051 | 25.15260089 | 2.994983589 |
| Prevalence | Saint Kitts and Nevis | Both | Age-standardized | CKD-T1DM | Rate | 2021 | 9.78059512 | 24.58618291 | 2.653679497 |
| Prevalence | Fiji | Both | Age-standardized | CKD-T1DM | Rate | 2021 | 9.714747441 | 26.67818739 | 2.796357057 |
| Prevalence | Antigua and Barbuda | Both | Age-standardized | CKD-T1DM | Rate | 2021 | 9.671687354 | 25.02892588 | 2.518792125 |
| Prevalence | Haiti | Both | Age-standardized | CKD-T1DM | Rate | 2021 | 9.636505733 | 26.8872368 | 2.388069179 |
| Prevalence | South Sudan | Both | Age-standardized | CKD-T1DM | Rate | 2021 | 9.631306797 | 23.99488129 | 2.755401582 |
| Prevalence | Iraq | Both | Age-standardized | CKD-T1DM | Rate | 2021 | 9.448621104 | 24.77189026 | 2.548070171 |
| Prevalence | Somalia | Both | Age-standardized | CKD-T1DM | Rate | 2021 | 9.445387509 | 24.73767991 | 2.717826271 |
| Prevalence | Eswatini | Both | Age-standardized | CKD-T1DM | Rate | 2021 | 9.430277949 | 24.87273393 | 2.170478383 |
| Prevalence | Grenada | Both | Age-standardized | CKD-T1DM | Rate | 2021 | 9.410890552 | 25.81204282 | 2.564927861 |
| Prevalence | Puerto Rico | Both | Age-standardized | CKD-T1DM | Rate | 2021 | 9.32350498 | 21.68791751 | 2.449960614 |
| Prevalence | Lesotho | Both | Age-standardized | CKD-T1DM | Rate | 2021 | 9.224316135 | 23.14858601 | 2.433939434 |
| Prevalence | Saint Vincent and the Grenadines | Both | Age-standardized | CKD-T1DM | Rate | 2021 | 9.106277435 | 23.05196312 | 2.290305789 |
| Prevalence | Dominica | Both | Age-standardized | CKD-T1DM | Rate | 2021 | 9.096712728 | 24.20427703 | 2.282232619 |
| Prevalence | Guyana | Both | Age-standardized | CKD-T1DM | Rate | 2021 | 8.928242369 | 23.93792776 | 2.311837921 |
| Prevalence | Solomon Islands | Both | Age-standardized | CKD-T1DM | Rate | 2021 | 8.859421455 | 23.64525808 | 2.444292678 |
| Prevalence | Madagascar | Both | Age-standardized | CKD-T1DM | Rate | 2021 | 8.838718842 | 23.32401922 | 2.318058298 |
| Prevalence | Botswana | Both | Age-standardized | CKD-T1DM | Rate | 2021 | 8.636273922 | 23.99012415 | 2.315892545 |
| Prevalence | Ethiopia | Both | Age-standardized | CKD-T1DM | Rate | 2021 | 8.523004916 | 15.21332692 | 4.378903287 |
| Prevalence | Eritrea | Both | Age-standardized | CKD-T1DM | Rate | 2021 | 8.409603832 | 21.63577778 | 2.324926238 |
| Prevalence | Jamaica | Both | Age-standardized | CKD-T1DM | Rate | 2021 | 8.297383641 | 19.32293698 | 2.347488145 |
| Prevalence | Trinidad and Tobago | Both | Age-standardized | CKD-T1DM | Rate | 2021 | 8.241411064 | 21.0014712 | 2.484638917 |
| Prevalence | Bahamas | Both | Age-standardized | CKD-T1DM | Rate | 2021 | 8.191258331 | 19.53103521 | 2.325929819 |
| Prevalence | Nepal | Both | Age-standardized | CKD-T1DM | Rate | 2021 | 8.160858686 | 22.32479324 | 2.015764459 |
| Prevalence | Namibia | Both | Age-standardized | CKD-T1DM | Rate | 2021 | 8.123038976 | 22.20929857 | 2.220749074 |
| Prevalence | Saint Lucia | Both | Age-standardized | CKD-T1DM | Rate | 2021 | 8.025741009 | 21.028743 | 2.218411531 |
| Prevalence | Dominican Republic | Both | Age-standardized | CKD-T1DM | Rate | 2021 | 7.893745188 | 19.05518867 | 2.397664291 |
| Prevalence | South Africa | Both | Age-standardized | CKD-T1DM | Rate | 2021 | 7.838537802 | 12.7594651 | 4.645599861 |
| Prevalence | Comoros | Both | Age-standardized | CKD-T1DM | Rate | 2021 | 7.726428054 | 20.43223489 | 1.926049145 |
| Prevalence | Mexico | Both | Age-standardized | CKD-T1DM | Rate | 2021 | 7.644961437 | 10.6447158 | 5.513891669 |
| Prevalence | Vanuatu | Both | Age-standardized | CKD-T1DM | Rate | 2021 | 7.529467045 | 18.56025234 | 1.845860749 |
| Prevalence | Bolivia (Plurinational State of) | Both | Age-standardized | CKD-T1DM | Rate | 2021 | 7.445079781 | 18.97956917 | 2.023195678 |
| Prevalence | United States Virgin Islands | Both | Age-standardized | CKD-T1DM | Rate | 2021 | 7.383680807 | 19.27981332 | 2.062024556 |
| Prevalence | Republic of Korea | Both | Age-standardized | CKD-T1DM | Rate | 2021 | 7.368602832 | 20.11617807 | 1.900291294 |
| Prevalence | Nigeria | Both | Age-standardized | CKD-T1DM | Rate | 2021 | 7.333533353 | 10.23030713 | 5.21204168 |
| Prevalence | Kiribati | Both | Age-standardized | CKD-T1DM | Rate | 2021 | 7.324204586 | 20.16729597 | 1.643071409 |
| Prevalence | Micronesia (Federated States of) | Both | Age-standardized | CKD-T1DM | Rate | 2021 | 7.293973927 | 19.70633835 | 1.887423028 |
| Prevalence | Nauru | Both | Age-standardized | CKD-T1DM | Rate | 2021 | 7.283256477 | 18.6954215 | 1.829036352 |
| Prevalence | Nicaragua | Both | Age-standardized | CKD-T1DM | Rate | 2021 | 7.258424991 | 18.74454405 | 1.872542696 |
| Prevalence | Taiwan (Province of China) | Both | Age-standardized | CKD-T1DM | Rate | 2021 | 7.241566346 | 19.50334513 | 1.981276589 |
| Prevalence | Zambia | Both | Age-standardized | CKD-T1DM | Rate | 2021 | 7.169844497 | 17.8543696 | 1.83154334 |
| Prevalence | Tuvalu | Both | Age-standardized | CKD-T1DM | Rate | 2021 | 7.161442933 | 17.74307169 | 1.780700241 |
| Prevalence | Zimbabwe | Both | Age-standardized | CKD-T1DM | Rate | 2021 | 7.127782404 | 18.06942389 | 1.856256339 |
| Prevalence | Congo | Both | Age-standardized | CKD-T1DM | Rate | 2021 | 7.1273402 | 19.35871086 | 1.968745476 |
| Prevalence | El Salvador | Both | Age-standardized | CKD-T1DM | Rate | 2021 | 7.054839747 | 18.68354499 | 1.735120318 |
| Prevalence | Bermuda | Both | Age-standardized | CKD-T1DM | Rate | 2021 | 7.038506792 | 18.16210399 | 2.169810576 |
| Prevalence | Tonga | Both | Age-standardized | CKD-T1DM | Rate | 2021 | 7.021147695 | 19.6405556 | 1.721230898 |
| Prevalence | Northern Mariana Islands | Both | Age-standardized | CKD-T1DM | Rate | 2021 | 7.001161733 | 17.20679291 | 1.57638094 |
| Prevalence | Gabon | Both | Age-standardized | CKD-T1DM | Rate | 2021 | 6.934708472 | 16.55291628 | 1.866286162 |
| Prevalence | Palau | Both | Age-standardized | CKD-T1DM | Rate | 2021 | 6.901419237 | 19.75865213 | 1.859904221 |
| Prevalence | Panama | Both | Age-standardized | CKD-T1DM | Rate | 2021 | 6.80506525 | 18.58458525 | 1.687759352 |
| Prevalence | Suriname | Both | Age-standardized | CKD-T1DM | Rate | 2021 | 6.733666387 | 18.337354 | 1.972380592 |
| Prevalence | Mauritania | Both | Age-standardized | CKD-T1DM | Rate | 2021 | 6.693207183 | 17.81315831 | 1.728122382 |
| Prevalence | United Kingdom | Both | Age-standardized | CKD-T1DM | Rate | 2021 | 6.641919848 | 9.021595747 | 4.766030467 |
| Prevalence | Cameroon | Both | Age-standardized | CKD-T1DM | Rate | 2021 | 6.498122127 | 16.44611427 | 1.48326732 |
| Prevalence | Guam | Both | Age-standardized | CKD-T1DM | Rate | 2021 | 6.490434809 | 17.54755332 | 1.44087384 |
| Prevalence | Togo | Both | Age-standardized | CKD-T1DM | Rate | 2021 | 6.483741609 | 17.29073539 | 1.712494245 |
| Prevalence | Papua New Guinea | Both | Age-standardized | CKD-T1DM | Rate | 2021 | 6.463915308 | 16.77611844 | 1.485468017 |
| Prevalence | American Samoa | Both | Age-standardized | CKD-T1DM | Rate | 2021 | 6.42526131 | 16.1956551 | 1.65222789 |
| Prevalence | Democratic Republic of the Congo | Both | Age-standardized | CKD-T1DM | Rate | 2021 | 6.423671987 | 15.87813186 | 1.47296167 |
| Prevalence | Peru | Both | Age-standardized | CKD-T1DM | Rate | 2021 | 6.409321529 | 16.55154202 | 1.778153681 |
| Prevalence | Ecuador | Both | Age-standardized | CKD-T1DM | Rate | 2021 | 6.400603074 | 16.18420395 | 1.664965773 |
| Prevalence | Guinea | Both | Age-standardized | CKD-T1DM | Rate | 2021 | 6.170023855 | 15.35470986 | 1.729595528 |
| Prevalence | Samoa | Both | Age-standardized | CKD-T1DM | Rate | 2021 | 6.166769831 | 16.65850706 | 1.448931267 |
| Prevalence | Angola | Both | Age-standardized | CKD-T1DM | Rate | 2021 | 6.145887638 | 16.60428031 | 1.599804146 |
| Prevalence | Central African Republic | Both | Age-standardized | CKD-T1DM | Rate | 2021 | 6.074827754 | 15.62822685 | 1.561945505 |
| Prevalence | Niue | Both | Age-standardized | CKD-T1DM | Rate | 2021 | 6.064422185 | 15.51839953 | 1.6946064 |
| Prevalence | Guinea-Bissau | Both | Age-standardized | CKD-T1DM | Rate | 2021 | 6.061300044 | 15.95769097 | 1.525065189 |
| Prevalence | Equatorial Guinea | Both | Age-standardized | CKD-T1DM | Rate | 2021 | 6.039920238 | 16.50699784 | 1.757167983 |
| Prevalence | Burkina Faso | Both | Age-standardized | CKD-T1DM | Rate | 2021 | 5.940485211 | 16.72847825 | 1.572321773 |
| Prevalence | Marshall Islands | Both | Age-standardized | CKD-T1DM | Rate | 2021 | 5.918613566 | 15.0735889 | 1.455695869 |
| Prevalence | Tokelau | Both | Age-standardized | CKD-T1DM | Rate | 2021 | 5.900707252 | 15.87492682 | 1.435990631 |
| Prevalence | Gambia | Both | Age-standardized | CKD-T1DM | Rate | 2021 | 5.898364827 | 16.60958761 | 1.489590465 |
| Prevalence | Sao Tome and Principe | Both | Age-standardized | CKD-T1DM | Rate | 2021 | 5.862619213 | 16.66335606 | 1.494738287 |
| Prevalence | C么te d'Ivoire | Both | Age-standardized | CKD-T1DM | Rate | 2021 | 5.774698712 | 15.52169037 | 1.520691292 |
| Prevalence | Sierra Leone | Both | Age-standardized | CKD-T1DM | Rate | 2021 | 5.714561972 | 15.12048522 | 1.446154696 |
| Prevalence | Chad | Both | Age-standardized | CKD-T1DM | Rate | 2021 | 5.494175657 | 15.09770877 | 1.471755502 |
| Prevalence | Benin | Both | Age-standardized | CKD-T1DM | Rate | 2021 | 5.451146436 | 15.55971411 | 1.276808297 |
| Prevalence | Venezuela (Bolivarian Republic of) | Both | Age-standardized | CKD-T1DM | Rate | 2021 | 5.430149433 | 13.02859862 | 1.316249169 |
| Prevalence | Cuba | Both | Age-standardized | CKD-T1DM | Rate | 2021 | 5.425160202 | 14.48308561 | 1.454221304 |
| Prevalence | Senegal | Both | Age-standardized | CKD-T1DM | Rate | 2021 | 5.406582161 | 14.65159143 | 1.262856225 |
| Prevalence | Cook Islands | Both | Age-standardized | CKD-T1DM | Rate | 2021 | 5.374815311 | 13.75757277 | 1.383932717 |
| Prevalence | Liberia | Both | Age-standardized | CKD-T1DM | Rate | 2021 | 5.324005789 | 14.24584332 | 1.342492828 |
| Prevalence | Honduras | Both | Age-standardized | CKD-T1DM | Rate | 2021 | 5.207868451 | 13.03683416 | 1.40560397 |
| Prevalence | Democratic People's Republic of Korea | Both | Age-standardized | CKD-T1DM | Rate | 2021 | 5.173590388 | 14.40281075 | 1.244224075 |
| Prevalence | Cabo Verde | Both | Age-standardized | CKD-T1DM | Rate | 2021 | 5.119371974 | 14.59851906 | 1.294121522 |
| Prevalence | Costa Rica | Both | Age-standardized | CKD-T1DM | Rate | 2021 | 5.070490525 | 14.34774741 | 1.199417808 |
| Prevalence | Ghana | Both | Age-standardized | CKD-T1DM | Rate | 2021 | 5.059394777 | 13.82399745 | 1.325691579 |
| Prevalence | Colombia | Both | Age-standardized | CKD-T1DM | Rate | 2021 | 5.00614669 | 12.80066859 | 1.280789048 |
| Prevalence | Niger | Both | Age-standardized | CKD-T1DM | Rate | 2021 | 4.780476893 | 12.7668673 | 1.138037705 |
| Prevalence | Mali | Both | Age-standardized | CKD-T1DM | Rate | 2021 | 4.758696342 | 11.90229434 | 0.993179742 |
| Prevalence | China | Both | Age-standardized | CKD-T1DM | Rate | 2021 | 4.176490524 | 6.164315475 | 2.851007294 |

Appendix

**Appendix 17：Global disease burden distribution of Chronic kidney disease due to diabetes mellitus type 1 in children and adolescents from 1990 to 2021**

| **measure** | **location** | **sex** | **age** | **cause** | **metric** | **year** | **val** | **upper** | **lower** |
| --- | --- | --- | --- | --- | --- | --- | --- | --- | --- |
| Deaths | Niue | Both | Age-standardized | CKD-T1DM | Rate | 2021 | 0.1697428 | 0.325063548 | 0.072675354 |
| Deaths | American Samoa | Both | Age-standardized | CKD-T1DM | Rate | 2021 | 0.124542047 | 0.198663833 | 0.065276011 |
| Deaths | Tokelau | Both | Age-standardized | CKD-T1DM | Rate | 2021 | 0.122115733 | 0.214686905 | 0.06266471 |
| Deaths | Palau | Both | Age-standardized | CKD-T1DM | Rate | 2021 | 0.115729298 | 0.204561474 | 0.051537516 |
| Deaths | Mauritius | Both | Age-standardized | CKD-T1DM | Rate | 2021 | 0.087521001 | 0.133808841 | 0.052139746 |
| Deaths | Nauru | Both | Age-standardized | CKD-T1DM | Rate | 2021 | 0.082685964 | 0.138690079 | 0.038911596 |
| Deaths | Kiribati | Both | Age-standardized | CKD-T1DM | Rate | 2021 | 0.082106183 | 0.157909031 | 0.036142887 |
| Deaths | Micronesia (Federated States of) | Both | Age-standardized | CKD-T1DM | Rate | 2021 | 0.074707025 | 0.130378152 | 0.036703829 |
| Deaths | Fiji | Both | Age-standardized | CKD-T1DM | Rate | 2021 | 0.073731452 | 0.125307179 | 0.037030903 |
| Deaths | Marshall Islands | Both | Age-standardized | CKD-T1DM | Rate | 2021 | 0.072290401 | 0.238858339 | 0.015853061 |
| Deaths | Lao People's Democratic Republic | Both | Age-standardized | CKD-T1DM | Rate | 2021 | 0.061271362 | 0.104614369 | 0.032155107 |
| Deaths | Myanmar | Both | Age-standardized | CKD-T1DM | Rate | 2021 | 0.06023433 | 0.097317619 | 0.031475835 |
| Deaths | Philippines | Both | Age-standardized | CKD-T1DM | Rate | 2021 | 0.058084927 | 0.08824358 | 0.033916564 |
| Deaths | Indonesia | Both | Age-standardized | CKD-T1DM | Rate | 2021 | 0.051050092 | 0.08314773 | 0.027384834 |
| Deaths | Tuvalu | Both | Age-standardized | CKD-T1DM | Rate | 2021 | 0.049983871 | 0.087607868 | 0.024301534 |
| Deaths | Vanuatu | Both | Age-standardized | CKD-T1DM | Rate | 2021 | 0.048136772 | 0.092495623 | 0.023100246 |
| Deaths | United States Virgin Islands | Both | Age-standardized | CKD-T1DM | Rate | 2021 | 0.047740836 | 0.089256285 | 0.023051298 |
| Deaths | Solomon Islands | Both | Age-standardized | CKD-T1DM | Rate | 2021 | 0.045874579 | 0.078055015 | 0.023724522 |
| Deaths | Samoa | Both | Age-standardized | CKD-T1DM | Rate | 2021 | 0.040906462 | 0.067632991 | 0.020650522 |
| Deaths | Northern Mariana Islands | Both | Age-standardized | CKD-T1DM | Rate | 2021 | 0.040109438 | 0.068482475 | 0.021175587 |
| Deaths | Papua New Guinea | Both | Age-standardized | CKD-T1DM | Rate | 2021 | 0.036076619 | 0.064967304 | 0.017349972 |
| Deaths | Dominica | Both | Age-standardized | CKD-T1DM | Rate | 2021 | 0.034467669 | 0.057861734 | 0.017006182 |
| Deaths | Cambodia | Both | Age-standardized | CKD-T1DM | Rate | 2021 | 0.03419712 | 0.060257349 | 0.015936922 |
| Deaths | Timor-Leste | Both | Age-standardized | CKD-T1DM | Rate | 2021 | 0.032871004 | 0.058629071 | 0.01672774 |
| Deaths | Saint Kitts and Nevis | Both | Age-standardized | CKD-T1DM | Rate | 2021 | 0.031471421 | 0.054841114 | 0.016085015 |
| Deaths | Thailand | Both | Age-standardized | CKD-T1DM | Rate | 2021 | 0.030057932 | 0.048997995 | 0.015994312 |
| Deaths | Ethiopia | Both | Age-standardized | CKD-T1DM | Rate | 2021 | 0.029796295 | 0.050975414 | 0.015622574 |
| Deaths | Suriname | Both | Age-standardized | CKD-T1DM | Rate | 2021 | 0.029441895 | 0.051026764 | 0.01429452 |
| Deaths | Guyana | Both | Age-standardized | CKD-T1DM | Rate | 2021 | 0.028235974 | 0.050050096 | 0.013929814 |
| Deaths | Grenada | Both | Age-standardized | CKD-T1DM | Rate | 2021 | 0.027172362 | 0.044525847 | 0.014641486 |
| Deaths | Viet Nam | Both | Age-standardized | CKD-T1DM | Rate | 2021 | 0.027147543 | 0.04500097 | 0.012850509 |
| Deaths | Belize | Both | Age-standardized | CKD-T1DM | Rate | 2021 | 0.026698707 | 0.045388023 | 0.013568673 |
| Deaths | Saint Lucia | Both | Age-standardized | CKD-T1DM | Rate | 2021 | 0.025509783 | 0.043594082 | 0.01260389 |
| Deaths | Maldives | Both | Age-standardized | CKD-T1DM | Rate | 2021 | 0.025327799 | 0.041550379 | 0.013712978 |
| Deaths | Saint Vincent and the Grenadines | Both | Age-standardized | CKD-T1DM | Rate | 2021 | 0.024598648 | 0.040881496 | 0.01317658 |
| Deaths | Democratic People's Republic of Korea | Both | Age-standardized | CKD-T1DM | Rate | 2021 | 0.024501432 | 0.04116578 | 0.012784339 |
| Deaths | Antigua and Barbuda | Both | Age-standardized | CKD-T1DM | Rate | 2021 | 0.024402362 | 0.040442287 | 0.012202762 |
| Deaths | Malaysia | Both | Age-standardized | CKD-T1DM | Rate | 2021 | 0.023026692 | 0.037972016 | 0.012032254 |
| Deaths | Sri Lanka | Both | Age-standardized | CKD-T1DM | Rate | 2021 | 0.021997057 | 0.037760671 | 0.01121859 |
| Deaths | Trinidad and Tobago | Both | Age-standardized | CKD-T1DM | Rate | 2021 | 0.021328773 | 0.037485887 | 0.010692213 |
| Deaths | Bahamas | Both | Age-standardized | CKD-T1DM | Rate | 2021 | 0.020700866 | 0.036134716 | 0.010665504 |
| Deaths | Tonga | Both | Age-standardized | CKD-T1DM | Rate | 2021 | 0.020096822 | 0.034757203 | 0.009531473 |
| Deaths | Guam | Both | Age-standardized | CKD-T1DM | Rate | 2021 | 0.018558179 | 0.031916302 | 0.008977874 |
| Deaths | Dominican Republic | Both | Age-standardized | CKD-T1DM | Rate | 2021 | 0.018252978 | 0.031423163 | 0.008444788 |
| Deaths | Haiti | Both | Age-standardized | CKD-T1DM | Rate | 2021 | 0.018095917 | 0.04014412 | 0.007575082 |
| Deaths | Cook Islands | Both | Age-standardized | CKD-T1DM | Rate | 2021 | 0.016619771 | 0.0273389 | 0.008869826 |
| Deaths | Barbados | Both | Age-standardized | CKD-T1DM | Rate | 2021 | 0.014451053 | 0.025569944 | 0.007092276 |
| Deaths | Taiwan (Province of China) | Both | Age-standardized | CKD-T1DM | Rate | 2021 | 0.013856377 | 0.020736525 | 0.008243116 |
| Deaths | China | Both | Age-standardized | CKD-T1DM | Rate | 2021 | 0.013648323 | 0.020594113 | 0.007885831 |
| Deaths | South Sudan | Both | Age-standardized | CKD-T1DM | Rate | 2021 | 0.013424888 | 0.02448562 | 0.006050533 |
| Deaths | Cameroon | Both | Age-standardized | CKD-T1DM | Rate | 2021 | 0.012693375 | 0.023616152 | 0.005653931 |
| Deaths | Zambia | Both | Age-standardized | CKD-T1DM | Rate | 2021 | 0.011961969 | 0.027071444 | 0.004814177 |
| Deaths | Mozambique | Both | Age-standardized | CKD-T1DM | Rate | 2021 | 0.011228556 | 0.021783092 | 0.005233903 |
| Deaths | Somalia | Both | Age-standardized | CKD-T1DM | Rate | 2021 | 0.01114518 | 0.022176047 | 0.004656143 |
| Deaths | Gabon | Both | Age-standardized | CKD-T1DM | Rate | 2021 | 0.011073127 | 0.021255087 | 0.004706461 |
| Deaths | Jamaica | Both | Age-standardized | CKD-T1DM | Rate | 2021 | 0.010993741 | 0.019430676 | 0.005419912 |
| Deaths | Central African Republic | Both | Age-standardized | CKD-T1DM | Rate | 2021 | 0.010803156 | 0.021131185 | 0.004737807 |
| Deaths | Malawi | Both | Age-standardized | CKD-T1DM | Rate | 2021 | 0.010187594 | 0.020230702 | 0.004765745 |
| Deaths | Equatorial Guinea | Both | Age-standardized | CKD-T1DM | Rate | 2021 | 0.010003406 | 0.019552886 | 0.003721565 |
| Deaths | Congo | Both | Age-standardized | CKD-T1DM | Rate | 2021 | 0.009134766 | 0.017020271 | 0.004169398 |
| Deaths | Uganda | Both | Age-standardized | CKD-T1DM | Rate | 2021 | 0.008898533 | 0.016387136 | 0.004183174 |
| Deaths | Bolivia (Plurinational State of) | Both | Age-standardized | CKD-T1DM | Rate | 2021 | 0.008722184 | 0.016420053 | 0.003881139 |
| Deaths | Sao Tome and Principe | Both | Age-standardized | CKD-T1DM | Rate | 2021 | 0.008598633 | 0.016871434 | 0.003708829 |
| Deaths | Kenya | Both | Age-standardized | CKD-T1DM | Rate | 2021 | 0.008421094 | 0.015600295 | 0.004163395 |
| Deaths | Eritrea | Both | Age-standardized | CKD-T1DM | Rate | 2021 | 0.008346766 | 0.017194357 | 0.003570009 |
| Deaths | Democratic Republic of the Congo | Both | Age-standardized | CKD-T1DM | Rate | 2021 | 0.008042549 | 0.01609162 | 0.003459606 |
| Deaths | Ghana | Both | Age-standardized | CKD-T1DM | Rate | 2021 | 0.007953221 | 0.015029293 | 0.003802706 |
| Deaths | Djibouti | Both | Age-standardized | CKD-T1DM | Rate | 2021 | 0.007658396 | 0.014181445 | 0.003343975 |
| Deaths | Burundi | Both | Age-standardized | CKD-T1DM | Rate | 2021 | 0.007579252 | 0.015008513 | 0.003481131 |
| Deaths | Guinea-Bissau | Both | Age-standardized | CKD-T1DM | Rate | 2021 | 0.007441084 | 0.015412021 | 0.003307247 |
| Deaths | Comoros | Both | Age-standardized | CKD-T1DM | Rate | 2021 | 0.007092594 | 0.013125546 | 0.003207421 |
| Deaths | Liberia | Both | Age-standardized | CKD-T1DM | Rate | 2021 | 0.006978447 | 0.012850658 | 0.00308627 |
| Deaths | El Salvador | Both | Age-standardized | CKD-T1DM | Rate | 2021 | 0.006887827 | 0.01191769 | 0.003456992 |
| Deaths | Cuba | Both | Age-standardized | CKD-T1DM | Rate | 2021 | 0.00680659 | 0.011619078 | 0.003649451 |
| Deaths | Madagascar | Both | Age-standardized | CKD-T1DM | Rate | 2021 | 0.006795672 | 0.012885922 | 0.002963071 |
| Deaths | Senegal | Both | Age-standardized | CKD-T1DM | Rate | 2021 | 0.006729479 | 0.013023507 | 0.002801736 |
| Deaths | Gambia | Both | Age-standardized | CKD-T1DM | Rate | 2021 | 0.006581145 | 0.012960264 | 0.003014168 |
| Deaths | Bermuda | Both | Age-standardized | CKD-T1DM | Rate | 2021 | 0.006553933 | 0.01133322 | 0.003226818 |
| Deaths | Angola | Both | Age-standardized | CKD-T1DM | Rate | 2021 | 0.006267059 | 0.012058982 | 0.002898215 |
| Deaths | Peru | Both | Age-standardized | CKD-T1DM | Rate | 2021 | 0.006210355 | 0.011134804 | 0.002807669 |
| Deaths | Ecuador | Both | Age-standardized | CKD-T1DM | Rate | 2021 | 0.006055843 | 0.010576863 | 0.002949526 |
| Deaths | Guatemala | Both | Age-standardized | CKD-T1DM | Rate | 2021 | 0.005796145 | 0.010113939 | 0.002845146 |
| Deaths | Rwanda | Both | Age-standardized | CKD-T1DM | Rate | 2021 | 0.005682092 | 0.010796988 | 0.00259615 |
| Deaths | Mexico | Both | Age-standardized | CKD-T1DM | Rate | 2021 | 0.005223142 | 0.00896524 | 0.00277654 |
| Deaths | Pakistan | Both | Age-standardized | CKD-T1DM | Rate | 2021 | 0.00518864 | 0.008885468 | 0.002786173 |
| Deaths | Burkina Faso | Both | Age-standardized | CKD-T1DM | Rate | 2021 | 0.005176258 | 0.009867445 | 0.002195562 |
| Deaths | Puerto Rico | Both | Age-standardized | CKD-T1DM | Rate | 2021 | 0.005159273 | 0.008316546 | 0.002844873 |
| Deaths | Eswatini | Both | Age-standardized | CKD-T1DM | Rate | 2021 | 0.005142843 | 0.009617978 | 0.002147396 |
| Deaths | Chad | Both | Age-standardized | CKD-T1DM | Rate | 2021 | 0.005105388 | 0.010399476 | 0.002257908 |
| Deaths | Benin | Both | Age-standardized | CKD-T1DM | Rate | 2021 | 0.005100736 | 0.009163029 | 0.002382744 |
| Deaths | Turkmenistan | Both | Age-standardized | CKD-T1DM | Rate | 2021 | 0.004914214 | 0.008970293 | 0.002311115 |
| Deaths | Mali | Both | Age-standardized | CKD-T1DM | Rate | 2021 | 0.004772201 | 0.008856407 | 0.002215355 |
| Deaths | C么te d'Ivoire | Both | Age-standardized | CKD-T1DM | Rate | 2021 | 0.004771384 | 0.008867152 | 0.002044519 |
| Deaths | Togo | Both | Age-standardized | CKD-T1DM | Rate | 2021 | 0.004746515 | 0.008769548 | 0.002057475 |
| Deaths | Sierra Leone | Both | Age-standardized | CKD-T1DM | Rate | 2021 | 0.004243933 | 0.008595708 | 0.001918848 |
| Deaths | Mauritania | Both | Age-standardized | CKD-T1DM | Rate | 2021 | 0.004126279 | 0.007755371 | 0.001748062 |
| Deaths | Nigeria | Both | Age-standardized | CKD-T1DM | Rate | 2021 | 0.004027867 | 0.00777626 | 0.001765415 |
| Deaths | Nicaragua | Both | Age-standardized | CKD-T1DM | Rate | 2021 | 0.003917303 | 0.007005304 | 0.001848617 |
| Deaths | Paraguay | Both | Age-standardized | CKD-T1DM | Rate | 2021 | 0.003860176 | 0.006980928 | 0.001829469 |
| Deaths | Venezuela (Bolivarian Republic of) | Both | Age-standardized | CKD-T1DM | Rate | 2021 | 0.003749788 | 0.006636171 | 0.001769709 |
| Deaths | Cabo Verde | Both | Age-standardized | CKD-T1DM | Rate | 2021 | 0.003689065 | 0.007060241 | 0.001648023 |
| Deaths | Guinea | Both | Age-standardized | CKD-T1DM | Rate | 2021 | 0.003513474 | 0.006888858 | 0.001521801 |
| Deaths | Syrian Arab Republic | Both | Age-standardized | CKD-T1DM | Rate | 2021 | 0.003298793 | 0.006354009 | 0.001438738 |
| Deaths | Seychelles | Both | Age-standardized | CKD-T1DM | Rate | 2021 | 0.003252002 | 0.006108059 | 0.001554855 |
| Deaths | Zimbabwe | Both | Age-standardized | CKD-T1DM | Rate | 2021 | 0.003115996 | 0.005853516 | 0.001372061 |
| Deaths | Lesotho | Both | Age-standardized | CKD-T1DM | Rate | 2021 | 0.003023686 | 0.005680091 | 0.001210426 |
| Deaths | Afghanistan | Both | Age-standardized | CKD-T1DM | Rate | 2021 | 0.003013464 | 0.007061784 | 0.001120681 |
| Deaths | United Republic of Tanzania | Both | Age-standardized | CKD-T1DM | Rate | 2021 | 0.003009638 | 0.005573085 | 0.001443577 |
| Deaths | Brazil | Both | Age-standardized | CKD-T1DM | Rate | 2021 | 0.002854475 | 0.004790803 | 0.001428244 |
| Deaths | Saudi Arabia | Both | Age-standardized | CKD-T1DM | Rate | 2021 | 0.00279278 | 0.005465235 | 0.001175333 |
| Deaths | South Africa | Both | Age-standardized | CKD-T1DM | Rate | 2021 | 0.002724014 | 0.004950376 | 0.00133675 |
| Deaths | Brunei Darussalam | Both | Age-standardized | CKD-T1DM | Rate | 2021 | 0.00251532 | 0.004634696 | 0.001160659 |
| Deaths | Panama | Both | Age-standardized | CKD-T1DM | Rate | 2021 | 0.002498495 | 0.004269377 | 0.001185142 |
| Deaths | Niger | Both | Age-standardized | CKD-T1DM | Rate | 2021 | 0.002460129 | 0.005017794 | 0.000961315 |
| Deaths | Uzbekistan | Both | Age-standardized | CKD-T1DM | Rate | 2021 | 0.002362655 | 0.004183089 | 0.001135774 |
| Deaths | Egypt | Both | Age-standardized | CKD-T1DM | Rate | 2021 | 0.002352468 | 0.004903885 | 0.001026514 |
| Deaths | Nepal | Both | Age-standardized | CKD-T1DM | Rate | 2021 | 0.002272558 | 0.004399067 | 0.001016752 |
| Deaths | Iran (Islamic Republic of) | Both | Age-standardized | CKD-T1DM | Rate | 2021 | 0.00227207 | 0.004079583 | 0.001118374 |
| Deaths | Azerbaijan | Both | Age-standardized | CKD-T1DM | Rate | 2021 | 0.002102421 | 0.004015338 | 0.000970834 |
| Deaths | Bhutan | Both | Age-standardized | CKD-T1DM | Rate | 2021 | 0.002062435 | 0.003941027 | 0.000843583 |
| Deaths | Libya | Both | Age-standardized | CKD-T1DM | Rate | 2021 | 0.002060596 | 0.004097235 | 0.000802443 |
| Deaths | Costa Rica | Both | Age-standardized | CKD-T1DM | Rate | 2021 | 0.002011889 | 0.003458481 | 0.001002069 |
| Deaths | Jordan | Both | Age-standardized | CKD-T1DM | Rate | 2021 | 0.00197063 | 0.003702011 | 0.000927102 |
| Deaths | Botswana | Both | Age-standardized | CKD-T1DM | Rate | 2021 | 0.001942769 | 0.004014353 | 0.000837468 |
| Deaths | Georgia | Both | Age-standardized | CKD-T1DM | Rate | 2021 | 0.001873882 | 0.00329368 | 0.000909834 |
| Deaths | Mongolia | Both | Age-standardized | CKD-T1DM | Rate | 2021 | 0.001836918 | 0.003318399 | 0.000893658 |
| Deaths | Estonia | Both | Age-standardized | CKD-T1DM | Rate | 2021 | 0.00181035 | 0.003148258 | 0.000909675 |
| Deaths | Iraq | Both | Age-standardized | CKD-T1DM | Rate | 2021 | 0.001795764 | 0.003779526 | 0.000778388 |
| Deaths | Kyrgyzstan | Both | Age-standardized | CKD-T1DM | Rate | 2021 | 0.001642557 | 0.003044446 | 0.000781284 |
| Deaths | India | Both | Age-standardized | CKD-T1DM | Rate | 2021 | 0.001557143 | 0.002766374 | 0.000764242 |
| Deaths | Namibia | Both | Age-standardized | CKD-T1DM | Rate | 2021 | 0.001543064 | 0.003116273 | 0.000660928 |
| Deaths | Bangladesh | Both | Age-standardized | CKD-T1DM | Rate | 2021 | 0.001537886 | 0.00300061 | 0.00075377 |
| Deaths | Palestine | Both | Age-standardized | CKD-T1DM | Rate | 2021 | 0.001449786 | 0.002798581 | 0.000639888 |
| Deaths | Oman | Both | Age-standardized | CKD-T1DM | Rate | 2021 | 0.001399354 | 0.002702019 | 0.000646564 |
| Deaths | Algeria | Both | Age-standardized | CKD-T1DM | Rate | 2021 | 0.001371796 | 0.002615623 | 0.000598566 |
| Deaths | Bahrain | Both | Age-standardized | CKD-T1DM | Rate | 2021 | 0.001352618 | 0.002576106 | 0.000593773 |
| Deaths | Sudan | Both | Age-standardized | CKD-T1DM | Rate | 2021 | 0.001329308 | 0.002824231 | 0.000535326 |
| Deaths | Colombia | Both | Age-standardized | CKD-T1DM | Rate | 2021 | 0.001286526 | 0.002197021 | 0.000638181 |
| Deaths | T眉rkiye | Both | Age-standardized | CKD-T1DM | Rate | 2021 | 0.001241575 | 0.002323206 | 0.000552195 |
| Deaths | Kazakhstan | Both | Age-standardized | CKD-T1DM | Rate | 2021 | 0.001198473 | 0.002207895 | 0.000573939 |
| Deaths | Argentina | Both | Age-standardized | CKD-T1DM | Rate | 2021 | 0.001187798 | 0.002053657 | 0.000594507 |
| Deaths | Honduras | Both | Age-standardized | CKD-T1DM | Rate | 2021 | 0.001158352 | 0.002280536 | 0.000501882 |
| Deaths | Lebanon | Both | Age-standardized | CKD-T1DM | Rate | 2021 | 0.001131006 | 0.002196132 | 0.000484976 |
| Deaths | Morocco | Both | Age-standardized | CKD-T1DM | Rate | 2021 | 0.001060359 | 0.002126441 | 0.000467036 |
| Deaths | Qatar | Both | Age-standardized | CKD-T1DM | Rate | 2021 | 0.001019409 | 0.00199875 | 0.000451251 |
| Deaths | Armenia | Both | Age-standardized | CKD-T1DM | Rate | 2021 | 0.001005351 | 0.001858472 | 0.000474496 |
| Deaths | Singapore | Both | Age-standardized | CKD-T1DM | Rate | 2021 | 0.000991545 | 0.001814536 | 0.000466594 |
| Deaths | Montenegro | Both | Age-standardized | CKD-T1DM | Rate | 2021 | 0.000946178 | 0.001788388 | 0.000446927 |
| Deaths | United Arab Emirates | Both | Age-standardized | CKD-T1DM | Rate | 2021 | 0.000882395 | 0.001743715 | 0.000324074 |
| Deaths | Kuwait | Both | Age-standardized | CKD-T1DM | Rate | 2021 | 0.000833782 | 0.001596342 | 0.000377532 |
| Deaths | Tunisia | Both | Age-standardized | CKD-T1DM | Rate | 2021 | 0.000810554 | 0.001474599 | 0.000341612 |
| Deaths | Bosnia and Herzegovina | Both | Age-standardized | CKD-T1DM | Rate | 2021 | 0.000699104 | 0.001238 | 0.000344939 |
| Deaths | Uruguay | Both | Age-standardized | CKD-T1DM | Rate | 2021 | 0.000692146 | 0.001190347 | 0.000340343 |
| Deaths | Russian Federation | Both | Age-standardized | CKD-T1DM | Rate | 2021 | 0.000661138 | 0.00112961 | 0.000363195 |
| Deaths | Bulgaria | Both | Age-standardized | CKD-T1DM | Rate | 2021 | 0.000651972 | 0.001187715 | 0.000321885 |
| Deaths | Republic of Korea | Both | Age-standardized | CKD-T1DM | Rate | 2021 | 0.000628401 | 0.001129005 | 0.000306876 |
| Deaths | Chile | Both | Age-standardized | CKD-T1DM | Rate | 2021 | 0.000608862 | 0.00105227 | 0.000307039 |
| Deaths | Yemen | Both | Age-standardized | CKD-T1DM | Rate | 2021 | 0.0005725 | 0.001514851 | 0.000202073 |
| Deaths | North Macedonia | Both | Age-standardized | CKD-T1DM | Rate | 2021 | 0.00057074 | 0.001088873 | 0.000258448 |
| Deaths | Serbia | Both | Age-standardized | CKD-T1DM | Rate | 2021 | 0.000562502 | 0.001013751 | 0.000291615 |
| Deaths | Ukraine | Both | Age-standardized | CKD-T1DM | Rate | 2021 | 0.000542563 | 0.000921752 | 0.000270256 |
| Deaths | Tajikistan | Both | Age-standardized | CKD-T1DM | Rate | 2021 | 0.000500803 | 0.000998591 | 0.000208358 |
| Deaths | Republic of Moldova | Both | Age-standardized | CKD-T1DM | Rate | 2021 | 0.000489162 | 0.000841574 | 0.000243653 |
| Deaths | Slovakia | Both | Age-standardized | CKD-T1DM | Rate | 2021 | 0.000481668 | 0.000871534 | 0.000237294 |
| Deaths | Latvia | Both | Age-standardized | CKD-T1DM | Rate | 2021 | 0.000480669 | 0.00085554 | 0.0002335 |
| Deaths | Albania | Both | Age-standardized | CKD-T1DM | Rate | 2021 | 0.000457027 | 0.000880303 | 0.000218272 |
| Deaths | Lithuania | Both | Age-standardized | CKD-T1DM | Rate | 2021 | 0.000428302 | 0.000744272 | 0.000217926 |
| Deaths | Japan | Both | Age-standardized | CKD-T1DM | Rate | 2021 | 0.000409932 | 0.00071395 | 0.00022847 |
| Deaths | Israel | Both | Age-standardized | CKD-T1DM | Rate | 2021 | 0.000340526 | 0.000606625 | 0.00015976 |
| Deaths | Croatia | Both | Age-standardized | CKD-T1DM | Rate | 2021 | 0.000304821 | 0.000549614 | 0.000142781 |
| Deaths | Poland | Both | Age-standardized | CKD-T1DM | Rate | 2021 | 0.000277194 | 0.000480056 | 0.000145211 |
| Deaths | Malta | Both | Age-standardized | CKD-T1DM | Rate | 2021 | 0.000274884 | 0.000483124 | 0.000137771 |
| Deaths | Greece | Both | Age-standardized | CKD-T1DM | Rate | 2021 | 0.000243171 | 0.000386635 | 0.000129266 |
| Deaths | Hungary | Both | Age-standardized | CKD-T1DM | Rate | 2021 | 0.000225508 | 0.000444423 | 9.94E-05 |
| Deaths | Czechia | Both | Age-standardized | CKD-T1DM | Rate | 2021 | 0.000215105 | 0.00042402 | 0.000102598 |
| Deaths | Portugal | Both | Age-standardized | CKD-T1DM | Rate | 2021 | 0.000209437 | 0.000362648 | 0.000106653 |
| Deaths | Belarus | Both | Age-standardized | CKD-T1DM | Rate | 2021 | 0.000200158 | 0.000351834 | 9.98E-05 |
| Deaths | Austria | Both | Age-standardized | CKD-T1DM | Rate | 2021 | 0.000191639 | 0.000309951 | 0.000105095 |
| Deaths | United States of America | Both | Age-standardized | CKD-T1DM | Rate | 2021 | 0.000186465 | 0.000307937 | 9.60E-05 |
| Deaths | Monaco | Both | Age-standardized | CKD-T1DM | Rate | 2021 | 0.000181638 | 0.000320904 | 8.88E-05 |
| Deaths | Romania | Both | Age-standardized | CKD-T1DM | Rate | 2021 | 0.000172812 | 0.000315451 | 8.16E-05 |
| Deaths | New Zealand | Both | Age-standardized | CKD-T1DM | Rate | 2021 | 0.000166526 | 0.000273416 | 8.62E-05 |
| Deaths | United Kingdom | Both | Age-standardized | CKD-T1DM | Rate | 2021 | 0.000158493 | 0.000271125 | 8.29E-05 |
| Deaths | Spain | Both | Age-standardized | CKD-T1DM | Rate | 2021 | 0.000155992 | 0.000272254 | 8.25E-05 |
| Deaths | Italy | Both | Age-standardized | CKD-T1DM | Rate | 2021 | 0.000144703 | 0.000248903 | 7.60E-05 |
| Deaths | Germany | Both | Age-standardized | CKD-T1DM | Rate | 2021 | 0.000133627 | 0.000241057 | 6.46E-05 |
| Deaths | Slovenia | Both | Age-standardized | CKD-T1DM | Rate | 2021 | 0.000132961 | 0.000251128 | 6.38E-05 |
| Deaths | Andorra | Both | Age-standardized | CKD-T1DM | Rate | 2021 | 0.000127199 | 0.000235164 | 5.75E-05 |
| Deaths | Belgium | Both | Age-standardized | CKD-T1DM | Rate | 2021 | 0.000122892 | 0.00020563 | 6.25E-05 |
| Deaths | Luxembourg | Both | Age-standardized | CKD-T1DM | Rate | 2021 | 0.000117239 | 0.000206873 | 5.55E-05 |
| Deaths | Switzerland | Both | Age-standardized | CKD-T1DM | Rate | 2021 | 0.00010384 | 0.00017193 | 5.45E-05 |
| Deaths | Netherlands | Both | Age-standardized | CKD-T1DM | Rate | 2021 | 9.43E-05 | 0.000158139 | 4.90E-05 |
| Deaths | Canada | Both | Age-standardized | CKD-T1DM | Rate | 2021 | 8.82E-05 | 0.000164518 | 4.12E-05 |
| Deaths | Finland | Both | Age-standardized | CKD-T1DM | Rate | 2021 | 8.80E-05 | 0.000142453 | 4.69E-05 |
| Deaths | Norway | Both | Age-standardized | CKD-T1DM | Rate | 2021 | 8.60E-05 | 0.000143137 | 4.50E-05 |
| Deaths | France | Both | Age-standardized | CKD-T1DM | Rate | 2021 | 8.50E-05 | 0.000140891 | 4.59E-05 |
| Deaths | Ireland | Both | Age-standardized | CKD-T1DM | Rate | 2021 | 7.88E-05 | 0.00013652 | 3.92E-05 |
| Deaths | Cyprus | Both | Age-standardized | CKD-T1DM | Rate | 2021 | 7.84E-05 | 0.000163401 | 3.56E-05 |
| Deaths | Greenland | Both | Age-standardized | CKD-T1DM | Rate | 2021 | 7.63E-05 | 0.00016135 | 3.14E-05 |
| Deaths | Denmark | Both | Age-standardized | CKD-T1DM | Rate | 2021 | 7.53E-05 | 0.000120235 | 4.11E-05 |
| Deaths | San Marino | Both | Age-standardized | CKD-T1DM | Rate | 2021 | 6.73E-05 | 0.00012447 | 3.18E-05 |
| Deaths | Sweden | Both | Age-standardized | CKD-T1DM | Rate | 2021 | 6.05E-05 | 0.000104031 | 3.17E-05 |
| Deaths | Iceland | Both | Age-standardized | CKD-T1DM | Rate | 2021 | 5.49E-05 | 9.76E-05 | 2.82E-05 |
| Deaths | Australia | Both | Age-standardized | CKD-T1DM | Rate | 2021 | 4.26E-05 | 7.65E-05 | 1.93E-05 |

Appendix

**Appendix 18：Global disease burden distribution of Chronic kidney disease due to diabetes mellitus type 1 in children and adolescents from 1990 to 2021**

| **measure** | **location** | **sex** | **age** | **cause** | **metric** | **year** | **val** | **upper** | **lower** |
| --- | --- | --- | --- | --- | --- | --- | --- | --- | --- |
| DALYs | Niue | Both | Age-standardized | CKD-T1DM | Rate | 2021 | 12.61114856 | 24.19498362 | 5.40983008 |
| DALYs | American Samoa | Both | Age-standardized | CKD-T1DM | Rate | 2021 | 9.175551602 | 14.58458879 | 4.85295026 |
| DALYs | Tokelau | Both | Age-standardized | CKD-T1DM | Rate | 2021 | 9.05552374 | 15.95801173 | 4.64219765 |
| DALYs | Palau | Both | Age-standardized | CKD-T1DM | Rate | 2021 | 8.434401275 | 14.84182832 | 3.75988112 |
| DALYs | Mauritius | Both | Age-standardized | CKD-T1DM | Rate | 2021 | 6.515854605 | 9.990941237 | 3.93352651 |
| DALYs | Nauru | Both | Age-standardized | CKD-T1DM | Rate | 2021 | 6.100863448 | 10.1614776 | 2.89615632 |
| DALYs | Kiribati | Both | Age-standardized | CKD-T1DM | Rate | 2021 | 6.069970501 | 11.61443996 | 2.69327964 |
| DALYs | Micronesia (Federated States of) | Both | Age-standardized | CKD-T1DM | Rate | 2021 | 5.504603777 | 9.535701356 | 2.72733001 |
| DALYs | Fiji | Both | Age-standardized | CKD-T1DM | Rate | 2021 | 5.477473798 | 9.245628535 | 2.7723313 |
| DALYs | Marshall Islands | Both | Age-standardized | CKD-T1DM | Rate | 2021 | 5.323054969 | 17.5214514 | 1.19048958 |
| DALYs | Lao People's Democratic Republic | Both | Age-standardized | CKD-T1DM | Rate | 2021 | 4.581598406 | 7.773018563 | 2.43187127 |
| DALYs | Myanmar | Both | Age-standardized | CKD-T1DM | Rate | 2021 | 4.518077231 | 7.295101224 | 2.40551013 |
| DALYs | Philippines | Both | Age-standardized | CKD-T1DM | Rate | 2021 | 4.358784449 | 6.575873716 | 2.58079954 |
| DALYs | Indonesia | Both | Age-standardized | CKD-T1DM | Rate | 2021 | 3.814182603 | 6.1698908 | 2.05305784 |
| DALYs | Tuvalu | Both | Age-standardized | CKD-T1DM | Rate | 2021 | 3.695468381 | 6.430975296 | 1.82494142 |
| DALYs | Vanuatu | Both | Age-standardized | CKD-T1DM | Rate | 2021 | 3.563977787 | 6.847066926 | 1.71147675 |
| DALYs | United States Virgin Islands | Both | Age-standardized | CKD-T1DM | Rate | 2021 | 3.523265632 | 6.495901901 | 1.73589541 |
| DALYs | Solomon Islands | Both | Age-standardized | CKD-T1DM | Rate | 2021 | 3.409329575 | 5.749177688 | 1.77852043 |
| DALYs | Samoa | Both | Age-standardized | CKD-T1DM | Rate | 2021 | 3.030320439 | 5.008069749 | 1.5369641 |
| DALYs | Northern Mariana Islands | Both | Age-standardized | CKD-T1DM | Rate | 2021 | 2.971452442 | 5.029476293 | 1.57364756 |
| DALYs | Papua New Guinea | Both | Age-standardized | CKD-T1DM | Rate | 2021 | 2.686376067 | 4.815636255 | 1.30228734 |
| DALYs | Dominica | Both | Age-standardized | CKD-T1DM | Rate | 2021 | 2.609204871 | 4.301042113 | 1.29974508 |
| DALYs | Cambodia | Both | Age-standardized | CKD-T1DM | Rate | 2021 | 2.569797752 | 4.467201881 | 1.23092444 |
| DALYs | Timor-Leste | Both | Age-standardized | CKD-T1DM | Rate | 2021 | 2.462347427 | 4.344205764 | 1.25997523 |
| DALYs | Saint Kitts and Nevis | Both | Age-standardized | CKD-T1DM | Rate | 2021 | 2.374541814 | 4.07902203 | 1.25782171 |
| DALYs | Thailand | Both | Age-standardized | CKD-T1DM | Rate | 2021 | 2.256873875 | 3.664101935 | 1.20580992 |
| DALYs | Suriname | Both | Age-standardized | CKD-T1DM | Rate | 2021 | 2.221215676 | 3.827369836 | 1.09481108 |
| DALYs | Ethiopia | Both | Age-standardized | CKD-T1DM | Rate | 2021 | 2.213410421 | 3.75518628 | 1.1721956 |
| DALYs | Guyana | Both | Age-standardized | CKD-T1DM | Rate | 2021 | 2.143236108 | 3.719238764 | 1.06297593 |
| DALYs | Grenada | Both | Age-standardized | CKD-T1DM | Rate | 2021 | 2.068954141 | 3.349120034 | 1.13864803 |
| DALYs | Viet Nam | Both | Age-standardized | CKD-T1DM | Rate | 2021 | 2.041044279 | 3.367065327 | 1.01249568 |
| DALYs | Belize | Both | Age-standardized | CKD-T1DM | Rate | 2021 | 2.026171864 | 3.412108995 | 1.04174823 |
| DALYs | Saint Lucia | Both | Age-standardized | CKD-T1DM | Rate | 2021 | 1.929776301 | 3.244021337 | 0.97388097 |
| DALYs | Maldives | Both | Age-standardized | CKD-T1DM | Rate | 2021 | 1.923347741 | 3.135449549 | 1.05124395 |
| DALYs | Saint Vincent and the Grenadines | Both | Age-standardized | CKD-T1DM | Rate | 2021 | 1.873308108 | 3.051903547 | 1.01868261 |
| DALYs | Antigua and Barbuda | Both | Age-standardized | CKD-T1DM | Rate | 2021 | 1.850146344 | 3.008012279 | 0.95390635 |
| DALYs | Democratic People's Republic of Korea | Both | Age-standardized | CKD-T1DM | Rate | 2021 | 1.817933946 | 3.040365024 | 0.95345315 |
| DALYs | Malaysia | Both | Age-standardized | CKD-T1DM | Rate | 2021 | 1.738790418 | 2.839412687 | 0.93379014 |
| DALYs | Sri Lanka | Both | Age-standardized | CKD-T1DM | Rate | 2021 | 1.654584596 | 2.810101186 | 0.86939616 |
| DALYs | Trinidad and Tobago | Both | Age-standardized | CKD-T1DM | Rate | 2021 | 1.615298433 | 2.779262039 | 0.84601764 |
| DALYs | Bahamas | Both | Age-standardized | CKD-T1DM | Rate | 2021 | 1.565614223 | 2.671169108 | 0.81380043 |
| DALYs | Tonga | Both | Age-standardized | CKD-T1DM | Rate | 2021 | 1.510143247 | 2.585894209 | 0.74292748 |
| DALYs | Haiti | Both | Age-standardized | CKD-T1DM | Rate | 2021 | 1.407786228 | 2.99579671 | 0.61355418 |
| DALYs | Guam | Both | Age-standardized | CKD-T1DM | Rate | 2021 | 1.394180661 | 2.385683801 | 0.70258527 |
| DALYs | Dominican Republic | Both | Age-standardized | CKD-T1DM | Rate | 2021 | 1.383744654 | 2.367636945 | 0.6637173 |
| DALYs | Cook Islands | Both | Age-standardized | CKD-T1DM | Rate | 2021 | 1.241263707 | 2.058429673 | 0.67339972 |
| DALYs | Barbados | Both | Age-standardized | CKD-T1DM | Rate | 2021 | 1.113647289 | 1.931172243 | 0.5673427 |
| DALYs | Taiwan (Province of China) | Both | Age-standardized | CKD-T1DM | Rate | 2021 | 1.063254429 | 1.561909998 | 0.65450133 |
| DALYs | South Sudan | Both | Age-standardized | CKD-T1DM | Rate | 2021 | 1.024388796 | 1.823411535 | 0.46850123 |
| DALYs | China | Both | Age-standardized | CKD-T1DM | Rate | 2021 | 1.018967857 | 1.530047622 | 0.59113959 |
| DALYs | Cameroon | Both | Age-standardized | CKD-T1DM | Rate | 2021 | 0.976641722 | 1.768067744 | 0.45491794 |
| DALYs | Zambia | Both | Age-standardized | CKD-T1DM | Rate | 2021 | 0.922752877 | 2.063321514 | 0.40822935 |
| DALYs | Mozambique | Both | Age-standardized | CKD-T1DM | Rate | 2021 | 0.865500776 | 1.635207595 | 0.41325791 |
| DALYs | Gabon | Both | Age-standardized | CKD-T1DM | Rate | 2021 | 0.862860914 | 1.622130732 | 0.37965021 |
| DALYs | Jamaica | Both | Age-standardized | CKD-T1DM | Rate | 2021 | 0.861826692 | 1.473131912 | 0.43712025 |
| DALYs | Somalia | Both | Age-standardized | CKD-T1DM | Rate | 2021 | 0.855049774 | 1.634451665 | 0.37303195 |
| DALYs | Central African Republic | Both | Age-standardized | CKD-T1DM | Rate | 2021 | 0.827111911 | 1.57487222 | 0.37929922 |
| DALYs | Malawi | Both | Age-standardized | CKD-T1DM | Rate | 2021 | 0.799477297 | 1.53243714 | 0.38150139 |
| DALYs | Equatorial Guinea | Both | Age-standardized | CKD-T1DM | Rate | 2021 | 0.76949717 | 1.457190732 | 0.30413 |
| DALYs | Congo | Both | Age-standardized | CKD-T1DM | Rate | 2021 | 0.706544685 | 1.265306802 | 0.33279691 |
| DALYs | Uganda | Both | Age-standardized | CKD-T1DM | Rate | 2021 | 0.68630365 | 1.236018612 | 0.33103429 |
| DALYs | Bolivia (Plurinational State of) | Both | Age-standardized | CKD-T1DM | Rate | 2021 | 0.673818425 | 1.248938974 | 0.31234745 |
| DALYs | Sao Tome and Principe | Both | Age-standardized | CKD-T1DM | Rate | 2021 | 0.671619459 | 1.28047241 | 0.30559445 |
| DALYs | Eritrea | Both | Age-standardized | CKD-T1DM | Rate | 2021 | 0.642071643 | 1.298715089 | 0.28308065 |
| DALYs | Kenya | Both | Age-standardized | CKD-T1DM | Rate | 2021 | 0.63750564 | 1.158399461 | 0.32279157 |
| DALYs | Democratic Republic of the Congo | Both | Age-standardized | CKD-T1DM | Rate | 2021 | 0.627856027 | 1.205992403 | 0.2907521 |
| DALYs | Ghana | Both | Age-standardized | CKD-T1DM | Rate | 2021 | 0.608468232 | 1.138143198 | 0.31296633 |
| DALYs | Djibouti | Both | Age-standardized | CKD-T1DM | Rate | 2021 | 0.598333395 | 1.057386108 | 0.28064965 |
| DALYs | Guinea-Bissau | Both | Age-standardized | CKD-T1DM | Rate | 2021 | 0.587597525 | 1.164779741 | 0.26661098 |
| DALYs | Burundi | Both | Age-standardized | CKD-T1DM | Rate | 2021 | 0.58407246 | 1.111576025 | 0.28224639 |
| DALYs | Comoros | Both | Age-standardized | CKD-T1DM | Rate | 2021 | 0.550099179 | 0.998060441 | 0.25495525 |
| DALYs | Liberia | Both | Age-standardized | CKD-T1DM | Rate | 2021 | 0.545370042 | 0.983843579 | 0.24950928 |
| DALYs | El Salvador | Both | Age-standardized | CKD-T1DM | Rate | 2021 | 0.531037706 | 0.901405137 | 0.27424944 |
| DALYs | Senegal | Both | Age-standardized | CKD-T1DM | Rate | 2021 | 0.52896249 | 0.995468847 | 0.23294673 |
| DALYs | Madagascar | Both | Age-standardized | CKD-T1DM | Rate | 2021 | 0.527073388 | 0.977253617 | 0.24859784 |
| DALYs | Cuba | Both | Age-standardized | CKD-T1DM | Rate | 2021 | 0.525219546 | 0.864704412 | 0.28380935 |
| DALYs | Bermuda | Both | Age-standardized | CKD-T1DM | Rate | 2021 | 0.5177684 | 0.875878711 | 0.26025501 |
| DALYs | Gambia | Both | Age-standardized | CKD-T1DM | Rate | 2021 | 0.517281228 | 0.981680428 | 0.24792627 |
| DALYs | Angola | Both | Age-standardized | CKD-T1DM | Rate | 2021 | 0.48725331 | 0.918564065 | 0.22722741 |
| DALYs | Peru | Both | Age-standardized | CKD-T1DM | Rate | 2021 | 0.485205491 | 0.852453543 | 0.22844687 |
| DALYs | Turkmenistan | Both | Age-standardized | CKD-T1DM | Rate | 2021 | 0.467560743 | 0.852211536 | 0.23807372 |
| DALYs | Guatemala | Both | Age-standardized | CKD-T1DM | Rate | 2021 | 0.466707709 | 0.806321115 | 0.24039395 |
| DALYs | Ecuador | Both | Age-standardized | CKD-T1DM | Rate | 2021 | 0.461364874 | 0.806042917 | 0.23155461 |
| DALYs | Pakistan | Both | Age-standardized | CKD-T1DM | Rate | 2021 | 0.451267189 | 0.719567387 | 0.26633006 |
| DALYs | Rwanda | Both | Age-standardized | CKD-T1DM | Rate | 2021 | 0.44470742 | 0.815495163 | 0.21547072 |
| DALYs | Puerto Rico | Both | Age-standardized | CKD-T1DM | Rate | 2021 | 0.432969416 | 0.680127844 | 0.24835118 |
| DALYs | Burkina Faso | Both | Age-standardized | CKD-T1DM | Rate | 2021 | 0.42238788 | 0.764965013 | 0.20210793 |
| DALYs | Benin | Both | Age-standardized | CKD-T1DM | Rate | 2021 | 0.413769876 | 0.711877248 | 0.20540565 |
| DALYs | Eswatini | Both | Age-standardized | CKD-T1DM | Rate | 2021 | 0.41264714 | 0.749638719 | 0.17722485 |
| DALYs | Chad | Both | Age-standardized | CKD-T1DM | Rate | 2021 | 0.411472136 | 0.799774828 | 0.19083887 |
| DALYs | Mexico | Both | Age-standardized | CKD-T1DM | Rate | 2021 | 0.410864767 | 0.684804795 | 0.22678735 |
| DALYs | Mali | Both | Age-standardized | CKD-T1DM | Rate | 2021 | 0.399567226 | 0.745499824 | 0.1934677 |
| DALYs | Togo | Both | Age-standardized | CKD-T1DM | Rate | 2021 | 0.392562918 | 0.681075078 | 0.18365557 |
| DALYs | C么te d'Ivoire | Both | Age-standardized | CKD-T1DM | Rate | 2021 | 0.387800185 | 0.694345732 | 0.17824947 |
| DALYs | Nigeria | Both | Age-standardized | CKD-T1DM | Rate | 2021 | 0.352186657 | 0.639123742 | 0.18334567 |
| DALYs | Sierra Leone | Both | Age-standardized | CKD-T1DM | Rate | 2021 | 0.351001225 | 0.678022335 | 0.18022102 |
| DALYs | Uzbekistan | Both | Age-standardized | CKD-T1DM | Rate | 2021 | 0.345170597 | 0.748771554 | 0.15735072 |
| DALYs | Mauritania | Both | Age-standardized | CKD-T1DM | Rate | 2021 | 0.337470714 | 0.60799067 | 0.15293475 |
| DALYs | Nicaragua | Both | Age-standardized | CKD-T1DM | Rate | 2021 | 0.317727413 | 0.550098618 | 0.15803942 |
| DALYs | Paraguay | Both | Age-standardized | CKD-T1DM | Rate | 2021 | 0.31383833 | 0.538611023 | 0.15077368 |
| DALYs | Guinea | Both | Age-standardized | CKD-T1DM | Rate | 2021 | 0.298960293 | 0.558231489 | 0.14467743 |
| DALYs | Cabo Verde | Both | Age-standardized | CKD-T1DM | Rate | 2021 | 0.294503698 | 0.554570635 | 0.13727392 |
| DALYs | Venezuela (Bolivarian Republic of) | Both | Age-standardized | CKD-T1DM | Rate | 2021 | 0.293535215 | 0.511790747 | 0.14422828 |
| DALYs | Seychelles | Both | Age-standardized | CKD-T1DM | Rate | 2021 | 0.288546976 | 0.524876656 | 0.14739165 |
| DALYs | Syrian Arab Republic | Both | Age-standardized | CKD-T1DM | Rate | 2021 | 0.282383626 | 0.529004923 | 0.13764148 |
| DALYs | United Republic of Tanzania | Both | Age-standardized | CKD-T1DM | Rate | 2021 | 0.275284527 | 0.499544646 | 0.14147078 |
| DALYs | Azerbaijan | Both | Age-standardized | CKD-T1DM | Rate | 2021 | 0.273916408 | 0.613436226 | 0.12134042 |
| DALYs | Brunei Darussalam | Both | Age-standardized | CKD-T1DM | Rate | 2021 | 0.257740163 | 0.438682708 | 0.13447962 |
| DALYs | Zimbabwe | Both | Age-standardized | CKD-T1DM | Rate | 2021 | 0.256068158 | 0.459253057 | 0.11719101 |
| DALYs | Lesotho | Both | Age-standardized | CKD-T1DM | Rate | 2021 | 0.252912606 | 0.473607686 | 0.11100728 |
| DALYs | Brazil | Both | Age-standardized | CKD-T1DM | Rate | 2021 | 0.250561026 | 0.39156792 | 0.1424992 |
| DALYs | Afghanistan | Both | Age-standardized | CKD-T1DM | Rate | 2021 | 0.249611932 | 0.543622623 | 0.10938088 |
| DALYs | Saudi Arabia | Both | Age-standardized | CKD-T1DM | Rate | 2021 | 0.241015251 | 0.438931854 | 0.11717022 |
| DALYs | South Africa | Both | Age-standardized | CKD-T1DM | Rate | 2021 | 0.233277415 | 0.402352054 | 0.12799097 |
| DALYs | Nepal | Both | Age-standardized | CKD-T1DM | Rate | 2021 | 0.222236211 | 0.411663475 | 0.1064991 |
| DALYs | Egypt | Both | Age-standardized | CKD-T1DM | Rate | 2021 | 0.218947098 | 0.403108122 | 0.1013368 |
| DALYs | Niger | Both | Age-standardized | CKD-T1DM | Rate | 2021 | 0.212314091 | 0.425493403 | 0.09523535 |
| DALYs | Georgia | Both | Age-standardized | CKD-T1DM | Rate | 2021 | 0.205851556 | 0.40027574 | 0.10520781 |
| DALYs | Panama | Both | Age-standardized | CKD-T1DM | Rate | 2021 | 0.204363974 | 0.354662287 | 0.10569929 |
| DALYs | Bhutan | Both | Age-standardized | CKD-T1DM | Rate | 2021 | 0.201341332 | 0.366270799 | 0.08966499 |
| DALYs | Kyrgyzstan | Both | Age-standardized | CKD-T1DM | Rate | 2021 | 0.199057688 | 0.418540341 | 0.09709399 |
| DALYs | Estonia | Both | Age-standardized | CKD-T1DM | Rate | 2021 | 0.196894366 | 0.380607395 | 0.09718465 |
| DALYs | Iran (Islamic Republic of) | Both | Age-standardized | CKD-T1DM | Rate | 2021 | 0.196140511 | 0.332374327 | 0.10718735 |
| DALYs | Mongolia | Both | Age-standardized | CKD-T1DM | Rate | 2021 | 0.194950895 | 0.353964287 | 0.09635531 |
| DALYs | Libya | Both | Age-standardized | CKD-T1DM | Rate | 2021 | 0.179974117 | 0.330173631 | 0.07869215 |
| DALYs | Jordan | Both | Age-standardized | CKD-T1DM | Rate | 2021 | 0.17955946 | 0.308144214 | 0.08721799 |
| DALYs | Botswana | Both | Age-standardized | CKD-T1DM | Rate | 2021 | 0.174299098 | 0.329449843 | 0.07766769 |
| DALYs | Montenegro | Both | Age-standardized | CKD-T1DM | Rate | 2021 | 0.171286246 | 0.490888968 | 0.06651098 |
| DALYs | Bulgaria | Both | Age-standardized | CKD-T1DM | Rate | 2021 | 0.169732518 | 0.510000906 | 0.05963908 |
| DALYs | North Macedonia | Both | Age-standardized | CKD-T1DM | Rate | 2021 | 0.162383201 | 0.500459271 | 0.05574508 |
| DALYs | Costa Rica | Both | Age-standardized | CKD-T1DM | Rate | 2021 | 0.161980344 | 0.289404973 | 0.08530784 |
| DALYs | Iraq | Both | Age-standardized | CKD-T1DM | Rate | 2021 | 0.155507831 | 0.308899821 | 0.07101167 |
| DALYs | India | Both | Age-standardized | CKD-T1DM | Rate | 2021 | 0.154667635 | 0.243997196 | 0.09632906 |
| DALYs | Kazakhstan | Both | Age-standardized | CKD-T1DM | Rate | 2021 | 0.149215502 | 0.280209335 | 0.0693596 |
| DALYs | Palestine | Both | Age-standardized | CKD-T1DM | Rate | 2021 | 0.148679513 | 0.293879312 | 0.06675898 |
| DALYs | Oman | Both | Age-standardized | CKD-T1DM | Rate | 2021 | 0.148166776 | 0.274701826 | 0.07024632 |
| DALYs | Albania | Both | Age-standardized | CKD-T1DM | Rate | 2021 | 0.142140351 | 0.449124821 | 0.04431328 |
| DALYs | Bangladesh | Both | Age-standardized | CKD-T1DM | Rate | 2021 | 0.141422458 | 0.260596497 | 0.07543582 |
| DALYs | Armenia | Both | Age-standardized | CKD-T1DM | Rate | 2021 | 0.140763626 | 0.317901613 | 0.06341643 |
| DALYs | Namibia | Both | Age-standardized | CKD-T1DM | Rate | 2021 | 0.139497721 | 0.257803723 | 0.06695097 |
| DALYs | Bosnia and Herzegovina | Both | Age-standardized | CKD-T1DM | Rate | 2021 | 0.137277267 | 0.340902244 | 0.05723929 |
| DALYs | Bahrain | Both | Age-standardized | CKD-T1DM | Rate | 2021 | 0.130281606 | 0.238609294 | 0.06340311 |
| DALYs | Sudan | Both | Age-standardized | CKD-T1DM | Rate | 2021 | 0.128754746 | 0.247990668 | 0.0573007 |
| DALYs | Algeria | Both | Age-standardized | CKD-T1DM | Rate | 2021 | 0.128674048 | 0.251473986 | 0.06206402 |
| DALYs | Republic of Moldova | Both | Age-standardized | CKD-T1DM | Rate | 2021 | 0.125561518 | 0.405777783 | 0.04308462 |
| DALYs | T眉rkiye | Both | Age-standardized | CKD-T1DM | Rate | 2021 | 0.122634754 | 0.216461274 | 0.06103418 |
| DALYs | United Arab Emirates | Both | Age-standardized | CKD-T1DM | Rate | 2021 | 0.119821413 | 0.243131251 | 0.05122653 |
| DALYs | Argentina | Both | Age-standardized | CKD-T1DM | Rate | 2021 | 0.118431416 | 0.208281916 | 0.06358011 |
| DALYs | Slovakia | Both | Age-standardized | CKD-T1DM | Rate | 2021 | 0.117146011 | 0.305411056 | 0.04262103 |
| DALYs | Morocco | Both | Age-standardized | CKD-T1DM | Rate | 2021 | 0.109710037 | 0.215539213 | 0.05314978 |
| DALYs | Tajikistan | Both | Age-standardized | CKD-T1DM | Rate | 2021 | 0.108452669 | 0.264003932 | 0.04189875 |
| DALYs | Colombia | Both | Age-standardized | CKD-T1DM | Rate | 2021 | 0.107837135 | 0.184860497 | 0.05661255 |
| DALYs | Qatar | Both | Age-standardized | CKD-T1DM | Rate | 2021 | 0.10767503 | 0.20066097 | 0.0499537 |
| DALYs | Lebanon | Both | Age-standardized | CKD-T1DM | Rate | 2021 | 0.105843533 | 0.198432426 | 0.04865515 |
| DALYs | Russian Federation | Both | Age-standardized | CKD-T1DM | Rate | 2021 | 0.101674215 | 0.155492794 | 0.0639704 |
| DALYs | Honduras | Both | Age-standardized | CKD-T1DM | Rate | 2021 | 0.101265447 | 0.183188337 | 0.04701031 |
| DALYs | Poland | Both | Age-standardized | CKD-T1DM | Rate | 2021 | 0.09901972 | 0.17670491 | 0.05150637 |
| DALYs | Hungary | Both | Age-standardized | CKD-T1DM | Rate | 2021 | 0.098286589 | 0.335379839 | 0.02638152 |
| DALYs | Singapore | Both | Age-standardized | CKD-T1DM | Rate | 2021 | 0.095231117 | 0.167187422 | 0.0533813 |
| DALYs | Serbia | Both | Age-standardized | CKD-T1DM | Rate | 2021 | 0.094724775 | 0.237715996 | 0.04007257 |
| DALYs | Latvia | Both | Age-standardized | CKD-T1DM | Rate | 2021 | 0.092549069 | 0.241578768 | 0.03597006 |
| DALYs | Croatia | Both | Age-standardized | CKD-T1DM | Rate | 2021 | 0.08993794 | 0.275100136 | 0.02935635 |
| DALYs | Czechia | Both | Age-standardized | CKD-T1DM | Rate | 2021 | 0.088673841 | 0.293482137 | 0.02383489 |
| DALYs | Lithuania | Both | Age-standardized | CKD-T1DM | Rate | 2021 | 0.088039759 | 0.226578671 | 0.03537853 |
| DALYs | Romania | Both | Age-standardized | CKD-T1DM | Rate | 2021 | 0.087777204 | 0.293085373 | 0.023303 |
| DALYs | Ukraine | Both | Age-standardized | CKD-T1DM | Rate | 2021 | 0.08731494 | 0.214285325 | 0.03829647 |
| DALYs | Tunisia | Both | Age-standardized | CKD-T1DM | Rate | 2021 | 0.084812849 | 0.172407077 | 0.04092052 |
| DALYs | Kuwait | Both | Age-standardized | CKD-T1DM | Rate | 2021 | 0.084333838 | 0.160212903 | 0.04199453 |
| DALYs | Uruguay | Both | Age-standardized | CKD-T1DM | Rate | 2021 | 0.079511128 | 0.154868214 | 0.04112627 |
| DALYs | Slovenia | Both | Age-standardized | CKD-T1DM | Rate | 2021 | 0.079495727 | 0.315814394 | 0.0196725 |
| DALYs | Yemen | Both | Age-standardized | CKD-T1DM | Rate | 2021 | 0.072497893 | 0.169607587 | 0.03126638 |
| DALYs | Chile | Both | Age-standardized | CKD-T1DM | Rate | 2021 | 0.070889666 | 0.124776894 | 0.03821969 |
| DALYs | Italy | Both | Age-standardized | CKD-T1DM | Rate | 2021 | 0.070704072 | 0.134955991 | 0.03424049 |
| DALYs | Belarus | Both | Age-standardized | CKD-T1DM | Rate | 2021 | 0.068118547 | 0.214954269 | 0.02086643 |
| DALYs | Ireland | Both | Age-standardized | CKD-T1DM | Rate | 2021 | 0.067676175 | 0.250043208 | 0.01148845 |
| DALYs | Israel | Both | Age-standardized | CKD-T1DM | Rate | 2021 | 0.064729621 | 0.158354974 | 0.02635216 |
| DALYs | Malta | Both | Age-standardized | CKD-T1DM | Rate | 2021 | 0.061145031 | 0.190864091 | 0.02235998 |
| DALYs | Republic of Korea | Both | Age-standardized | CKD-T1DM | Rate | 2021 | 0.060239955 | 0.101288183 | 0.03353264 |
| DALYs | Japan | Both | Age-standardized | CKD-T1DM | Rate | 2021 | 0.058651754 | 0.085026336 | 0.03727032 |
| DALYs | Austria | Both | Age-standardized | CKD-T1DM | Rate | 2021 | 0.058062535 | 0.201121658 | 0.01764812 |
| DALYs | Netherlands | Both | Age-standardized | CKD-T1DM | Rate | 2021 | 0.051343868 | 0.177359791 | 0.01224279 |
| DALYs | Denmark | Both | Age-standardized | CKD-T1DM | Rate | 2021 | 0.048421843 | 0.202356885 | 0.0103493 |
| DALYs | Finland | Both | Age-standardized | CKD-T1DM | Rate | 2021 | 0.043166088 | 0.154409716 | 0.01166564 |
| DALYs | Greece | Both | Age-standardized | CKD-T1DM | Rate | 2021 | 0.041569475 | 0.105186503 | 0.01963595 |
| DALYs | Cyprus | Both | Age-standardized | CKD-T1DM | Rate | 2021 | 0.040712195 | 0.157236228 | 0.01043542 |
| DALYs | Canada | Both | Age-standardized | CKD-T1DM | Rate | 2021 | 0.039786424 | 0.139920125 | 0.01186898 |
| DALYs | United States of America | Both | Age-standardized | CKD-T1DM | Rate | 2021 | 0.039685398 | 0.06006918 | 0.02448196 |
| DALYs | Germany | Both | Age-standardized | CKD-T1DM | Rate | 2021 | 0.038953216 | 0.13184467 | 0.0128367 |
| DALYs | Belgium | Both | Age-standardized | CKD-T1DM | Rate | 2021 | 0.038165581 | 0.132718622 | 0.01237679 |
| DALYs | Monaco | Both | Age-standardized | CKD-T1DM | Rate | 2021 | 0.038030344 | 0.12488913 | 0.01362842 |
| DALYs | Spain | Both | Age-standardized | CKD-T1DM | Rate | 2021 | 0.037623889 | 0.1089643 | 0.01355963 |
| DALYs | Portugal | Both | Age-standardized | CKD-T1DM | Rate | 2021 | 0.036617823 | 0.09937704 | 0.01735241 |
| DALYs | Andorra | Both | Age-standardized | CKD-T1DM | Rate | 2021 | 0.035977174 | 0.116074147 | 0.01102805 |
| DALYs | Luxembourg | Both | Age-standardized | CKD-T1DM | Rate | 2021 | 0.035405794 | 0.122837401 | 0.01115856 |
| DALYs | United Kingdom | Both | Age-standardized | CKD-T1DM | Rate | 2021 | 0.034529496 | 0.056828722 | 0.02059538 |
| DALYs | Norway | Both | Age-standardized | CKD-T1DM | Rate | 2021 | 0.034328391 | 0.067309313 | 0.0168645 |
| DALYs | San Marino | Both | Age-standardized | CKD-T1DM | Rate | 2021 | 0.029859094 | 0.100306616 | 0.00805158 |
| DALYs | Sweden | Both | Age-standardized | CKD-T1DM | Rate | 2021 | 0.029065729 | 0.085135124 | 0.00915852 |
| DALYs | Switzerland | Both | Age-standardized | CKD-T1DM | Rate | 2021 | 0.028582433 | 0.088046631 | 0.00918139 |
| DALYs | New Zealand | Both | Age-standardized | CKD-T1DM | Rate | 2021 | 0.025440927 | 0.055933624 | 0.0118115 |
| DALYs | France | Both | Age-standardized | CKD-T1DM | Rate | 2021 | 0.023473962 | 0.084069843 | 0.00836052 |
| DALYs | Greenland | Both | Age-standardized | CKD-T1DM | Rate | 2021 | 0.023180919 | 0.066722554 | 0.00812725 |
| DALYs | Australia | Both | Age-standardized | CKD-T1DM | Rate | 2021 | 0.022779765 | 0.082918384 | 0.0061796 |
| DALYs | Iceland | Both | Age-standardized | CKD-T1DM | Rate | 2021 | 0.018712814 | 0.06051447 | 0.0055363 |

Appendix

**Appendix 19：Global disease burden distribution of Chronic kidney disease due to diabetes mellitus type 1 in children and adolescents from 1990 to 2021**

| **location** | **EAPC** | **LCI** | **UCI** | **EAPC_CI** |
| --- | --- | --- | --- | --- |
| Albania | 4.24 | 3.56 | 4.92 | 4.24(3.56 to 4.92) |
| Republic of Moldova | 3.93 | 2.46 | 5.41 | 3.93(2.46 to 5.41) |
| Bulgaria | 3.89 | 2.76 | 5.03 | 3.89(2.76 to 5.03) |
| Philippines | 3.57 | 3.1 | 4.04 | 3.57(3.1 to 4.04) |
| North Macedonia | 3.54 | 2.63 | 4.45 | 3.54(2.63 to 4.45) |
| Ukraine | 3.48 | 2.21 | 4.77 | 3.48(2.21 to 4.77) |
| El Salvador | 3.29 | 0.48 | 6.17 | 3.29(0.48 to 6.17) |
| Serbia | 3.22 | 2.76 | 3.69 | 3.22(2.76 to 3.69) |
| Bosnia and Herzegovina | 3.15 | 2.61 | 3.7 | 3.15(2.61 to 3.7) |
| Saudi Arabia | 3.11 | 2.21 | 4.02 | 3.11(2.21 to 4.02) |
| Hungary | 3.08 | 2.21 | 3.96 | 3.08(2.21 to 3.96) |
| Montenegro | 2.96 | 1.4 | 4.54 | 2.96(1.4 to 4.54) |
| Belarus | 2.92 | 1.95 | 3.9 | 2.92(1.95 to 3.9) |
| Mexico | 2.9 | 0.75 | 5.1 | 2.9(0.75 to 5.1) |
| Croatia | 2.85 | 2.31 | 3.39 | 2.85(2.31 to 3.39) |
| Czechia | 2.83 | 2.12 | 3.54 | 2.83(2.12 to 3.54) |
| Belize | 2.77 | 2.22 | 3.32 | 2.77(2.22 to 3.32) |
| Latvia | 2.73 | 1.86 | 3.6 | 2.73(1.86 to 3.6) |
| Slovenia | 2.72 | 2.13 | 3.32 | 2.72(2.13 to 3.32) |
| Grenada | 2.67 | 2.34 | 3 | 2.67(2.34 to 3) |
| Dominican Republic | 2.65 | 2.01 | 3.29 | 2.65(2.01 to 3.29) |
| Finland | 2.62 | 1.94 | 3.31 | 2.62(1.94 to 3.31) |
| Israel | 2.56 | 0.49 | 4.68 | 2.56(0.49 to 4.68) |
| Estonia | 2.55 | 1.84 | 3.25 | 2.55(1.84 to 3.25) |
| Morocco | 2.55 | 1.09 | 4.03 | 2.55(1.09 to 4.03) |
| Bahamas | 2.53 | 1.82 | 3.25 | 2.53(1.82 to 3.25) |
| Turkmenistan | 2.46 | 1.92 | 3 | 2.46(1.92 to 3) |
| Romania | 2.46 | 1.39 | 3.54 | 2.46(1.39 to 3.54) |
| Azerbaijan | 2.44 | 1.15 | 3.76 | 2.44(1.15 to 3.76) |
| Guatemala | 2.41 | 0.41 | 4.46 | 2.41(0.41 to 4.46) |
| Russian Federation | 2.37 | 1.3 | 3.46 | 2.37(1.3 to 3.46) |
| Saint Vincent and the Grenadines | 2.35 | 2.11 | 2.59 | 2.35(2.11 to 2.59) |
| Slovakia | 2.33 | 1.62 | 3.04 | 2.33(1.62 to 3.04) |
| Australia | 2.3 | 1.3 | 3.32 | 2.3(1.3 to 3.32) |
| Lithuania | 2.3 | 1.5 | 3.11 | 2.3(1.5 to 3.11) |
| Malta | 2.3 | 1.04 | 3.57 | 2.3(1.04 to 3.57) |
| Cyprus | 2.27 | 0.35 | 4.22 | 2.27(0.35 to 4.22) |
| United Arab Emirates | 2.27 | 1.96 | 2.58 | 2.27(1.96 to 2.58) |
| Guyana | 2.26 | 2 | 2.51 | 2.26(2 to 2.51) |
| Peru | 2.24 | 1.13 | 3.36 | 2.24(1.13 to 3.36) |
| Libya | 2.23 | 1.55 | 2.9 | 2.23(1.55 to 2.9) |
| Oman | 2.22 | 1.38 | 3.07 | 2.22(1.38 to 3.07) |
| Cook Islands | 2.21 | 1.78 | 2.65 | 2.21(1.78 to 2.65) |
| Uzbekistan | 2.2 | 1.78 | 2.62 | 2.2(1.78 to 2.62) |
| Saint Lucia | 2.19 | 0.81 | 3.59 | 2.19(0.81 to 3.59) |
| Kenya | 2.18 | -2.76 | 7.38 | 2.18(-2.76 to 7.38) |
| Mauritius | 2.17 | 0.71 | 3.66 | 2.17(0.71 to 3.66) |
| Ireland | 2.16 | -0.88 | 5.29 | 2.16(-0.88 to 5.29) |
| Indonesia | 2.14 | 0.7 | 3.6 | 2.14(0.7 to 3.6) |
| Viet Nam | 2.13 | 1.64 | 2.61 | 2.13(1.64 to 2.61) |
| Guam | 2.1 | 1.84 | 2.37 | 2.1(1.84 to 2.37) |
| Georgia | 2.08 | 1.49 | 2.66 | 2.08(1.49 to 2.66) |
| Suriname | 2.08 | 1.48 | 2.69 | 2.08(1.48 to 2.69) |
| Paraguay | 2.08 | 0.81 | 3.38 | 2.08(0.81 to 3.38) |
| Tokelau | 2.08 | 1.21 | 2.97 | 2.08(1.21 to 2.97) |
| Andorra | 2.07 | 0.29 | 3.88 | 2.07(0.29 to 3.88) |
| Palau | 2.07 | 1.75 | 2.39 | 2.07(1.75 to 2.39) |
| Northern Mariana Islands | 2.06 | 0.97 | 3.16 | 2.06(0.97 to 3.16) |
| Luxembourg | 2.05 | 0.44 | 3.69 | 2.05(0.44 to 3.69) |
| Trinidad and Tobago | 2.04 | 1.28 | 2.8 | 2.04(1.28 to 2.8) |
| San Marino | 2.03 | 0.38 | 3.71 | 2.03(0.38 to 3.71) |
| Monaco | 2.02 | 0.28 | 3.79 | 2.02(0.28 to 3.79) |
| Saint Kitts and Nevis | 2.02 | 1.13 | 2.93 | 2.02(1.13 to 2.93) |
| Armenia | 2.01 | 1.61 | 2.41 | 2.01(1.61 to 2.41) |
| Bermuda | 2.01 | 0.75 | 3.27 | 2.01(0.75 to 3.27) |
| Nepal | 1.99 | 0.56 | 3.45 | 1.99(0.56 to 3.45) |
| Micronesia (Federated States of) | 1.96 | 1.44 | 2.49 | 1.96(1.44 to 2.49) |
| Austria | 1.96 | 0.32 | 3.63 | 1.96(0.32 to 3.63) |
| Bahrain | 1.92 | 1.27 | 2.57 | 1.92(1.27 to 2.57) |
| United States Virgin Islands | 1.92 | -0.13 | 4.01 | 1.92(-0.13 to 4.01) |
| Tonga | 1.91 | 1.4 | 2.43 | 1.91(1.4 to 2.43) |
| Egypt | 1.88 | 1.01 | 2.77 | 1.88(1.01 to 2.77) |
| T眉rkiye | 1.88 | 1.56 | 2.2 | 1.88(1.56 to 2.2) |
| Bhutan | 1.88 | 0.21 | 3.58 | 1.88(0.21 to 3.58) |
| Tuvalu | 1.86 | 1.23 | 2.49 | 1.86(1.23 to 2.49) |
| Antigua and Barbuda | 1.85 | 0.17 | 3.56 | 1.85(0.17 to 3.56) |
| Ecuador | 1.83 | 0.58 | 3.09 | 1.83(0.58 to 3.09) |
| Pakistan | 1.82 | 0.9 | 2.75 | 1.82(0.9 to 2.75) |
| Samoa | 1.81 | 1.29 | 2.33 | 1.81(1.29 to 2.33) |
| American Samoa | 1.77 | 1.25 | 2.29 | 1.77(1.25 to 2.29) |
| Nicaragua | 1.75 | -2.16 | 5.81 | 1.75(-2.16 to 5.81) |
| Tunisia | 1.73 | 1 | 2.46 | 1.73(1 to 2.46) |
| Vanuatu | 1.71 | 1.04 | 2.39 | 1.71(1.04 to 2.39) |
| Lebanon | 1.7 | 1.16 | 2.24 | 1.7(1.16 to 2.24) |
| Kiribati | 1.66 | 1.08 | 2.25 | 1.66(1.08 to 2.25) |
| Netherlands | 1.66 | -0.01 | 3.35 | 1.66(-0.01 to 3.35) |
| Cuba | 1.65 | 0.97 | 2.34 | 1.65(0.97 to 2.34) |
| Tajikistan | 1.64 | 1.24 | 2.05 | 1.64(1.24 to 2.05) |
| Belgium | 1.64 | 0.06 | 3.24 | 1.64(0.06 to 3.24) |
| Barbados | 1.61 | 0.66 | 2.56 | 1.61(0.66 to 2.56) |
| Bangladesh | 1.61 | -0.2 | 3.46 | 1.61(-0.2 to 3.46) |
| Gabon | 1.61 | -1.77 | 5.11 | 1.61(-1.77 to 5.11) |
| Niue | 1.61 | 1.27 | 1.95 | 1.61(1.27 to 1.95) |
| Democratic People's Republic of Korea | 1.59 | 0.5 | 2.69 | 1.59(0.5 to 2.69) |
| Uruguay | 1.59 | 0.59 | 2.6 | 1.59(0.59 to 2.6) |
| Lesotho | 1.59 | 0.1 | 3.1 | 1.59(0.1 to 3.1) |
| Iraq | 1.58 | 0.99 | 2.17 | 1.58(0.99 to 2.17) |
| Afghanistan | 1.58 | -0.25 | 3.45 | 1.58(-0.25 to 3.45) |
| Mozambique | 1.58 | -4.07 | 7.55 | 1.58(-4.07 to 7.55) |
| Panama | 1.57 | -1.05 | 4.25 | 1.57(-1.05 to 4.25) |
| Venezuela (Bolivarian Republic of) | 1.56 | -0.58 | 3.75 | 1.56(-0.58 to 3.75) |
| Qatar | 1.56 | 0.67 | 2.46 | 1.56(0.67 to 2.46) |
| Nauru | 1.56 | 1.27 | 1.85 | 1.56(1.27 to 1.85) |
| Switzerland | 1.52 | 0.79 | 2.25 | 1.52(0.79 to 2.25) |
| Jamaica | 1.52 | 0.94 | 2.1 | 1.52(0.94 to 2.1) |
| France | 1.51 | 0.28 | 2.76 | 1.51(0.28 to 2.76) |
| Taiwan (Province of China) | 1.5 | 0.34 | 2.68 | 1.5(0.34 to 2.68) |
| Denmark | 1.49 | -0.23 | 3.25 | 1.49(-0.23 to 3.25) |
| Canada | 1.49 | -1.08 | 4.13 | 1.49(-1.08 to 4.13) |
| Dominica | 1.48 | 0.73 | 2.24 | 1.48(0.73 to 2.24) |
| Sao Tome and Principe | 1.46 | 0.15 | 2.79 | 1.46(0.15 to 2.79) |
| Iceland | 1.39 | -0.43 | 3.23 | 1.39(-0.43 to 3.23) |
| Djibouti | 1.38 | -3.89 | 6.94 | 1.38(-3.89 to 6.94) |
| Puerto Rico | 1.38 | 1.05 | 1.71 | 1.38(1.05 to 1.71) |
| Botswana | 1.36 | -0.93 | 3.7 | 1.36(-0.93 to 3.7) |
| Marshall Islands | 1.35 | 0.57 | 2.13 | 1.35(0.57 to 2.13) |
| Germany | 1.35 | -0.53 | 3.26 | 1.35(-0.53 to 3.26) |
| Malawi | 1.35 | -3.89 | 6.88 | 1.35(-3.89 to 6.88) |
| Sudan | 1.34 | 0.99 | 1.69 | 1.34(0.99 to 1.69) |
| Greece | 1.33 | -0.36 | 3.06 | 1.33(-0.36 to 3.06) |
| Algeria | 1.33 | 0.58 | 2.09 | 1.33(0.58 to 2.09) |
| Portugal | 1.31 | -0.41 | 3.06 | 1.31(-0.41 to 3.06) |
| Cabo Verde | 1.31 | -1.44 | 4.15 | 1.31(-1.44 to 4.15) |
| Mongolia | 1.29 | 0.82 | 1.77 | 1.29(0.82 to 1.77) |
| Equatorial Guinea | 1.26 | -2 | 4.62 | 1.26(-2 to 4.62) |
| Seychelles | 1.22 | 0.59 | 1.85 | 1.22(0.59 to 1.85) |
| Papua New Guinea | 1.21 | -0.06 | 2.51 | 1.21(-0.06 to 2.51) |
| Malaysia | 1.2 | 0.93 | 1.46 | 1.2(0.93 to 1.46) |
| Ghana | 1.2 | -0.26 | 2.69 | 1.2(-0.26 to 2.69) |
| Gambia | 1.2 | -1.32 | 3.77 | 1.2(-1.32 to 3.77) |
| Fiji | 1.19 | 0.9 | 1.47 | 1.19(0.9 to 1.47) |
| South Sudan | 1.19 | -3.38 | 5.97 | 1.19(-3.38 to 5.97) |
| Chile | 1.16 | -0.22 | 2.55 | 1.16(-0.22 to 2.55) |
| Palestine | 1.16 | 0.85 | 1.46 | 1.16(0.85 to 1.46) |
| Sri Lanka | 1.13 | 0.01 | 2.27 | 1.13(0.01 to 2.27) |
| Italy | 1.13 | -0.09 | 2.35 | 1.13(-0.09 to 2.35) |
| Myanmar | 1.12 | 0.98 | 1.26 | 1.12(0.98 to 1.26) |
| Norway | 1.12 | 0.28 | 1.96 | 1.12(0.28 to 1.96) |
| Syrian Arab Republic | 1.12 | 1.02 | 1.22 | 1.12(1.02 to 1.22) |
| Somalia | 1.12 | -2.59 | 4.97 | 1.12(-2.59 to 4.97) |
| Kuwait | 1.11 | 0.63 | 1.59 | 1.11(0.63 to 1.59) |
| Uganda | 1.11 | -4.29 | 6.82 | 1.11(-4.29 to 6.82) |
| Haiti | 1.1 | 0.51 | 1.69 | 1.1(0.51 to 1.69) |
| Jordan | 1.1 | -0.03 | 2.24 | 1.1(-0.03 to 2.24) |
| Yemen | 1.1 | 0.59 | 1.61 | 1.1(0.59 to 1.61) |
| Brazil | 1.09 | 0.25 | 1.94 | 1.09(0.25 to 1.94) |
| Democratic Republic of the Congo | 1.09 | -1.75 | 4 | 1.09(-1.75 to 4) |
| Kazakhstan | 1.05 | 0.39 | 1.72 | 1.05(0.39 to 1.72) |
| Central African Republic | 1.05 | -1.92 | 4.1 | 1.05(-1.92 to 4.1) |
| Liberia | 1.04 | -0.83 | 2.94 | 1.04(-0.83 to 2.94) |
| Guinea | 1.02 | -1.09 | 3.18 | 1.02(-1.09 to 3.18) |
| Eswatini | 1 | -0.8 | 2.83 | 1(-0.8 to 2.83) |
| Eritrea | 0.99 | -3.6 | 5.8 | 0.99(-3.6 to 5.8) |
| Timor-Leste | 0.98 | 0.27 | 1.69 | 0.98(0.27 to 1.69) |
| Honduras | 0.98 | -0.14 | 2.13 | 0.98(-0.14 to 2.13) |
| Congo | 0.96 | -1.17 | 3.15 | 0.96(-1.17 to 3.15) |
| Iran (Islamic Republic of) | 0.92 | 0.69 | 1.15 | 0.92(0.69 to 1.15) |
| Kyrgyzstan | 0.9 | 0.31 | 1.51 | 0.9(0.31 to 1.51) |
| Bolivia (Plurinational State of) | 0.89 | -1.26 | 3.08 | 0.89(-1.26 to 3.08) |
| Madagascar | 0.83 | -3.44 | 5.28 | 0.83(-3.44 to 5.28) |
| Zimbabwe | 0.82 | -0.43 | 2.1 | 0.82(-0.43 to 2.1) |
| India | 0.8 | -0.92 | 2.56 | 0.8(-0.92 to 2.56) |
| Angola | 0.8 | -2.12 | 3.81 | 0.8(-2.12 to 3.81) |
| Cambodia | 0.79 | 0.58 | 1 | 0.79(0.58 to 1) |
| Argentina | 0.79 | -0.87 | 2.47 | 0.79(-0.87 to 2.47) |
| Costa Rica | 0.79 | -1.74 | 3.39 | 0.79(-1.74 to 3.39) |
| Togo | 0.79 | -2.05 | 3.7 | 0.79(-2.05 to 3.7) |
| Guinea-Bissau | 0.78 | -1.45 | 3.06 | 0.78(-1.45 to 3.06) |
| Comoros | 0.72 | -2.75 | 4.32 | 0.72(-2.75 to 4.32) |
| Chad | 0.72 | -2.15 | 3.66 | 0.72(-2.15 to 3.66) |
| Zambia | 0.71 | -3.38 | 4.99 | 0.71(-3.38 to 4.99) |
| Sierra Leone | 0.71 | -1.5 | 2.97 | 0.71(-1.5 to 2.97) |
| Brunei Darussalam | 0.69 | -1.67 | 3.1 | 0.69(-1.67 to 3.1) |
| Lao People's Democratic Republic | 0.68 | 0.59 | 0.77 | 0.68(0.59 to 0.77) |
| Greenland | 0.68 | -1.71 | 3.12 | 0.68(-1.71 to 3.12) |
| Nigeria | 0.68 | -1.55 | 2.96 | 0.68(-1.55 to 2.96) |
| C么te d'Ivoire | 0.64 | -1.85 | 3.2 | 0.64(-1.85 to 3.2) |
| Benin | 0.61 | -1.93 | 3.22 | 0.61(-1.93 to 3.22) |
| New Zealand | 0.59 | -0.12 | 1.32 | 0.59(-0.12 to 1.32) |
| Mauritania | 0.58 | -1.48 | 2.69 | 0.58(-1.48 to 2.69) |
| Poland | 0.57 | -0.09 | 1.23 | 0.57(-0.09 to 1.23) |
| United Kingdom | 0.57 | -0.73 | 1.88 | 0.57(-0.73 to 1.88) |
| Cameroon | 0.54 | -1.83 | 2.97 | 0.54(-1.83 to 2.97) |
| Namibia | 0.52 | -1.58 | 2.66 | 0.52(-1.58 to 2.66) |
| China | 0.51 | -0.37 | 1.41 | 0.51(-0.37 to 1.41) |
| Burkina Faso | 0.43 | -2.18 | 3.1 | 0.43(-2.18 to 3.1) |
| South Africa | 0.42 | -0.45 | 1.3 | 0.42(-0.45 to 1.3) |
| Niger | 0.4 | -2.08 | 2.95 | 0.4(-2.08 to 2.95) |
| Senegal | 0.36 | -2.25 | 3.05 | 0.36(-2.25 to 3.05) |
| Sweden | 0.35 | -1.08 | 1.8 | 0.35(-1.08 to 1.8) |
| Solomon Islands | 0.31 | -0.29 | 0.93 | 0.31(-0.29 to 0.93) |
| Spain | 0.31 | -1.31 | 1.97 | 0.31(-1.31 to 1.97) |
| United Republic of Tanzania | 0.31 | -3.78 | 4.57 | 0.31(-3.78 to 4.57) |
| Japan | 0.23 | -1.28 | 1.77 | 0.23(-1.28 to 1.77) |
| Ethiopia | 0.21 | -3.45 | 4.01 | 0.21(-3.45 to 4.01) |
| Mali | 0.09 | -1.75 | 1.96 | 0.09(-1.75 to 1.96) |
| Rwanda | 0.05 | -4.04 | 4.32 | 0.05(-4.04 to 4.32) |
| Burundi | 0.03 | -4.1 | 4.34 | 0.03(-4.1 to 4.34) |
| Colombia | 0 | -2.53 | 2.59 | 0(-2.53 to 2.59) |
| Maldives | -0.01 | -0.24 | 0.23 | -0.01(-0.24 to 0.23) |
| Thailand | -0.06 | -1.6 | 1.5 | -0.06(-1.6 to 1.5) |
| Singapore | -0.3 | -1.63 | 1.05 | -0.3(-1.63 to 1.05) |
| Republic of Korea | -1.26 | -3.52 | 1.05 | -1.26(-3.52 to 1.05) |
| United States of America | -1.28 | -3.95 | 1.47 | -1.28(-3.95 to 1.47) |

Appendix

**Appendix 20：Global disease burden distribution of Chronic kidney disease due to diabetes mellitus type 1 in children and adolescents from 1990 to 2021**

| **location** | **EAPC** | **LCI** | **UCI** | **EAPC_CI** |
| --- | --- | --- | --- | --- |
| Ireland | 3.29 | 2.7 | 3.89 | 3.29(2.7 to 3.89) |
| Israel | 3.13 | 2.71 | 3.55 | 3.13(2.71 to 3.55) |
| Republic of Moldova | 2.7 | 2.22 | 3.18 | 2.7(2.22 to 3.18) |
| Russian Federation | 2.6 | 1.83 | 3.37 | 2.6(1.83 to 3.37) |
| Albania | 2.58 | 1.94 | 3.23 | 2.58(1.94 to 3.23) |
| Andorra | 2.52 | 2.18 | 2.87 | 2.52(2.18 to 2.87) |
| Cyprus | 2.52 | 1.9 | 3.15 | 2.52(1.9 to 3.15) |
| Monaco | 2.49 | 1.82 | 3.17 | 2.49(1.82 to 3.17) |
| San Marino | 2.44 | 1.86 | 3.03 | 2.44(1.86 to 3.03) |
| Austria | 2.42 | 1.74 | 3.11 | 2.42(1.74 to 3.11) |
| Luxembourg | 2.28 | 1.69 | 2.88 | 2.28(1.69 to 2.88) |
| Hungary | 2.28 | 1.66 | 2.91 | 2.28(1.66 to 2.91) |
| Ukraine | 2.28 | 1.44 | 3.13 | 2.28(1.44 to 3.13) |
| Norway | 2.24 | 1.3 | 3.19 | 2.24(1.3 to 3.19) |
| Sweden | 2.2 | 1.41 | 2.98 | 2.2(1.41 to 2.98) |
| Belarus | 2.14 | 1.43 | 2.86 | 2.14(1.43 to 2.86) |
| Denmark | 2.11 | 1.75 | 2.47 | 2.11(1.75 to 2.47) |
| Iceland | 2.06 | 1.35 | 2.79 | 2.06(1.35 to 2.79) |
| Spain | 2.06 | 1.31 | 2.8 | 2.06(1.31 to 2.8) |
| Netherlands | 2.06 | 1.2 | 2.93 | 2.06(1.2 to 2.93) |
| Croatia | 2.06 | 1.15 | 2.98 | 2.06(1.15 to 2.98) |
| Belgium | 2.05 | 1.64 | 2.45 | 2.05(1.64 to 2.45) |
| Malta | 2.04 | 1.41 | 2.68 | 2.04(1.41 to 2.68) |
| Czechia | 1.96 | 1.16 | 2.76 | 1.96(1.16 to 2.76) |
| Bosnia and Herzegovina | 1.95 | 1.14 | 2.77 | 1.95(1.14 to 2.77) |
| North Macedonia | 1.95 | 0.98 | 2.93 | 1.95(0.98 to 2.93) |
| Switzerland | 1.93 | 1.07 | 2.81 | 1.93(1.07 to 2.81) |
| Italy | 1.93 | 1.04 | 2.82 | 1.93(1.04 to 2.82) |
| Germany | 1.92 | 1.2 | 2.64 | 1.92(1.2 to 2.64) |
| Romania | 1.89 | 1.04 | 2.76 | 1.89(1.04 to 2.76) |
| Australia | 1.88 | 0.84 | 2.93 | 1.88(0.84 to 2.93) |
| Armenia | 1.87 | 1.06 | 2.68 | 1.87(1.06 to 2.68) |
| Finland | 1.85 | 1 | 2.7 | 1.85(1 to 2.7) |
| Canada | 1.84 | 1.1 | 2.59 | 1.84(1.1 to 2.59) |
| Slovenia | 1.83 | 1.1 | 2.57 | 1.83(1.1 to 2.57) |
| Philippines | 1.83 | 0.89 | 2.77 | 1.83(0.89 to 2.77) |
| Serbia | 1.82 | 1.59 | 2.04 | 1.82(1.59 to 2.04) |
| Uzbekistan | 1.82 | 1.18 | 2.47 | 1.82(1.18 to 2.47) |
| Montenegro | 1.82 | 1.11 | 2.52 | 1.82(1.11 to 2.52) |
| Portugal | 1.81 | 1.52 | 2.11 | 1.81(1.52 to 2.11) |
| Bulgaria | 1.76 | 1.1 | 2.43 | 1.76(1.1 to 2.43) |
| Greece | 1.62 | 1.11 | 2.13 | 1.62(1.11 to 2.13) |
| France | 1.58 | 1.01 | 2.15 | 1.58(1.01 to 2.15) |
| Slovakia | 1.56 | 0.89 | 2.24 | 1.56(0.89 to 2.24) |
| Kenya | 1.56 | 0.75 | 2.38 | 1.56(0.75 to 2.38) |
| Guatemala | 1.53 | 0.85 | 2.21 | 1.53(0.85 to 2.21) |
| Lithuania | 1.52 | 0.76 | 2.29 | 1.52(0.76 to 2.29) |
| Latvia | 1.51 | 0.75 | 2.27 | 1.51(0.75 to 2.27) |
| Tajikistan | 1.46 | 0.81 | 2.11 | 1.46(0.81 to 2.11) |
| Georgia | 1.46 | 0.8 | 2.13 | 1.46(0.8 to 2.13) |
| Brazil | 1.46 | 0.65 | 2.28 | 1.46(0.65 to 2.28) |
| Estonia | 1.46 | 0.64 | 2.29 | 1.46(0.64 to 2.29) |
| Indonesia | 1.46 | 0.14 | 2.79 | 1.46(0.14 to 2.79) |
| Azerbaijan | 1.45 | 1.25 | 1.66 | 1.45(1.25 to 1.66) |
| Paraguay | 1.37 | 0.52 | 2.22 | 1.37(0.52 to 2.22) |
| Republic of Korea | -1.29 | -1.93 | -0.65 | -1.29(-1.93 to -0.65) |
| Argentina | 1.29 | 0.7 | 1.88 | 1.29(0.7 to 1.88) |
| Poland | 1.29 | 0.15 | 2.45 | 1.29(0.15 to 2.45) |
| Uruguay | 1.25 | 0.68 | 1.83 | 1.25(0.68 to 1.83) |
| Kyrgyzstan | 1.2 | 0.67 | 1.74 | 1.2(0.67 to 1.74) |
| United Republic of Tanzania | 1.18 | 1.14 | 1.21 | 1.18(1.14 to 1.21) |
| Malawi | 1.17 | 0.61 | 1.73 | 1.17(0.61 to 1.73) |
| Rwanda | 1.13 | 1.04 | 1.23 | 1.13(1.04 to 1.23) |
| Ethiopia | 1.13 | 0.84 | 1.42 | 1.13(0.84 to 1.42) |
| Oman | 1.13 | 0.02 | 2.26 | 1.13(0.02 to 2.26) |
| Turkmenistan | 1.1 | 0.33 | 1.87 | 1.1(0.33 to 1.87) |
| Viet Nam | 1.09 | -0.39 | 2.59 | 1.09(-0.39 to 2.59) |
| El Salvador | 1.06 | 0.62 | 1.51 | 1.06(0.62 to 1.51) |
| Yemen | 1.03 | 0.29 | 1.78 | 1.03(0.29 to 1.78) |
| Kazakhstan | 0.98 | 0.39 | 1.58 | 0.98(0.39 to 1.58) |
| T眉rkiye | 0.97 | 0.37 | 1.57 | 0.97(0.37 to 1.57) |
| Bhutan | 0.97 | 0.16 | 1.77 | 0.97(0.16 to 1.77) |
| Palestine | 0.9 | 0.3 | 1.51 | 0.9(0.3 to 1.51) |
| Lesotho | 0.88 | 0.51 | 1.25 | 0.88(0.51 to 1.25) |
| Saudi Arabia | 0.88 | -0.15 | 1.91 | 0.88(-0.15 to 1.91) |
| Somalia | 0.87 | 0.81 | 0.94 | 0.87(0.81 to 0.94) |
| Panama | 0.87 | 0.48 | 1.26 | 0.87(0.48 to 1.26) |
| Egypt | 0.85 | 0.34 | 1.36 | 0.85(0.34 to 1.36) |
| United Arab Emirates | 0.84 | 0.54 | 1.13 | 0.84(0.54 to 1.13) |
| Pakistan | 0.82 | -0.51 | 2.17 | 0.82(-0.51 to 2.17) |
| Nigeria | 0.82 | 0.36 | 1.29 | 0.82(0.36 to 1.29) |
| Syrian Arab Republic | 0.82 | 0.21 | 1.43 | 0.82(0.21 to 1.43) |
| Taiwan (Province of China) | 0.82 | -0.18 | 1.84 | 0.82(-0.18 to 1.84) |
| Djibouti | 0.81 | 0.54 | 1.07 | 0.81(0.54 to 1.07) |
| Eswatini | 0.81 | 0.46 | 1.15 | 0.81(0.46 to 1.15) |
| Burundi | 0.8 | 0.64 | 0.97 | 0.8(0.64 to 0.97) |
| Uganda | 0.79 | 0.71 | 0.87 | 0.79(0.71 to 0.87) |
| Madagascar | 0.78 | 0.55 | 1.01 | 0.78(0.55 to 1.01) |
| Botswana | 0.78 | 0.3 | 1.26 | 0.78(0.3 to 1.26) |
| Guam | 0.78 | 0.29 | 1.26 | 0.78(0.29 to 1.26) |
| Greenland | 0.78 | -0.1 | 1.67 | 0.78(-0.1 to 1.67) |
| South Sudan | 0.77 | 0.43 | 1.11 | 0.77(0.43 to 1.11) |
| Chile | 0.76 | 0.03 | 1.49 | 0.76(0.03 to 1.49) |
| Kuwait | 0.75 | 0.48 | 1.02 | 0.75(0.48 to 1.02) |
| Zimbabwe | 0.73 | 0.55 | 0.9 | 0.73(0.55 to 0.9) |
| South Africa | 0.72 | 0.56 | 0.88 | 0.72(0.56 to 0.88) |
| Sudan | 0.72 | 0.02 | 1.43 | 0.72(0.02 to 1.43) |
| Peru | 0.71 | 0.14 | 1.28 | 0.71(0.14 to 1.28) |
| Mexico | 0.7 | 0.43 | 0.97 | 0.7(0.43 to 0.97) |
| Namibia | 0.7 | 0.41 | 0.99 | 0.7(0.41 to 0.99) |
| Sri Lanka | 0.7 | -0.2 | 1.61 | 0.7(-0.2 to 1.61) |
| Eritrea | 0.69 | 0.42 | 0.97 | 0.69(0.42 to 0.97) |
| Northern Mariana Islands | 0.69 | 0.34 | 1.04 | 0.69(0.34 to 1.04) |
| Bahrain | 0.67 | -0.2 | 1.55 | 0.67(-0.2 to 1.55) |
| India | 0.67 | -0.1 | 1.46 | 0.67(-0.1 to 1.46) |
| Mozambique | 0.66 | 0.58 | 0.75 | 0.66(0.58 to 0.75) |
| Iran (Islamic Republic of) | 0.66 | 0.34 | 0.98 | 0.66(0.34 to 0.98) |
| Mongolia | 0.66 | 0.2 | 1.11 | 0.66(0.2 to 1.11) |
| Libya | 0.65 | -0.11 | 1.41 | 0.65(-0.11 to 1.41) |
| Solomon Islands | 0.64 | 0.12 | 1.16 | 0.64(0.12 to 1.16) |
| Malaysia | 0.63 | -0.54 | 1.82 | 0.63(-0.54 to 1.82) |
| Dominican Republic | 0.63 | 0.34 | 0.92 | 0.63(0.34 to 0.92) |
| Myanmar | 0.61 | -0.76 | 2 | 0.61(-0.76 to 2) |
| Haiti | 0.61 | -0.21 | 1.44 | 0.61(-0.21 to 1.44) |
| Comoros | 0.59 | 0.54 | 0.64 | 0.59(0.54 to 0.64) |
| Ecuador | 0.59 | 0.21 | 0.96 | 0.59(0.21 to 0.96) |
| Qatar | 0.59 | -0.14 | 1.33 | 0.59(-0.14 to 1.33) |
| Bangladesh | 0.57 | -0.2 | 1.35 | 0.57(-0.2 to 1.35) |
| Cambodia | 0.56 | -0.69 | 1.83 | 0.56(-0.69 to 1.83) |
| Dominica | 0.56 | 0.33 | 0.8 | 0.56(0.33 to 0.8) |
| Papua New Guinea | 0.55 | 0.49 | 0.61 | 0.55(0.49 to 0.61) |
| Belize | 0.55 | 0.41 | 0.69 | 0.55(0.41 to 0.69) |
| Lebanon | 0.53 | -0.46 | 1.52 | 0.53(-0.46 to 1.52) |
| Kiribati | 0.51 | -0.07 | 1.09 | 0.51(-0.07 to 1.09) |
| Fiji | 0.51 | -0.04 | 1.07 | 0.51(-0.04 to 1.07) |
| Grenada | 0.5 | 0.26 | 0.74 | 0.5(0.26 to 0.74) |
| Bolivia (Plurinational State of) | 0.49 | -0.26 | 1.25 | 0.49(-0.26 to 1.25) |
| Mauritania | 0.49 | -0.06 | 1.04 | 0.49(-0.06 to 1.04) |
| United States Virgin Islands | 0.48 | 0.36 | 0.59 | 0.48(0.36 to 0.59) |
| Mauritius | 0.48 | -0.27 | 1.24 | 0.48(-0.27 to 1.24) |
| Tonga | 0.48 | 0.08 | 0.88 | 0.48(0.08 to 0.88) |
| Timor-Leste | 0.46 | -0.92 | 1.85 | 0.46(-0.92 to 1.85) |
| Tokelau | 0.46 | -0.39 | 1.32 | 0.46(-0.39 to 1.32) |
| Antigua and Barbuda | 0.46 | 0.37 | 0.56 | 0.46(0.37 to 0.56) |
| Zambia | 0.46 | 0.3 | 0.61 | 0.46(0.3 to 0.61) |
| Lao People's Democratic Republic | 0.45 | -0.95 | 1.88 | 0.45(-0.95 to 1.88) |
| Guyana | 0.45 | 0.04 | 0.86 | 0.45(0.04 to 0.86) |
| Puerto Rico | 0.45 | -0.03 | 0.93 | 0.45(-0.03 to 0.93) |
| Palau | 0.44 | 0.16 | 0.72 | 0.44(0.16 to 0.72) |
| Maldives | 0.43 | -0.74 | 1.61 | 0.43(-0.74 to 1.61) |
| Vanuatu | 0.43 | -0.3 | 1.16 | 0.43(-0.3 to 1.16) |
| Nicaragua | 0.43 | -0.22 | 1.1 | 0.43(-0.22 to 1.1) |
| Ghana | 0.43 | -0.14 | 1 | 0.43(-0.14 to 1) |
| Equatorial Guinea | 0.42 | 0.2 | 0.64 | 0.42(0.2 to 0.64) |
| Saint Lucia | 0.42 | 0.15 | 0.69 | 0.42(0.15 to 0.69) |
| Nauru | 0.4 | -0.1 | 0.9 | 0.4(-0.1 to 0.9) |
| Saint Vincent and the Grenadines | 0.4 | 0.08 | 0.72 | 0.4(0.08 to 0.72) |
| American Samoa | 0.39 | 0.19 | 0.58 | 0.39(0.19 to 0.58) |
| Democratic People's Republic of Korea | 0.36 | -0.38 | 1.12 | 0.36(-0.38 to 1.12) |
| Suriname | 0.35 | 0.27 | 0.43 | 0.35(0.27 to 0.43) |
| Bahamas | 0.35 | 0.1 | 0.59 | 0.35(0.1 to 0.59) |
| Togo | 0.35 | -0.05 | 0.77 | 0.35(-0.05 to 0.77) |
| Tunisia | 0.34 | -0.4 | 1.09 | 0.34(-0.4 to 1.09) |
| Cameroon | 0.34 | 0.01 | 0.68 | 0.34(0.01 to 0.68) |
| Jordan | -0.33 | -1.67 | 1.03 | -0.33(-1.67 to 1.03) |
| Gabon | 0.33 | 0.27 | 0.4 | 0.33(0.27 to 0.4) |
| Guinea | 0.33 | -0.21 | 0.88 | 0.33(-0.21 to 0.88) |
| Colombia | 0.33 | 0.16 | 0.49 | 0.33(0.16 to 0.49) |
| Morocco | 0.32 | -0.41 | 1.05 | 0.32(-0.41 to 1.05) |
| Tuvalu | 0.32 | -0.34 | 0.98 | 0.32(-0.34 to 0.98) |
| Chad | 0.32 | -0.16 | 0.8 | 0.32(-0.16 to 0.8) |
| Micronesia (Federated States of) | 0.32 | -0.1 | 0.74 | 0.32(-0.1 to 0.74) |
| Saint Kitts and Nevis | 0.32 | 0.09 | 0.56 | 0.32(0.09 to 0.56) |
| Trinidad and Tobago | 0.3 | 0.12 | 0.47 | 0.3(0.12 to 0.47) |
| Gambia | 0.3 | -0.11 | 0.71 | 0.3(-0.11 to 0.71) |
| Japan | 0.29 | -0.72 | 1.31 | 0.29(-0.72 to 1.31) |
| Brunei Darussalam | 0.29 | -0.1 | 0.68 | 0.29(-0.1 to 0.68) |
| Afghanistan | 0.26 | -0.98 | 1.51 | 0.26(-0.98 to 1.51) |
| Venezuela (Bolivarian Republic of) | 0.26 | 0.11 | 0.42 | 0.26(0.11 to 0.42) |
| Iraq | 0.25 | -0.29 | 0.79 | 0.25(-0.29 to 0.79) |
| Algeria | 0.25 | -0.24 | 0.73 | 0.25(-0.24 to 0.73) |
| Honduras | 0.24 | -0.22 | 0.71 | 0.24(-0.22 to 0.71) |
| Central African Republic | 0.24 | 0.16 | 0.33 | 0.24(0.16 to 0.33) |
| Angola | 0.24 | 0 | 0.49 | 0.24(0 to 0.49) |
| C么te d'Ivoire | 0.21 | -0.13 | 0.56 | 0.21(-0.13 to 0.56) |
| Seychelles | 0.2 | -0.77 | 1.19 | 0.2(-0.77 to 1.19) |
| New Zealand | 0.2 | -0.47 | 0.88 | 0.2(-0.47 to 0.88) |
| Samoa | 0.2 | -0.17 | 0.58 | 0.2(-0.17 to 0.58) |
| Sierra Leone | 0.19 | -0.29 | 0.67 | 0.19(-0.29 to 0.67) |
| Burkina Faso | 0.19 | -0.28 | 0.67 | 0.19(-0.28 to 0.67) |
| Congo | 0.18 | -0.1 | 0.46 | 0.18(-0.1 to 0.46) |
| Senegal | 0.17 | -0.29 | 0.63 | 0.17(-0.29 to 0.63) |
| Barbados | 0.17 | -0.21 | 0.55 | 0.17(-0.21 to 0.55) |
| Costa Rica | 0.17 | 0.1 | 0.24 | 0.17(0.1 to 0.24) |
| United States of America | -0.16 | -0.88 | 0.57 | -0.16(-0.88 to 0.57) |
| Jamaica | 0.16 | -0.45 | 0.77 | 0.16(-0.45 to 0.77) |
| Cook Islands | 0.16 | -0.26 | 0.58 | 0.16(-0.26 to 0.58) |
| Thailand | 0.15 | -1.17 | 1.49 | 0.15(-1.17 to 1.49) |
| Democratic Republic of the Congo | 0.15 | -0.02 | 0.31 | 0.15(-0.02 to 0.31) |
| Niger | 0.14 | -0.33 | 0.62 | 0.14(-0.33 to 0.62) |
| China | 0.13 | -0.62 | 0.9 | 0.13(-0.62 to 0.9) |
| Guinea-Bissau | 0.13 | -0.19 | 0.46 | 0.13(-0.19 to 0.46) |
| Niue | 0.12 | -0.36 | 0.6 | 0.12(-0.36 to 0.6) |
| Singapore | 0.11 | -0.93 | 1.16 | 0.11(-0.93 to 1.16) |
| Nepal | 0.11 | -0.05 | 0.28 | 0.11(-0.05 to 0.28) |
| Bermuda | 0.11 | 0.05 | 0.17 | 0.11(0.05 to 0.17) |
| United Kingdom | 0.09 | -0.24 | 0.41 | 0.09(-0.24 to 0.41) |
| Liberia | 0.07 | -0.59 | 0.73 | 0.07(-0.59 to 0.73) |
| Mali | 0.05 | -0.69 | 0.79 | 0.05(-0.69 to 0.79) |
| Marshall Islands | 0.05 | -0.53 | 0.64 | 0.05(-0.53 to 0.64) |
| Sao Tome and Principe | -0.05 | -0.36 | 0.27 | -0.05(-0.36 to 0.27) |
| Cabo Verde | 0.02 | -0.37 | 0.42 | 0.02(-0.37 to 0.42) |
| Cuba | -0.02 | -0.3 | 0.25 | -0.02(-0.3 to 0.25) |
| Benin | 0.01 | -0.38 | 0.41 | 0.01(-0.38 to 0.41) |

Appendix

**Appendix 21：Global disease burden distribution of Chronic kidney disease due to diabetes mellitus type 1 in children and adolescents from 1990 to 2021**

| **location** | **EAPC** | **LCI** | **UCI** | **EAPC_CI** |
| --- | --- | --- | --- | --- |
| Seychelles | -6.81 | -9.82 | -3.7 | -6.81(-9.82 to -3.7) |
| Niue | 5.02 | 3.4 | 6.66 | 5.02(3.4 to 6.66) |
| Tokelau | 4.41 | 2.61 | 6.23 | 4.41(2.61 to 6.23) |
| Republic of Korea | -4.2 | -4.35 | -4.05 | -4.2(-4.35 to -4.05) |
| Ukraine | 3.58 | 3.15 | 4.01 | 3.58(3.15 to 4.01) |
| China | -3.28 | -3.81 | -2.74 | -3.28(-3.81 to -2.74) |
| Maldives | -2.79 | -3.14 | -2.44 | -2.79(-3.14 to -2.44) |
| Lesotho | 2.78 | 1.27 | 4.32 | 2.78(1.27 to 4.32) |
| Palau | 2.72 | -0.23 | 5.75 | 2.72(-0.23 to 5.75) |
| Ireland | 2.71 | 1.48 | 3.96 | 2.71(1.48 to 3.96) |
| Marshall Islands | 2.69 | 1.95 | 3.43 | 2.69(1.95 to 3.43) |
| American Samoa | 2.61 | 1.39 | 3.85 | 2.61(1.39 to 3.85) |
| Zimbabwe | 2.57 | 1.76 | 3.38 | 2.57(1.76 to 3.38) |
| Taiwan (Province of China) | -2.45 | -2.6 | -2.3 | -2.45(-2.6 to -2.3) |
| Canada | 2.42 | 1.18 | 3.68 | 2.42(1.18 to 3.68) |
| Ethiopia | -2.36 | -4.01 | -0.69 | -2.36(-4.01 to -0.69) |
| Russian Federation | -2.3 | -2.9 | -1.69 | -2.3(-2.9 to -1.69) |
| Dominica | 2.3 | -1.36 | 6.1 | 2.3(-1.36 to 6.1) |
| Colombia | -2.28 | -2.58 | -1.98 | -2.28(-2.58 to -1.98) |
| Norway | 2.27 | 0.77 | 3.79 | 2.27(0.77 to 3.79) |
| Armenia | 2.18 | 1.86 | 2.51 | 2.18(1.86 to 2.51) |
| Kuwait | -2.09 | -2.52 | -1.65 | -2.09(-2.52 to -1.65) |
| Uzbekistan | 2 | 1.82 | 2.17 | 2(1.82 to 2.17) |
| Rwanda | -1.98 | -3.84 | -0.09 | -1.98(-3.84 to -0.09) |
| United States Virgin Islands | -1.91 | -12.93 | 10.5 | -1.91(-12.93 to 10.5) |
| Brunei Darussalam | -1.89 | -2.31 | -1.46 | -1.89(-2.31 to -1.46) |
| Sweden | 1.87 | 1.4 | 2.34 | 1.87(1.4 to 2.34) |
| Denmark | 1.87 | 1.07 | 2.67 | 1.87(1.07 to 2.67) |
| Antigua and Barbuda | 1.86 | -1.86 | 5.71 | 1.86(-1.86 to 5.71) |
| Mauritius | 1.86 | 1.36 | 2.37 | 1.86(1.36 to 2.37) |
| South Sudan | 1.84 | 0.15 | 3.56 | 1.84(0.15 to 3.56) |
| Vanuatu | 1.83 | 1.42 | 2.24 | 1.83(1.42 to 2.24) |
| Belize | 1.82 | -1.37 | 5.11 | 1.82(-1.37 to 5.11) |
| Saint Kitts and Nevis | 1.8 | -1.8 | 5.54 | 1.8(-1.8 to 5.54) |
| Turkmenistan | 1.79 | 1.25 | 2.34 | 1.79(1.25 to 2.34) |
| Serbia | -1.76 | -3.52 | 0.02 | -1.76(-3.52 to 0.02) |
| Micronesia (Federated States of) | 1.76 | 0.56 | 2.98 | 1.76(0.56 to 2.98) |
| El Salvador | 1.75 | 1.37 | 2.13 | 1.75(1.37 to 2.13) |
| Fiji | 1.74 | 1.56 | 1.91 | 1.74(1.56 to 1.91) |
| Nauru | 1.73 | 1.19 | 2.26 | 1.73(1.19 to 2.26) |
| Finland | 1.7 | 1.38 | 2.03 | 1.7(1.38 to 2.03) |
| Georgia | 1.7 | 1.12 | 2.27 | 1.7(1.12 to 2.27) |
| Austria | 1.69 | 1.03 | 2.37 | 1.69(1.03 to 2.37) |
| Venezuela (Bolivarian Republic of) | 1.64 | 1.23 | 2.04 | 1.64(1.23 to 2.04) |
| Saint Vincent and the Grenadines | 1.64 | -0.86 | 4.2 | 1.64(-0.86 to 4.2) |
| Tonga | 1.63 | -5.18 | 8.93 | 1.63(-5.18 to 8.93) |
| Monaco | 1.62 | 1.32 | 1.92 | 1.62(1.32 to 1.92) |
| T眉rkiye | -1.6 | -1.73 | -1.47 | -1.6(-1.73 to -1.47) |
| Republic of Moldova | 1.58 | 1.32 | 1.83 | 1.58(1.32 to 1.83) |
| Saint Lucia | 1.55 | -2.27 | 5.51 | 1.55(-2.27 to 5.51) |
| Malta | 1.54 | 0.76 | 2.33 | 1.54(0.76 to 2.33) |
| Guyana | 1.47 | -1.98 | 5.04 | 1.47(-1.98 to 5.04) |
| San Marino | 1.46 | 0.75 | 2.18 | 1.46(0.75 to 2.18) |
| Thailand | -1.44 | -1.99 | -0.88 | -1.44(-1.99 to -0.88) |
| Bahamas | 1.43 | -2.03 | 5 | 1.43(-2.03 to 5) |
| Japan | -1.42 | -1.78 | -1.06 | -1.42(-1.78 to -1.06) |
| Brazil | -1.39 | -2.38 | -0.39 | -1.39(-2.38 to -0.39) |
| Iceland | 1.39 | 0.3 | 2.49 | 1.39(0.3 to 2.49) |
| Grenada | 1.38 | -1.67 | 4.53 | 1.38(-1.67 to 4.53) |
| Pakistan | 1.37 | 0.97 | 1.77 | 1.37(0.97 to 1.77) |
| Niger | -1.33 | -3.83 | 1.24 | -1.33(-3.83 to 1.24) |
| Australia | 1.32 | 0.67 | 1.97 | 1.32(0.67 to 1.97) |
| Chile | -1.3 | -1.51 | -1.09 | -1.3(-1.51 to -1.09) |
| South Africa | 1.3 | 0.12 | 2.48 | 1.3(0.12 to 2.48) |
| Dominican Republic | 1.29 | -2.81 | 5.55 | 1.29(-2.81 to 5.55) |
| Iraq | -1.26 | -1.68 | -0.84 | -1.26(-1.68 to -0.84) |
| Poland | -1.22 | -1.82 | -0.61 | -1.22(-1.82 to -0.61) |
| Uganda | 1.22 | -1.43 | 3.94 | 1.22(-1.43 to 3.94) |
| Eswatini | 1.21 | -0.37 | 2.81 | 1.21(-0.37 to 2.81) |
| Myanmar | -1.19 | -2.21 | -0.16 | -1.19(-2.21 to -0.16) |
| Burundi | -1.16 | -3.23 | 0.95 | -1.16(-3.23 to 0.95) |
| Guam | 1.16 | -2.13 | 4.57 | 1.16(-2.13 to 4.57) |
| Italy | 1.16 | 0.59 | 1.74 | 1.16(0.59 to 1.74) |
| Paraguay | 1.15 | 0.5 | 1.82 | 1.15(0.5 to 1.82) |
| Suriname | 1.14 | -1.4 | 3.75 | 1.14(-1.4 to 3.75) |
| Bangladesh | -1.11 | -1.82 | -0.39 | -1.11(-1.82 to -0.39) |
| Bulgaria | 1.08 | 0.02 | 2.15 | 1.08(0.02 to 2.15) |
| Democratic People's Republic of Korea | -1.07 | -1.45 | -0.7 | -1.07(-1.45 to -0.7) |
| Cambodia | -1.06 | -2 | -0.1 | -1.06(-2 to -0.1) |
| Papua New Guinea | 1.06 | 0.78 | 1.34 | 1.06(0.78 to 1.34) |
| Mauritania | -1.05 | -2.66 | 0.58 | -1.05(-2.66 to 0.58) |
| North Macedonia | 1.05 | 0.73 | 1.37 | 1.05(0.73 to 1.37) |
| Belarus | 1.03 | 0.4 | 1.66 | 1.03(0.4 to 1.66) |
| Netherlands | 1.03 | 0.25 | 1.81 | 1.03(0.25 to 1.81) |
| Singapore | -1.02 | -2.18 | 0.15 | -1.02(-2.18 to 0.15) |
| Lebanon | -1.02 | -1.52 | -0.52 | -1.02(-1.52 to -0.52) |
| Costa Rica | 1.02 | 0.46 | 1.59 | 1.02(0.46 to 1.59) |
| Panama | 1.02 | 0.16 | 1.88 | 1.02(0.16 to 1.88) |
| Honduras | -1.01 | -2.44 | 0.43 | -1.01(-2.44 to 0.43) |
| Kenya | 1.01 | -1.22 | 3.29 | 1.01(-1.22 to 3.29) |
| Ghana | 1 | -2.14 | 4.24 | 1(-2.14 to 4.24) |
| Gabon | 0.99 | -1.28 | 3.31 | 0.99(-1.28 to 3.31) |
| Portugal | -0.96 | -2.01 | 0.1 | -0.96(-2.01 to 0.1) |
| Sri Lanka | -0.95 | -1.04 | -0.86 | -0.95(-1.04 to -0.86) |
| Libya | 0.95 | 0.56 | 1.35 | 0.95(0.56 to 1.35) |
| Guinea-Bissau | -0.93 | -3.86 | 2.09 | -0.93(-3.86 to 2.09) |
| United States of America | -0.93 | -1.83 | -0.03 | -0.93(-1.83 to -0.03) |
| Germany | 0.92 | 0.41 | 1.42 | 0.92(0.41 to 1.42) |
| Guatemala | 0.91 | -0.04 | 1.87 | 0.91(-0.04 to 1.87) |
| Trinidad and Tobago | 0.9 | -3.27 | 5.25 | 0.9(-3.27 to 5.25) |
| Lao People's Democratic Republic | -0.9 | -1.34 | -0.46 | -0.9(-1.34 to -0.46) |
| Argentina | -0.89 | -1.1 | -0.67 | -0.89(-1.1 to -0.67) |
| Mozambique | 0.88 | -2.02 | 3.87 | 0.88(-2.02 to 3.87) |
| Kiribati | 0.88 | 0.24 | 1.53 | 0.88(0.24 to 1.53) |
| Bolivia (Plurinational State of) | -0.86 | -1.28 | -0.45 | -0.86(-1.28 to -0.45) |
| United Arab Emirates | -0.86 | -1.08 | -0.64 | -0.86(-1.08 to -0.64) |
| Jordan | -0.85 | -2.02 | 0.33 | -0.85(-2.02 to 0.33) |
| Ecuador | -0.85 | -1.93 | 0.24 | -0.85(-1.93 to 0.24) |
| Algeria | -0.84 | -1.52 | -0.15 | -0.84(-1.52 to -0.15) |
| India | -0.84 | -1.3 | -0.38 | -0.84(-1.3 to -0.38) |
| Israel | 0.83 | 0.09 | 1.57 | 0.83(0.09 to 1.57) |
| Northern Mariana Islands | 0.79 | -0.93 | 2.55 | 0.79(-0.93 to 2.55) |
| Montenegro | 0.79 | -0.08 | 1.67 | 0.79(-0.08 to 1.67) |
| Bermuda | 0.77 | -2.55 | 4.21 | 0.77(-2.55 to 4.21) |
| Kazakhstan | -0.77 | -1.03 | -0.51 | -0.77(-1.03 to -0.51) |
| Afghanistan | -0.76 | -2.82 | 1.34 | -0.76(-2.82 to 1.34) |
| Qatar | -0.76 | -1.32 | -0.2 | -0.76(-1.32 to -0.2) |
| Palestine | -0.76 | -0.97 | -0.55 | -0.76(-0.97 to -0.55) |
| Tuvalu | 0.73 | 0.2 | 1.26 | 0.73(0.2 to 1.26) |
| Samoa | 0.73 | -0.19 | 1.65 | 0.73(-0.19 to 1.65) |
| Djibouti | 0.72 | -1.92 | 3.43 | 0.72(-1.92 to 3.43) |
| Belgium | 0.7 | -0.05 | 1.46 | 0.7(-0.05 to 1.46) |
| Cuba | -0.69 | -3.74 | 2.45 | -0.69(-3.74 to 2.45) |
| Syrian Arab Republic | -0.69 | -0.94 | -0.43 | -0.69(-0.94 to -0.43) |
| Tajikistan | 0.68 | 0.43 | 0.93 | 0.68(0.43 to 0.93) |
| Senegal | -0.66 | -2.97 | 1.71 | -0.66(-2.97 to 1.71) |
| Kyrgyzstan | 0.64 | 0.52 | 0.75 | 0.64(0.52 to 0.75) |
| Cabo Verde | 0.63 | -2.31 | 3.65 | 0.63(-2.31 to 3.65) |
| Puerto Rico | -0.61 | -1.67 | 0.45 | -0.61(-1.67 to 0.45) |
| Hungary | 0.61 | -0.46 | 1.68 | 0.61(-0.46 to 1.68) |
| Oman | 0.58 | 0.19 | 0.96 | 0.58(0.19 to 0.96) |
| Switzerland | 0.58 | -0.07 | 1.23 | 0.58(-0.07 to 1.23) |
| Azerbaijan | 0.58 | 0.02 | 1.14 | 0.58(0.02 to 1.14) |
| Angola | -0.57 | -2.87 | 1.78 | -0.57(-2.87 to 1.78) |
| Andorra | 0.57 | 0.27 | 0.87 | 0.57(0.27 to 0.87) |
| Greece | 0.57 | 0.04 | 1.1 | 0.57(0.04 to 1.1) |
| Solomon Islands | 0.56 | 0.21 | 0.91 | 0.56(0.21 to 0.91) |
| Nigeria | -0.55 | -3.33 | 2.31 | -0.55(-3.33 to 2.31) |
| Timor-Leste | -0.54 | -1.01 | -0.06 | -0.54(-1.01 to -0.06) |
| Gambia | 0.53 | -2.59 | 3.76 | 0.53(-2.59 to 3.76) |
| Peru | -0.52 | -1.33 | 0.3 | -0.52(-1.33 to 0.3) |
| Botswana | 0.47 | -1.09 | 2.05 | 0.47(-1.09 to 2.05) |
| Slovenia | 0.47 | -0.7 | 1.66 | 0.47(-0.7 to 1.66) |
| Mali | -0.45 | -2.1 | 1.22 | -0.45(-2.1 to 1.22) |
| Sao Tome and Principe | 0.44 | -1.68 | 2.6 | 0.44(-1.68 to 2.6) |
| Croatia | 0.44 | -0.35 | 1.23 | 0.44(-0.35 to 1.23) |
| Democratic Republic of the Congo | -0.43 | -2.32 | 1.5 | -0.43(-2.32 to 1.5) |
| Malaysia | -0.42 | -1.12 | 0.28 | -0.42(-1.12 to 0.28) |
| Somalia | 0.39 | -2.16 | 2.99 | 0.39(-2.16 to 2.99) |
| Bosnia and Herzegovina | 0.39 | -0.41 | 1.19 | 0.39(-0.41 to 1.19) |
| Mexico | -0.38 | -1 | 0.25 | -0.38(-1 to 0.25) |
| Tunisia | -0.35 | -0.48 | -0.22 | -0.35(-0.48 to -0.22) |
| Uruguay | 0.35 | 0.01 | 0.69 | 0.35(0.01 to 0.69) |
| Bahrain | -0.34 | -1.19 | 0.51 | -0.34(-1.19 to 0.51) |
| Liberia | -0.33 | -2.39 | 1.77 | -0.33(-2.39 to 1.77) |
| Luxembourg | 0.33 | -0.58 | 1.25 | 0.33(-0.58 to 1.25) |
| Haiti | -0.3 | -2.38 | 1.83 | -0.3(-2.38 to 1.83) |
| United Kingdom | -0.3 | -1.15 | 0.56 | -0.3(-1.15 to 0.56) |
| Saudi Arabia | 0.3 | -0.86 | 1.47 | 0.3(-0.86 to 1.47) |
| Romania | 0.3 | -0.45 | 1.06 | 0.3(-0.45 to 1.06) |
| Cyprus | 0.29 | -0.36 | 0.96 | 0.29(-0.36 to 0.96) |
| Chad | 0.27 | -2.88 | 3.52 | 0.27(-2.88 to 3.52) |
| Morocco | -0.27 | -1.15 | 0.61 | -0.27(-1.15 to 0.61) |
| Sierra Leone | -0.26 | -2.88 | 2.44 | -0.26(-2.88 to 2.44) |
| Togo | -0.26 | -2.41 | 1.93 | -0.26(-2.41 to 1.93) |
| Iran (Islamic Republic of) | -0.25 | -0.33 | -0.16 | -0.25(-0.33 to -0.16) |
| Benin | -0.23 | -2.53 | 2.13 | -0.23(-2.53 to 2.13) |
| Burkina Faso | 0.23 | -1.06 | 1.53 | 0.23(-1.06 to 1.53) |
| Albania | -0.23 | -0.91 | 0.46 | -0.23(-0.91 to 0.46) |
| Czechia | 0.21 | -0.75 | 1.19 | 0.21(-0.75 to 1.19) |
| Egypt | 0.21 | -0.29 | 0.72 | 0.21(-0.29 to 0.72) |
| Greenland | 0.21 | -0.29 | 0.71 | 0.21(-0.29 to 0.71) |
| New Zealand | 0.2 | -0.33 | 0.73 | 0.2(-0.33 to 0.73) |
| Indonesia | -0.19 | -0.59 | 0.2 | -0.19(-0.59 to 0.2) |
| Viet Nam | -0.18 | -0.49 | 0.12 | -0.18(-0.49 to 0.12) |
| Madagascar | -0.16 | -1.88 | 1.58 | -0.16(-1.88 to 1.58) |
| Lithuania | 0.16 | -0.2 | 0.52 | 0.16(-0.2 to 0.52) |
| Guinea | -0.15 | -2.24 | 1.99 | -0.15(-2.24 to 1.99) |
| Nepal | 0.15 | -0.58 | 0.89 | 0.15(-0.58 to 0.89) |
| France | 0.15 | -0.38 | 0.68 | 0.15(-0.38 to 0.68) |
| Comoros | -0.14 | -1.48 | 1.21 | -0.14(-1.48 to 1.21) |
| Yemen | -0.14 | -0.39 | 0.11 | -0.14(-0.39 to 0.11) |
| Nicaragua | 0.14 | -0.14 | 0.42 | 0.14(-0.14 to 0.42) |
| Sudan | 0.13 | -0.48 | 0.74 | 0.13(-0.48 to 0.74) |
| Eritrea | -0.12 | -3.34 | 3.21 | -0.12(-3.34 to 3.21) |
| Estonia | -0.11 | -1.32 | 1.12 | -0.11(-1.32 to 1.12) |
| Latvia | -0.11 | -0.39 | 0.16 | -0.11(-0.39 to 0.16) |
| Jamaica | 0.1 | -3.1 | 3.39 | 0.1(-3.1 to 3.39) |
| Barbados | 0.1 | -2.82 | 3.1 | 0.1(-2.82 to 3.1) |
| Equatorial Guinea | 0.07 | -2.23 | 2.42 | 0.07(-2.23 to 2.42) |
| Namibia | -0.07 | -1.58 | 1.47 | -0.07(-1.58 to 1.47) |
| Philippines | 0.07 | -0.16 | 0.29 | 0.07(-0.16 to 0.29) |
| Slovakia | 0.06 | -0.72 | 0.85 | 0.06(-0.72 to 0.85) |
| Spain | -0.06 | -0.53 | 0.42 | -0.06(-0.53 to 0.42) |
| United Republic of Tanzania | -0.05 | -1.84 | 1.76 | -0.05(-1.84 to 1.76) |
| Cook Islands | 0.05 | -0.25 | 0.34 | 0.05(-0.25 to 0.34) |
| Zambia | 0.04 | -1.81 | 1.92 | 0.04(-1.81 to 1.92) |
| Cameroon | 0.03 | -1.79 | 1.89 | 0.03(-1.79 to 1.89) |
| Bhutan | -0.03 | -0.54 | 0.49 | -0.03(-0.54 to 0.49) |
| Mongolia | 0.03 | -0.4 | 0.46 | 0.03(-0.4 to 0.46) |
| C么te d'Ivoire | 0.02 | -2.67 | 2.79 | 0.02(-2.67 to 2.79) |
| Malawi | 0.02 | -2.14 | 2.23 | 0.02(-2.14 to 2.23) |
| Central African Republic | 0 | -2.97 | 3.05 | 0(-2.97 to 3.05) |
| Congo | 0 | -2.26 | 2.32 | 0(-2.26 to 2.32) |

Appendix

**Appendix 22：Global disease burden distribution of Chronic kidney disease due to diabetes mellitus type 1 in children and adolescents from 1990 to 2021**

| **location** | **EAPC** | **LCI** | **UCI** | **EAPC_CI** |
| --- | --- | --- | --- | --- |
| Ukraine | 7.79 | 6.62 | 8.97 | 7.79(6.62 to 8.97) |
| Niue | 5.02 | 3.39 | 6.68 | 5.02(3.39 to 6.68) |
| Armenia | 4.77 | 4.17 | 5.37 | 4.77(4.17 to 5.37) |
| Tokelau | 4.41 | 2.59 | 6.27 | 4.41(2.59 to 6.27) |
| Lesotho | 3.23 | 1.33 | 5.16 | 3.23(1.33 to 5.16) |
| Zimbabwe | 2.97 | 1.96 | 4 | 2.97(1.96 to 4) |
| Uzbekistan | 2.84 | 2.5 | 3.18 | 2.84(2.5 to 3.18) |
| Palau | 2.75 | -0.24 | 5.83 | 2.75(-0.24 to 5.83) |
| Marshall Islands | 2.72 | 1.98 | 3.47 | 2.72(1.98 to 3.47) |
| American Samoa | 2.63 | 1.41 | 3.88 | 2.63(1.41 to 3.88) |
| Dominica | 2.36 | -1.4 | 6.27 | 2.36(-1.4 to 6.27) |
| Georgia | 2.21 | 1.61 | 2.82 | 2.21(1.61 to 2.82) |
| Turkmenistan | 2.21 | 1.27 | 3.16 | 2.21(1.27 to 3.16) |
| South Sudan | 1.95 | 0.18 | 3.75 | 1.95(0.18 to 3.75) |
| Antigua and Barbuda | 1.93 | -1.89 | 5.91 | 1.93(-1.89 to 5.91) |
| Saint Kitts and Nevis | 1.92 | -1.75 | 5.74 | 1.92(-1.75 to 5.74) |
| Mauritius | 1.91 | 1.39 | 2.44 | 1.91(1.39 to 2.44) |
| Belize | 1.91 | -1.37 | 5.31 | 1.91(-1.37 to 5.31) |
| Vanuatu | 1.86 | 1.44 | 2.28 | 1.86(1.44 to 2.28) |
| Venezuela (Bolivarian Republic of) | 1.84 | 1.32 | 2.36 | 1.84(1.32 to 2.36) |
| El Salvador | 1.82 | 1.44 | 2.19 | 1.82(1.44 to 2.19) |
| Micronesia (Federated States of) | 1.8 | 0.57 | 3.04 | 1.8(0.57 to 3.04) |
| Tonga | 1.79 | -5.7 | 9.87 | 1.79(-5.7 to 9.87) |
| Fiji | 1.75 | 1.56 | 1.94 | 1.75(1.56 to 1.94) |
| Nauru | 1.75 | 1.2 | 2.3 | 1.75(1.2 to 2.3) |
| Saint Vincent and the Grenadines | 1.7 | -0.87 | 4.33 | 1.7(-0.87 to 4.33) |
| Saint Lucia | 1.6 | -2.29 | 5.65 | 1.6(-2.29 to 5.65) |
| Guyana | 1.53 | -1.99 | 5.18 | 1.53(-1.99 to 5.18) |
| Pakistan | 1.5 | 0.98 | 2.02 | 1.5(0.98 to 2.02) |
| Bahamas | 1.5 | -2.06 | 5.18 | 1.5(-2.06 to 5.18) |
| South Africa | 1.43 | 0.06 | 2.83 | 1.43(0.06 to 2.83) |
| Grenada | 1.42 | -1.67 | 4.6 | 1.42(-1.67 to 4.6) |
| Canada | 1.39 | 0.03 | 2.77 | 1.39(0.03 to 2.77) |
| Dominican Republic | 1.35 | -2.86 | 5.75 | 1.35(-2.86 to 5.75) |
| Uganda | 1.31 | -1.51 | 4.22 | 1.31(-1.51 to 4.22) |
| Sweden | 1.31 | 0.64 | 1.98 | 1.31(0.64 to 1.98) |
| Eswatini | 1.29 | -0.44 | 3.05 | 1.29(-0.44 to 3.05) |
| Costa Rica | 1.28 | 0.51 | 2.05 | 1.28(0.51 to 2.05) |
| Libya | 1.24 | 0.72 | 1.75 | 1.24(0.72 to 1.75) |
| Paraguay | 1.21 | 0.5 | 1.93 | 1.21(0.5 to 1.93) |
| Guam | 1.18 | -2.19 | 4.66 | 1.18(-2.19 to 4.66) |
| Suriname | 1.17 | -1.42 | 3.83 | 1.17(-1.42 to 3.83) |
| Panama | 1.16 | 0.07 | 2.27 | 1.16(0.07 to 2.27) |
| Gabon | 1.09 | -1.29 | 3.53 | 1.09(-1.29 to 3.53) |
| Papua New Guinea | 1.08 | 0.8 | 1.37 | 1.08(0.8 to 1.37) |
| Malta | 1.05 | 0.46 | 1.64 | 1.05(0.46 to 1.64) |
| Ghana | 1.03 | -2.25 | 4.42 | 1.03(-2.25 to 4.42) |
| Kenya | 1.02 | -1.25 | 3.34 | 1.02(-1.25 to 3.34) |
| Guatemala | 1 | -0.08 | 2.09 | 1(-0.08 to 2.09) |
| Kyrgyzstan | 0.97 | 0.79 | 1.16 | 0.97(0.79 to 1.16) |
| Oman | 0.97 | 0.68 | 1.26 | 0.97(0.68 to 1.26) |
| Trinidad and Tobago | 0.96 | -3.33 | 5.44 | 0.96(-3.33 to 5.44) |
| Mozambique | 0.94 | -2.08 | 4.06 | 0.94(-2.08 to 4.06) |
| Kiribati | 0.89 | 0.23 | 1.55 | 0.89(0.23 to 1.55) |
| Bermuda | 0.85 | -2.64 | 4.46 | 0.85(-2.64 to 4.46) |
| Northern Mariana Islands | 0.81 | -0.9 | 2.54 | 0.81(-0.9 to 2.54) |
| Cabo Verde | 0.8 | -2.42 | 4.12 | 0.8(-2.42 to 4.12) |
| Djibouti | 0.79 | -2.01 | 3.67 | 0.79(-2.01 to 3.67) |
| Tuvalu | 0.75 | 0.21 | 1.3 | 0.75(0.21 to 1.3) |
| Samoa | 0.75 | -0.19 | 1.7 | 0.75(-0.19 to 1.7) |
| Gambia | 0.62 | -2.7 | 4.06 | 0.62(-2.7 to 4.06) |
| Monaco | 0.62 | 0.04 | 1.2 | 0.62(0.04 to 1.2) |
| Sao Tome and Principe | 0.6 | -1.63 | 2.88 | 0.6(-1.63 to 2.88) |
| Solomon Islands | 0.57 | 0.21 | 0.93 | 0.57(0.21 to 0.93) |
| Botswana | 0.57 | -1.37 | 2.55 | 0.57(-1.37 to 2.55) |
| Nepal | 0.5 | -0.7 | 1.71 | 0.5(-0.7 to 1.71) |
| Egypt | 0.43 | -0.35 | 1.21 | 0.43(-0.35 to 1.21) |
| Somalia | 0.42 | -2.25 | 3.16 | 0.42(-2.25 to 3.16) |
| Norway | 0.42 | -1.71 | 2.59 | 0.42(-1.71 to 2.59) |
| Mongolia | 0.42 | 0.26 | 0.59 | 0.42(0.26 to 0.59) |
| Sudan | 0.37 | -0.72 | 1.46 | 0.37(-0.72 to 1.46) |
| Saudi Arabia | 0.35 | -0.94 | 1.66 | 0.35(-0.94 to 1.66) |
| Republic of Moldova | 0.32 | -0.47 | 1.12 | 0.32(-0.47 to 1.12) |
| Chad | 0.29 | -3.09 | 3.79 | 0.29(-3.09 to 3.79) |
| New Zealand | 0.25 | -1.23 | 1.74 | 0.25(-1.23 to 1.74) |
| Burkina Faso | 0.24 | -1.15 | 1.64 | 0.24(-1.15 to 1.64) |
| Nicaragua | 0.23 | -0.21 | 0.68 | 0.23(-0.21 to 0.68) |
| Azerbaijan | 0.22 | -0.27 | 0.71 | 0.22(-0.27 to 0.71) |
| Montenegro | 0.2 | -0.07 | 0.48 | 0.2(-0.07 to 0.48) |
| Tajikistan | 0.17 | -0.57 | 0.92 | 0.17(-0.57 to 0.92) |
| Equatorial Guinea | 0.13 | -2.31 | 2.63 | 0.13(-2.31 to 2.63) |
| Finland | 0.13 | -0.54 | 0.81 | 0.13(-0.54 to 0.81) |
| Jamaica | 0.12 | -3.21 | 3.56 | 0.12(-3.21 to 3.56) |
| Namibia | 0.12 | -1.97 | 2.25 | 0.12(-1.97 to 2.25) |
| Barbados | 0.11 | -2.86 | 3.18 | 0.11(-2.86 to 3.18) |
| Philippines | 0.08 | -0.15 | 0.3 | 0.08(-0.15 to 0.3) |
| Uruguay | 0.08 | -0.54 | 0.7 | 0.08(-0.54 to 0.7) |
| Austria | 0.08 | -0.74 | 0.91 | 0.08(-0.74 to 0.91) |
| Cook Islands | 0.07 | -0.24 | 0.38 | 0.07(-0.24 to 0.38) |
| C么te d'Ivoire | 0.06 | -2.85 | 3.07 | 0.06(-2.85 to 3.07) |
| Congo | 0.05 | -2.35 | 2.51 | 0.05(-2.35 to 2.51) |
| Zambia | 0.05 | -1.84 | 1.98 | 0.05(-1.84 to 1.98) |
| Cameroon | 0.03 | -1.83 | 1.93 | 0.03(-1.83 to 1.93) |
| Malawi | 0.02 | -2.26 | 2.36 | 0.02(-2.26 to 2.36) |
| Central African Republic | 0.01 | -3.06 | 3.18 | 0.01(-3.06 to 3.18) |
| Iran (Islamic Republic of) | -0.03 | -0.13 | 0.07 | -0.03(-0.13 to 0.07) |
| Eritrea | -0.06 | -3.46 | 3.46 | -0.06(-3.46 to 3.46) |
| Bhutan | -0.06 | -0.71 | 0.6 | -0.06(-0.71 to 0.6) |
| United Republic of Tanzania | -0.06 | -2.06 | 1.98 | -0.06(-2.06 to 1.98) |
| Comoros | -0.1 | -1.48 | 1.31 | -0.1(-1.48 to 1.31) |
| Greece | -0.11 | -0.46 | 0.23 | -0.11(-0.46 to 0.23) |
| Madagascar | -0.14 | -1.91 | 1.66 | -0.14(-1.91 to 1.66) |
| Iceland | -0.14 | -1.72 | 1.47 | -0.14(-1.72 to 1.47) |
| Morocco | -0.16 | -1.65 | 1.34 | -0.16(-1.65 to 1.34) |
| Indonesia | -0.17 | -0.59 | 0.24 | -0.17(-0.59 to 0.24) |
| Guinea | -0.17 | -2.45 | 2.17 | -0.17(-2.45 to 2.17) |
| Viet Nam | -0.19 | -0.51 | 0.14 | -0.19(-0.51 to 0.14) |
| North Macedonia | -0.21 | -0.65 | 0.23 | -0.21(-0.65 to 0.23) |
| Sierra Leone | -0.24 | -3.12 | 2.71 | -0.24(-3.12 to 2.71) |
| Benin | -0.26 | -2.76 | 2.31 | -0.26(-2.76 to 2.31) |
| Bahrain | -0.26 | -1.3 | 0.79 | -0.26(-1.3 to 0.79) |
| Tunisia | -0.26 | -0.4 | -0.11 | -0.26(-0.4 to -0.11) |
| Yemen | -0.27 | -0.4 | -0.14 | -0.27(-0.4 to -0.14) |
| Togo | -0.29 | -2.65 | 2.13 | -0.29(-2.65 to 2.13) |
| Belarus | -0.3 | -0.73 | 0.13 | -0.3(-0.73 to 0.13) |
| Liberia | -0.3 | -2.44 | 1.88 | -0.3(-2.44 to 1.88) |
| Haiti | -0.31 | -2.4 | 1.83 | -0.31(-2.4 to 1.83) |
| Mexico | -0.38 | -1.08 | 0.32 | -0.38(-1.08 to 0.32) |
| Australia | -0.38 | -0.89 | 0.13 | -0.38(-0.89 to 0.13) |
| Democratic Republic of the Congo | -0.38 | -2.39 | 1.67 | -0.38(-2.39 to 1.67) |
| Malaysia | -0.42 | -1.15 | 0.31 | -0.42(-1.15 to 0.31) |
| Peru | -0.49 | -1.32 | 0.35 | -0.49(-1.32 to 0.35) |
| Timor-Leste | -0.51 | -1.01 | -0.02 | -0.51(-1.01 to -0.02) |
| Mali | -0.51 | -2.39 | 1.41 | -0.51(-2.39 to 1.41) |
| Israel | -0.54 | -1.2 | 0.11 | -0.54(-1.2 to 0.11) |
| Angola | -0.58 | -3.03 | 1.93 | -0.58(-3.03 to 1.93) |
| Syrian Arab Republic | -0.62 | -0.95 | -0.29 | -0.62(-0.95 to -0.29) |
| San Marino | -0.62 | -1.15 | -0.08 | -0.62(-1.15 to -0.08) |
| Greenland | -0.63 | -2.19 | 0.97 | -0.63(-2.19 to 0.97) |
| Senegal | -0.65 | -3.05 | 1.8 | -0.65(-3.05 to 1.8) |
| Netherlands | -0.65 | -1.58 | 0.29 | -0.65(-1.58 to 0.29) |
| Nigeria | -0.69 | -3.96 | 2.69 | -0.69(-3.96 to 2.69) |
| United Kingdom | -0.71 | -1.28 | -0.13 | -0.71(-1.28 to -0.13) |
| Cuba | -0.72 | -3.84 | 2.5 | -0.72(-3.84 to 2.5) |
| Denmark | -0.72 | -1.22 | -0.21 | -0.72(-1.22 to -0.21) |
| Puerto Rico | -0.73 | -1.85 | 0.4 | -0.73(-1.85 to 0.4) |
| Afghanistan | -0.74 | -3.14 | 1.72 | -0.74(-3.14 to 1.72) |
| Belgium | -0.76 | -0.97 | -0.54 | -0.76(-0.97 to -0.54) |
| Algeria | -0.79 | -1.71 | 0.15 | -0.79(-1.71 to 0.15) |
| Bulgaria | -0.8 | -1.27 | -0.32 | -0.8(-1.27 to -0.32) |
| Germany | -0.81 | -1.54 | -0.07 | -0.81(-1.54 to -0.07) |
| Qatar | -0.81 | -1.68 | 0.06 | -0.81(-1.68 to 0.06) |
| Estonia | -0.83 | -2.75 | 1.12 | -0.83(-2.75 to 1.12) |
| Bolivia (Plurinational State of) | -0.84 | -1.22 | -0.45 | -0.84(-1.22 to -0.45) |
| Jordan | -0.85 | -2.13 | 0.45 | -0.85(-2.13 to 0.45) |
| Ecuador | -0.86 | -1.98 | 0.26 | -0.86(-1.98 to 0.26) |
| Lao People's Democratic Republic | -0.88 | -1.33 | -0.43 | -0.88(-1.33 to -0.43) |
| India | -0.92 | -1.42 | -0.41 | -0.92(-1.42 to -0.41) |
| Palestine | -0.93 | -1.15 | -0.71 | -0.93(-1.15 to -0.71) |
| Honduras | -0.94 | -2.94 | 1.1 | -0.94(-2.94 to 1.1) |
| Sri Lanka | -0.95 | -1.05 | -0.85 | -0.95(-1.05 to -0.85) |
| Guinea-Bissau | -0.95 | -4.03 | 2.24 | -0.95(-4.03 to 2.24) |
| Bosnia and Herzegovina | -1.03 | -1.19 | -0.86 | -1.03(-1.19 to -0.86) |
| Cambodia | -1.04 | -2.01 | -0.06 | -1.04(-2.01 to -0.06) |
| Democratic People's Republic of Korea | -1.07 | -1.45 | -0.69 | -1.07(-1.45 to -0.69) |
| Lebanon | -1.09 | -1.7 | -0.48 | -1.09(-1.7 to -0.48) |
| Mauritania | -1.1 | -2.83 | 0.65 | -1.1(-2.83 to 0.65) |
| Bangladesh | -1.12 | -1.69 | -0.55 | -1.12(-1.69 to -0.55) |
| Lithuania | -1.13 | -1.48 | -0.78 | -1.13(-1.48 to -0.78) |
| Kazakhstan | -1.14 | -1.43 | -0.85 | -1.14(-1.43 to -0.85) |
| Ireland | -1.17 | -1.58 | -0.75 | -1.17(-1.58 to -0.75) |
| Myanmar | -1.19 | -2.23 | -0.14 | -1.19(-2.23 to -0.14) |
| Argentina | -1.19 | -1.46 | -0.93 | -1.19(-1.46 to -0.93) |
| Burundi | -1.19 | -3.33 | 0.99 | -1.19(-3.33 to 0.99) |
| Slovakia | -1.24 | -1.36 | -1.11 | -1.24(-1.36 to -1.11) |
| Iraq | -1.29 | -1.84 | -0.73 | -1.29(-1.84 to -0.73) |
| France | -1.35 | -1.54 | -1.17 | -1.35(-1.54 to -1.17) |
| Niger | -1.4 | -4.19 | 1.48 | -1.4(-4.19 to 1.48) |
| Switzerland | -1.4 | -2.17 | -0.63 | -1.4(-2.17 to -0.63) |
| Singapore | -1.41 | -2.8 | -0.01 | -1.41(-2.8 to -0.01) |
| Thailand | -1.42 | -1.99 | -0.86 | -1.42(-1.99 to -0.86) |
| United Arab Emirates | -1.46 | -1.8 | -1.13 | -1.46(-1.8 to -1.13) |
| Brazil | -1.61 | -2.76 | -0.45 | -1.61(-2.76 to -0.45) |
| Andorra | -1.66 | -2.54 | -0.76 | -1.66(-2.54 to -0.76) |
| Croatia | -1.74 | -2.12 | -1.36 | -1.74(-2.12 to -1.36) |
| T眉rkiye | -1.78 | -2.06 | -1.5 | -1.78(-2.06 to -1.5) |
| Chile | -1.82 | -2.01 | -1.64 | -1.82(-2.01 to -1.64) |
| Luxembourg | -1.83 | -2.76 | -0.89 | -1.83(-2.76 to -0.89) |
| Spain | -1.89 | -2.45 | -1.33 | -1.89(-2.45 to -1.33) |
| Latvia | -1.9 | -3.6 | -0.17 | -1.9(-3.6 to -0.17) |
| Italy | -2.04 | -2.84 | -1.24 | -2.04(-2.84 to -1.24) |
| Rwanda | -2.05 | -3.95 | -0.12 | -2.05(-3.95 to -0.12) |
| Portugal | -2.19 | -3.05 | -1.32 | -2.19(-3.05 to -1.32) |
| Hungary | -2.22 | -3.03 | -1.41 | -2.22(-3.03 to -1.41) |
| Japan | -2.24 | -2.88 | -1.59 | -2.24(-2.88 to -1.59) |
| United States of America | -2.29 | -3.04 | -1.53 | -2.29(-3.04 to -1.53) |
| Ethiopia | -2.37 | -4.03 | -0.67 | -2.37(-4.03 to -0.67) |
| Colombia | -2.4 | -2.77 | -2.04 | -2.4(-2.77 to -2.04) |
| Romania | -2.43 | -2.8 | -2.07 | -2.43(-2.8 to -2.07) |
| Albania | -2.44 | -2.66 | -2.22 | -2.44(-2.66 to -2.22) |
| Brunei Darussalam | -2.47 | -2.97 | -1.97 | -2.47(-2.97 to -1.97) |
| Taiwan (Province of China) | -2.52 | -2.68 | -2.36 | -2.52(-2.68 to -2.36) |
| Slovenia | -2.53 | -3.24 | -1.82 | -2.53(-3.24 to -1.82) |
| Czechia | -2.55 | -2.77 | -2.33 | -2.55(-2.77 to -2.33) |
| Kuwait | -2.57 | -3.15 | -1.98 | -2.57(-3.15 to -1.98) |
| Maldives | -2.8 | -3.17 | -2.42 | -2.8(-3.17 to -2.42) |
| Serbia | -3.23 | -4.11 | -2.33 | -3.23(-4.11 to -2.33) |
| China | -3.3 | -3.84 | -2.75 | -3.3(-3.84 to -2.75) |
| Cyprus | -3.3 | -3.99 | -2.62 | -3.3(-3.99 to -2.62) |
| United States Virgin Islands | -3.78 | -17.74 | 12.55 | -3.78(-17.74 to 12.55) |
| Poland | -3.8 | -4 | -3.6 | -3.8(-4 to -3.6) |
| Russian Federation | -4.11 | -5.34 | -2.85 | -4.11(-5.34 to -2.85) |
| Republic of Korea | -4.78 | -5 | -4.57 | -4.78(-5 to -4.57) |
| Seychelles | -7.58 | -11.19 | -3.83 | -7.58(-11.19 to -3.83) |

Appendix

**Appendix 23：Percentage contribution of risk factors to Chronic kidney disease due to diabetes mellitus type 1（CKD-T1DM） in 2019**

| **measure** | **location** | **sex** | **age** | **cause** | **rei** | **metric** | **year** | **val** | **upper** | **lower** |
| --- | --- | --- | --- | --- | --- | --- | --- | --- | --- | --- |
| Deaths | North Africa and Middle East | Male | 10-19 years | CKD-T1DM | Environmental/occupational risks | Percent | 2021 | 0.097655 | 0.15643 | 0.051623 |
| Deaths | North Africa and Middle East | Both | 10-19 years | CKD-T1DM | Environmental/occupational risks | Percent | 2021 | 0.096901 | 0.154584 | 0.052038 |
| Deaths | North Africa and Middle East | Female | 10-19 years | CKD-T1DM | Environmental/occupational risks | Percent | 2021 | 0.096332 | 0.152306 | 0.052544 |
| Deaths | South Asia | Female | 10-19 years | CKD-T1DM | Environmental/occupational risks | Percent | 2021 | 0.093139 | 0.162428 | 0.028925 |
| Deaths | Central Asia | Female | 10-19 years | CKD-T1DM | Environmental/occupational risks | Percent | 2021 | 0.092706 | 0.117388 | 0.066941 |
| Deaths | Central Asia | Both | 10-19 years | CKD-T1DM | Environmental/occupational risks | Percent | 2021 | 0.092454 | 0.11679 | 0.067119 |
| Deaths | Central Europe | Male | 10-19 years | CKD-T1DM | Environmental/occupational risks | Percent | 2021 | 0.092346 | 0.104699 | 0.079161 |
| Deaths | Central Europe | Both | 10-19 years | CKD-T1DM | Environmental/occupational risks | Percent | 2021 | 0.092313 | 0.103964 | 0.079433 |
| Deaths | Central Europe | Female | 10-19 years | CKD-T1DM | Environmental/occupational risks | Percent | 2021 | 0.09229 | 0.104054 | 0.079462 |
| Deaths | Central Asia | Male | 10-19 years | CKD-T1DM | Environmental/occupational risks | Percent | 2021 | 0.092234 | 0.115993 | 0.067089 |
| Deaths | Central Europe | Female | 10-19 years | CKD-T1DM | Low temperature | Percent | 2021 | 0.092002 | 0.105145 | 0.083499 |
| Deaths | Central Europe | Both | 10-19 years | CKD-T1DM | Low temperature | Percent | 2021 | 0.091978 | 0.105536 | 0.083218 |
| Deaths | Central Europe | Male | 10-19 years | CKD-T1DM | Low temperature | Percent | 2021 | 0.091973 | 0.105784 | 0.082926 |
| Deaths | South Asia | Both | 10-19 years | CKD-T1DM | Environmental/occupational risks | Percent | 2021 | 0.088724 | 0.154981 | 0.027971 |
| Deaths | Western Europe | Female | 10-19 years | CKD-T1DM | Environmental/occupational risks | Percent | 2021 | 0.088615 | 0.104795 | 0.078991 |
| Deaths | Western Europe | Both | 10-19 years | CKD-T1DM | Environmental/occupational risks | Percent | 2021 | 0.088109 | 0.104378 | 0.078486 |
| Deaths | Western Europe | Male | 10-19 years | CKD-T1DM | Environmental/occupational risks | Percent | 2021 | 0.087561 | 0.103508 | 0.0776 |
| Deaths | Western Europe | Female | 10-19 years | CKD-T1DM | Low temperature | Percent | 2021 | 0.087502 | 0.104964 | 0.078085 |
| Deaths | Western Europe | Both | 10-19 years | CKD-T1DM | Low temperature | Percent | 2021 | 0.086909 | 0.104553 | 0.078222 |
| Deaths | Western Europe | Male | 10-19 years | CKD-T1DM | Low temperature | Percent | 2021 | 0.086269 | 0.104114 | 0.077484 |
| Deaths | South Asia | Male | 10-19 years | CKD-T1DM | Environmental/occupational risks | Percent | 2021 | 0.084765 | 0.148321 | 0.02714 |
| Deaths | Central Asia | Female | 10-19 years | CKD-T1DM | Low temperature | Percent | 2021 | 0.082854 | 0.091891 | 0.069675 |
| Deaths | Central Asia | Both | 10-19 years | CKD-T1DM | Low temperature | Percent | 2021 | 0.082797 | 0.09166 | 0.069597 |
| Deaths | Central Asia | Male | 10-19 years | CKD-T1DM | Low temperature | Percent | 2021 | 0.082756 | 0.091694 | 0.069696 |
| Deaths | High SDI | Female | 10-19 years | CKD-T1DM | Environmental/occupational risks | Percent | 2021 | 0.081874 | 0.119822 | 0.050177 |
| Deaths | High SDI | Both | 10-19 years | CKD-T1DM | Environmental/occupational risks | Percent | 2021 | 0.08069 | 0.114929 | 0.050577 |
| Deaths | High-income Asia Pacific | Male | 10-19 years | CKD-T1DM | Environmental/occupational risks | Percent | 2021 | 0.079721 | 0.088946 | 0.068958 |
| Deaths | High SDI | Male | 10-19 years | CKD-T1DM | Environmental/occupational risks | Percent | 2021 | 0.079613 | 0.111642 | 0.051013 |
| Deaths | High-income Asia Pacific | Both | 10-19 years | CKD-T1DM | Environmental/occupational risks | Percent | 2021 | 0.078851 | 0.087975 | 0.068161 |
| Deaths | East Asia | Male | 10-19 years | CKD-T1DM | Environmental/occupational risks | Percent | 2021 | 0.078755 | 0.094066 | 0.06242 |
| Deaths | East Asia | Both | 10-19 years | CKD-T1DM | Environmental/occupational risks | Percent | 2021 | 0.078581 | 0.094465 | 0.062089 |
| Deaths | East Asia | Female | 10-19 years | CKD-T1DM | Environmental/occupational risks | Percent | 2021 | 0.078325 | 0.094142 | 0.061999 |
| Deaths | Australasia | Female | 10-19 years | CKD-T1DM | Environmental/occupational risks | Percent | 2021 | 0.078218 | 0.098792 | 0.067532 |
| Deaths | High-income Asia Pacific | Female | 10-19 years | CKD-T1DM | Environmental/occupational risks | Percent | 2021 | 0.078142 | 0.08661 | 0.067884 |
| Deaths | High-income Asia Pacific | Male | 10-19 years | CKD-T1DM | Low temperature | Percent | 2021 | 0.077973 | 0.088792 | 0.070737 |
| Deaths | Southern Latin America | Female | 10-19 years | CKD-T1DM | Environmental/occupational risks | Percent | 2021 | 0.077823 | 0.084794 | 0.072057 |
| Deaths | Australasia | Female | 10-19 years | CKD-T1DM | Low temperature | Percent | 2021 | 0.077712 | 0.098801 | 0.066494 |
| Deaths | Southern Latin America | Both | 10-19 years | CKD-T1DM | Environmental/occupational risks | Percent | 2021 | 0.077684 | 0.0843 | 0.072127 |
| Deaths | Southern Latin America | Male | 10-19 years | CKD-T1DM | Environmental/occupational risks | Percent | 2021 | 0.077567 | 0.083984 | 0.071884 |
| Deaths | Australasia | Both | 10-19 years | CKD-T1DM | Environmental/occupational risks | Percent | 2021 | 0.077428 | 0.096485 | 0.066796 |
| Deaths | High-income North America | Female | 10-19 years | CKD-T1DM | Environmental/occupational risks | Percent | 2021 | 0.077193 | 0.093731 | 0.060405 |
| Deaths | High-income North America | Both | 10-19 years | CKD-T1DM | Environmental/occupational risks | Percent | 2021 | 0.0771 | 0.093494 | 0.060588 |
| Deaths | High-income Asia Pacific | Both | 10-19 years | CKD-T1DM | Low temperature | Percent | 2021 | 0.077076 | 0.087691 | 0.070461 |
| Deaths | High-income North America | Male | 10-19 years | CKD-T1DM | Environmental/occupational risks | Percent | 2021 | 0.076999 | 0.09344 | 0.060307 |
| Deaths | Australasia | Both | 10-19 years | CKD-T1DM | Low temperature | Percent | 2021 | 0.076858 | 0.096486 | 0.06566 |
| Deaths | Southern Latin America | Female | 10-19 years | CKD-T1DM | Low temperature | Percent | 2021 | 0.076478 | 0.084855 | 0.07189 |
| Deaths | High-income Asia Pacific | Female | 10-19 years | CKD-T1DM | Low temperature | Percent | 2021 | 0.076344 | 0.086806 | 0.070009 |
| Deaths | Southern Latin America | Both | 10-19 years | CKD-T1DM | Low temperature | Percent | 2021 | 0.076334 | 0.084162 | 0.071978 |
| Deaths | Australasia | Male | 10-19 years | CKD-T1DM | Environmental/occupational risks | Percent | 2021 | 0.076284 | 0.093898 | 0.065742 |
| Deaths | Southern Latin America | Male | 10-19 years | CKD-T1DM | Low temperature | Percent | 2021 | 0.076213 | 0.083784 | 0.071883 |
| Deaths | Australasia | Male | 10-19 years | CKD-T1DM | Low temperature | Percent | 2021 | 0.075623 | 0.094317 | 0.06413 |
| Deaths | East Asia | Male | 10-19 years | CKD-T1DM | Low temperature | Percent | 2021 | 0.073086 | 0.079516 | 0.065428 |
| Deaths | East Asia | Both | 10-19 years | CKD-T1DM | Low temperature | Percent | 2021 | 0.072961 | 0.07918 | 0.065324 |
| Deaths | Eastern Europe | Male | 10-19 years | CKD-T1DM | Environmental/occupational risks | Percent | 2021 | 0.072925 | 0.10011 | 0.044531 |
| Deaths | High-income North America | Female | 10-19 years | CKD-T1DM | Low temperature | Percent | 2021 | 0.07286 | 0.080069 | 0.064843 |
| Deaths | East Asia | Female | 10-19 years | CKD-T1DM | Low temperature | Percent | 2021 | 0.072778 | 0.079014 | 0.064453 |
| Deaths | Eastern Europe | Both | 10-19 years | CKD-T1DM | Environmental/occupational risks | Percent | 2021 | 0.072668 | 0.100409 | 0.044105 |
| Deaths | High-income North America | Both | 10-19 years | CKD-T1DM | Low temperature | Percent | 2021 | 0.072665 | 0.080061 | 0.064907 |
| Deaths | Eastern Europe | Female | 10-19 years | CKD-T1DM | Environmental/occupational risks | Percent | 2021 | 0.072513 | 0.10014 | 0.044166 |
| Deaths | High-income North America | Male | 10-19 years | CKD-T1DM | Low temperature | Percent | 2021 | 0.072442 | 0.079915 | 0.064532 |
| Deaths | Eastern Europe | Male | 10-19 years | CKD-T1DM | Low temperature | Percent | 2021 | 0.072112 | 0.092576 | 0.05014 |
| Deaths | Eastern Europe | Both | 10-19 years | CKD-T1DM | Low temperature | Percent | 2021 | 0.071823 | 0.093146 | 0.050075 |
| Deaths | Eastern Europe | Female | 10-19 years | CKD-T1DM | Low temperature | Percent | 2021 | 0.071646 | 0.093534 | 0.049904 |
| Deaths | High SDI | Male | 10-19 years | CKD-T1DM | Low temperature | Percent | 2021 | 0.064546 | 0.071829 | 0.055692 |
| Deaths | Southern Sub-Saharan Africa | Male | 10-19 years | CKD-T1DM | Environmental/occupational risks | Percent | 2021 | 0.063573 | 0.071018 | 0.055561 |
| Deaths | High SDI | Both | 10-19 years | CKD-T1DM | Low temperature | Percent | 2021 | 0.063474 | 0.070718 | 0.05411 |
| Deaths | Andean Latin America | Female | 10-19 years | CKD-T1DM | Environmental/occupational risks | Percent | 2021 | 0.063196 | 0.079115 | 0.054412 |
| Deaths | Andean Latin America | Female | 10-19 years | CKD-T1DM | Low temperature | Percent | 2021 | 0.062568 | 0.079891 | 0.052937 |
| Deaths | Southern Sub-Saharan Africa | Male | 10-19 years | CKD-T1DM | Low temperature | Percent | 2021 | 0.062376 | 0.07001 | 0.055261 |
| Deaths | Andean Latin America | Both | 10-19 years | CKD-T1DM | Environmental/occupational risks | Percent | 2021 | 0.062324 | 0.077645 | 0.054082 |
| Deaths | Southern Sub-Saharan Africa | Both | 10-19 years | CKD-T1DM | Environmental/occupational risks | Percent | 2021 | 0.062235 | 0.070271 | 0.053853 |
| Deaths | High SDI | Female | 10-19 years | CKD-T1DM | Low temperature | Percent | 2021 | 0.062201 | 0.070534 | 0.052142 |
| Deaths | North Africa and Middle East | Female | 10-19 years | CKD-T1DM | Low temperature | Percent | 2021 | 0.061795 | 0.07405 | 0.050112 |
| Deaths | Andean Latin America | Both | 10-19 years | CKD-T1DM | Low temperature | Percent | 2021 | 0.061734 | 0.078294 | 0.052593 |
| Deaths | North Africa and Middle East | Both | 10-19 years | CKD-T1DM | Low temperature | Percent | 2021 | 0.061703 | 0.074681 | 0.049867 |
| Deaths | Andean Latin America | Male | 10-19 years | CKD-T1DM | Environmental/occupational risks | Percent | 2021 | 0.061683 | 0.07584 | 0.053209 |
| Deaths | North Africa and Middle East | Male | 10-19 years | CKD-T1DM | Low temperature | Percent | 2021 | 0.061594 | 0.075425 | 0.04892 |
| Deaths | Andean Latin America | Male | 10-19 years | CKD-T1DM | Low temperature | Percent | 2021 | 0.061121 | 0.07635 | 0.051607 |
| Deaths | Southern Sub-Saharan Africa | Both | 10-19 years | CKD-T1DM | Low temperature | Percent | 2021 | 0.060918 | 0.068403 | 0.053905 |
| Deaths | Southern Sub-Saharan Africa | Female | 10-19 years | CKD-T1DM | Environmental/occupational risks | Percent | 2021 | 0.059903 | 0.069028 | 0.050323 |
| Deaths | Southern Sub-Saharan Africa | Female | 10-19 years | CKD-T1DM | Low temperature | Percent | 2021 | 0.05837 | 0.066797 | 0.051418 |
| Deaths | South Asia | Female | 10-19 years | CKD-T1DM | Low temperature | Percent | 2021 | 0.057128 | 0.087189 | 0.026838 |
| Deaths | South Asia | Both | 10-19 years | CKD-T1DM | Low temperature | Percent | 2021 | 0.054623 | 0.083151 | 0.026345 |
| Deaths | South Asia | Male | 10-19 years | CKD-T1DM | Low temperature | Percent | 2021 | 0.052378 | 0.080749 | 0.026422 |
| Deaths | High-middle SDI | Male | 10-19 years | CKD-T1DM | Environmental/occupational risks | Percent | 2021 | 0.05068 | 0.067967 | 0.036013 |
| Deaths | High-middle SDI | Both | 10-19 years | CKD-T1DM | Environmental/occupational risks | Percent | 2021 | 0.050569 | 0.066769 | 0.035909 |
| Deaths | High-middle SDI | Female | 10-19 years | CKD-T1DM | Environmental/occupational risks | Percent | 2021 | 0.050347 | 0.067581 | 0.035401 |
| Deaths | Central Latin America | Male | 10-19 years | CKD-T1DM | Environmental/occupational risks | Percent | 2021 | 0.047989 | 0.055784 | 0.04295 |
| Deaths | Central Latin America | Both | 10-19 years | CKD-T1DM | Environmental/occupational risks | Percent | 2021 | 0.047831 | 0.055501 | 0.043005 |
| Deaths | Central Latin America | Female | 10-19 years | CKD-T1DM | Environmental/occupational risks | Percent | 2021 | 0.047638 | 0.055408 | 0.042303 |
| Deaths | Low SDI | Female | 10-19 years | CKD-T1DM | Environmental/occupational risks | Percent | 2021 | 0.045342 | 0.058962 | 0.034534 |
| Deaths | High-middle SDI | Male | 10-19 years | CKD-T1DM | Low temperature | Percent | 2021 | 0.044853 | 0.053449 | 0.037023 |
| Deaths | High-middle SDI | Both | 10-19 years | CKD-T1DM | Low temperature | Percent | 2021 | 0.044752 | 0.052145 | 0.037679 |
| Deaths | Central Latin America | Male | 10-19 years | CKD-T1DM | Low temperature | Percent | 2021 | 0.044623 | 0.055321 | 0.039156 |
| Deaths | High-middle SDI | Female | 10-19 years | CKD-T1DM | Low temperature | Percent | 2021 | 0.044548 | 0.052303 | 0.036789 |
| Deaths | Central Latin America | Both | 10-19 years | CKD-T1DM | Low temperature | Percent | 2021 | 0.044408 | 0.055305 | 0.039205 |
| Deaths | Eastern Sub-Saharan Africa | Female | 10-19 years | CKD-T1DM | Environmental/occupational risks | Percent | 2021 | 0.044376 | 0.050641 | 0.037341 |
| Deaths | Central Latin America | Female | 10-19 years | CKD-T1DM | Low temperature | Percent | 2021 | 0.044153 | 0.054991 | 0.038482 |
| Deaths | Eastern Sub-Saharan Africa | Both | 10-19 years | CKD-T1DM | Environmental/occupational risks | Percent | 2021 | 0.043737 | 0.050025 | 0.037567 |
| Deaths | Eastern Sub-Saharan Africa | Male | 10-19 years | CKD-T1DM | Environmental/occupational risks | Percent | 2021 | 0.043421 | 0.049745 | 0.036955 |
| Deaths | Low SDI | Both | 10-19 years | CKD-T1DM | Environmental/occupational risks | Percent | 2021 | 0.043109 | 0.055494 | 0.033068 |
| Deaths | Low SDI | Male | 10-19 years | CKD-T1DM | Environmental/occupational risks | Percent | 2021 | 0.041985 | 0.054114 | 0.031826 |
| Deaths | Eastern Sub-Saharan Africa | Female | 10-19 years | CKD-T1DM | Low temperature | Percent | 2021 | 0.040336 | 0.049681 | 0.034413 |
| Deaths | Global | Male | 10-19 years | CKD-T1DM | Environmental/occupational risks | Percent | 2021 | 0.040065 | 0.053125 | 0.029509 |
| Deaths | Global | Both | 10-19 years | CKD-T1DM | Environmental/occupational risks | Percent | 2021 | 0.039808 | 0.053558 | 0.029043 |
| Deaths | Low-middle SDI | Female | 10-19 years | CKD-T1DM | Environmental/occupational risks | Percent | 2021 | 0.039791 | 0.065583 | 0.019886 |
| Deaths | Eastern Sub-Saharan Africa | Both | 10-19 years | CKD-T1DM | Low temperature | Percent | 2021 | 0.03979 | 0.048481 | 0.03412 |
| Deaths | Eastern Sub-Saharan Africa | Male | 10-19 years | CKD-T1DM | Low temperature | Percent | 2021 | 0.03951 | 0.048791 | 0.033426 |
| Deaths | Global | Female | 10-19 years | CKD-T1DM | Environmental/occupational risks | Percent | 2021 | 0.039491 | 0.054427 | 0.027982 |
| Deaths | South Asia | Female | 10-19 years | CKD-T1DM | High temperature | Percent | 2021 | 0.039263 | 0.084687 | 0.001498 |
| Deaths | Low-middle SDI | Both | 10-19 years | CKD-T1DM | Environmental/occupational risks | Percent | 2021 | 0.039049 | 0.062177 | 0.02071 |
| Deaths | North Africa and Middle East | Male | 10-19 years | CKD-T1DM | High temperature | Percent | 2021 | 0.038668 | 0.089359 | 0.001556 |
| Deaths | Low-middle SDI | Male | 10-19 years | CKD-T1DM | Environmental/occupational risks | Percent | 2021 | 0.038425 | 0.061557 | 0.021039 |
| Deaths | North Africa and Middle East | Both | 10-19 years | CKD-T1DM | High temperature | Percent | 2021 | 0.037697 | 0.087159 | 0.001892 |
| Deaths | Middle SDI | Male | 10-19 years | CKD-T1DM | Environmental/occupational risks | Percent | 2021 | 0.037343 | 0.047241 | 0.028624 |
| Deaths | South Asia | Both | 10-19 years | CKD-T1DM | High temperature | Percent | 2021 | 0.037078 | 0.080008 | 0.001397 |
| Deaths | Western Sub-Saharan Africa | Female | 10-19 years | CKD-T1DM | Environmental/occupational risks | Percent | 2021 | 0.037001 | 0.067299 | 0.018041 |
| Deaths | North Africa and Middle East | Female | 10-19 years | CKD-T1DM | High temperature | Percent | 2021 | 0.036952 | 0.086228 | 0.001796 |
| Deaths | Middle SDI | Both | 10-19 years | CKD-T1DM | Environmental/occupational risks | Percent | 2021 | 0.03628 | 0.045151 | 0.028463 |
| Deaths | Low SDI | Female | 10-19 years | CKD-T1DM | Low temperature | Percent | 2021 | 0.035428 | 0.04043 | 0.030045 |
| Deaths | South Asia | Male | 10-19 years | CKD-T1DM | High temperature | Percent | 2021 | 0.035116 | 0.075707 | 0.001419 |
| Deaths | Middle SDI | Female | 10-19 years | CKD-T1DM | Environmental/occupational risks | Percent | 2021 | 0.034923 | 0.044065 | 0.027196 |
| Deaths | Western Sub-Saharan Africa | Both | 10-19 years | CKD-T1DM | Environmental/occupational risks | Percent | 2021 | 0.034883 | 0.062443 | 0.01643 |
| Deaths | Western Sub-Saharan Africa | Male | 10-19 years | CKD-T1DM | Environmental/occupational risks | Percent | 2021 | 0.034142 | 0.061386 | 0.016013 |
| Deaths | Low SDI | Both | 10-19 years | CKD-T1DM | Low temperature | Percent | 2021 | 0.033296 | 0.038676 | 0.028358 |
| Deaths | Low SDI | Male | 10-19 years | CKD-T1DM | Low temperature | Percent | 2021 | 0.032206 | 0.038261 | 0.026916 |
| Deaths | Global | Male | 10-19 years | CKD-T1DM | Low temperature | Percent | 2021 | 0.03139 | 0.036794 | 0.026697 |
| Deaths | Middle SDI | Male | 10-19 years | CKD-T1DM | Low temperature | Percent | 2021 | 0.031351 | 0.037854 | 0.025855 |
| Deaths | Global | Both | 10-19 years | CKD-T1DM | Low temperature | Percent | 2021 | 0.030822 | 0.035589 | 0.026424 |
| Deaths | Middle SDI | Both | 10-19 years | CKD-T1DM | Low temperature | Percent | 2021 | 0.030264 | 0.03533 | 0.025804 |
| Deaths | Global | Female | 10-19 years | CKD-T1DM | Low temperature | Percent | 2021 | 0.030078 | 0.035484 | 0.02543 |
| Deaths | Oceania | Male | 10-19 years | CKD-T1DM | Environmental/occupational risks | Percent | 2021 | 0.02918 | 0.036978 | 0.021983 |
| Deaths | Western Sub-Saharan Africa | Female | 10-19 years | CKD-T1DM | High temperature | Percent | 2021 | 0.029008 | 0.054699 | 0.013002 |
| Deaths | Middle SDI | Female | 10-19 years | CKD-T1DM | Low temperature | Percent | 2021 | 0.028871 | 0.033573 | 0.02444 |
| Deaths | Oceania | Male | 10-19 years | CKD-T1DM | Low temperature | Percent | 2021 | 0.028754 | 0.037046 | 0.021578 |
| Deaths | Oceania | Both | 10-19 years | CKD-T1DM | Environmental/occupational risks | Percent | 2021 | 0.028537 | 0.035908 | 0.021867 |
| Deaths | Oceania | Both | 10-19 years | CKD-T1DM | Low temperature | Percent | 2021 | 0.028104 | 0.036487 | 0.021091 |
| Deaths | Oceania | Female | 10-19 years | CKD-T1DM | Environmental/occupational risks | Percent | 2021 | 0.02785 | 0.035728 | 0.02037 |
| Deaths | Oceania | Female | 10-19 years | CKD-T1DM | Low temperature | Percent | 2021 | 0.027412 | 0.03529 | 0.019908 |
| Deaths | Western Sub-Saharan Africa | Both | 10-19 years | CKD-T1DM | High temperature | Percent | 2021 | 0.027037 | 0.050359 | 0.012061 |
| Deaths | Western Sub-Saharan Africa | Male | 10-19 years | CKD-T1DM | High temperature | Percent | 2021 | 0.026351 | 0.048747 | 0.011659 |
| Deaths | Low-middle SDI | Male | 10-19 years | CKD-T1DM | Low temperature | Percent | 2021 | 0.023898 | 0.033451 | 0.017029 |
| Deaths | Low-middle SDI | Both | 10-19 years | CKD-T1DM | Low temperature | Percent | 2021 | 0.023753 | 0.032666 | 0.016607 |
| Deaths | Tropical Latin America | Female | 10-19 years | CKD-T1DM | Environmental/occupational risks | Percent | 2021 | 0.023671 | 0.029761 | 0.019354 |
| Deaths | Low-middle SDI | Female | 10-19 years | CKD-T1DM | Low temperature | Percent | 2021 | 0.023638 | 0.032834 | 0.016089 |
| Deaths | Tropical Latin America | Both | 10-19 years | CKD-T1DM | Environmental/occupational risks | Percent | 2021 | 0.022925 | 0.028924 | 0.018774 |
| Deaths | Tropical Latin America | Male | 10-19 years | CKD-T1DM | Environmental/occupational risks | Percent | 2021 | 0.0225 | 0.028737 | 0.018296 |
| Deaths | Tropical Latin America | Female | 10-19 years | CKD-T1DM | Low temperature | Percent | 2021 | 0.022476 | 0.025298 | 0.019175 |
| Deaths | Tropical Latin America | Both | 10-19 years | CKD-T1DM | Low temperature | Percent | 2021 | 0.021733 | 0.024687 | 0.01855 |
| Deaths | Tropical Latin America | Male | 10-19 years | CKD-T1DM | Low temperature | Percent | 2021 | 0.02131 | 0.024336 | 0.018278 |
| Deaths | High SDI | Female | 10-19 years | CKD-T1DM | High temperature | Percent | 2021 | 0.021036 | 0.054358 | -0.00416 |
| Deaths | High SDI | Both | 10-19 years | CKD-T1DM | High temperature | Percent | 2021 | 0.018409 | 0.048729 | -0.00514 |
| Deaths | Low-middle SDI | Female | 10-19 years | CKD-T1DM | High temperature | Percent | 2021 | 0.017015 | 0.03487 | 0.004003 |
| Deaths | High SDI | Male | 10-19 years | CKD-T1DM | High temperature | Percent | 2021 | 0.016111 | 0.04485 | -0.00639 |
| Deaths | Low-middle SDI | Both | 10-19 years | CKD-T1DM | High temperature | Percent | 2021 | 0.016083 | 0.032578 | 0.003618 |
| Deaths | Low-middle SDI | Male | 10-19 years | CKD-T1DM | High temperature | Percent | 2021 | 0.015247 | 0.031292 | 0.003361 |
| Deaths | Southeast Asia | Female | 10-19 years | CKD-T1DM | Environmental/occupational risks | Percent | 2021 | 0.014977 | 0.021702 | 0.010638 |
| Deaths | Southeast Asia | Both | 10-19 years | CKD-T1DM | Environmental/occupational risks | Percent | 2021 | 0.014434 | 0.020801 | 0.010501 |
| Deaths | Southeast Asia | Male | 10-19 years | CKD-T1DM | Environmental/occupational risks | Percent | 2021 | 0.013947 | 0.020308 | 0.010079 |
| Deaths | Central Sub-Saharan Africa | Female | 10-19 years | CKD-T1DM | Environmental/occupational risks | Percent | 2021 | 0.013891 | 0.01907 | 0.009202 |
| Deaths | Central Sub-Saharan Africa | Both | 10-19 years | CKD-T1DM | Environmental/occupational risks | Percent | 2021 | 0.013881 | 0.019163 | 0.009102 |
| Deaths | Central Sub-Saharan Africa | Male | 10-19 years | CKD-T1DM | Environmental/occupational risks | Percent | 2021 | 0.013876 | 0.019102 | 0.009085 |
| Deaths | Central Sub-Saharan Africa | Female | 10-19 years | CKD-T1DM | Low temperature | Percent | 2021 | 0.013173 | 0.016114 | 0.010617 |
| Deaths | Central Sub-Saharan Africa | Both | 10-19 years | CKD-T1DM | Low temperature | Percent | 2021 | 0.013128 | 0.015946 | 0.010765 |
| Deaths | Central Sub-Saharan Africa | Male | 10-19 years | CKD-T1DM | Low temperature | Percent | 2021 | 0.013107 | 0.015962 | 0.010766 |
| Deaths | Central Asia | Female | 10-19 years | CKD-T1DM | High temperature | Percent | 2021 | 0.010804 | 0.027732 | -0.00479 |
| Deaths | Central Asia | Both | 10-19 years | CKD-T1DM | High temperature | Percent | 2021 | 0.010589 | 0.02717 | -0.00492 |
| Deaths | Central Asia | Male | 10-19 years | CKD-T1DM | High temperature | Percent | 2021 | 0.010393 | 0.026681 | -0.00506 |
| Deaths | Low SDI | Female | 10-19 years | CKD-T1DM | High temperature | Percent | 2021 | 0.010205 | 0.022086 | 0.001837 |
| Deaths | Low SDI | Both | 10-19 years | CKD-T1DM | High temperature | Percent | 2021 | 0.010057 | 0.021744 | 0.002019 |
| Deaths | Low SDI | Male | 10-19 years | CKD-T1DM | High temperature | Percent | 2021 | 0.01 | 0.021555 | 0.002051 |
| Deaths | Global | Female | 10-19 years | CKD-T1DM | High temperature | Percent | 2021 | 0.009839 | 0.021134 | 0.001547 |
| Deaths | Global | Both | 10-19 years | CKD-T1DM | High temperature | Percent | 2021 | 0.009366 | 0.02032 | 0.001405 |
| Deaths | Global | Male | 10-19 years | CKD-T1DM | High temperature | Percent | 2021 | 0.009022 | 0.019869 | 0.001113 |
| Deaths | Western Sub-Saharan Africa | Female | 10-19 years | CKD-T1DM | Low temperature | Percent | 2021 | 0.00832 | 0.013577 | 0.004766 |
| Deaths | Western Sub-Saharan Africa | Both | 10-19 years | CKD-T1DM | Low temperature | Percent | 2021 | 0.008155 | 0.013198 | 0.00462 |
| Deaths | Western Sub-Saharan Africa | Male | 10-19 years | CKD-T1DM | Low temperature | Percent | 2021 | 0.008095 | 0.0131 | 0.004562 |
| Deaths | Southeast Asia | Female | 10-19 years | CKD-T1DM | Low temperature | Percent | 2021 | 0.007985 | 0.010077 | 0.005939 |
| Deaths | Southeast Asia | Both | 10-19 years | CKD-T1DM | Low temperature | Percent | 2021 | 0.007745 | 0.009673 | 0.005646 |
| Deaths | Southeast Asia | Male | 10-19 years | CKD-T1DM | Low temperature | Percent | 2021 | 0.007525 | 0.009421 | 0.005524 |
| Deaths | Caribbean | Male | 10-19 years | CKD-T1DM | Environmental/occupational risks | Percent | 2021 | 0.007215 | 0.010367 | 0.00448 |
| Deaths | Southeast Asia | Female | 10-19 years | CKD-T1DM | High temperature | Percent | 2021 | 0.007081 | 0.012238 | 0.004057 |
| Deaths | Caribbean | Both | 10-19 years | CKD-T1DM | Environmental/occupational risks | Percent | 2021 | 0.006956 | 0.010083 | 0.004363 |
| Deaths | Southeast Asia | Both | 10-19 years | CKD-T1DM | High temperature | Percent | 2021 | 0.006774 | 0.011454 | 0.00398 |
| Deaths | Southeast Asia | Male | 10-19 years | CKD-T1DM | High temperature | Percent | 2021 | 0.006503 | 0.011116 | 0.003803 |
| Deaths | Middle SDI | Female | 10-19 years | CKD-T1DM | High temperature | Percent | 2021 | 0.006285 | 0.013403 | 0.001174 |
| Deaths | Middle SDI | Both | 10-19 years | CKD-T1DM | High temperature | Percent | 2021 | 0.006249 | 0.013489 | 0.000943 |
| Deaths | Middle SDI | Male | 10-19 years | CKD-T1DM | High temperature | Percent | 2021 | 0.006226 | 0.013581 | 0.000889 |
| Deaths | Caribbean | Female | 10-19 years | CKD-T1DM | Environmental/occupational risks | Percent | 2021 | 0.006199 | 0.009613 | 0.003691 |
| Deaths | High-middle SDI | Male | 10-19 years | CKD-T1DM | High temperature | Percent | 2021 | 0.006107 | 0.018466 | -0.00424 |
| Deaths | High-middle SDI | Both | 10-19 years | CKD-T1DM | High temperature | Percent | 2021 | 0.006102 | 0.018721 | -0.00405 |
| Deaths | High-middle SDI | Female | 10-19 years | CKD-T1DM | High temperature | Percent | 2021 | 0.006088 | 0.018267 | -0.0038 |
| Deaths | East Asia | Male | 10-19 years | CKD-T1DM | High temperature | Percent | 2021 | 0.006049 | 0.020499 | -0.00663 |
| Deaths | East Asia | Both | 10-19 years | CKD-T1DM | High temperature | Percent | 2021 | 0.005996 | 0.020761 | -0.00648 |
| Deaths | East Asia | Female | 10-19 years | CKD-T1DM | High temperature | Percent | 2021 | 0.005915 | 0.020257 | -0.00633 |
| Deaths | Caribbean | Male | 10-19 years | CKD-T1DM | Low temperature | Percent | 2021 | 0.005801 | 0.006922 | 0.004478 |
| Deaths | Caribbean | Both | 10-19 years | CKD-T1DM | Low temperature | Percent | 2021 | 0.00558 | 0.006559 | 0.004345 |
| Deaths | Caribbean | Female | 10-19 years | CKD-T1DM | Low temperature | Percent | 2021 | 0.004908 | 0.005809 | 0.003997 |
| Deaths | High-income North America | Male | 10-19 years | CKD-T1DM | High temperature | Percent | 2021 | 0.004867 | 0.019367 | -0.00649 |
| Deaths | High-income North America | Both | 10-19 years | CKD-T1DM | High temperature | Percent | 2021 | 0.004739 | 0.019012 | -0.00628 |
| Deaths | High-income North America | Female | 10-19 years | CKD-T1DM | High temperature | Percent | 2021 | 0.004631 | 0.018715 | -0.00622 |
| Deaths | Eastern Sub-Saharan Africa | Female | 10-19 years | CKD-T1DM | High temperature | Percent | 2021 | 0.004113 | 0.009932 | 0.000222 |
| Deaths | Eastern Sub-Saharan Africa | Both | 10-19 years | CKD-T1DM | High temperature | Percent | 2021 | 0.004022 | 0.009696 | 2.31E-05 |
| Deaths | Eastern Sub-Saharan Africa | Male | 10-19 years | CKD-T1DM | High temperature | Percent | 2021 | 0.003988 | 0.009611 | -8.67E-05 |
| Deaths | Central Latin America | Female | 10-19 years | CKD-T1DM | High temperature | Percent | 2021 | 0.003646 | 0.00893 | -0.00037 |
| Deaths | Central Latin America | Both | 10-19 years | CKD-T1DM | High temperature | Percent | 2021 | 0.003583 | 0.008707 | -0.00038 |
| Deaths | Central Latin America | Male | 10-19 years | CKD-T1DM | High temperature | Percent | 2021 | 0.003525 | 0.008586 | -0.0004 |
| Deaths | High-income Asia Pacific | Female | 10-19 years | CKD-T1DM | High temperature | Percent | 2021 | 0.001921 | 0.009556 | -0.00523 |
| Deaths | High-income Asia Pacific | Both | 10-19 years | CKD-T1DM | High temperature | Percent | 2021 | 0.001899 | 0.009962 | -0.00555 |
| Deaths | High-income Asia Pacific | Male | 10-19 years | CKD-T1DM | High temperature | Percent | 2021 | 0.001872 | 0.01065 | -0.00588 |
| Deaths | Southern Sub-Saharan Africa | Female | 10-19 years | CKD-T1DM | High temperature | Percent | 2021 | 0.001603 | 0.007742 | -0.00311 |
| Deaths | Southern Latin America | Male | 10-19 years | CKD-T1DM | High temperature | Percent | 2021 | 0.001461 | 0.005868 | -0.00188 |
| Deaths | Southern Latin America | Both | 10-19 years | CKD-T1DM | High temperature | Percent | 2021 | 0.001458 | 0.005938 | -0.00189 |
| Deaths | Southern Latin America | Female | 10-19 years | CKD-T1DM | High temperature | Percent | 2021 | 0.001452 | 0.005916 | -0.00188 |
| Deaths | Caribbean | Male | 10-19 years | CKD-T1DM | High temperature | Percent | 2021 | 0.001424 | 0.003924 | -0.0006 |
| Deaths | Caribbean | Both | 10-19 years | CKD-T1DM | High temperature | Percent | 2021 | 0.001385 | 0.003932 | -0.00062 |
| Deaths | Southern Sub-Saharan Africa | Both | 10-19 years | CKD-T1DM | High temperature | Percent | 2021 | 0.001377 | 0.006831 | -0.00277 |
| Deaths | Western Europe | Male | 10-19 years | CKD-T1DM | High temperature | Percent | 2021 | 0.00136 | 0.006026 | -0.0023 |
| Deaths | Caribbean | Female | 10-19 years | CKD-T1DM | High temperature | Percent | 2021 | 0.001299 | 0.003943 | -0.00062 |
| Deaths | Western Europe | Both | 10-19 years | CKD-T1DM | High temperature | Percent | 2021 | 0.001265 | 0.005736 | -0.00221 |
| Deaths | Southern Sub-Saharan Africa | Male | 10-19 years | CKD-T1DM | High temperature | Percent | 2021 | 0.001252 | 0.006314 | -0.00256 |
| Deaths | Tropical Latin America | Female | 10-19 years | CKD-T1DM | High temperature | Percent | 2021 | 0.001239 | 0.005282 | -0.00158 |
| Deaths | Tropical Latin America | Both | 10-19 years | CKD-T1DM | High temperature | Percent | 2021 | 0.001234 | 0.005176 | -0.00143 |
| Deaths | Tropical Latin America | Male | 10-19 years | CKD-T1DM | High temperature | Percent | 2021 | 0.001232 | 0.005144 | -0.0014 |
| Deaths | Western Europe | Female | 10-19 years | CKD-T1DM | High temperature | Percent | 2021 | 0.001177 | 0.005499 | -0.00215 |
| Deaths | Eastern Europe | Female | 10-19 years | CKD-T1DM | High temperature | Percent | 2021 | 0.000955 | 0.007991 | -0.00612 |
| Deaths | Eastern Europe | Both | 10-19 years | CKD-T1DM | High temperature | Percent | 2021 | 0.000931 | 0.007906 | -0.00612 |
| Deaths | Eastern Europe | Male | 10-19 years | CKD-T1DM | High temperature | Percent | 2021 | 0.000895 | 0.007768 | -0.0061 |
| Deaths | Central Sub-Saharan Africa | Male | 10-19 years | CKD-T1DM | High temperature | Percent | 2021 | 0.000777 | 0.006187 | -0.00418 |
| Deaths | Central Sub-Saharan Africa | Both | 10-19 years | CKD-T1DM | High temperature | Percent | 2021 | 0.000761 | 0.006182 | -0.00406 |
| Deaths | Central Sub-Saharan Africa | Female | 10-19 years | CKD-T1DM | High temperature | Percent | 2021 | 0.000726 | 0.006115 | -0.00403 |
| Deaths | Australasia | Male | 10-19 years | CKD-T1DM | High temperature | Percent | 2021 | 0.000706 | 0.003182 | -0.00103 |
| Deaths | Andean Latin America | Female | 10-19 years | CKD-T1DM | High temperature | Percent | 2021 | 0.00067 | 0.002635 | -0.00085 |
| Deaths | Andean Latin America | Both | 10-19 years | CKD-T1DM | High temperature | Percent | 2021 | 0.00063 | 0.00252 | -0.00083 |
| Deaths | Australasia | Both | 10-19 years | CKD-T1DM | High temperature | Percent | 2021 | 0.000609 | 0.002666 | -0.00096 |
| Deaths | Andean Latin America | Male | 10-19 years | CKD-T1DM | High temperature | Percent | 2021 | 0.0006 | 0.002408 | -0.00085 |
| Deaths | Australasia | Female | 10-19 years | CKD-T1DM | High temperature | Percent | 2021 | 0.000541 | 0.002308 | -0.00089 |
| Deaths | Oceania | Female | 10-19 years | CKD-T1DM | High temperature | Percent | 2021 | 0.000448 | 0.000942 | -0.00033 |
| Deaths | Oceania | Both | 10-19 years | CKD-T1DM | High temperature | Percent | 2021 | 0.000444 | 0.000952 | -0.00032 |
| Deaths | Oceania | Male | 10-19 years | CKD-T1DM | High temperature | Percent | 2021 | 0.000438 | 0.000967 | -0.00031 |
| Deaths | Central Europe | Male | 10-19 years | CKD-T1DM | High temperature | Percent | 2021 | 0.000404 | 0.006249 | -0.0061 |
| Deaths | Central Europe | Both | 10-19 years | CKD-T1DM | High temperature | Percent | 2021 | 0.000363 | 0.006122 | -0.00608 |
| Deaths | Central Europe | Female | 10-19 years | CKD-T1DM | High temperature | Percent | 2021 | 0.000312 | 0.005966 | -0.00592 |

Appendix

**Appendix 24：Percentage contribution of risk factors to Chronic kidney disease due to diabetes mellitus type 1（CKD-T1DM） in 2019**

| **measure** | **location** | **sex** | **age** | **cause** | **rei** | **metric** | **year** | **val** | **upper** | **lower** |
| --- | --- | --- | --- | --- | --- | --- | --- | --- | --- | --- |
| DALYs | North Africa and Middle East | Female | 10-19 years | CKD-T1DM | Environmental/occupational risks | Percent | 2021 | 0.078392 | 0.125141 | 0.042088 |
| DALYs | East Asia | Male | 10-19 years | CKD-T1DM | Environmental/occupational risks | Percent | 2021 | 0.07773 | 0.092818 | 0.06154 |
| DALYs | East Asia | Both | 10-19 years | CKD-T1DM | Environmental/occupational risks | Percent | 2021 | 0.077449 | 0.092737 | 0.061337 |
| DALYs | East Asia | Female | 10-19 years | CKD-T1DM | Environmental/occupational risks | Percent | 2021 | 0.077019 | 0.091839 | 0.061233 |
| DALYs | North Africa and Middle East | Both | 10-19 years | CKD-T1DM | Environmental/occupational risks | Percent | 2021 | 0.076455 | 0.123757 | 0.03929 |
| DALYs | North Africa and Middle East | Male | 10-19 years | CKD-T1DM | Environmental/occupational risks | Percent | 2021 | 0.074237 | 0.120922 | 0.036956 |
| DALYs | South Asia | Female | 10-19 years | CKD-T1DM | Environmental/occupational risks | Percent | 2021 | 0.072845 | 0.129364 | 0.022976 |
| DALYs | East Asia | Male | 10-19 years | CKD-T1DM | Low temperature | Percent | 2021 | 0.072137 | 0.07866 | 0.064573 |
| DALYs | East Asia | Both | 10-19 years | CKD-T1DM | Low temperature | Percent | 2021 | 0.071912 | 0.078322 | 0.064143 |
| DALYs | East Asia | Female | 10-19 years | CKD-T1DM | Low temperature | Percent | 2021 | 0.071567 | 0.077799 | 0.063306 |
| DALYs | South Asia | Both | 10-19 years | CKD-T1DM | Environmental/occupational risks | Percent | 2021 | 0.068468 | 0.120101 | 0.022142 |
| DALYs | South Asia | Male | 10-19 years | CKD-T1DM | Environmental/occupational risks | Percent | 2021 | 0.064522 | 0.114188 | 0.020955 |
| DALYs | Andean Latin America | Female | 10-19 years | CKD-T1DM | Environmental/occupational risks | Percent | 2021 | 0.059663 | 0.075082 | 0.047914 |
| DALYs | Andean Latin America | Both | 10-19 years | CKD-T1DM | Environmental/occupational risks | Percent | 2021 | 0.059097 | 0.07488 | 0.048536 |
| DALYs | Andean Latin America | Female | 10-19 years | CKD-T1DM | Low temperature | Percent | 2021 | 0.059072 | 0.075671 | 0.046612 |
| DALYs | Andean Latin America | Male | 10-19 years | CKD-T1DM | Environmental/occupational risks | Percent | 2021 | 0.058651 | 0.0737 | 0.048645 |
| DALYs | Andean Latin America | Both | 10-19 years | CKD-T1DM | Low temperature | Percent | 2021 | 0.058539 | 0.075491 | 0.047007 |
| DALYs | Andean Latin America | Male | 10-19 years | CKD-T1DM | Low temperature | Percent | 2021 | 0.058118 | 0.074261 | 0.046993 |
| DALYs | Southern Latin America | Male | 10-19 years | CKD-T1DM | Environmental/occupational risks | Percent | 2021 | 0.056751 | 0.070265 | 0.036331 |
| DALYs | Southern Sub-Saharan Africa | Male | 10-19 years | CKD-T1DM | Environmental/occupational risks | Percent | 2021 | 0.056588 | 0.065038 | 0.046673 |
| DALYs | High SDI | Male | 10-19 years | CKD-T1DM | Environmental/occupational risks | Percent | 2021 | 0.0563 | 0.082229 | 0.034171 |
| DALYs | High SDI | Both | 10-19 years | CKD-T1DM | Environmental/occupational risks | Percent | 2021 | 0.056261 | 0.083066 | 0.033569 |
| DALYs | High SDI | Female | 10-19 years | CKD-T1DM | Environmental/occupational risks | Percent | 2021 | 0.056124 | 0.085487 | 0.032831 |
| DALYs | Southern Latin America | Male | 10-19 years | CKD-T1DM | Low temperature | Percent | 2021 | 0.055752 | 0.068672 | 0.036188 |
| DALYs | Southern Latin America | Both | 10-19 years | CKD-T1DM | Environmental/occupational risks | Percent | 2021 | 0.05564 | 0.069295 | 0.035194 |
| DALYs | Southern Sub-Saharan Africa | Male | 10-19 years | CKD-T1DM | Low temperature | Percent | 2021 | 0.05552 | 0.064227 | 0.046158 |
| DALYs | Southern Latin America | Both | 10-19 years | CKD-T1DM | Low temperature | Percent | 2021 | 0.054664 | 0.068247 | 0.03449 |
| DALYs | Southern Latin America | Female | 10-19 years | CKD-T1DM | Environmental/occupational risks | Percent | 2021 | 0.054608 | 0.068982 | 0.031929 |
| DALYs | Southern Sub-Saharan Africa | Both | 10-19 years | CKD-T1DM | Environmental/occupational risks | Percent | 2021 | 0.053911 | 0.06295 | 0.043179 |
| DALYs | Southern Latin America | Female | 10-19 years | CKD-T1DM | Low temperature | Percent | 2021 | 0.053655 | 0.068107 | 0.031734 |
| DALYs | Central Asia | Female | 10-19 years | CKD-T1DM | Environmental/occupational risks | Percent | 2021 | 0.052813 | 0.078787 | 0.026233 |
| DALYs | Southern Sub-Saharan Africa | Both | 10-19 years | CKD-T1DM | Low temperature | Percent | 2021 | 0.052772 | 0.061273 | 0.042582 |
| DALYs | Central Asia | Male | 10-19 years | CKD-T1DM | Environmental/occupational risks | Percent | 2021 | 0.052638 | 0.079634 | 0.027466 |
| DALYs | Central Asia | Both | 10-19 years | CKD-T1DM | Environmental/occupational risks | Percent | 2021 | 0.052557 | 0.078273 | 0.02925 |
| DALYs | North Africa and Middle East | Female | 10-19 years | CKD-T1DM | Low temperature | Percent | 2021 | 0.050297 | 0.062914 | 0.038002 |
| DALYs | Southern Sub-Saharan Africa | Female | 10-19 years | CKD-T1DM | Environmental/occupational risks | Percent | 2021 | 0.049509 | 0.059438 | 0.037426 |
| DALYs | North Africa and Middle East | Both | 10-19 years | CKD-T1DM | Low temperature | Percent | 2021 | 0.04868 | 0.061456 | 0.035576 |
| DALYs | High-income Asia Pacific | Female | 10-19 years | CKD-T1DM | Environmental/occupational risks | Percent | 2021 | 0.048555 | 0.061789 | 0.032679 |
| DALYs | Southern Sub-Saharan Africa | Female | 10-19 years | CKD-T1DM | Low temperature | Percent | 2021 | 0.048253 | 0.057675 | 0.035969 |
| DALYs | High-middle SDI | Male | 10-19 years | CKD-T1DM | Environmental/occupational risks | Percent | 2021 | 0.047575 | 0.063959 | 0.034035 |
| DALYs | High-income Asia Pacific | Female | 10-19 years | CKD-T1DM | Low temperature | Percent | 2021 | 0.047461 | 0.061026 | 0.032608 |
| DALYs | High-middle SDI | Both | 10-19 years | CKD-T1DM | Environmental/occupational risks | Percent | 2021 | 0.047289 | 0.062644 | 0.033436 |
| DALYs | Central Asia | Female | 10-19 years | CKD-T1DM | Low temperature | Percent | 2021 | 0.047251 | 0.067033 | 0.024135 |
| DALYs | Central Asia | Male | 10-19 years | CKD-T1DM | Low temperature | Percent | 2021 | 0.04725 | 0.067112 | 0.025565 |
| DALYs | Central Asia | Both | 10-19 years | CKD-T1DM | Low temperature | Percent | 2021 | 0.047104 | 0.066345 | 0.025487 |
| DALYs | High-income Asia Pacific | Both | 10-19 years | CKD-T1DM | Environmental/occupational risks | Percent | 2021 | 0.046832 | 0.060612 | 0.03119 |
| DALYs | North Africa and Middle East | Male | 10-19 years | CKD-T1DM | Low temperature | Percent | 2021 | 0.046807 | 0.060531 | 0.032417 |
| DALYs | High-middle SDI | Female | 10-19 years | CKD-T1DM | Environmental/occupational risks | Percent | 2021 | 0.046802 | 0.063321 | 0.033266 |
| DALYs | High-income Asia Pacific | Both | 10-19 years | CKD-T1DM | Low temperature | Percent | 2021 | 0.045801 | 0.060183 | 0.030855 |
| DALYs | High SDI | Male | 10-19 years | CKD-T1DM | Low temperature | Percent | 2021 | 0.045691 | 0.056241 | 0.034254 |
| DALYs | High-income Asia Pacific | Male | 10-19 years | CKD-T1DM | Environmental/occupational risks | Percent | 2021 | 0.044859 | 0.060216 | 0.02863 |
| DALYs | South Asia | Female | 10-19 years | CKD-T1DM | Low temperature | Percent | 2021 | 0.044685 | 0.070396 | 0.021449 |
| DALYs | Central Latin America | Female | 10-19 years | CKD-T1DM | Environmental/occupational risks | Percent | 2021 | 0.044415 | 0.052006 | 0.038912 |
| DALYs | High SDI | Both | 10-19 years | CKD-T1DM | Low temperature | Percent | 2021 | 0.0443 | 0.054091 | 0.03362 |
| DALYs | Central Latin America | Both | 10-19 years | CKD-T1DM | Environmental/occupational risks | Percent | 2021 | 0.043924 | 0.051222 | 0.038728 |
| DALYs | High-income Asia Pacific | Male | 10-19 years | CKD-T1DM | Low temperature | Percent | 2021 | 0.043898 | 0.059122 | 0.027911 |
| DALYs | Central Latin America | Male | 10-19 years | CKD-T1DM | Environmental/occupational risks | Percent | 2021 | 0.043453 | 0.050866 | 0.037625 |
| DALYs | High SDI | Female | 10-19 years | CKD-T1DM | Low temperature | Percent | 2021 | 0.042683 | 0.052786 | 0.032381 |
| DALYs | Eastern Sub-Saharan Africa | Female | 10-19 years | CKD-T1DM | Environmental/occupational risks | Percent | 2021 | 0.042457 | 0.048714 | 0.035815 |
| DALYs | South Asia | Both | 10-19 years | CKD-T1DM | Low temperature | Percent | 2021 | 0.042156 | 0.066082 | 0.020878 |
| DALYs | Eastern Sub-Saharan Africa | Both | 10-19 years | CKD-T1DM | Environmental/occupational risks | Percent | 2021 | 0.042139 | 0.048356 | 0.035853 |
| DALYs | High-middle SDI | Male | 10-19 years | CKD-T1DM | Low temperature | Percent | 2021 | 0.042109 | 0.050284 | 0.035012 |
| DALYs | Eastern Sub-Saharan Africa | Male | 10-19 years | CKD-T1DM | Environmental/occupational risks | Percent | 2021 | 0.04196 | 0.048269 | 0.035522 |
| DALYs | High-middle SDI | Both | 10-19 years | CKD-T1DM | Low temperature | Percent | 2021 | 0.041851 | 0.04862 | 0.035412 |
| DALYs | High-middle SDI | Female | 10-19 years | CKD-T1DM | Low temperature | Percent | 2021 | 0.041413 | 0.048996 | 0.034555 |
| DALYs | Low SDI | Female | 10-19 years | CKD-T1DM | Environmental/occupational risks | Percent | 2021 | 0.041407 | 0.053216 | 0.031557 |
| DALYs | Eastern Europe | Female | 10-19 years | CKD-T1DM | Environmental/occupational risks | Percent | 2021 | 0.04117 | 0.063618 | 0.022708 |
| DALYs | Central Latin America | Female | 10-19 years | CKD-T1DM | Low temperature | Percent | 2021 | 0.041168 | 0.051046 | 0.034922 |
| DALYs | Central Latin America | Both | 10-19 years | CKD-T1DM | Low temperature | Percent | 2021 | 0.040784 | 0.050635 | 0.034681 |
| DALYs | Eastern Europe | Female | 10-19 years | CKD-T1DM | Low temperature | Percent | 2021 | 0.040689 | 0.060822 | 0.023453 |
| DALYs | Central Latin America | Male | 10-19 years | CKD-T1DM | Low temperature | Percent | 2021 | 0.040411 | 0.050335 | 0.034089 |
| DALYs | Low SDI | Both | 10-19 years | CKD-T1DM | Environmental/occupational risks | Percent | 2021 | 0.04008 | 0.051106 | 0.030378 |
| DALYs | South Asia | Male | 10-19 years | CKD-T1DM | Low temperature | Percent | 2021 | 0.039872 | 0.062391 | 0.019779 |
| DALYs | Low SDI | Male | 10-19 years | CKD-T1DM | Environmental/occupational risks | Percent | 2021 | 0.039378 | 0.0506 | 0.029696 |
| DALYs | Eastern Sub-Saharan Africa | Female | 10-19 years | CKD-T1DM | Low temperature | Percent | 2021 | 0.038589 | 0.047834 | 0.033126 |
| DALYs | Eastern Sub-Saharan Africa | Both | 10-19 years | CKD-T1DM | Low temperature | Percent | 2021 | 0.038333 | 0.047493 | 0.032814 |
| DALYs | Eastern Sub-Saharan Africa | Male | 10-19 years | CKD-T1DM | Low temperature | Percent | 2021 | 0.038176 | 0.047489 | 0.032299 |
| DALYs | Global | Male | 10-19 years | CKD-T1DM | Environmental/occupational risks | Percent | 2021 | 0.037878 | 0.049869 | 0.027608 |
| DALYs | Global | Both | 10-19 years | CKD-T1DM | Environmental/occupational risks | Percent | 2021 | 0.037534 | 0.050036 | 0.02756 |
| DALYs | Global | Female | 10-19 years | CKD-T1DM | Environmental/occupational risks | Percent | 2021 | 0.037088 | 0.050713 | 0.026556 |
| DALYs | Low-middle SDI | Female | 10-19 years | CKD-T1DM | Environmental/occupational risks | Percent | 2021 | 0.036938 | 0.060078 | 0.01835 |
| DALYs | Middle SDI | Male | 10-19 years | CKD-T1DM | Environmental/occupational risks | Percent | 2021 | 0.03615 | 0.045564 | 0.027655 |
| DALYs | Low-middle SDI | Both | 10-19 years | CKD-T1DM | Environmental/occupational risks | Percent | 2021 | 0.036097 | 0.058056 | 0.018737 |
| DALYs | Low-middle SDI | Male | 10-19 years | CKD-T1DM | Environmental/occupational risks | Percent | 2021 | 0.035359 | 0.056733 | 0.019325 |
| DALYs | Middle SDI | Both | 10-19 years | CKD-T1DM | Environmental/occupational risks | Percent | 2021 | 0.035036 | 0.043608 | 0.027686 |
| DALYs | Eastern Europe | Both | 10-19 years | CKD-T1DM | Environmental/occupational risks | Percent | 2021 | 0.034071 | 0.05698 | 0.017191 |
| DALYs | Eastern Europe | Both | 10-19 years | CKD-T1DM | Low temperature | Percent | 2021 | 0.033684 | 0.054613 | 0.017538 |
| DALYs | Middle SDI | Female | 10-19 years | CKD-T1DM | Environmental/occupational risks | Percent | 2021 | 0.033612 | 0.042483 | 0.026461 |
| DALYs | Low SDI | Female | 10-19 years | CKD-T1DM | Low temperature | Percent | 2021 | 0.032342 | 0.037192 | 0.02759 |
| DALYs | Western Sub-Saharan Africa | Male | 10-19 years | CKD-T1DM | Environmental/occupational risks | Percent | 2021 | 0.03119 | 0.055526 | 0.015031 |
| DALYs | Low SDI | Both | 10-19 years | CKD-T1DM | Low temperature | Percent | 2021 | 0.030923 | 0.035646 | 0.026062 |
| DALYs | Western Sub-Saharan Africa | Both | 10-19 years | CKD-T1DM | Environmental/occupational risks | Percent | 2021 | 0.030874 | 0.055466 | 0.014972 |
| DALYs | South Asia | Female | 10-19 years | CKD-T1DM | High temperature | Percent | 2021 | 0.030702 | 0.066489 | 0.001216 |
| DALYs | Middle SDI | Male | 10-19 years | CKD-T1DM | Low temperature | Percent | 2021 | 0.030344 | 0.036566 | 0.025175 |
| DALYs | Low SDI | Male | 10-19 years | CKD-T1DM | Low temperature | Percent | 2021 | 0.030161 | 0.03567 | 0.025027 |
| DALYs | Western Sub-Saharan Africa | Female | 10-19 years | CKD-T1DM | Environmental/occupational risks | Percent | 2021 | 0.030127 | 0.052651 | 0.014289 |
| DALYs | North Africa and Middle East | Female | 10-19 years | CKD-T1DM | High temperature | Percent | 2021 | 0.030058 | 0.069081 | 0.001472 |
| DALYs | North Africa and Middle East | Both | 10-19 years | CKD-T1DM | High temperature | Percent | 2021 | 0.029744 | 0.068851 | 0.001498 |
| DALYs | Global | Male | 10-19 years | CKD-T1DM | Low temperature | Percent | 2021 | 0.029659 | 0.034766 | 0.025092 |
| DALYs | North Africa and Middle East | Male | 10-19 years | CKD-T1DM | High temperature | Percent | 2021 | 0.029409 | 0.06821 | 0.001152 |
| DALYs | Middle SDI | Both | 10-19 years | CKD-T1DM | Low temperature | Percent | 2021 | 0.029223 | 0.034134 | 0.025037 |
| DALYs | Global | Both | 10-19 years | CKD-T1DM | Low temperature | Percent | 2021 | 0.029047 | 0.033586 | 0.024859 |
| DALYs | Oceania | Male | 10-19 years | CKD-T1DM | Environmental/occupational risks | Percent | 2021 | 0.028732 | 0.036492 | 0.021705 |
| DALYs | South Asia | Both | 10-19 years | CKD-T1DM | High temperature | Percent | 2021 | 0.028608 | 0.061903 | 0.001153 |
| DALYs | Oceania | Male | 10-19 years | CKD-T1DM | Low temperature | Percent | 2021 | 0.028313 | 0.036547 | 0.021285 |
| DALYs | Global | Female | 10-19 years | CKD-T1DM | Low temperature | Percent | 2021 | 0.028238 | 0.033297 | 0.023755 |
| DALYs | Oceania | Both | 10-19 years | CKD-T1DM | Environmental/occupational risks | Percent | 2021 | 0.028186 | 0.035674 | 0.021546 |
| DALYs | Middle SDI | Female | 10-19 years | CKD-T1DM | Low temperature | Percent | 2021 | 0.027785 | 0.032308 | 0.023577 |
| DALYs | Oceania | Both | 10-19 years | CKD-T1DM | Low temperature | Percent | 2021 | 0.027759 | 0.036181 | 0.020762 |
| DALYs | Western Europe | Female | 10-19 years | CKD-T1DM | Environmental/occupational risks | Percent | 2021 | 0.027741 | 0.046269 | 0.013237 |
| DALYs | Oceania | Female | 10-19 years | CKD-T1DM | Environmental/occupational risks | Percent | 2021 | 0.027581 | 0.035468 | 0.020128 |
| DALYs | High-income North America | Male | 10-19 years | CKD-T1DM | Environmental/occupational risks | Percent | 2021 | 0.027475 | 0.04278 | 0.014224 |
| DALYs | Western Europe | Female | 10-19 years | CKD-T1DM | Low temperature | Percent | 2021 | 0.027398 | 0.045575 | 0.013225 |
| DALYs | Oceania | Female | 10-19 years | CKD-T1DM | Low temperature | Percent | 2021 | 0.027148 | 0.035039 | 0.019665 |
| DALYs | Eastern Europe | Male | 10-19 years | CKD-T1DM | Environmental/occupational risks | Percent | 2021 | 0.026879 | 0.048116 | 0.012267 |
| DALYs | South Asia | Male | 10-19 years | CKD-T1DM | High temperature | Percent | 2021 | 0.026726 | 0.05837 | 0.00111 |
| DALYs | Eastern Europe | Male | 10-19 years | CKD-T1DM | Low temperature | Percent | 2021 | 0.026587 | 0.045956 | 0.012589 |
| DALYs | Central Europe | Female | 10-19 years | CKD-T1DM | Environmental/occupational risks | Percent | 2021 | 0.025937 | 0.043335 | 0.011577 |
| DALYs | High-income North America | Male | 10-19 years | CKD-T1DM | Low temperature | Percent | 2021 | 0.025871 | 0.040457 | 0.013295 |
| DALYs | Central Europe | Female | 10-19 years | CKD-T1DM | Low temperature | Percent | 2021 | 0.025855 | 0.043096 | 0.011437 |
| DALYs | High-income North America | Both | 10-19 years | CKD-T1DM | Environmental/occupational risks | Percent | 2021 | 0.025758 | 0.040907 | 0.013256 |
| DALYs | High-income North America | Female | 10-19 years | CKD-T1DM | Environmental/occupational risks | Percent | 2021 | 0.02452 | 0.03975 | 0.012202 |
| DALYs | High-income North America | Both | 10-19 years | CKD-T1DM | Low temperature | Percent | 2021 | 0.024301 | 0.038079 | 0.012625 |
| DALYs | Western Sub-Saharan Africa | Male | 10-19 years | CKD-T1DM | High temperature | Percent | 2021 | 0.024066 | 0.044773 | 0.010974 |
| DALYs | Western Sub-Saharan Africa | Both | 10-19 years | CKD-T1DM | High temperature | Percent | 2021 | 0.023923 | 0.044876 | 0.010852 |
| DALYs | Western Sub-Saharan Africa | Female | 10-19 years | CKD-T1DM | High temperature | Percent | 2021 | 0.023612 | 0.043245 | 0.010596 |
| DALYs | High-income North America | Female | 10-19 years | CKD-T1DM | Low temperature | Percent | 2021 | 0.023169 | 0.037075 | 0.011959 |
| DALYs | Central Europe | Both | 10-19 years | CKD-T1DM | Environmental/occupational risks | Percent | 2021 | 0.022981 | 0.040933 | 0.00923 |
| DALYs | Australasia | Female | 10-19 years | CKD-T1DM | Environmental/occupational risks | Percent | 2021 | 0.02292 | 0.051424 | 0.005196 |
| DALYs | Western Europe | Both | 10-19 years | CKD-T1DM | Environmental/occupational risks | Percent | 2021 | 0.022918 | 0.041209 | 0.010894 |
| DALYs | Central Europe | Both | 10-19 years | CKD-T1DM | Low temperature | Percent | 2021 | 0.022897 | 0.040898 | 0.009257 |
| DALYs | Australasia | Female | 10-19 years | CKD-T1DM | Low temperature | Percent | 2021 | 0.022775 | 0.051613 | 0.005174 |
| DALYs | Western Europe | Both | 10-19 years | CKD-T1DM | Low temperature | Percent | 2021 | 0.02261 | 0.041336 | 0.011186 |
| DALYs | Low-middle SDI | Male | 10-19 years | CKD-T1DM | Low temperature | Percent | 2021 | 0.021963 | 0.030491 | 0.015683 |
| DALYs | Low-middle SDI | Both | 10-19 years | CKD-T1DM | Low temperature | Percent | 2021 | 0.021937 | 0.030265 | 0.015253 |
| DALYs | Low-middle SDI | Female | 10-19 years | CKD-T1DM | Low temperature | Percent | 2021 | 0.021933 | 0.03069 | 0.014649 |
| DALYs | Central Europe | Male | 10-19 years | CKD-T1DM | Environmental/occupational risks | Percent | 2021 | 0.021044 | 0.039868 | 0.008263 |
| DALYs | Australasia | Both | 10-19 years | CKD-T1DM | Environmental/occupational risks | Percent | 2021 | 0.020976 | 0.047416 | 0.004487 |
| DALYs | Central Europe | Male | 10-19 years | CKD-T1DM | Low temperature | Percent | 2021 | 0.020958 | 0.039949 | 0.008412 |
| DALYs | Australasia | Both | 10-19 years | CKD-T1DM | Low temperature | Percent | 2021 | 0.020824 | 0.047214 | 0.004422 |
| DALYs | Tropical Latin America | Male | 10-19 years | CKD-T1DM | Environmental/occupational risks | Percent | 2021 | 0.019363 | 0.024776 | 0.015208 |
| DALYs | Western Europe | Male | 10-19 years | CKD-T1DM | Environmental/occupational risks | Percent | 2021 | 0.019277 | 0.036542 | 0.008619 |
| DALYs | Tropical Latin America | Both | 10-19 years | CKD-T1DM | Environmental/occupational risks | Percent | 2021 | 0.019069 | 0.024645 | 0.014684 |
| DALYs | Western Europe | Male | 10-19 years | CKD-T1DM | Low temperature | Percent | 2021 | 0.018995 | 0.036062 | 0.008601 |
| DALYs | Australasia | Male | 10-19 years | CKD-T1DM | Environmental/occupational risks | Percent | 2021 | 0.018979 | 0.042991 | 0.003539 |
| DALYs | Australasia | Male | 10-19 years | CKD-T1DM | Low temperature | Percent | 2021 | 0.018818 | 0.042936 | 0.003516 |
| DALYs | Tropical Latin America | Female | 10-19 years | CKD-T1DM | Environmental/occupational risks | Percent | 2021 | 0.018606 | 0.023999 | 0.013969 |
| DALYs | Tropical Latin America | Male | 10-19 years | CKD-T1DM | Low temperature | Percent | 2021 | 0.018338 | 0.021422 | 0.015205 |
| DALYs | Tropical Latin America | Both | 10-19 years | CKD-T1DM | Low temperature | Percent | 2021 | 0.018078 | 0.021162 | 0.014649 |
| DALYs | Tropical Latin America | Female | 10-19 years | CKD-T1DM | Low temperature | Percent | 2021 | 0.01767 | 0.021331 | 0.013864 |
| DALYs | Low-middle SDI | Female | 10-19 years | CKD-T1DM | High temperature | Percent | 2021 | 0.015807 | 0.03263 | 0.003715 |
| DALYs | Low-middle SDI | Both | 10-19 years | CKD-T1DM | High temperature | Percent | 2021 | 0.014889 | 0.030279 | 0.003444 |
| DALYs | Southeast Asia | Female | 10-19 years | CKD-T1DM | Environmental/occupational risks | Percent | 2021 | 0.01471 | 0.021335 | 0.01052 |
| DALYs | High SDI | Female | 10-19 years | CKD-T1DM | High temperature | Percent | 2021 | 0.014371 | 0.037434 | -0.00287 |
| DALYs | Southeast Asia | Both | 10-19 years | CKD-T1DM | Environmental/occupational risks | Percent | 2021 | 0.014222 | 0.020273 | 0.010304 |
| DALYs | Low-middle SDI | Male | 10-19 years | CKD-T1DM | High temperature | Percent | 2021 | 0.014059 | 0.028769 | 0.003158 |
| DALYs | Southeast Asia | Male | 10-19 years | CKD-T1DM | Environmental/occupational risks | Percent | 2021 | 0.013782 | 0.020148 | 0.009928 |
| DALYs | Central Sub-Saharan Africa | Male | 10-19 years | CKD-T1DM | Environmental/occupational risks | Percent | 2021 | 0.013126 | 0.018341 | 0.008499 |
| DALYs | Central Sub-Saharan Africa | Both | 10-19 years | CKD-T1DM | Environmental/occupational risks | Percent | 2021 | 0.013027 | 0.018142 | 0.008449 |
| DALYs | Central Sub-Saharan Africa | Female | 10-19 years | CKD-T1DM | Environmental/occupational risks | Percent | 2021 | 0.012793 | 0.017531 | 0.007893 |
| DALYs | High SDI | Both | 10-19 years | CKD-T1DM | High temperature | Percent | 2021 | 0.012788 | 0.03352 | -0.00368 |
| DALYs | Central Sub-Saharan Africa | Male | 10-19 years | CKD-T1DM | Low temperature | Percent | 2021 | 0.012388 | 0.015174 | 0.009903 |
| DALYs | Central Sub-Saharan Africa | Both | 10-19 years | CKD-T1DM | Low temperature | Percent | 2021 | 0.012309 | 0.015099 | 0.009664 |
| DALYs | Central Sub-Saharan Africa | Female | 10-19 years | CKD-T1DM | Low temperature | Percent | 2021 | 0.012119 | 0.014793 | 0.008992 |
| DALYs | High SDI | Male | 10-19 years | CKD-T1DM | High temperature | Percent | 2021 | 0.011343 | 0.031643 | -0.00414 |
| DALYs | Low SDI | Male | 10-19 years | CKD-T1DM | High temperature | Percent | 2021 | 0.009425 | 0.020247 | 0.001919 |
| DALYs | Low SDI | Both | 10-19 years | CKD-T1DM | High temperature | Percent | 2021 | 0.009386 | 0.019936 | 0.001865 |
| DALYs | Low SDI | Female | 10-19 years | CKD-T1DM | High temperature | Percent | 2021 | 0.009332 | 0.020212 | 0.001694 |
| DALYs | Global | Female | 10-19 years | CKD-T1DM | High temperature | Percent | 2021 | 0.00925 | 0.020064 | 0.00143 |
| DALYs | Global | Both | 10-19 years | CKD-T1DM | High temperature | Percent | 2021 | 0.008845 | 0.019213 | 0.001327 |
| DALYs | Global | Male | 10-19 years | CKD-T1DM | High temperature | Percent | 2021 | 0.008546 | 0.018607 | 0.001038 |
| DALYs | Southeast Asia | Female | 10-19 years | CKD-T1DM | Low temperature | Percent | 2021 | 0.007841 | 0.009843 | 0.005812 |
| DALYs | Southeast Asia | Both | 10-19 years | CKD-T1DM | Low temperature | Percent | 2021 | 0.007629 | 0.009496 | 0.005601 |
| DALYs | Southeast Asia | Male | 10-19 years | CKD-T1DM | Low temperature | Percent | 2021 | 0.007434 | 0.009294 | 0.005452 |
| DALYs | Western Sub-Saharan Africa | Male | 10-19 years | CKD-T1DM | Low temperature | Percent | 2021 | 0.007402 | 0.011839 | 0.004176 |
| DALYs | Western Sub-Saharan Africa | Both | 10-19 years | CKD-T1DM | Low temperature | Percent | 2021 | 0.007224 | 0.011604 | 0.004099 |
| DALYs | Southeast Asia | Female | 10-19 years | CKD-T1DM | High temperature | Percent | 2021 | 0.006957 | 0.011965 | 0.003993 |
| DALYs | Caribbean | Male | 10-19 years | CKD-T1DM | Environmental/occupational risks | Percent | 2021 | 0.006944 | 0.009877 | 0.004345 |
| DALYs | Western Sub-Saharan Africa | Female | 10-19 years | CKD-T1DM | Low temperature | Percent | 2021 | 0.00678 | 0.011227 | 0.00374 |
| DALYs | Southeast Asia | Both | 10-19 years | CKD-T1DM | High temperature | Percent | 2021 | 0.006677 | 0.011268 | 0.003918 |
| DALYs | Caribbean | Both | 10-19 years | CKD-T1DM | Environmental/occupational risks | Percent | 2021 | 0.006644 | 0.009568 | 0.004161 |
| DALYs | Southeast Asia | Male | 10-19 years | CKD-T1DM | High temperature | Percent | 2021 | 0.006428 | 0.010986 | 0.00376 |
| DALYs | Central Asia | Female | 10-19 years | CKD-T1DM | High temperature | Percent | 2021 | 0.006098 | 0.016695 | -0.00297 |
| DALYs | Middle SDI | Female | 10-19 years | CKD-T1DM | High temperature | Percent | 2021 | 0.006051 | 0.013042 | 0.001119 |
| DALYs | Middle SDI | Both | 10-19 years | CKD-T1DM | High temperature | Percent | 2021 | 0.006038 | 0.012826 | 0.000919 |
| DALYs | Middle SDI | Male | 10-19 years | CKD-T1DM | High temperature | Percent | 2021 | 0.006032 | 0.013204 | 0.000871 |
| DALYs | Central Asia | Both | 10-19 years | CKD-T1DM | High temperature | Percent | 2021 | 0.005979 | 0.016519 | -0.00281 |
| DALYs | East Asia | Male | 10-19 years | CKD-T1DM | High temperature | Percent | 2021 | 0.005968 | 0.020267 | -0.00657 |
| DALYs | East Asia | Both | 10-19 years | CKD-T1DM | High temperature | Percent | 2021 | 0.005907 | 0.020145 | -0.0064 |
| DALYs | Central Asia | Male | 10-19 years | CKD-T1DM | High temperature | Percent | 2021 | 0.005907 | 0.016244 | -0.00277 |
| DALYs | East Asia | Female | 10-19 years | CKD-T1DM | High temperature | Percent | 2021 | 0.005815 | 0.019523 | -0.00621 |
| DALYs | Caribbean | Female | 10-19 years | CKD-T1DM | Environmental/occupational risks | Percent | 2021 | 0.005756 | 0.008765 | 0.003455 |
| DALYs | High-middle SDI | Male | 10-19 years | CKD-T1DM | High temperature | Percent | 2021 | 0.005729 | 0.017601 | -0.00396 |
| DALYs | High-middle SDI | Both | 10-19 years | CKD-T1DM | High temperature | Percent | 2021 | 0.005702 | 0.01744 | -0.00374 |
| DALYs | High-middle SDI | Female | 10-19 years | CKD-T1DM | High temperature | Percent | 2021 | 0.005657 | 0.016884 | -0.00358 |
| DALYs | Caribbean | Male | 10-19 years | CKD-T1DM | Low temperature | Percent | 2021 | 0.005586 | 0.006671 | 0.004249 |
| DALYs | Caribbean | Both | 10-19 years | CKD-T1DM | Low temperature | Percent | 2021 | 0.005333 | 0.006313 | 0.004143 |
| DALYs | Caribbean | Female | 10-19 years | CKD-T1DM | Low temperature | Percent | 2021 | 0.004562 | 0.005417 | 0.003743 |
| DALYs | Eastern Sub-Saharan Africa | Female | 10-19 years | CKD-T1DM | High temperature | Percent | 2021 | 0.003938 | 0.009562 | 0.000215 |
| DALYs | Eastern Sub-Saharan Africa | Both | 10-19 years | CKD-T1DM | High temperature | Percent | 2021 | 0.003879 | 0.00938 | 2.41E-05 |
| DALYs | Eastern Sub-Saharan Africa | Male | 10-19 years | CKD-T1DM | High temperature | Percent | 2021 | 0.003857 | 0.009375 | -8.22E-05 |
| DALYs | Central Latin America | Female | 10-19 years | CKD-T1DM | High temperature | Percent | 2021 | 0.003397 | 0.008295 | -0.00034 |
| DALYs | Central Latin America | Both | 10-19 years | CKD-T1DM | High temperature | Percent | 2021 | 0.003286 | 0.008027 | -0.00034 |
| DALYs | Central Latin America | Male | 10-19 years | CKD-T1DM | High temperature | Percent | 2021 | 0.003186 | 0.007834 | -0.00037 |
| DALYs | High-income North America | Male | 10-19 years | CKD-T1DM | High temperature | Percent | 2021 | 0.001712 | 0.007171 | -0.00218 |
| DALYs | High-income North America | Both | 10-19 years | CKD-T1DM | High temperature | Percent | 2021 | 0.001557 | 0.006618 | -0.00208 |
| DALYs | High-income North America | Female | 10-19 years | CKD-T1DM | High temperature | Percent | 2021 | 0.001443 | 0.006126 | -0.00196 |
| DALYs | Caribbean | Male | 10-19 years | CKD-T1DM | High temperature | Percent | 2021 | 0.001367 | 0.003799 | -0.00058 |
| DALYs | Caribbean | Both | 10-19 years | CKD-T1DM | High temperature | Percent | 2021 | 0.001319 | 0.003656 | -0.0006 |
| DALYs | Southern Sub-Saharan Africa | Female | 10-19 years | CKD-T1DM | High temperature | Percent | 2021 | 0.001313 | 0.006177 | -0.00258 |
| DALYs | Caribbean | Female | 10-19 years | CKD-T1DM | High temperature | Percent | 2021 | 0.001202 | 0.00356 | -0.00058 |
| DALYs | Southern Sub-Saharan Africa | Both | 10-19 years | CKD-T1DM | High temperature | Percent | 2021 | 0.001192 | 0.005787 | -0.0024 |
| DALYs | High-income Asia Pacific | Female | 10-19 years | CKD-T1DM | High temperature | Percent | 2021 | 0.001168 | 0.005954 | -0.00361 |
| DALYs | Southern Sub-Saharan Africa | Male | 10-19 years | CKD-T1DM | High temperature | Percent | 2021 | 0.001117 | 0.005638 | -0.00227 |
| DALYs | High-income Asia Pacific | Both | 10-19 years | CKD-T1DM | High temperature | Percent | 2021 | 0.001102 | 0.005971 | -0.00363 |
| DALYs | Southern Latin America | Male | 10-19 years | CKD-T1DM | High temperature | Percent | 2021 | 0.001078 | 0.004542 | -0.00138 |
| DALYs | Tropical Latin America | Male | 10-19 years | CKD-T1DM | High temperature | Percent | 2021 | 0.00106 | 0.004355 | -0.00118 |
| DALYs | Southern Latin America | Both | 10-19 years | CKD-T1DM | High temperature | Percent | 2021 | 0.001053 | 0.004505 | -0.00131 |
| DALYs | High-income Asia Pacific | Male | 10-19 years | CKD-T1DM | High temperature | Percent | 2021 | 0.001029 | 0.005645 | -0.00364 |
| DALYs | Southern Latin America | Female | 10-19 years | CKD-T1DM | High temperature | Percent | 2021 | 0.001029 | 0.004367 | -0.00128 |
| DALYs | Tropical Latin America | Both | 10-19 years | CKD-T1DM | High temperature | Percent | 2021 | 0.001025 | 0.004359 | -0.00119 |
| DALYs | Tropical Latin America | Female | 10-19 years | CKD-T1DM | High temperature | Percent | 2021 | 0.00097 | 0.004336 | -0.00124 |
| DALYs | Central Sub-Saharan Africa | Male | 10-19 years | CKD-T1DM | High temperature | Percent | 2021 | 0.000746 | 0.005922 | -0.00388 |
| DALYs | Central Sub-Saharan Africa | Both | 10-19 years | CKD-T1DM | High temperature | Percent | 2021 | 0.000725 | 0.005858 | -0.00377 |
| DALYs | Central Sub-Saharan Africa | Female | 10-19 years | CKD-T1DM | High temperature | Percent | 2021 | 0.00068 | 0.005699 | -0.00364 |
| DALYs | Andean Latin America | Female | 10-19 years | CKD-T1DM | High temperature | Percent | 2021 | 0.000631 | 0.002422 | -0.00079 |
| DALYs | Andean Latin America | Both | 10-19 years | CKD-T1DM | High temperature | Percent | 2021 | 0.000596 | 0.002346 | -0.00077 |
| DALYs | Andean Latin America | Male | 10-19 years | CKD-T1DM | High temperature | Percent | 2021 | 0.00057 | 0.002245 | -0.00078 |
| DALYs | Eastern Europe | Female | 10-19 years | CKD-T1DM | High temperature | Percent | 2021 | 0.000529 | 0.004193 | -0.00355 |
| DALYs | Oceania | Female | 10-19 years | CKD-T1DM | High temperature | Percent | 2021 | 0.000444 | 0.000933 | -0.00032 |
| DALYs | Oceania | Both | 10-19 years | CKD-T1DM | High temperature | Percent | 2021 | 0.000438 | 0.000936 | -0.00031 |
| DALYs | Oceania | Male | 10-19 years | CKD-T1DM | High temperature | Percent | 2021 | 0.000431 | 0.000948 | -0.00031 |
| DALYs | Eastern Europe | Both | 10-19 years | CKD-T1DM | High temperature | Percent | 2021 | 0.000426 | 0.003487 | -0.00296 |
| DALYs | Western Europe | Female | 10-19 years | CKD-T1DM | High temperature | Percent | 2021 | 0.000363 | 0.00184 | -0.00065 |
| DALYs | Western Europe | Both | 10-19 years | CKD-T1DM | High temperature | Percent | 2021 | 0.000325 | 0.001571 | -0.00056 |
| DALYs | Eastern Europe | Male | 10-19 years | CKD-T1DM | High temperature | Percent | 2021 | 0.000322 | 0.002814 | -0.00245 |
| DALYs | Western Europe | Male | 10-19 years | CKD-T1DM | High temperature | Percent | 2021 | 0.000297 | 0.001474 | -0.00048 |
| DALYs | Australasia | Male | 10-19 years | CKD-T1DM | High temperature | Percent | 2021 | 0.000172 | 0.000981 | -0.00027 |
| DALYs | Australasia | Both | 10-19 years | CKD-T1DM | High temperature | Percent | 2021 | 0.000162 | 0.000947 | -0.00027 |
| DALYs | Australasia | Female | 10-19 years | CKD-T1DM | High temperature | Percent | 2021 | 0.000155 | 0.000893 | -0.00028 |
| DALYs | Central Europe | Male | 10-19 years | CKD-T1DM | High temperature | Percent | 2021 | 9.26E-05 | 0.001463 | -0.00139 |
| DALYs | Central Europe | Both | 10-19 years | CKD-T1DM | High temperature | Percent | 2021 | 9.07E-05 | 0.001523 | -0.0015 |
| DALYs | Central Europe | Female | 10-19 years | CKD-T1DM | High temperature | Percent | 2021 | 8.82E-05 | 0.001641 | -0.00165 |
